# A Simple and General Platform for Generating Stereochemically Complex Polyene Frameworks by Iterative Cross-Coupling**

**DOI:** 10.1002/anie.201004911

**Published:** 2010-10-06

**Authors:** Suk Joong Lee, Thomas M Anderson, Martin D Burke

**Affiliations:** Howard Hughes Medical Institute, Department of Chemistry, University of Illinois at Urbana-Champaign600 S. Mathews Ave, Urbana, IL 61801 (USA)

**Keywords:** haloboronic acids, iterative cross-coupling, MIDA boronates, polyenes, vacidin A

Aiming to maximally harness the inherent modularity of small molecules, we are pursuing the development of a synthesis strategy based on the iterative cross-coupling (ICC) of bifunctional building blocks representing the substructures that most commonly appear in natural products.^1^, ^2^ In this vein, various combinations of *trans*- and *cis*-olefins are found in many small molecules derived from a wide range of biosynthetic pathways, including polyketides, hybrid peptide/polyketides, polyterpines, and fatty acids (Scheme 1).^3^, ^4^ Enabling stereospecific access to these stereochemically complex polyene frameworks, we herein describe the development of a novel ICC platform that yields bifunctional iodopolyenyl *N*-methyliminodiacetic acid (MIDA) boronates in all possible stereoisomeric forms. The power of this approach has been realized in the first synthesis of the highly complex (*E*,*E*,*E*,*Z*,*Z*,*E*,*E*)-heptaene framework of the ion channel-forming polyene macrolide vacidin A.

**Scheme 1:**
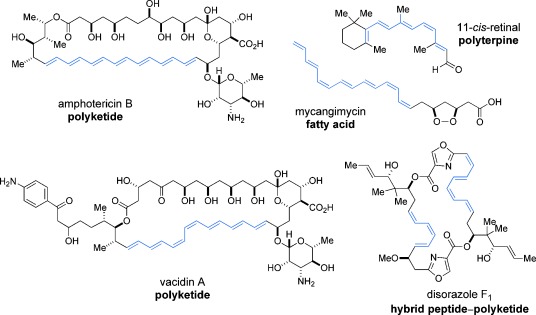
Polyene natural products derived from a wide range of biosynthetic pathways.

We recently reported three haloalkenyl MIDA boronates that enabled the preparation of a subset of polyene motifs by ICC.2b Albeit an important step forward, this collection only provided access to all-*trans*-polyene substructures and utilized polyenylchlorides. Accessing stereochemically complex polyene motifs is substantially more challenging because, in addition to the sensitivities observed with all polyenes to light, oxygen, and acid, frameworks containing *cis* double bonds can isomerize to the typically more thermodynamically stable all-*trans* structures. Moreover, poorly reactive polyenylchlorides proved to be minimally effective in complex applications.

To overcome both of these limitations, we pursued a novel strategy for making iodopolyenyl MIDA boronates by ICC of iodide-masked^5^ bifunctional building blocks. As shown in Scheme 2 A, the approach involves metal-selective cross-coupling of Sn/Ge bis-metalated olefins^6^ to generate polyenylgermanium intermediates followed by stereospecific iododegermylations.^7^ To the best of our knowledge, iododegermylations of polyenylgermanium species were unreported. However, the facility of halodegermylation^7^ vs. halodesilylation^8^ of simple olefins suggested that the former process had superior potential to be efficient and stereoretentive in the context of structurally and stereochemically complex polyene systems. Guided by this logic, we hypothesized that iterative cycles of metal-selective coupling/iododegermylation with core building blocks **1** and **2** (Scheme 2 B) could provide access to iodopolyenyl MIDA boronates in all possible stereoisomeric forms (Scheme 2 C).

**Scheme 2:**
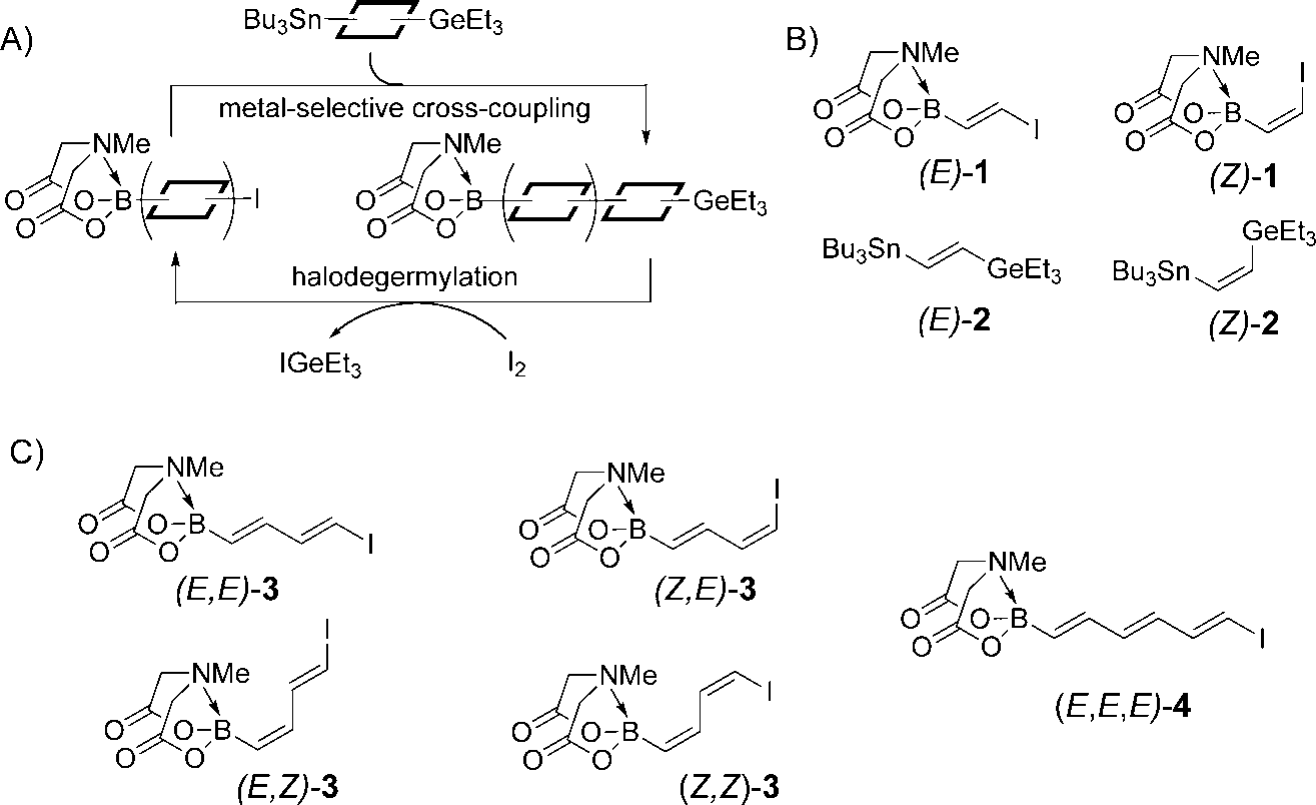
A) A strategy for ICC of halogen-masked bifunctional building blocks. B) Core building blocks to enable general access to stereoisomeric iodopolyenyl MIDA boronates. C) New iodopolyenyl MIDA boronates for the synthesis of polyene natural products.

We discovered that (*E*)-**1** and (*Z*)-**1** can both be generated from the novel ethynyl MIDA boronate **6**, which in turn can be prepared from readily available Grignard reagent **5**.2h, 9 Specifically, as shown in Scheme 3, the addition of **5** to trimethyl borate followed by direct transligation of the resulting magnesium ate complex with MIDA generated **6** as a colorless, crystalline solid. When this transligation was executed at 115 °C,2f a very good yield of **6** was achieved on the decagram scale. Although **6** is fully compatible with silica gel chromatography, this highly versatile^10^ new building block can also be conveniently isolated in excellent purity by recrystallization. One-pot hydrostannylation^14^ of **6** followed by iododestannylation of the resulting bis-metalated intermediate provided an excellent yield of (*E*)-**1**. Alternatively subjecting **6** to a series of silver-promoted alkyne iodination^12^ followed by PADC-mediated semireduction^13^ provided the complementary building block (*Z*)-**1**. Importantly, both (*E*)-**1** and (*Z*)-**1** are air- and chromatographically stable, highly crystalline free-flowing solids.

**Scheme 3:**
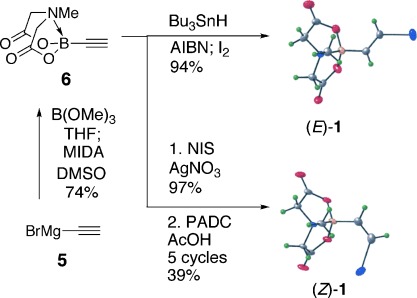
Synthesis of bifunctional MIDA boronate building blocks (*E*)-1 and (*Z*)-1 from the common intermediate ethynyl MIDA boronate 6. Color code: red, O; gray, C; green, H; yellow, B; light blue, N; dark blue, I. DMSO=dimethyl sulfoxide, AIBN=azobisisobutyronitrile, NIS=*N*-iodosuccinimide, PADC=potassium azodicarboxylate.

A synthesis of (*E*)-**2** has been previously reported,^6^ but a stereocontrolled route to (*Z*)-**2** was unknown.^14^ As shown in Scheme 4, hybridizing methodology previously reported for the germylstannylation of substituted alkynes^15^ and the silylstannylation of acetylene,^16^ core building block (*Z*)-**2** was efficiently prepared as a single stereoisomer by the palladium-mediated *cis*-germylstannylation of acetylene gas.

**Scheme 4:**
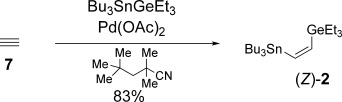
The stereocontrolled preparation of (*Z*)-2.

With these four core building blocks in hand, we sought general conditions for efficient cycles of stereospecific metal-selective couplings and iododegermylations (Scheme 2 A). As shown in Scheme 5, we found that Liebeskind-type conditions^17^ are remarkably effective for the targeted metal-selective Stille couplings. In fact, using the exact same set of very mild conditions ([Pd(PPh_3_)_4_]/CuTC, DMF, 0 °C to 23 °C), all possible combinations of **1** and **2** were stereospecifically coupled in excellent yields to generate dienylgermanium intermediates **8**. Completing the envisioned cycle, stereospecific iododegermylations of all four of these intermediates were readily achieved by treatment with I_2_ in MeOH at −78 °C, thereby providing all of the targeted iododienyl MIDA boronate building blocks **3** in good yields and as single stereoisomers. Harnessing the iterative nature of this strategy, the more advanced trienyl halide (*E*,*E*,*E*)-**4** was also readily prepared by simply executing an additional cycle of metal-selective coupling and stereospecific iododegermylation (Scheme 6).

**Scheme 5:**
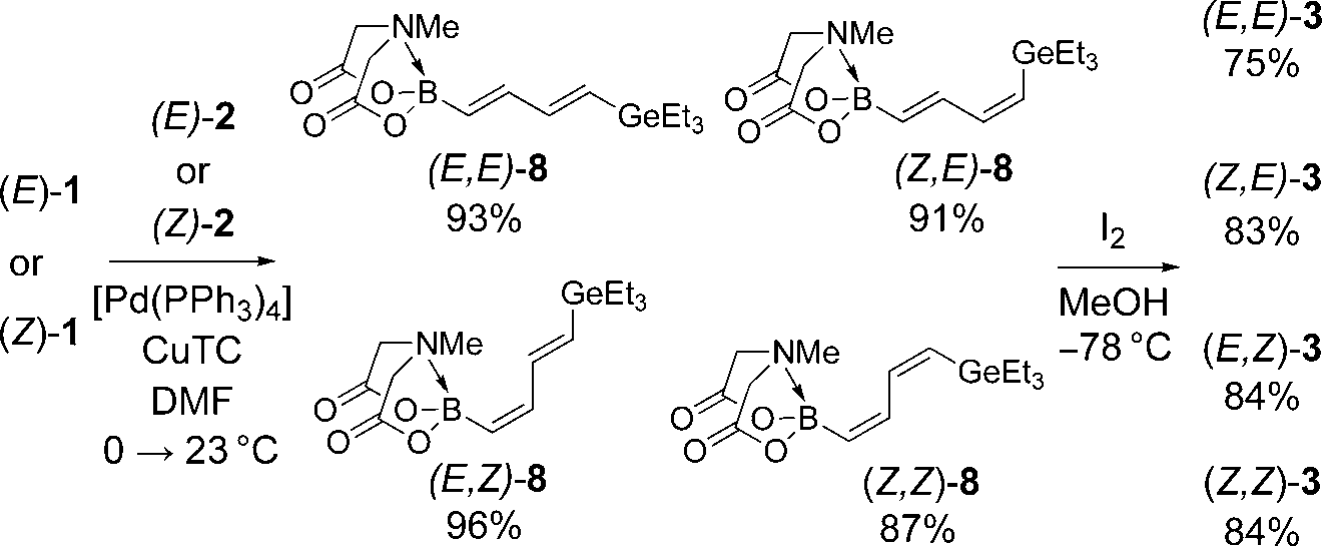
Efficient and stereospecific syntheses of all possible stereoisomers of 3 by metal-selective ICC. TC=thiophene-2-carboxylate.

**Scheme 6:**
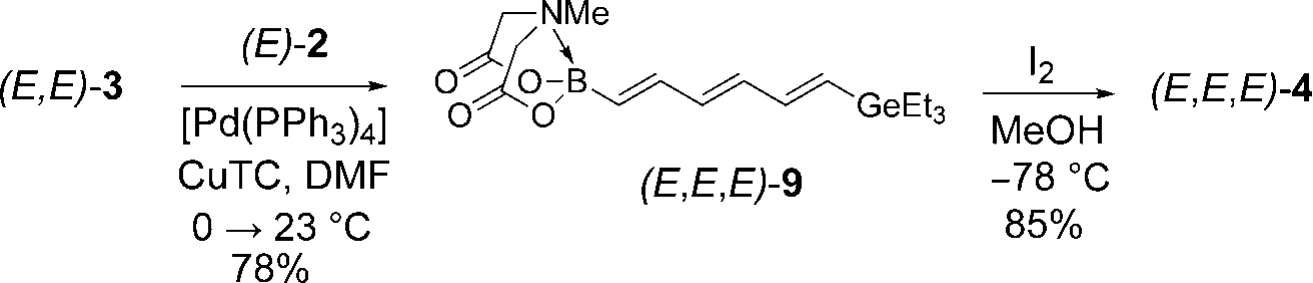
Preparation of iodotrienyl MIDA boronate *(E*,*E*,*E)*-4 by metal-selective ICC.

As shown in Table 1, these new bifunctional building blocks collectively enable the preparation of a broad range of stereochemically complex polyene natural product frameworks. After surveying a variety of catalysts, bases, and solvents we found a very mild set of Buchwald-type^18^ cross-coupling conditions [Pd(OAc)_2_, SPhos or XPhos, Cs_2_CO_3_, THF, 23 °C] that proved to be highly effective. Specifically, all possible stereoisomers of 1 and 3 were cross-coupled with both (*E*)- and (*Z*)-pentenyl boronic acid 10 in good to excellent yields and with outstanding levels of stereoretention.^19^ Observations of complete stereoretention even when coupling the sterically encumbered MIDA boronate (*Z*)-1 (entries 2 and 8) and preparing the very challenging (*Z*,*Z*,*Z*)-triene 22 (entry 10) are particularly notable. Collectively, products 11–22 represent all possible stereoisomers of the core dienyl and trienyl substructures that appear in a wide range of natural products derived from all major biosynthetic pathways (see Scheme 1). Importantly, these products all retain the potential for subsequent cross-coupling reactions upon hydrolysis of the MIDA boronate functional group with mild, aqueous base.

**Table 1:** Stereospecific Suzuki–Miyaura cross-couplings yielding all possible stereoisomers of di- and trienyl MIDA boronates.^[a]^

Entry	Boronic acid	Iodoalkenyl MIDA boronate	Product	Yield [%]
1	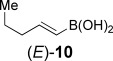	(*E*)-**1**	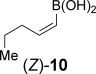	**11**	95
2	(*E*)-**10**	(*Z*)-**1**	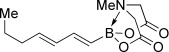	**12**	77
3	(*E*)-**10**	(*E*,*E*)-**3**	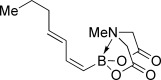	**13**	75
4	(*E*)-**10**	(*E*,*Z*)-**3**	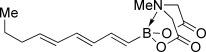	**14**	78
5	(*E*)-**10**	(*Z*,*E*)-**3**	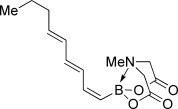	**15**	87
6	(*E*)-**10**	(*Z*,*Z*)-**3**	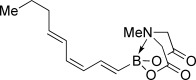	**16**	64
7	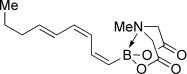	(*E*)-**1**	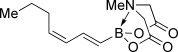	**17**	91
8	(*Z*)-**10**	(*Z*)-**1**	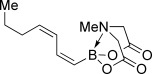	**18**	74
9	(*Z*)-**10**	(*E*,*E*)-**3**	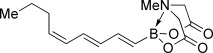	**19**	77
10	(*Z*)-**10**	(*E*,*Z*)-**3**	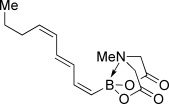	**20**	84
11	(*Z*)-**10**	(*Z*,*E*)-**3**	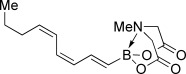	**21**	82
12	(*Z*)-**10**	(*Z*,*Z*)-**3**	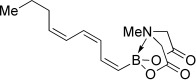	**22**	62

[a] 1.0 equiv **1** or **3**, 1.5 equiv **10**, Pd(OAc)_2_, SPhos (entries 1, 3, 4, 7, 9, 10) or XPhos (entries 2, 5, 6, 8, 11, 12), Cs_2_CO_3_, THF, 23 °C.

As a final test for this new platform, we targeted an ICC-based synthesis of the stereochemically complex polyene core of the exceptionally potent ion channel forming natural product vacidin A (Scheme 1).^20^ Interestingly, and in contrast to almost all other known polyene macrolide antibiotics, vacidin A contains two *cis* double bonds embedded within an otherwise all-*trans* heptaene framework. As shown in Scheme 7, hydrolysis of cross-coupling product **13** (Table 1, entry 3) with aqueous base followed by coupling of the resulting (*E*,*E*,*E*)-trienylboronic acid to iododienyl MIDA boronate (*Z,Z*)-**3** under anhydrous conditions yielded the desired (*Z,Z,E,E,E*)-pentaene **23**. Avoiding the need to isolate the corresponding highly unstable boronic acid, a final one-pot MIDA hydrolysis and cross-coupling2d with dienyl iodide **24**^21^ completed the first synthesis of the vacidin A (*E*,*E*,*E*,*Z*,*Z*,*E*,*E*)-heptaene framework **25**.^22^ Demonstrating the powerful simplicity of the ICC approach, this highly complex polyene motif was generated using only a single reaction to unite a collection of building blocks in which all of the required stereochemical relationships were pre-installed.

**Scheme 7:**
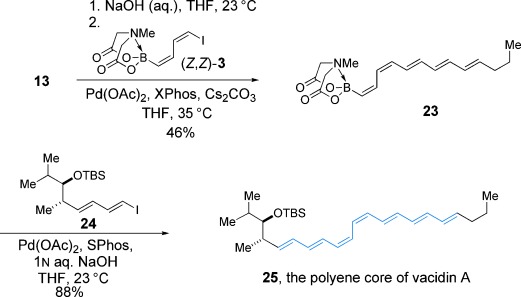
Synthesis of the stereochemically complex heptaene core of vacidin A.

The metal-selective coupling/iododegermylation strategy described herein provides access to a wide range of useful building blocks for the synthesis of complex polyene motifs. Overcoming previous limitations, this platform enables stereospecific preparation of polyenes in all possible stereoisomeric forms. These new building blocks represent important additions to a growing collection of MIDA boronates designed to support the development of a simple and flexible platform for the efficient synthesis of small molecules by ICC.^2^

## Notes

Detailed facts of importance to specialist readers are published as ”Supporting Information”. Such documents are peer-reviewed, but not copy-edited or typeset. They are made available as submitted by the authors.
