# Supplementary material for: A Simple and General Platform for Generating Stereochemically Complex Polyene Frameworks by Iterative Cross-Coupling
Source: Angew Chem Int Ed Engl. 2010 Oct 6;49(47):8860–3. doi: 10.1002/anie.201004911 (PMC3037596; doi:10.1002/anie.201004911)

Angewandte Chemie
Eine Zeitschrift der Gesellschaft Deutscher Chemiker

Supporting Information

© Wiley-VCH 2010

69451 Weinheim, Germany

A Simple and General Platform for Generating Stereochemically Complex Polyene Frameworks by Iterative Cross-Coupling**

*Suk Joong Lee, Thomas M. Anderson, and Martin D. Burke**

anie_201004911_sm_miscellaneous_information.pdf

SUPPORTING INFORMATION

Part A

I.	General Methods	S-1
II.	Synthesis of 1-Ethynylboronate ester **6** (Scheme 1)	S-3
III.	Synthesis of (***E***)-**1**, (***Z***)-**1** and (***Z***)-**2** (Schemes 1 and 2)	S-4
IV.	Syntheses of **3** (Scheme 3)	S-9
V.	Synthesis of **4** (Scheme 4)	S-15
VI.	Suzuki-Miyaura Cross-Couplings (**11**–**22**) (Table 1)	S-18
VII.	Synthesis of the Heptaene Core of Vacidin A **25** (Scheme 5)	S-28

Part B

VIII. NMR Spectra (pdf file)

Part C

IX. X-Ray Data for **6**, (***E***)-**1** and (***Z***)-**1** (cif files)

I. General Methods

Materials Commercial reagents were purchased from Sigma-Aldrich, Strem, Fisher Scientific, or Smith and were used without further purification unless otherwise noted. Solvents were purified *via* passage through packed columns as described by Pangborn and coworkers[1] (THF, Et_2O, CH_3CN, CH_2Cl_2: dry neutral alumina; hexane, benzene, and toluene: dry neutral alumina and Q5 reactant; DMSO, DMF: activated molecular sieves). Triethylamine and 2,6-lutidine were freshly distilled under an atmosphere of nitrogen from CaH_2. The following compounds were prepared according to literature precedent: (*E*)-1-(tributylstannyl)-2-(triethylgermanium)ethylene (***E***)-**2**[2], (1-pentyn-1-yl)diisopropyloxyborane[3], aldehyde **26**[4], potassium azodicarboxylate (PADC)[5].

General Experimental Procedures All palladium-mediated Suzuki-Miyaura cross-coupling reactions were set up in an argon-filled Innovative Technologies glove box and performed under an atmosphere of argon or nitrogen in oven- or flame-dried I-Chem or Wheaton vials sealed with PTFE-lined plastic caps unless otherwise indicated. All other reactions were performed in oven- or flame-dried round-bottom or modified Schlenk flasks fitted with rubber septa under a positive pressure of nitrogen unless otherwise indicated. Organic solutions were concentrated *via* rotary evaporation under reduced pressure. Reactions were monitored by analytical thin layer chromatography (TLC) performed using the indicated solvent on E. Merck silica gel 60 F254 plates (0.25 mm) or Florisil® 254 nm plate. Compounds were visualized by exposure to a UV lamp (λ = 254 or 365 nm), a solution of $KMnO_4$, or a solution of ceric ammonium molybdate (CAM) followed by brief heating using a Varitemp heat gun. Flash column chromatography was performed as described by Still and coworkers[6] using EM Merck silica gel 60 (230-400 mesh) and/or Fisher Scientific Florisil® (an activated magnesium silicate: 100-200 mesh).

Structural Analysis ^{1}H NMR spectra were recorded at 23 °C on one of the following instruments: Varian Unity 400, Varian Unity 500, Varian Unity Inova 500NB, Varian Unity Inova 600. Chemical

[1] Pangborn, A. B.; Giardello, M. A.; Grubbs, R. H.; Rosen, R. K.; Timmers, F. J. *Organometallics* **1996**, *15*, 1518-1520
[2] David-Quillot, F.; Thibonnet, J.; Marsacq, D.; Abarbri, M.; Duchêne, A. *Tetrahedron Lett.* **2000**, *41*, 9981-9984
[3] Micalizio, G.C.; Schreiber, S.L. *Angew. Chem. Int. Ed.* **2002**, *41*, 3272-3276
[4] Evans, D.A.; Allison, B.D.; Yang, M.G.; Masse, C.E. *J. Am. Chem. Soc.* **2001**, *123*, 10840-10852
[5] Beruben, D.; Marek, I.; Normant, J.F.; Platzer, N. *J. Org. Chem.* **1995**, *60*, 2488-2501
[6] Still, W.C.; Kahn, M.; Mitra, A. *J. Org. Chem.* **1978**, *43*, 2923-2925

shifts (δ) are reported in parts per million (ppm) downfield from tetramethylsilane and referenced to residual protium in the NMR solvent ($CDCl_3$, δ = 7.26; CD_3CN, δ = 1.93). When solvent mixtures were used, spectra were referenced to an internal standard of tetramethylsilane. Data are reported as follows: chemical shift, multiplicity (s = singlet, d = doublet, t = triplet, q = quartet, qn = quintet, sext = sextet, dd = doublet of doublets, dt = doublet of triplets, ddt = doublet of doublet of triplets, dtd = doublet of triplet of doublets, m = multiplet, br = broad), coupling constant (*J*) in Hertz (Hz), and integration. Based on ^{1}H NMR analysis, unless otherwise indicated the isomeric purity of all olefin-containing compounds was judged to be >95 % (^{1}H and ^{13}C NMR spectra of all compounds are provided in SI.B). MIDA boronates were judged to be stable to long-term storage based on ^{1}H NMR spectra acquired after the indicated time. (^{1}H NMR spectra of **(*E*)-1**, **(*Z*)-1**, and **6** collected both before and after storage on the benchtop under air in a subdued light environment for 6 months are included in SI.B). ^{13}C NMR spectra were recorded at 23 °C on one of the following instruments: Varian Unity 400, Varian Unity 500 Varian Unity Inova 500, Varian Unity Inova 600. Chemical shifts (δ) are reported in ppm downfield from tetramethylsilane and referenced to carbon resonances in the NMR solvent ($CDCl_3$, δ = 77.0; CD_3CN, δ = 118.2). When solvent mixtures were used, spectra were referenced to an internal standard of tetramethylsilane. **Carbons bearing boron substituents were not reported (quadrupolar relaxation)**. ^{11}B NMR were recorded using a General Electric GN300WB instrument and referenced to an external standard of (BF_3•Et_2O). High resolution mass spectra (HRMS) were performed by Furong Sun, Beth Eves, Dr. Haijun Yao, and Dr. Steve Mullen at the University of Illinois School of Chemical Sciences Mass Spectrometry Laboratory. X-ray crystallographic analyses of **6**, (***E***)-**1** and (***Z***)-**1** were carried out by Dr. Danielle Gray at the University of Illinois George L. Clark X-Ray facility.

II. Synthesis of 1-Ethynylboronate ester (6)

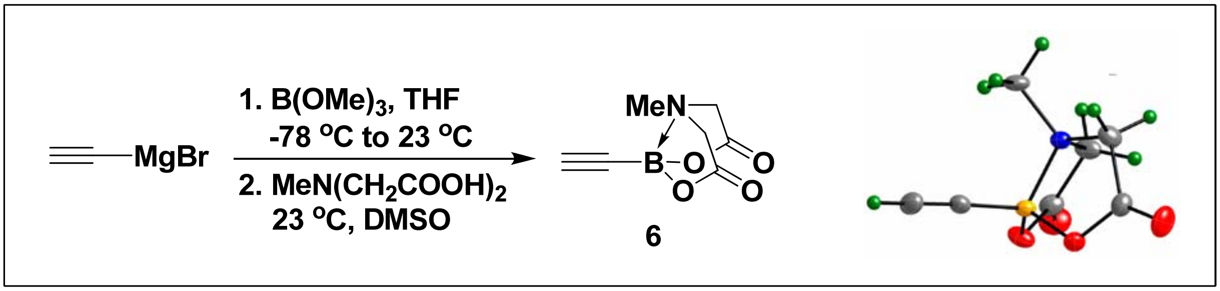

To maximize the yield for this reaction, the MIDA ligand was purified and dried as follows: MIDA was dissolved in a minimum volume of deionized water. Using a mechanical stirrer and a large separatory funnel, the MIDA was precipitated through the dropwise addition of acetone (5X volume relative to water used to dissolve MIDA). The resulting slurry was filtered and the collected white solid was washed with small portions of acetone. This solid was then transferred to a recrystallization dish and placed in a 60 ^{o}C oven for 12 h. The resulting solid was ground in a mortar and then placed into a 120 ^{o}C oven for four hours.

Ethynylboronate ester (6)

To an oven-dried 5000-mL 3-neck round-bottomed flask equipped with a magnetic stir bar, a 500-mL pressure-equalizing addition funnel, and two rubber septa was added THF (750 mL) and trimethyl borate (61 mL, 550 mmol, 1.1 equiv) and the resulting solution was cooled to -78 °C. The addition funnel was charged with the first portion of ethynyl magnesium bromide solution (500 mL, 250 mmol, 0.50 M in THF) which was then added drop-wise over 35 min. The addition funnel was charged with the second portion of ethynyl magnesium bromide solution (500 mL, 250 mmol, 0.50 M in THF) which was then added drop-wise over 30 min. The reaction vessel was removed from the bath and allowed to warm to ambient temperature over the course of 3 h resulting in a thick white slurry. A separate oven-dried 3000-mL 3-neck round-bottomed flask equipped with a magnetic stir bar, a thermometer, 500 mL addition funnel, and a distillation train was charged with MIDA (162 g, 1100 mmol, 2.2 equiv) and DMSO (750 mL). Using a heating mantle and variac, this suspension was brought to an internal temp of 130 °C. To the addition funnel was added 500 mL of hexanes which was then added drop-wise to the MIDA solution (this step was included to azeotropically dry the MIDA solution, head temperature of 60–69 °C) resulting in a homogeneous light-orange solution. The previously prepared suspension of the "ate" complex was added over the course of 1.5 h via cannula transfer under a positive pressure of Ar(g) at a rate such that the internal temperature remained between 120–160 °C. After the addition was completed the reaction vessel was washed with THF (2 x 60 mL) and the washes added via cannula transfer to the reaction vessel containing the MIDA solution. The remaining THF and MeOH were allowed to distill off (~15 min). The reaction vessel was allowed to cool to ambient temperature. The reaction mixture was then transferred to a 6 L separatory funnel. To this was added 1 L of de-ionized water, 1 L of brine, 1.5 L of ethyl acetate, and 1 L of acetone. The organic layer was separated, and the aqueous layer was extracted twice with 500 mL of a 3:2 ethyl acetate : acetone solution and once with 500 mL of ethyl acetate. The combined organic fractions were then washed with 500 mL of brine, and dried with $MgSO_4$. The organic fractions were then concentrated to form a light brown solid. The solid was dissolved in 250 mL of acetone and then was precipitated by the drop-wise addition of 3 L of diethyl ether. The resulting solid was collected via filtration and washed with diethyl ether (2 x 50 mL). The solid was dissolved using 800 mL of acetone. To this solution was added activated charcoal. This was stirred for 30 min, and then filtered through celite

washing with acetone (2 x 50 mL). The resulting solution was concentrated *in vacuo* to afford ethynyl MIDA boronate (66.6 g, 74%).

This compound was stable to long-term storage in a vial on the benchtop under air in a subdued light environment (as judged by the ^{1}H NMR spectrum acquired after 6 months of storage – see SI.B).

TLC (EtOAc)
  R_f = 0.46, visualized with $KMnO_4$.

^{1}H NMR (400 MHz, CD_3CN)
  δ 4.00 (d, J = 17.2 Hz, 2H), 3.87 (d, J = 17.2 Hz, 2H), 3.03 (s, 3H), 2.69 (s, 1H)

^{13}C NMR (100 MHz, CD_3CN)
  δ 168.5, 90.1 (br), 62.2, 48.6

HRMS (ESI)

Calculated for $C_7H_9BNO_4$ $(M+H)^+$:	182.0625
Found:	182.0623

III. Synthesis of (*E*)-1, (*Z*)-1 and (*Z*)-2 (Schemes 1 and 2)

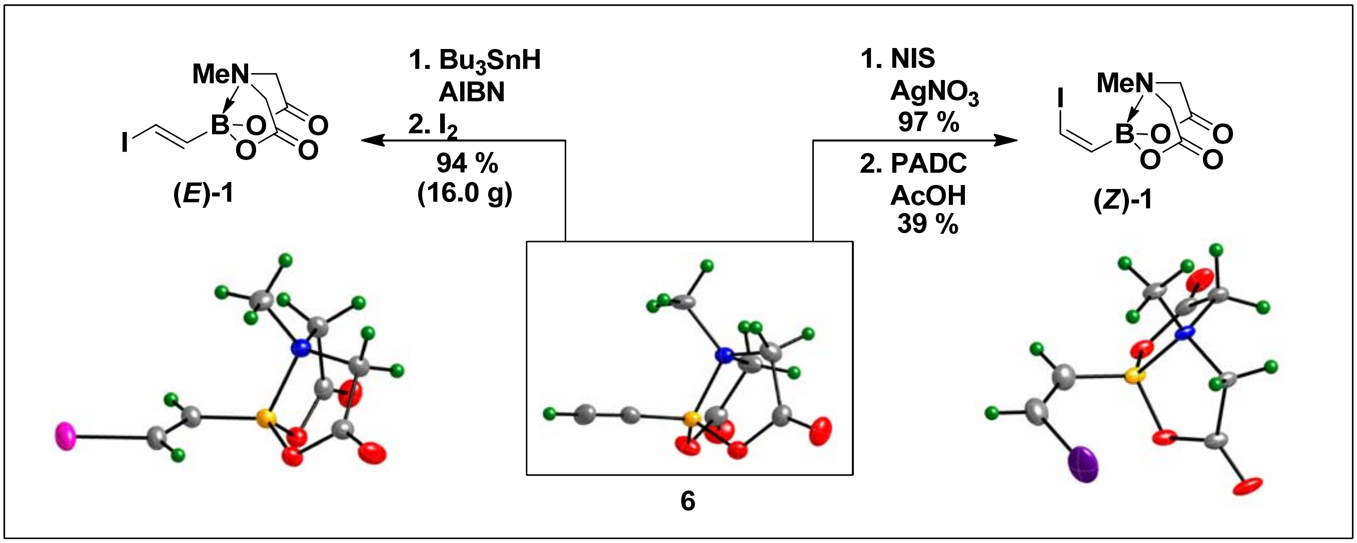

(*E*)-(2-Iodoethenyl)boronate ester (*E*)-1
An oven-dried 1000-ML three-neck round bottom flask with a magnetic stir bar, which was connected to a condenser with Schlenk line, was charged with **6** (10.0 g, 55.2 mmol, 1.0 eq.) and AIBN (0.930 g, 5.52 mmol, 0.10 equiv.). The reaction flask was evacuated and refilled with nitrogen three times. THF (276 mL) and Bu_3SnH (23.0 mL, 82.8 mmol, 1.50 eq.) were added into the reaction flask in order. The colorless suspension was stirred for 18 h at 70 °C using an oil bath. The resulting clear colorless solution was cooled to 23 °C. An oven-dried 250-mL additional funnel was attached to the reaction flask and I_2 (28.0 g, 110.4 mmol, 2.00 eq.) dissolved in THF (165.6 mL) was added into the additional funnel. The reaction mixture was then cooled to –78 °C and the I_2 solution was added dropwise into the reaction mixture over the course of 2 h. The reaction mixture was allowed to stir for 3 h at –78 °C and then slowly

warmed to 23 °C. The resulting mixture was then treated with (sat.) aqueous $Na_2S_2O_3$ (500 mL) and extracted with EtOAc (3 × 750 mL). The combined organic extracts were washed with (sat.) aqueous $Na_2S_2O_3$ (2 × 500 mL) again to remove any remaining iodine resulting in a clear colorless solution. The organic layer was washed with brine (1000 mL) and dried over anhydrous magnesium sulfate, filtered under reduced pressure, and concentrated *in vacuo* to provide a white solid as crude product. The white solid was treated with Et_2O (500 mL) and the resulting colorless crystalline solid was filtered and washed with additional Et_2O (500 mL) to provide **(*E*)-1** (16.02 g, 51.86 mmol, 94 %) as a colorless crystalline solid. This compound was stable to long-term storage in a vial on the benchtop under air in a subdued light environment (as judged by the ^1H NMR spectrum acquired after 6 months of storage – see SI.B).

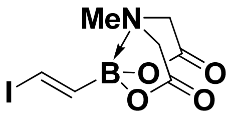

(*E*)-1

TLC (EtOAc)
R_f = 0.50, visualized with $KMnO_4$.

^1H NMR (400 MHz, CD_3CN)
δ 6.85 (d, *J* = 16 Hz, 1H), 6.81 (d, *J* = 16 Hz, 1H), 3.96 (d, *J* = 17.2 Hz, 2H), 3.81 (d, *J* = 17.2 Hz, 2H), 2.79 (s, 3H),

^{13}C NMR (100 MHz, CD_3CN)
δ 168.8, 91.3, 62.5, 47.8

HRMS (ESI)

Calculated for $C_7H_{10}BINO_4$ $(M+H)^+$:	309.9748
Found:	309.9756

Synthesis of (*Z*)-(2-Iodoethenyl)boronate ester (*Z*)-1

2-(Iodoethynyl)boronate ester
In an unoptimized procedure, an oven-dried 200-ML one-neck round bottom flask fitted with a magnetic stir bar was charged with **6** (3.26 g, 18.01 mmol, 1.0 eq.), NIS (5.064 g, 22.51 mmol, 1.25 eq.), $AgNO_3$ (0.459 g, 2.702 mmol, 0.15 equiv.) and acetone (90 mL) under nitrogen. The resulting heterogeneous mixture was stirred for 5 h at 23 °C. The reaction mixture was treated with cold ice-water (250 mL) and extracted with ethyl acetate (3 × 250 mL). The organic layer was washed with brine (200 mL) and dried over anhydrous magnesium sulfate, filtered under reduced pressure, and concentrated *in vacuo* to provide crude product as a yellow oil. To this crude product was added Et_2O (150 mL) and the resulting mixture was concentrated *in vacuo* again to provide a semi-solid. Ethyl acetate (5.0 mL) and Et_2O (150 mL) were added and the resulting white precipitate was filtered and washed with additional Et_2O (50 mL) under reduced pressure to provide 2-(iodoethynyl)boronate ester (5.365 g, 17.48 mmol, 97 %) as a colorless crystalline solid.

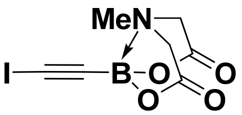

TLC (EtOAc)

R_f = 0.61, visualized with $KMnO_4$.

^{1}H NMR (500 MHz, CD_3CN)

δ 3.97 (d, *J* = 17 Hz, 2H), 3.85 (d, *J* = 17 Hz, 2H), 3.00 (s, 3H)

^{13}C NMR (125 MHz, CD_3CN)

δ 168.5, 62.3, 48.7 (iodinated C not observed due to a very long T1)

HRMS (ESI)

Calculated for $C_7H_8BINO_4$ $(M+H)^+$:	307.9591
Found:	307.9586

(*Z*)-(2-Iodoethenyl)boronate ester (*Z*)-1

An oven-dried 250-ML one-neck round bottom flask with a magnetic stir bar was charged with 2-(iodoethynyl)boronate ester (5.364 g, 17.47 mmol, 1.0 eq.) and potassium azodicarboxylate (PADC) (7.794 g, 40.18 mmol, 2.3 eq.). The reaction flask was evacuated and refilled with nitrogen three-times. THF (150 mL) and freshly distilled pyridine (6.356 g, 80.36 mmol, 4.6 eq.) were added into the reaction mixture in order. To the resulting stirred heterogeneous mixture, a solution of acetic acid (4.825 g, 80.36 mmol, 4.6 eq.) in THF (3.0 mL) was added over the course of 3 h at 23 ^oC via syringe pump. After successful addition of acetic acid, the reaction mixture was allowed to stir for an additional 2 h at 23 ^oC. The reaction mixture including yellow solid was diluted with ethyl acetate (200 mL) and filtered through a short pad Celite®. The colorless filtrate was concentrated *in vacuo* to provide a white solid as crude product. The crude product was separated by MPLC (ethyl acetate:acetone 30:1 (*v/v*), SiO_2) to give **(Z)-1** (0.431 g, 1.396 mmol, 8.0 %) and unreacted 2-(iodoethynyl)boronate ester (4.470 g, 14.57 mmol, recovered yield: 83 %). This process was repeated four times with recovered starting material from each previous reaction. The combined products were further purified via recrystallizations from EtOAc:Et_2O 1:20 (*v/v*) to yield stereochemically pure **(Z)-1** (2.08 g, 6.734 mmol, 39%) as colorless crystalline solid. This compound was stable to long-term storage in a vial on the benchtop under air in a subdued light environment (as judged by the ^{1}H NMR spectrum acquired after 6 months of storage – see SI.B).

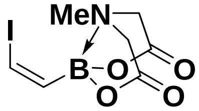

(*Z*)-1

TLC (EtOAc)

R_f = 0.42, visualized with $KMnO_4$.

^{1}H NMR (500 MHz, CD_3CN)

δ 7.12 (br d, *J* = 9 Hz, 1H), 6.84 (d, *J* = 10 Hz, 1H), 4.03 (d, *J* = 17 Hz, 2H), 3.88 (d, *J* = 17.5 Hz, 2H), 2.87 (s, 3H)

^{13}C NMR (125 MHz, CD_3CN)
δ 169.0, 94.7, 63.8, 48.2

HRMS (ESI)
Calculated for $C_7H_{10}BINO_4$ $(M+H)^+$: 309.9748
Found: 309.9751

Synthesis of (*Z*)-1-(Tributylstannyl)-2-triethylgermanium (*Z*)-2

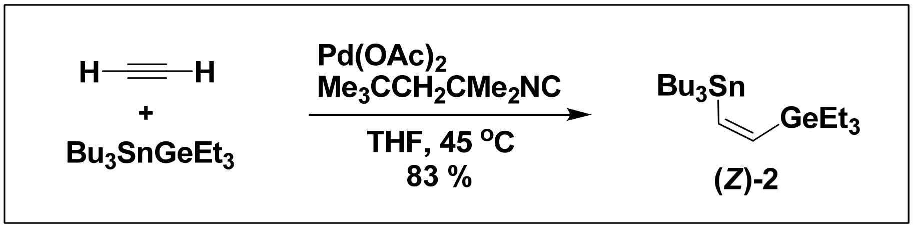

Triethylgermyl tri-*n*-butylstannane[7]
To a stirred solution of freshly distilled di-*iso*-propylamine (1.75 mL, 12.5 mmol) in THF (12.0 mL) at–78 °C was added *n*-butyllithium (4.20 mL, 10.5 mmol, 2.5 M solution) dropwise via syringe over 15 min. The mixture was stirred for 45 min at –78 °C and then slowly warmed to 23 °C. Tributyltin hydride (2.18 mL, 8.10 mmol) was added over the course of 15 min. and the reaction mixture was allowed to stir at 23 °C for 1h 30 min. Triethylgermyl chloride (1.96 mL, 11.8 mmol) was then added and the resulting cloudy solution was stirred at 23 °C for 16 h. The solvent was evaporated *in vacuo* and the resulting crude mixture was diluted with Et_2O and filtered under reduced pressure. The filtrate was concentrated *in vacuo* and the crude product was purified by flash chromatography on silica gel (hexanes) to give triethylgermyl tri-*n*-butylstannane as a clear, colorless oil (3.64 g, 8.09 mmol, 99 %). Spectral data for this compound were consistent with those reported by Nakano et al.[8]

(*Z*)-1-(Tributylstannyl)-2-(triethylgermanyl)-ethylene (*Z*)-2
An oven-dried 50-ML one-neck round bottom flask equipped with a magnetic stir bar was charged with triethylgermyl tri-*n*-butylstannane (3.374 g, 7.500 mmol, 1.0 eq.), $Pd(OAc)_2$ (0.152 g, 0.675 mmol, 9.0 mol % Pd), 1,1,3,3-tetramethylbutyl isocyanide (0.395 mL, 2.25 mmol, 30 mol %) and THF (15.0 mL) in an argon-filled glove box. The reaction flask was capped with a rubber septum and placed on a stir plate in a fume hood. An acetylene gas line (including a gas-bubbler) was connected to the reaction flask and acetylene gas was slowly introduced with gentle bubbling. The yellow reaction mixture was stirred at 45 °C for 60 h under an acetylene atmosphere. The resulting reddish mixture was concentrated *in vacuo* and the crude product was purified by flash chromatography on Florisil® (hexanes) to give the title compound **(*Z*)-2** as a clear, colorless oil (2.955 g, 6.210 mmol, 83 %).

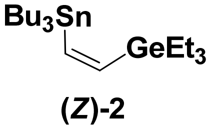

(*Z*)-2

[7] For a related synthesis of triethylsilyl-tri-*n*-butylstannane, see: *J. Am. Chem. Soc.* **2003**, *125*, 15402-15410.
[8] Nakano, T.; Ono, K.; Migita, T. *Chem. Lett.* **1996**, *25*, 697-698

TLC (Pet. ether)

R_f = 0.97, visualized by UV lamp (λ = 254 nm) or with $KMnO_4$.

^{1}H NMR (500 MHz, $CDCl_3$)[9]

δ 7.12 (d, *J* = 17.5 Hz, 1H), 7.06 (d, *J* = 17.5 Hz, 1H), 1.49 (m, 6H), 1.29 (app sext, *J* = 7.25 Hz, 6H), 1.02 (t, *J* = 8 Hz, 9H), 0.88 (m, 15H), 0.79 (q, *J* = 7.8 Hz, 6H)

^{13}C NMR (125 MHz, $CDCl_3$)

δ 151.8, 150.2, 29.1, 27.4, 13.7, 10.7, 9.1, 4.9

HRMS (CI)

Calculated for $C_{20}H_{44}GeSn$:	478.1677
Found:	478.1690

[9] The coupling constants for the olefinic protons in (***E***)-**2** are 22.4 and 22.4 Hz.

IV. Syntheses of 3 (Scheme 3)

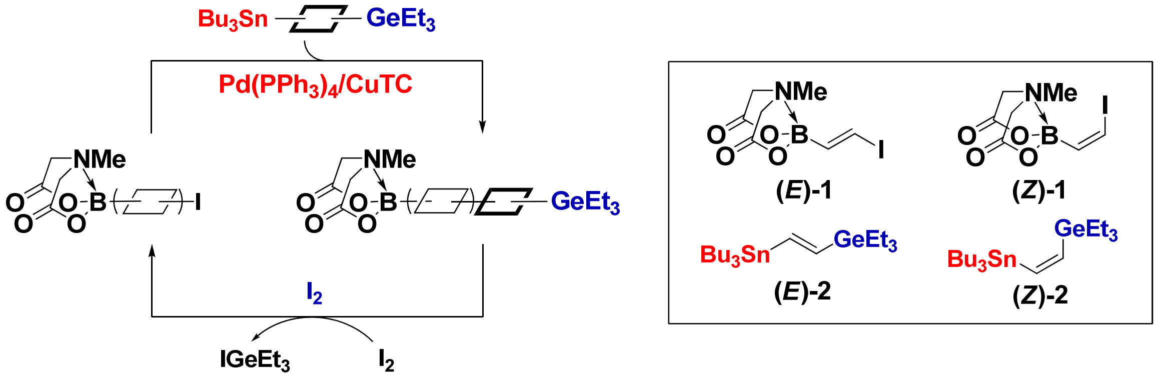

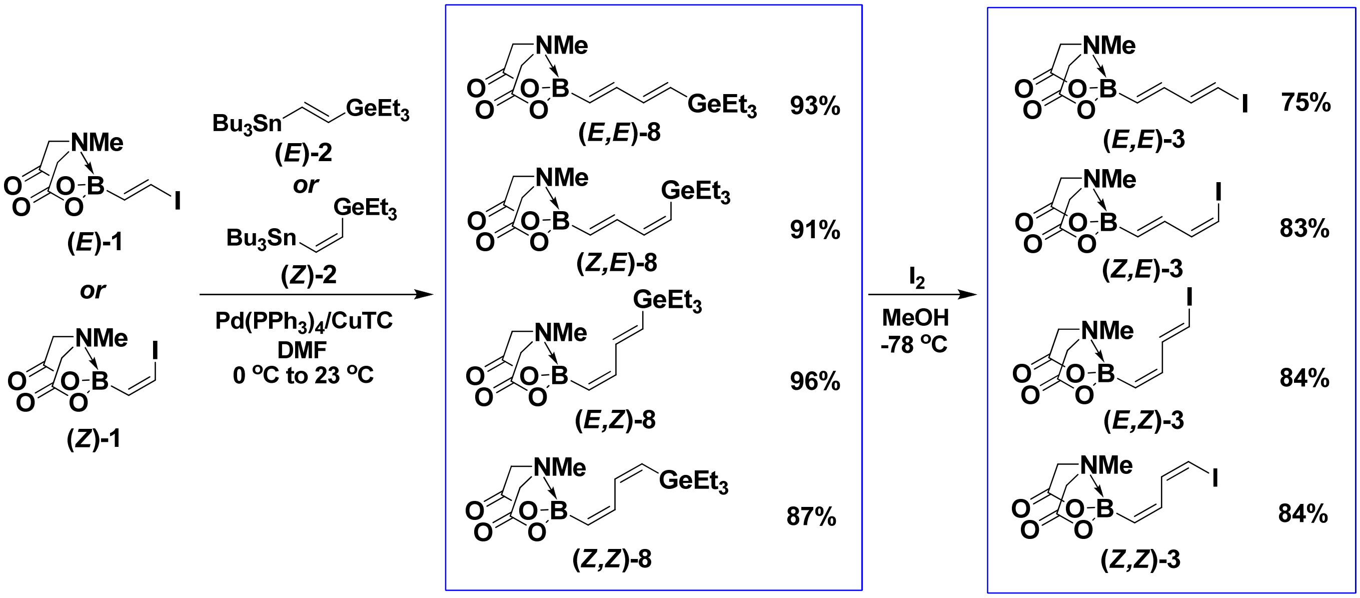

(*E*,*E*)-8

A 65-ML one-neck round bottom flask with a magnetic stir bar was charged with **(*E*)-1** (1.00 g, 3.236 mmol, 1.0 eq.), $Pd(PPh_3)_4$ (0.187 g, 0.162 mmol, 0.05 equiv.) and copper(I) thiophene-2-carboxylate (Cu(TC)) (0.926 g, 4.854 mmol, 1.50 eq.) and capped with a rubber septum. The reaction flask was cooled to 0 °C and DMF (11.2 mL) was added. **(*E*)-2** (1.771 g, 3.721 mmol, 1.15 eq.) dissolved in DMF (5.0 mL) was added via syringe at 0 °C. The resulting heterogeneous reddish mixture was stirred at 0 °C for 2 h and then slowly warmed to 23 °C and stirred for an additional 1 h at 23 °C. The reaction mixture was poured into (sat.) aqueous NaCl (150 mL) and extracted with ethyl acetate (3 × 150 mL). The combined organic phases were dried over anhydrous magnesium sulfate, and concentrated *in vacuo* to provide an orange oil. The residue of DMF was removed via azeotrope with toluene (3 × 30 mL) at 30 °C *in vacuo*. The crude product was purified by flash chromatography on silica gel (EtOAc:petroleum ether 1:1 → EtOAc → EtOAc:MeCN 9:1) to give the title compound **(*E*,*E*)-8** as a white solid (1.111 g, 3.020 mmol, 93 %).

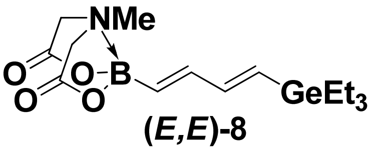

(*E*,*E*)-8

TLC (EtOAc)

R_f = 0.64, visualized by UV lamp (λ = 254 nm) or with $KMnO_4$.

^1H NMR (500 MHz, CD_3CN)

δ 6.59 (dd, *J* = 18, 10 Hz, 1H), 6.54 (dd, *J* = 17, 10 Hz, 1H), 6.11 (d, *J* = 17.5 Hz, 1H), 5.61 (d, *J* = 17.5 Hz, 1H), 3.94 (d, *J* = 17 Hz, 2H), 3.78 (d, *J* = 17 Hz, 2H), 2.76 (s, 3H), 1.02 (t, *J* = 7.75 Hz, 9H), 0.81 (q, *J* = 7.5 Hz, 6H)

^{13}C NMR (125 MHz, CD_3CN)

δ 169.2, 147.0, 146.0, 134.4, 62.3, 47.6, 9.1, 4.8

HRMS (ESI)

Calculated for $C_{15}H_{27}BGeNO_4$ $(M+H)^+$:	370.1245
Found:	370.1254

(*E*,*Z*)-8

A 25-ML Wheaton vial equipped with a magnetic stir bar was charged with **(*Z*)-1** (309 mg, 1.00 mmol, 1.0 eq.), $Pd(PPh_3)_4$ (58.2 mg, 0.05 mmol, 0.05 eq.) and copper(I) thiophene-2-carboxylate (Cu(TC)) (286 mg, 1.50 mmol, 1.50 eq.). The vial was then sealed with a PTFE-lined plastic cap. The reaction mixture was cooled to 0 °C and DMF (3.0 mL) was added. **(*E*)-2** (547 mg, 1.15 mmol, 1.15 eq.) dissolved in DMF (2.0 mL) was then added *via* syringe. The resulting heterogeneous reddish mixture was stirred at 0 °C - 10 °C for 3 h 30 min. The deep reddish reaction mixture was treated with (sat.) aqueous NaCl (100 mL) and extracted with ethyl acetate (3 × 125 mL). The combined organic phases were dried over anhydrous magnesium sulfate, and concentrated *in vacuo* to provide an orange oil. The residue of DMF was removed via azeotrope with toluene (3 × 35 mL) at 30 °C *in vacuo*. The crude orange product was purified by flash chromatography on Florisil® (EtOAc:petroleum ether 1:1 → EtOAc → EtOAc:MeCN 9:1) to give the title compound **(*E*,*Z*)-8** as a colorless crystalline solid (353 mg, 0.959 mmol, 96 %).

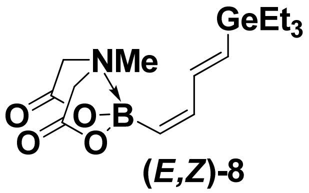

(*E*,*Z*)-8

TLC (EtOAc)

R_f = 0.64, visualized by UV lamp (λ = 254 nm) or with $KMnO_4$.

^1H NMR (500 MHz, CD_3CN)

δ 7.02 (ddd, *J* = 18, 11, 1 Hz, 1H), 6.69 (app br t, *J* = 12.3 Hz, 1H), 6.10 (d, *J* = 18 Hz, 1H), 6.32 (d, *J* = 14 Hz, 1H), 3.96 (d, *J* = 17 Hz, 2H), 3.78 (d, *J* = 17 Hz, 2H), 2.78 (s, 3H), 1.01 (t, *J* = 7.8 Hz, 9H), 0.80 (q, *J* = 8 Hz, 6H)

^{13}C NMR (125 MHz, CD_3CN)

δ 169.0, 147.1, 143.7, 136.4, 62.4, 47.5, 9.2, 4.8

HRMS (ESI)

Calculated for $C_{15}H_{27}BGeNO_4$ $(M+H)^+$: 370.1245
Found: 370.1251

(*Z*,*E*)-8

A 25-ML Wheaton vial equipped with a magnetic stir bar was charged with **(*E*)-1** (232 mg, 0.750 mmol, 1.0 eq.), $Pd(PPh_3)_4$ (43.3 mg, 0.0375 mmol, 0.05 equiv.) and copper (I) thiophene-2-carboxylate (Cu(TC)) (214 mg, 1.125 mmol, 1.50 eq.). The vial was then sealed with a PTFE-lined plastic cap. The reaction mixture was cooled to 0 °C and DMF (2.0 mL) was added, and then **(*Z*)-2** (410 mg, 0.862 mmol, 1.15 eq.) dissolved in DMF (1.75 mL) was added *via* syringe. The resulting heterogeneous reddish mixture was stirred at 0 °C - 10 °C for 3 h 30 min. The deep reddish reaction mixture was treated with (sat.) aqueous NaCl (100 mL) and extracted with ethyl acetate (3 × 125 mL). The combined organic phases were dried over anhydrous magnesium sulfate, and concentrated *in vacuo* to provide an orange oil. The residue of DMF was removed via azeotrope with toluene (3 × 35 mL) at 30 °C *in vacuo*. The crude orange product was purified by flash chromatography on Florisil® (EtOAc:petroleum ether 1:1 → EtOAc → EtOAc:MeCN 9:1) to give the title compound **(*Z*,*E*)-8** as a colorless crystalline solid (252 mg, 0.685 mmol, 91 %).

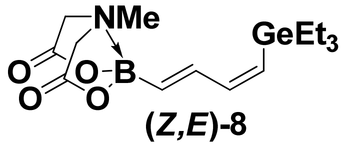

(*Z*,*E*)-8

TLC (EtOAc)

R_f = 0.64, visualized by UV lamp (λ = 254 nm) or with $KMnO_4$.

^{1}H NMR (500 MHz, CD_3CN)

δ 6.99 (dd, *J* = 12.5, 11 Hz, 1H), 6.65 (dd, *J* = 17.3, 11 Hz, 1H), 5.89 (d, *J* = 12.5 Hz, 1H), 5.67 (d, *J* = 17.5 Hz, 1H), 3.95 (d, *J* = 16.5 Hz, 2H), 3.74 (d, *J* = 17 Hz, 2H), 2.74 (s, 3H), 1.02 (t, *J* = 7.75 Hz, 9H), 0.86 (q, *J* = 7.75 Hz, 6H)

^{13}C NMR (125 MHz, CD_3CN)

δ 169.2, 147.4, 144.5, 134.2, 62.1, 47.5, 9.3, 6.4

HRMS (ESI)

Calculated for $C_{15}H_{27}BGeNO_4$ $(M+H)^+$: 370.1245
Found: 370.1244

(*Z*,*Z*)-8

A 25-ML Wheaton vial equipped with a magnetic stir bar was charged with **(*Z*)-1** (309 mg, 1.00 mmol, 1.0 eq.), $Pd(PPh_3)_4$ (58.2 mg, 0.05 mmol, 0.05 equiv.) and copper(I) thiophene-2-carboxylate (Cu(TC)) (286 mg, 1.50 mmol, 1.50 eq.). The vial was then sealed with a PTFE-lined plastic cap. The reaction mixture was cooled to 0 °C and DMF (3.0 mL) was added, and then **(*Z*)-2** (547 mg, 1.15 mmol, 1.15 eq.) dissolved in DMF (2.0 mL) was added *via* syringe. The resulting heterogeneous reddish mixture was stirred at 0 °C - 10 °C for 5 h. The deep reddish reaction mixture was treated with (sat.) aqueous NaCl (100 mL) and extracted with ethyl acetate (3 × 125 mL). The combined organic phases were dried over anhydrous magnesium sulfate, and concentrated *in vacuo* to provide an orange oil. The residue of DMF

was removed via azeotrope with toluene (3 × 35 mL) at 30 °C *in vacuo*. The crude orange product was purified by flash chromatography on Florisil® (EtOAc:petroleum ether 1:1 → EtOAc → EtOAc:MeCN 9:1) to give the title compound **(*Z,Z*)-8** as a colorless crystalline solid (320 mg, 0.869 mmol, 87 %).

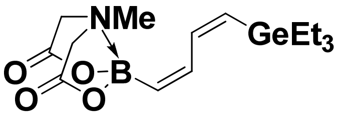

(*Z,Z*)-8

TLC (EtOAc)
R_f = 0.64, visualized by UV lamp (λ = 254 nm) or with $KMnO_4$.

^{1}H NMR (500 MHz, CD_3CN)
δ 7.34 (app td, *J* = 12.25, 1 Hz, 1H), 6.78 (app t, *J* = 12.25 Hz, 1H), 5.95 (d, *J* = 13 Hz, 1H), 5.46 (d, *J* = 14 Hz, 1H), 3.96 (d, *J* = 16.5 Hz, 2H), 3.79 (d, *J* = 17 Hz, 2H), 2.80 (s, 3H), 1.03 (t, *J* = 7.75 Hz, 9H), 0.87 (q, *J* = 8 Hz, 6H)

^{13}C NMR (125 MHz, CD_3CN)
δ 169.1, 145.1, 144.3, 135.5, 62.5, 47.5, 9.3, 6.4

HRMS (ESI)

Calculated for $C_{15}H_{27}BGeNO_4$ $(M+H)^+$:	370.1245
Found:	370.1248

Efficient and Stereospecific Syntheses of All Possible Stereoisomer of 3 from 8 via Iododegermylation

(*E,E*)-3

A 200-ML one-neck round bottom flask with a magnetic stir bar was charged with **(*E,E*)-8** (1.10 g, 3.00 mmol, 1.0 eq.) and capped with a rubber septum. The reaction flask was evacuated and refilled with nitrogen three-times. MeOH (70 mL) was added into the flask via syringe and the resulting mixture was stirred for 5 min. at 23 °C providing a clear solution. This solution was then cooled to –78 °C and I_2 (3.81 g, 15.0 mmol, 5.0 eq.) dissolved in MeOH (30 mL) was added dropwise into the reaction mixture for 45 min. The reddish reaction mixture was stirred for 3 h at –78 °C under nitrogen. (sat.) Aqueous $Na_2S_2O_3$ (12.0 mL) was added to the reaction mixture at –78 °C and the resulting yellow solution was warmed to 23 °C. The mixture was treated with additional (sat.) aqueous $Na_2S_2O_3$ (200 mL) and extracted with EtOAc (3 × 250 mL). The combined organic extracts were washed with (sat.) aqueous $Na_2S_2O_3$ (200 mL) again. The organic layer was washed with brine (200 mL) and dried over anhydrous magnesium sulfate, and concentrated *in vacuo* to provide a white solid as crude product. The crude product was treated with Et_2O (25 mL) and the resulting white precipitate was filtered and washed with additional Et_2O (70 mL) to provide **(*E,E*)-3** (0.7553 g, 2.255 mmol, 75 %) as a colorless crystalline solid.

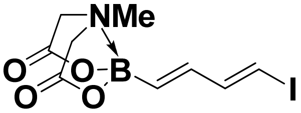

(*E,E*)-3

TLC (EtOAc)
R_f = 0.54, visualized by UV lamp (λ = 254 nm) or with $KMnO_4$.

^{1}H NMR (500 MHz, CD_3CN)

δ 7.16 (ddd, *J* = 14.25, 10.5, 0.75 Hz, 1H), 6.58 (dd, *J* = 14, 1 Hz, 1H), 6.50 (dd, *J* = 17.5, 10.5 Hz, 1H), 5.67 (dd, *J* = 17.5, 0.5 Hz, 1H), 3.95 (d, *J* = 16.5 Hz, 2H), 3.78 (d, *J* = 17 Hz, 2H), 2.76 (s, 3H)

^{13}C NMR (125 MHz, CD_3CN)

δ 169.2, 148.2, 142.8, 82.5, 62.3, 47.6

HRMS (ESI)

Calculated for $C_9H_{12}BINO_4$ $(M+H)^+$: 335.9904
Found: 335.9914

(*E,Z*)-3

A 65-ML one-neck round bottom flask with a magnetic stir bar was charged with **(*E,Z*)-8** (255 mg, 0.693 mmol, 1.0 eq.) and capped with a rubber septum. The reaction flask was evacuated and refilled with nitrogen three-times. MeOH (10 mL) was added into the flask via syringe and the resulting mixture was stirred for 5 min. at 23 °C providing a clear solution. This solution was then cooled to –78 °C and I_2 (879 mg, 3.465 mmol, 5.0 eq.) dissolved in MeOH (13.1 mL) was added dropwise into the reaction mixture for 30 min. The reddish reaction mixture was stirred for 3 h 30 min. at –78 °C under nitrogen. (sat.) Aqueous $Na_2S_2O_3$ (10 mL) was then added at –78 °C and the resulting yellow solution was warmed to 23 °C. This solution was treated with additional (sat.) aqueous $Na_2S_2O_3$ (100 mL) and extracted with EtOAc (3 × 125 mL). The combined organic extracts were washed with (sat.) aqueous $Na_2S_2O_3$ (100 mL) again. The organic layer was dried over anhydrous magnesium sulfate, and concentrated *in vacuo* to provide a white solid as crude product. The crude product was treated with Et_2O (1.5 mL) and the resulting white precipitate was filtered and washed with additional Et_2O (10 mL) to provide **(*E,Z*)-3** (194 mg, 0.580 mmol, 84 %) as a colorless crystalline solid.

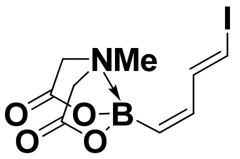

(*E,Z*)-3

TLC (EtOAc)

R_f = 0.54, visualized by UV lamp (λ = 254 nm) or with $KMnO_4$.

^{1}H NMR (500 MHz, CD_3CN)

δ 7.59 (ddd, *J* = 14, 11.5, 1.5 Hz, 1H), 6.61 (app br t, *J* = 12.5 Hz, 1H), 6.56 (dt, *J* = 14. 0.75 Hz, 1H), 5.38 (ddd, *J* = 13.75, 0.75, 0.75 Hz, 1H), 3.97 (d, *J* = 16.5 Hz, 2H), 3.81 (d, *J* = 16.5 Hz, 2H), 2.80 (s, 3H)

^{13}C NMR (125 MHz, CD_3CN)

δ 169.0, 145.2, 143.5, 84.2, 62.4, 47.5

HRMS (ESI)

Calculated for $C_9H_{12}BINO_4$ $(M+H)^+$: 335.9904
Found: 335.9901

(*Z,E*)-3

A 65-ML one-neck round bottom flask with a magnetic stir bar was charged with **(*Z,E*)-8** (207 mg, 0.563 mmol, 1.0 eq.) and capped with rubber septum. The reaction flask was evacuated and refilled with nitrogen three-times. MeOH (10 mL) was added into the flask via syringe and the resulting mixture was stirred for 5 min. at 23 °C providing a clear solution. This solution was then cooled to –78 °C and I_2 (715 mg, 2.815 mmol, 5.0 eq.) dissolved in MeOH (8.80 mL) was added dropwise over the course of 25 min. The reddish reaction mixture was stirred for 3 h at –78 °C under nitrogen. (sat.) aqueous $Na_2S_2O_3$ (10 mL) was then added at –78 °C and the resulting yellow solution was warmed to ambient temperature. ThThis solution was treated with additional (sat.) aqueous $Na_2S_2O_3$ (100 mL) and then extracted with EtOAc (3 × 125 mL). The combined organic extracts were washed with (sat.) aqueous $Na_2S_2O_3$ (100 mL) again. The organic layer was dried over anhydrous magnesium sulfate, and concentrated *in vacuo* to provide a white solid as crude product. The crude product was treated with Et_2O (1.5 mL) and the resulting white precipitate was filtered and washed with additional Et_2O (10 mL to provide **(*Z,E*)-3** (157 mg, 0.469 mmol, 83 %) as a colorless crystalline solid.

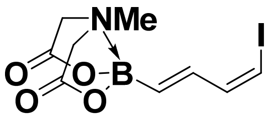

(*Z,E*)-3

TLC (EtOAc)

R_f = 0.54, visualized by UV lamp (λ = 254 nm) or with $KMnO_4$.

^{1}H NMR (500 MHz, CD_3CN)

δ 6.84 (ddd, *J* = 10, 8, 1 Hz, 1H), 6.66 (dd, *J* = 17.5, 10 Hz, 1H), 6.43 (d, *J* = 7.5 Hz, 1H), 5.99 (d, *J* = 17.5 Hz, 1H), 3.98 (d, *J* = 17 Hz, 2H), 3.82 (d, *J* = 17 Hz, 2H), 2.78 (s, 3H)

^{13}C NMR (125 MHz, CD_3CN)

δ 169.2, 142.7, 140.9, 84.3, 62.4, 47.7

HRMS (ESI)

Calculated for $C_9H_{12}BINO_4$ $(M+H)^+$: 335.9904

Found: 335.9903

(*Z,Z*)-3

A 65-ML one-neck round bottom flask with a magnetic stir bar was charged with **(*Z,Z*)-8** (242.6 mg, 0.659 mmol, 1.0 eq.) and capped with rubber septum. The reaction flask was evacuated and refilled with nitrogen three-times. MeOH (10 mL) was added into the flask via syringe and the resulting mixture was stirred for 5 min. at 23 °C providing a clear solution. This solution was then cooled to –78 °C and I_2 (836 mg, 3.295 mmol, 5.0 eq.) dissolved in MeOH (12.0 mL) was added dropwise over the course of 30 min. The resulting mixture was stirred for 3 h 30 min. at –78 °C under nitrogen. (sat.) Aqueous $Na_2S_2O_3$ (10 mL) was added at –78 °C and the resulting yellow solution was warmed to 23 °C. The mixture was treated with additional (sat.) aqueous $Na_2S_2O_3$ (100 mL) and extracted with EtOAc (3 × 125 mL). The combined organic extracts were washed with (sat.) aqueous $Na_2S_2O_3$ (100 mL) again. The organic layer was dried over anhydrous magnesium sulfate, and concentrated *in vacuo* to provide a white solid as crude product. The crude product was treated with Et_2O (1.0 mL) and the resulting white precipitate was filtered and

washed with additional Et_2O (10 mL) to provide **(*Z,Z*)-3** (184.3 mg, 0.550 mmol, 84 %) as a colorless crystalline solid.

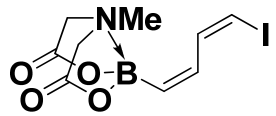

(*Z,Z*)-3

TLC (EtOAc)

R_f = 0.54, visualized by UV lamp (λ = 254 nm) or with $KMnO_4$.

^{1}H NMR (400 MHz, CD_3CN)

δ 7.23 (ddd, *J* = 10.4, 7.6, 1.2 Hz, 1H), 6.74 (app br t, *J* = 12 Hz, 1H), 6.54 (dt, *J* = 7.6, 1.2 Hz, 1H), 5.72 (dt, *J* = 14, 1.2 Hz, 1H), 3.97 (d *J* = 17.2 Hz, 2H), 3.81 (d, *J* = 18 Hz, 2H), 2.80 (s, 3H)

^{13}C NMR (100 MHz, CD_3CN)

δ 168.9, 143.4, 137.5, 87.5, 62.4, 47.5

HRMS (ESI)

Calculated for $C_9H_{12}BINO_4$ $(M+H)^+$:	335.9904
Found:	335.9912

V. Synthesis of 4 (Scheme 3)

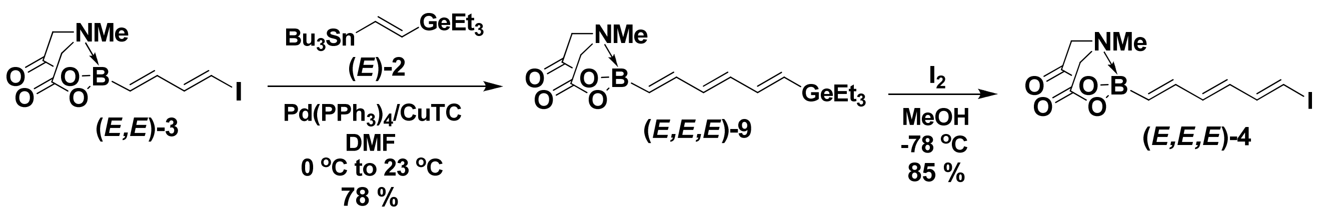

(*E,E,E*)-9

A 25-ML Wheaton vial equipped with a magnetic stir bar was charged with $Pd(PPh_3)_4$ (130 mg, 0.113 mmol, 0.05 equiv.) and copper(I) thiophene-2-carboxylate (Cu(TC)) (644 mg, 3.375 mmol, 1.50 eq.) and was then sealed with a PTFE-lined plastic cap. The vial was cooled to 0 °C and DMF (3.25 mL) was added. A solution of **(*E*)-2** (1.233 g, 2.59 mmol, 1.15 eq.) in DMF (2.0 mL) was added and then a solution of **(*E,E*)-3** (755 mg, 2.25 mmol, 1.0 eq.) in DMF (6.0 mL) was added dropwise via syringe at 0 °C over the course of 15 min. The resulting heterogeneous reddish mixture was stirred at 0 °C - 10 °C for 3 h 30 min. The reaction mixture was poured into (sat.) aqueous NaCl (100 mL) and extracted with ethyl acetate (3 × 100 mL). The combined organic phases were dried over anhydrous magnesium sulfate, filtered under reduced pressure, and concentrated *in vacuo* to provide a reddish oil. The residue of DMF was removed under high vacuum at 23 °C after azeotrope with toluene at 30 °C *in vacuo*. The crude product was purified by flash chromatography on Florisil® (EtOAc:petroleum ether 1:1 → EtOAc → EtOAc:MeCN 9:1) to give the title compound **(*E,E,E*)-9** as a white solid (693 mg, 1.760 mmol, 78 %).

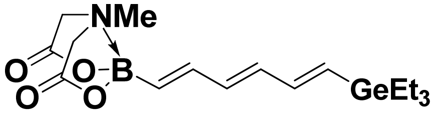

(*E*,*E*,*E*)-9

TLC (EtOAc)

R_f = 0.58, visualized by UV lamp (λ = 254 nm) *or* with $KMnO_4$

^{1}H NMR (500 MHz, CD_3CN)

δ 6.61-6.55 (m, 2H), 6.31-6.29 (m, 2H), 6.10 (d, *J* = 18.5 Hz, 1H), 5.67 (d, *J* = 17 Hz, 1H), 3.93 (d, *J* = 17 Hz, 2H), 3.78 (d, *J* = 17 Hz, 2H), 2.75 (s, 3H), 1.01 (t, *J* = 7.5 Hz, 9H), 0.80 (q, *J* = 8 Hz, 6H)

^{13}C NMR (125 MHz, CD_3CN)

δ 169.3, 144.9, 143.7, 136.9, 135.0, 134.8, 62.3, 47.6, 9.1, 4.8

HRMS (ESI)

Calculated for $C_{17}H_{29}BGeNO_4$ $(M+H)^+$:	396.1401
Found:	396.1401

(*E*,*E*,*E*)-4

A 65-ML one-neck round bottom flask with a magnetic stir bar was charged with **(*E*,*E*,*E*)-9** (346 mg, 0.878 mmol, 1.0 eq.) and capped with a rubber septum. The reaction flask was evacuated and refilled with nitrogen three-times. MeOH (20 mL) was added into the flask via syringe and the reaction mixture was stirred for 5 min. at 23 °C providing a clear solution. This solution was then cooled to –78 °C and I_2 (669 mg, 2.634 mmol, 3.0 eq.) dissolved in MeOH (11.4 mL) was added dropwise over the course of 25 min. The reddish reaction mixture was stirred for 3 h at –78 °C. Sat. aqueous $Na_2S_2O_3$ (10 mL) was added to the reaction mixture at –78 °C, and the resulting yellow solution was warmed to 23 °C. The mixture was treated with additional (sat.) aqueous $Na_2S_2O_3$ (100 mL) and extracted with EtOAc (3 × 125 mL). The combined organic extracts were washed with (sat.) aqueous $Na_2S_2O_3$ (100 mL) again. The organic layer was dried over anhydrous magnesium sulfate, filtered under reduced pressure, and concentrated *in vacuo* to provide a white solid as crude product. The crude product was treated with Et_2O (1.5 mL) and the resulting white precipitate was filtered and washed with additional Et_2O (10 mL) to provide **(*E*,*E*,*E*)-4** (269 mg, 0.746 mmol, 85 %) as a colorless crystalline solid.

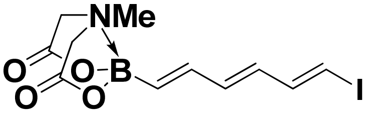

(*E*,*E*,*E*)-4

TLC (EtOAc)

R_f = 0.47, visualized by UV lamp (λ = 254 nm) *or* with $KMnO_4$

^{1}H NMR (400 MHz, CD_3CN)

δ 7.14 (dd, *J* = 14.4, 10.4 Hz, 1H), 6.55 (dd, *J* = 17.6, 10 Hz, 1H), 6.52 (d, *J* = 14.4 Hz, 1H), 6.35 (dd, *J* = 18.75, 13 Hz, 1H), 6.25 (dd, *J* = 18.75, 13.5 Hz, 1H), 5.78 (d, *J* = 17.2 Hz, 1H), 3.99 (d, *J* = 17.2 Hz, 2H), 3.82 (d, *J* = 16.8 Hz, 2H), 2.77 (s, 3H)

^{13}C NMR (125 MHz, CD_3CN)

δ 169.2, 146.1, 142.8, 136.0, 133.7, 81.1, 62.3, 47.6

HRMS (ESI)

Calculated for $C_{11}H_{14}BINO_4$ $(M+H)^+$:	362.0061
Found:	362.0046

IV. Suzuki-Miyaura Cross-Couplings

Synthesis of (*Z*)-1-pentenylboronic acid (*Z*)-10

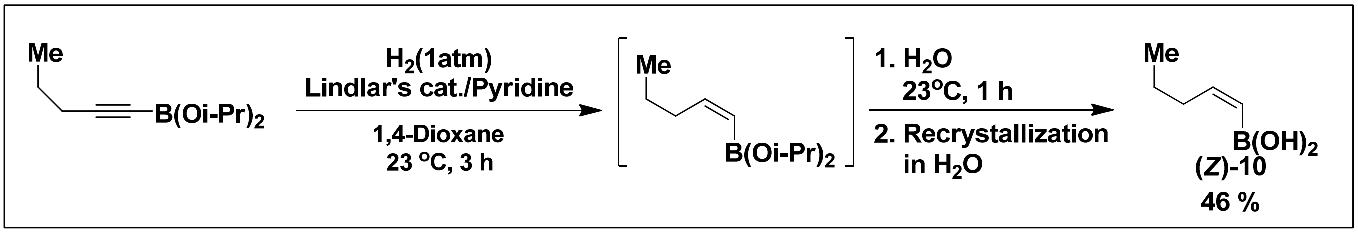

(1-pentyn-1-yl)-diisopropyloxyborane was prepared as previously described (*Angew. Chem. Int. Ed.*, **2002**, *41(17)*, 3272-3276). An oven-dried 100-ML one-neck round bottom flask with a magnetic stir bar was charged with (1-pentyn-1-yl)-diisopropyloxyborane (500 mg, 2.55 mmol, 1.0 eq.), Lindlar's catalyst (25.0 mg, 5 wt. %), 1,4-dioxane (3.0 mL) and pyridine (5.0 µl, 0.0638 mmol, 2.5 mol %). The reaction flask was sealed with a rubber septum and H_2 gas was then introduced using a balloon. The heterogeneous mixture was stirred at 23 °C for 3 h under hydrogen. After being stirred for 3 h, the reaction mixture was filtered under reduced pressure to remove Lindlar's catalyst. The filtrate was concentrated *in vacuo* to provide (*Z*)-(1-pentenyl)-diisopropyloxyborane as a colorless oil. This crude product was immediately converted to (Z)-1-pentenylboronic acid via hydrolysis in H_2O as follows: (*Z*)-(1-pentenyl)-diisopropyloxyborane in H_2O (10 mL) was stirred for 1 h at 23 °C. After being stirred for 1 h, the reaction mixture was extracted with Et_2O (3 × 25 mL) and the combined organic layer was dried over anhydrous magnesium sulfate, filtered under reduced pressure, and concentrated *in vacuo* to provide a clear, colorless oil. H_2O (1.5 mL) was added into the flask which caused the formation of some crystals. The mixture was slowly warmed to approx. 36 °C to yield a clear, colorless solution. This solution was cooled to –20 ^{o}C for 1 h and then warmed to 23 °C. The colorless crystals were quickly collected and dried at 23 °C for 45 min to provide **Z-(10)** along with a small amount of the corresponding boroxine (135 mg, 1.184 mmol, 46 %). The stereoisomeric purity of this compound was determined to be > 99 % based on ^{1}H NMR analysis.

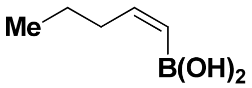

(*Z*)-10

^{1}H NMR (400 MHz, $CDCl_3$)

δ 6.34 (br s, 1H), 5.32 (d, *J* = 13.6 Hz, 1H), 2.31 (qd, *J* = 7.4, 1.5 Hz, 2H), 1.44 (app sext, *J* = 7.4 Hz, 2H), 0.93 (t, *J* = 7.4 Hz, 3H)

^{13}C NMR (100 MHz, $CDCl_3$)

δ 152.7, 34.2, 22.7, 13.7

Suzuki-Miyaura Cross-Couplings

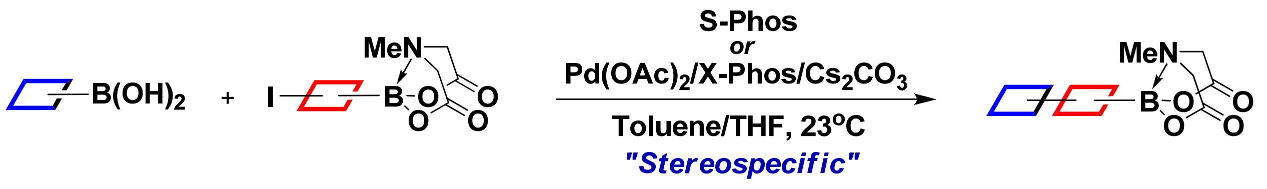

Catalyst solutions were prepared as follows:

Pd/S-Phos catalyst solution : An oven-dried Wheaton vial equipped with a magnetic stir bar was charged with $Pd(OAc)_2$ (5.60 mg, 0.025 mmol, 1.0 eq.) and 2-dicyclohexylphosphino-2',6'-dimethoxy-1,1'-biphenyl (S-Phos) (20.5 mg, 0.050 mmol, 2.0 eq.). Toluene (3.00 mL) was added and the vial was sealed with a PTFE-lined plastic cap. The resulting mixture was stirred at 23 °C for 45 min. resulting in a yellow Pd/S-Phos catalyst solution (0.00833 N Pd in toluene).

Pd/X-Phos catalyst solution : An oven-dried Wheaton vial equipped with a magnetic stir bar was charged with $Pd(OAc)_2$ (5.60 mg, 0.025 mmol, 1.0 eq.) and 2-dicyclohexylphosphino-2'.4',6'-tri-*iso*-propyl-1,1'-biphenyl (X-Phos) (24.5 mg, 0.050 mmol, 2.0 eq.). Toluene (3.0 mL) was added and the vial was sealed with a PTFE-lined plastic cap. The resulting mixture was stirred at 23 °C for 1 h to yield a reddish Pd/X-Phos catalyst solution (0.00833 N Pd in toluene).

These catalyst solutions were then immediately utilized in the following cross-coupling procedures.

(*E*,*E*)-11

An oven-dried Wheaton vial equipped with a magnetic stir bar was charged with **(*E*)-1** (30.9 mg, 0.10 mmol, 1.0 eq.), (*E*)-1-pentenylboronic acid **(*E*)-10** (17.1 mg, 0.15 mmol, 1.5 eq.), Cs_2CO_3 (97.7 mg, 0.30 mmol, 3.0 eq.), THF (0.89 mL) and the Pd/S-Phos catalyst solution (0.36 mL, 0.003 mmol, 3.0 mol% Pd). The vial was then sealed with a PTFE-lined plastic cap, and the reddish reaction mixture was stirred at 23 °C for 24 h. The resulting heterogeneous mixture was diluted with acetonitrile (1.0 mL) and the crude product was immediately purified by flash chromatography on silica gel (petroleum ether:EtOAc 1:1 → EtOAc → EtOAc:MeCN 9:1) to give the title compound **11** as a colorless crystalline solid (23.8 mg, 0.095 mmol, 95 %). Spectral data for **11** were consistent with those previously reported by our laboratories.[10]

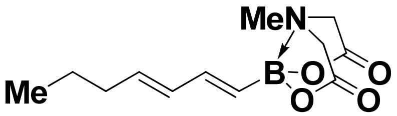

11

(*Z*,*E*)-12

An oven-dried Wheaton vial equipped with a magnetic stir bar was charged with **(*Z*)-1** (30.9 mg, 0.10 mmol, 1.0 eq.), (*E*)-1-pentenylboronic acid **(*E*)-10** (34.1 mg, 0.30 mmol, 3.0 eq.), Cs_2CO_3 (97.7 mg, 0.30 mmol, 3.0 eq.), THF (0.89 mL) and the Pd/X-Phos catalyst solution (0.36 mL, 0.003 mmol, 3.0 mol% Pd). The vial was then sealed with a PTFE-lined plastic cap, and the reddish reaction mixture was stirred at 23 °C for 24 h. The resulting heterogeneous orange mixture was diluted with acetonitrile (1.0 mL) and

[10] Lee, S.J.; Gray, K.C.; Paek, J.S.; Burke, M.D. *J. Am. Chem. Soc.* **2008**, *130*, 466-468

the crude product was immediately purified by flash chromatography on Florisil® (petroleum ether:EtOAc 1:1 → EtOAc → EtOAc:MeCN 9:1) to give the title compound **12** as a colorless crystalline solid (19.3 mg, 0.077 mmol, 77 %).

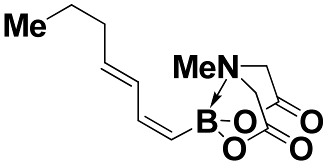

12

TLC (EtOAc)

R_f = 0.53, visualized by UV lamp (λ = 254 nm) *or* with $KMnO_4$

^{1}H NMR (500 MHz, CD_3CN)

δ 6.66 (app t, *J* = 12.25 Hz, 1H), 6.50 (app ddq, *J* = 15, 11, 1.5 Hz, 1H), 5.77 (dt, *J* = 15, 7 Hz, 1H), 5.19 (d, *J* = 14 Hz, 1H), 3.95 (d, *J* = 17 Hz, 2H), 3.78 (d, *J* = 17 Hz, 2H), 2.78 (s, 3H), 2.07 (qd, *J* = 7.5, 1.25 Hz, 2H), 1.40 (app sext, *J* = 7.25, 2H), 0.89 (t, *J* = 7.25 Hz, 3H)

^{13}C NMR (MHz,)

δ 169.1, 145.2, 139.1, 130.2, 62.3, 47.4, 35.3, 22.9, 13.9

HRMS (ESI)

Calculated for C12H19BNO4 $(M+H)^+$:	252.1407
Found:	252.1415

(*E*,*E*,*E*)-13

An oven-dried Wheaton vial equipped with a magnetic stir bar was charged with **(*E*,*E*)-3** (33.4 mg, 0.10 mmol, 1.0 eq.), (*E*)-1-pentenylboronic acid **(*E*)-10** (25.6 mg, 0.225 mmol, 2.25 eq.), Cs_2CO_3 (97.7 mg, 0.30 mmol, 3.0 eq.), THF (0.89 mL) and the Pd/S-Phos catalyst solution (0.36 mL, 0.003 mmol, 3.0 mol% Pd). The vial was then sealed with a PTFE-lined plastic cap, and the reddish reaction mixture was stirred at 23 °C for 20 h. The resulting heterogeneous yellow mixture was diluted with acetonitrile (1.0 mL) and the crude product was immediately purified by flash chromatography on Florisil® (petroleum ether:EtOAc 1:1 → EtOAc → EtOAc:MeCN 9:1) to give the title compound **(*E*,*E*,*E*)-13** as a light yellow crystalline solid (20.8 mg, 0.0753 mmol, 75 %).

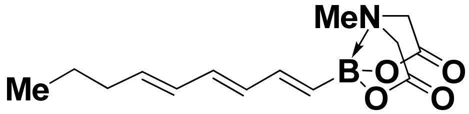

13

TLC (EtOAc)

R_f = 0.56, visualized by UV lamp (λ = 254 nm) *or* with $KMnO_4$

^{1}H NMR (500 MHz,CD_3CN)

δ 6.56 (dd, *J* = 17, 10 Hz, 1H), 6.29 (dd, *J* = 15, 10 Hz, 1H), 6.20 (dd, *J* = 15, 10.5 Hz, 1H), 6.11 (dd, *J* = 15.25, 10.25, 1H), 5.78 (app dt, *J* = 15, 7.25 Hz, 1H), 5.58 (d, *J* = 17.5 Hz, 1H), 3.93 (d, *J* = 17 Hz, 2H), 3.77 (d, *J* = 17 Hz, 2H), 2.74 (s, 3H), 2.07 (q, *J* = 7.25 Hz, 2H), 1.40 (app sext, *J* = 7.25 Hz, 2H), 0.88 (t, *J* = 7.5 Hz, 3H)

^{13}C NMR (125 MHz, CD_3CN)

δ 169.3, 143.9, 137.3, 135.2, 133.3, 131.2, 62.2, 47.5, 35.4, 23.0, 13.8

HRMS (ESI)

Calculated for C14H21BNO4 $(M+H)^+$:	278.1564
Found:	278.1562

(*Z,E,E*)-14

An oven-dried Wheaton vial equipped with a magnetic stir bar was charged with **(*E,Z*)-3** (33.4 mg, 0.10 mmol, 1.0 eq.), (*E*)-1-pentenylboronic acid **(*E*)-10** (25.6 mg, 0.225 mmol, 2.25 eq.), Cs_2CO_3 (97.7 mg, 0.30 mmol, 3.0 eq.), THF (0.89 mL) and the Pd/S-Phos catalyst solution (0.36 mL, 0.003 mmol, 3.0 mol% Pd). The vial was then sealed with a PTFE-lined plastic cap, and the reddish reaction mixture was stirred at 23 °C for 20 h. The resulting heterogeneous yellow mixture was diluted with acetonitrile (1.0 mL) and the crude product was immediately purified by flash chromatography on Florisil® (petroleum ether:EtOAc 1:1 → EtOAc → EtOAc:MeCN 9:1) to give the title compound **(*Z,E,E*)-14** as a colorless crystalline solid (21.7 mg, 0.0783 mmol, 78 %).

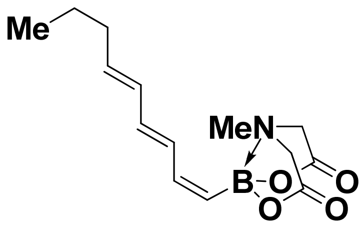

14

TLC (EtOAc)

$R_f = 0.56$, visualized by UV lamp (λ = 254 nm) *or* with $KMnO_4$

^{1}H NMR (500 MHz, CD_3CN)

δ 6.70 (app t, *J* = 12.5 Hz, 1H), 6.58 (dd, *J* = 14.5, 11.5 Hz, 1H), 6.25 (dd, *J* = 14.75, 10.75 Hz, 1H), 6.13 (ddt, *J* = 15. 10.75, 1.25 Hz, 1H), 5.79 (app dt, *J* = 15, 7 Hz, 1H), 5.29 (d, *J* = 13.5 Hz, 1H), 3.95 (d, *J* = 17 Hz, 2H), 3.79 (d, *J* = 17 Hz, 2H), 2.78 (s, 3H), 2.08 (qd, *J* = 7.25, 1 Hz, 2H), 1.40 (sext, *J* = 7.5 Hz, 2H), 0.88 (t, *J* = 7.5 Hz, 3H)

^{13}C NMR (125 MHz, CD_3CN)

δ 169.1, 144.8, 137.4, 137.0, 131.4, 130.2, 62.4, 47.5, 35.4, 23.0, 13.8

HRMS (ESI)

Calculated for $C_{14}H_{21}BNO_4$ $(M+H)^+$:	278.1564
Found:	278.1564

(*E,Z,E*)-15

An oven-dried Wheaton vial equipped with a magnetic stir bar was charged with **(*Z,E*)-3** (33.4 mg, 0.10 mmol, 1.0 eq.), (*E*)-1-pentenylboronic acid **(*E*)-10** (34.2 mg, 0.300 mmol, 3.00 eq.), Cs_2CO_3 (97.7 mg, 0.300 mmol, 3.0 eq.), THF (0.89 mL) and the Pd/X-Phos catalyst solution (0.36 mL, 0.003 mmol, 3.0

mol% Pd). The vial was then sealed with a PTFE-lined plastic cap, and the reddish reaction mixture was stirred at 23 °C for 20 h. The resulting heterogeneous yellow mixture was diluted with acetonitrile (1.0 mL) and the crude product was immediately purified by flash chromatography on Florisil® (petroleum ether:EtOAc 1:1 → EtOAc → EtOAc:MeCN 9:1) to give the title compound **15** as a colorless crystalline solid (24.2 mg, 0.0873 mmol, 87 %).

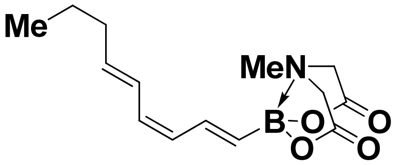

15

TLC (EtOAc)

R_f = 0.56, visualized by UV lamp (λ = 254 nm) *or* with $KMnO_4$

^{1}H NMR (500 MHz, CD_3CN)

δ 7.02 (dd, *J* = 17.25, 10.75 Hz, 1H), 6.61 (ddt, *J* = 15, 10.5, 1.25 Hz, 1H), 6.02-5.92 (m, 2H), 5.79 (app dt, *J* = 15, 7 Hz, 1H), 5.61 (d, *J* = 17.5 Hz, 1H), 3.94 (d, *J* = 17 Hz, 2H), 3.79 (d, *J* = 17 Hz, 2H), 2.75 (s, 3H), 2.11 (app qd, *J* = 7.25, 1Hz, 2H), 1.41 (app sext, *J* = 7.25 Hz, 2H), 0.90 (*J* = 7.25 Hz, 3H)

^{13}C NMR (125 MHz, CD_3CN)

δ 169.3, 138.6, 138.0, 131.5, 130.1, 126.8, 62.3, 47.6, 35.4, 23.1, 13.9

HRMS (ESI)

Calculated for $C_{14}H_{21}BNO_4$ $(M+H)^+$:	278.1564
Found:	278.1567

(*Z*,*Z*,*E*)-16

An oven-dried Wheaton vial equipped with a magnetic stir bar was charged with **(*Z*,*Z*)-3** (33.4 mg, 0.10 mmol, 1.0 eq.), (*E*)-1-pentenylboronic acid **(*E*)-10** (34.2 mg, 0.300 mmol, 3.00 eq.), Cs_2CO_3 (97.7 mg, 0.300 mmol, 3.0 eq.), THF (0.89 mL) and the Pd/X-Phos catalyst solution (0.36 mL, 0.003 mmol, 3.0 mol% Pd). The vial was then sealed with a PTFE-lined plastic cap, and the reddish reaction mixture was stirred at 23 °C for 22 h. The resulting heterogeneous yellow mixture was diluted with acetonitrile (1.0 mL) and the crude product was immediately purified by flash chromatography on Florisil® (petroleum ether:EtOAc 1:1 → EtOAc → EtOAc:MeCN 9:1) to give the title compound **16** as a colorless crystalline solid (17.7 mg, 0.0638 mmol, 64 %).

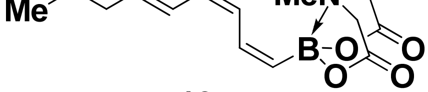

16

TLC (EtOAc)

R_f = 0.56, visualized by UV lamp (λ = 254 nm) *or* with $KMnO_4$

^{1}H NMR (500 MHz, CD_3CN)

δ 7.20 (app t, *J* = 13 Hz, 1H), 6.63 (app ddq, *J* = 15, 11, 1.5 Hz, 1H), 6.33 (app t, *J* 11.75 Hz, 1H), 6.07 (app t, *J* = 11.25 Hz, 1H), 5.81 (app dt, *J* = 15, 7 Hz, 1H), 5.37 (dt, *J* = 14, 1.25 Hz, 1H), 3.95

(d, J = 17 Hz, 2H), 3.78 (d, J = 17 Hz, 2H), 2.79 (s, 3H), 2.11 (app qd, J = 7.5, 1 Hz, 2H), 1.42 (app sext, J = 7.5 Hz, 2H), 0.89 (t, J = 7.5, 3H)

^{13}C NMR (125 MHz, CD_3CN)
δ 169.1, 139.3, 138.3, 132.7, 126.3, 126.1, 62.4, 47.5, 35.5, 23.0, 13.9

HRMS (ESI)

Calculated for $C_{14}H_{21}BNO_4$ $(M+H)^+$:	278.1564
Found:	278.1570

(*E*,*Z*)-17

An oven-dried Wheaton vial equipped with a magnetic stir bar was charged with **(*E*)-1** (30.9 mg, 0.10 mmol, 1.0 eq.), (*Z*)-1-pentenylboronic acid **(*Z*)-10** (22.7 mg, 0.20 mmol, 2.0 eq.), Cs_2CO_3 (97.7 mg, 0.30 mmol, 3.0 eq.), THF (0.89 mL) and the Pd/S-Phos catalyst solution (0.36 mL, 0.003 mmol, 3.0 mol% Pd). The vial was then sealed with a PTFE-lined plastic cap, and the reddish reaction mixture was stirred at 23 °C for 22 h. The resulting heterogeneous yellow mixture was diluted with acetonitrile (1.0 mL) and the crude product was immediately purified by flash chromatography on silica gel (petroleum ether:EtOAc 1:1 → EtOAc → EtOAc:MeCN 9:1) to give the title compound **17** as a colorless crystalline solid (22.8 mg, 0.091 mmol, 91 %).

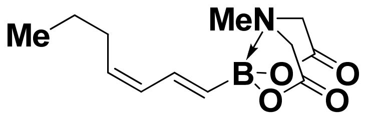

17

TLC (EtOAc)
R_f = 0.53, visualized by UV lamp (λ = 254 nm) *or* with $KMnO_4$

^1H NMR (500 MHz, CD_3CN)
δ 6.86 (dd, J = 17.5, 11 Hz, 1H), 6.07 (app tq, J = 11, 1 Hz, 1H), 5.58 (d, J = 17.5 Hz, 1H), 5.52-5.47 (m, 1H), 3.94 (d, J = 17 Hz, 2H), 3.78 (d, J = 16.5, 2H), 2.75 (s, 3H), 2.20 (app qd, J = 7.5, 1.5 Hz, 2H), 1.40 (app sext, J = 7.5 Hz, 2H), 0.90 (t, J = 7.5 Hz, 3H)

^{13}C NMR (125 MHz, CD_3CN)
δ 169.3, 138.8, 134.1, 131.7, 62.3, 47.6, 30.3, 23.4, 13.9

HRMS (ESI)

Calculated for $C_{12}H_{19}BNO_4$ $(M+H)^+$:	252.1407
Found:	252.1397

(*Z*,*Z*)-18

An oven-dried Wheaton vial equipped with a magnetic stir bar was charged with **(*Z*)-1** (30.9 mg, 0.10 mmol, 1.0 eq.), (*Z*)-1-pentenylboronic acid **(*Z*)-10** (34.1 mg, 0.30 mmol, 3.0 eq.), Cs_2CO_3 (97.7 mg, 0.30 mmol, 3.0 eq.), THF (0.89 mL) and the Pd/X-Phos catalyst solution (0.36 mL, 0.003 mmol, 3.0 mol% Pd). The vial was then sealed with a PTFE-lined plastic cap, and the reddish reaction mixture was stirred at 23 °C for 24 h. The resulting heterogeneous yellow mixture was diluted with acetonitrile (1.0 mL) and

the crude product was immediately purified by flash chromatography on Florisil® (petroleum ether:EtOAc 1:1 → EtOAc → EtOAc:MeCN 9:1) to give the title compound **18** as a colorless crystalline solid (18.6 mg, 0.074 mmol, 74 %).

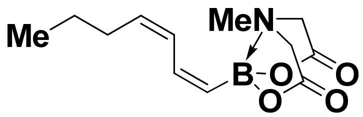

18

TLC (EtOAc)

R_f = 0.53, visualized by UV lamp (λ = 254 nm) *or* with $KMnO_4$

^{1}H NMR (500 MHz, CD_3CN)

δ 7.03 (app t, *J* = 13 Hz, 1H), 6.44 (app tq, *J* = 11, 1.5 Hz, 1H), 5.61-5.55 (m, 1H), 5.34 (dd, *J* = 14, 1 Hz, 1H), 3.95 (d, *J* = 17 Hz, 2H), 3.78 (d, *J* = 17 Hz, 2H), 2.79 (s, 3H), 2.18 (app qd, *J* = 7.5, 1.5 Hz, 2H), 1.40 (app sext, *J* = 7.25 Hz, 2H), 0.90 (t, *J* = 7.5 Hz, 3H),

^{13}C NMR (125 MHz, CD_3CN)

δ 169.1, 139.6, 135.5, 128.0, 62.4, 47.5, 29.6, 23.4, 13.9

HRMS (ESI)

Calculated for $C_{12}H_{19}BNO_4$ $(M+H)^+$:	252.1407
Found:	252.1413

(*E,E,Z*)-19

An oven-dried Wheaton vial equipped with a magnetic stir bar was charged with **(*E,E*)-3** (33.4 mg, 0.10 mmol, 1.0 eq.), (*Z*)-1-pentenylboronic acid **(*Z*)-10** (25.6 mg, 0.225 mmol, 2.25 eq.), Cs_2CO_3 (97.7 mg, 0.30 mmol, 3.0 eq.), THF (0.89 mL) and the Pd/S-Phos catalyst solution (0.36 mL, 0.003 mmol, 3.0 mol% Pd). The vial was then sealed with a PTFE-lined plastic cap, and the reddish reaction mixture was stirred at 23 °C for 20 h. The resulting heterogeneous orange mixture was diluted with acetonitrile (1.0 mL) and the crude product was immediately purified by flash chromatography on Florisil® (petroleum ether:EtOAc 1:1 → EtOAc → EtOAc:MeCN 9:1) to give the title compound **19** as a colorless crystalline solid (21.3 mg, 0.0768 mmol, 77 %).

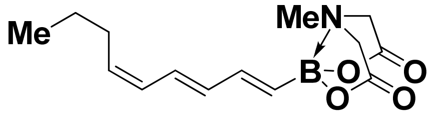

19

TLC (EtOAc)

R_f = 0.56, visualized by UV lamp (λ = 254 nm) *or* with $KMnO_4$

^{1}H NMR (500 MHz, CD_3CN)

δ 6.66-6.61 (m, 2H), 6.28 (dd, *J* = 15, 10.5 Hz, 1H), 6.06 (app t, *J* = 11 Hz, 1H), 5.63 (d, *J* = 17.5 Hz, 1H), 5.55-5.49 (m, 1H), 3.94 (d, *J* = 17 Hz, 2H), 3.78 (d, *J* = 17 Hz, 2H), 2.75 (s, 3H), 2.18 (app qd, *J* = 7.5, 1 Hz, 2H), 1.39 (app sext, *J* = 7.25 Hz, 2H), 0.89 (t, *J* = 7.5 Hz, 3H)

^{13}C NMR (125 MHz, CD_3CN)

δ 169.3, 143.9, 135.2, 134.7, 130.3, 129.4, 62.2, 47.5, 30.3, 23.4, 13.8

HRMS (ESI)

Calculated for $C_{14}H_{21}BNO_4$ $(M+H)^+$: 278.1564

Found: 278.1569

(*Z*,*E*,*Z*)-20

An oven-dried Wheaton vial equipped with a magnetic stir bar was charged with **(*E*,*Z*)-3** (33.4 mg, 0.10 mmol, 1.0 eq.), (*Z*)-1-pentenylboronic acid **(*Z*)-10** (34.2 mg, 0.300 mmol, 3.00 eq.), Cs_2CO_3 (97.7 mg, 0.30 mmol, 3.0 eq.), THF (0.89 mL) and the Pd/S-Phos catalyst solution (0.36 mL, 0.003 mmol, 3.0 mol% Pd). The vial was then sealed with a PTFE-lined plastic cap, and the reddish reaction mixture was stirred at 23 °C for 26 h. The resulting heterogeneous yellow mixture was diluted with acetonitrile (1.0 mL) and the crude product was *immediately* purified by flash chromatography on Florisil® (petroleum ether:EtOAc 1:1 → EtOAc → EtOAc:MeCN 9:1) to give the title compound **20** as a colorless crystalline solid (23.2 mg, 0.0837 mmol, 84 %).

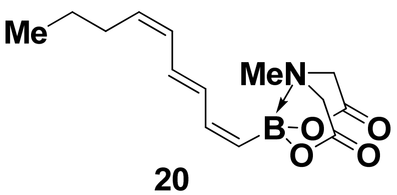

20

TLC (EtOAc)

$R_f = 0.56$, visualized by UV lamp (λ = 254 nm) *or* with $KMnO_4$

^{1}H NMR (500 MHz, CD_3CN)

δ 6.77 (app t, *J* = 14.5 Hz, 1H), 6.69-6.57 (m, 2H), 6.07 (app tt, *J* = 10.75, 1.5 Hz, 1H), 5.54-5.49 (m, 1H), 5.34 (d, *J* = 13.5 Hz, 1H), 3.95 (d, *J* = 16.5 Hz, 2H), 3.79 (d, *J* = 17 Hz, 2H), 2.79 (s, 3H), 2.19 (app qd, *J* = 7.75, 1.25 Hz, 2H), 1.40 (app sext, *J* = 7.25 Hz, 2H), 0.90 (t, *J* = 7.25 Hz, 3H)

^{13}C NMR (125 MHz, CD_3CN)

δ 169.1, 144.8, 134.7, 132.1, 132.0, 129.6, 62.4, 47.5, 30.4, 23.4, 13.9

HRMS (ESI)

Calculated for $C_{14}H_{21}BNO_4$ $(M+H)^+$: 278.1564

Found: 278.1563

(*E*,*Z*,*Z*)-21

An oven-dried Wheaton vial equipped with a magnetic stir bar was charged with **(*Z*,*E*)-3** (33.4 mg, 0.10 mmol, 1.0 eq.), (*Z*)-1-pentenylboronic acid **(*Z*)-10** (34.2 mg, 0.300 mmol, 3.00 eq.), Cs_2CO_3 (97.7 mg, 0.300 mmol, 3.0 eq.), THF (0.89 mL) and the Pd/X-Phos catalyst solution (0.36 mL, 0.003 mmol, 3.0 mol% Pd). The vial was then sealed with a PTFE-lined plastic cap, and the reddish reaction mixture was stirred at 23 °C for 20 h. The resulting heterogeneous orange mixture was diluted with acetonitrile (1.0 mL) and the crude product was immediately purified by flash chromatography on Florisil® (petroleum

ether:EtOAc 1:1 → EtOAc → EtOAc:MeCN 9:1) to give the title compound **21** as a colorless crystalline solid (22.7 mg, 0.0819 mmol, 82 %).

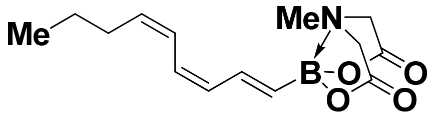

21

TLC (EtOAc)

R_f = 0.56, visualized by UV lamp (λ = 254 nm) *or* with $KMnO_4$

^{1}H NMR (500 MHz, CD_3CN)

δ 7.04 (dd, *J* = 17.5, 11.5 Hz, 1H), 6.55 (app td, *J* = 11.5, 1 Hz, 1H), 6.33 (app td, *J* = 11.5, 0.5 Hz, 1H), 6.07 (app t, *J* = 11 Hz, 1H), 5.67 (d, *J* = 17 Hz, 1H), 5.62 (m, 1H), 3.94 (d, *J* = 17 Hz, 2H), 3.79 (d, *J* = 17 Hz, 2H), 2.75 (s, 3H), 2.18 (app qd, *J* = 7.5, 1.5 Hz, 2H), 1.40 (app sext, *J* = 7.25 Hz, 2H), 0.90 (t, *J* = 7.5 Hz, 3H)

^{13}C NMR (125 MHz, CD_3CN)

δ 169.3, 138.4, 134.9, 131.7, 126.1, 124.6, 62.3, 47.6, 30.0, 23.3, 13.9

HRMS (ESI)

Calculated for $C_{14}H_{21}BNO_4$ $(M+H)^+$:	278.1564
Found:	278.1567

(*Z*,*Z*,*Z*)-22

An oven-dried Wheaton vial equipped with a magnetic stir bar was charged with **(*Z*,*Z*)-3** (33.4 mg, 0.10 mmol, 1.0 eq.), (*Z*)-1-pentenylboronic acid **(*Z*)-10** (34.2 mg, 0.300 mmol, 3.00 eq.), Cs_2CO_3 (97.7 mg, 0.300 mmol, 3.0 eq.), THF (0.89 mL) and the Pd/X-Phos catalyst solution (0.36 mL, 0.003 mmol, 3.0 mol% Pd). The vial was then sealed with a PTFE-lined plastic cap, and the reddish reaction mixture was stirred at 23 °C for 22 h. The resulting heterogeneous orange mixture was diluted with acetonitrile (1.0 mL) and the crude product was immediately purified by flash chromatography on Florisil® (petroleum ether:EtOAc 1:1 → EtOAc → EtOAc:MeCN 9:1) to give the title compound **22** as a colorless crystalline solid (17.2 mg, 0.0620 mmol, 62 %).

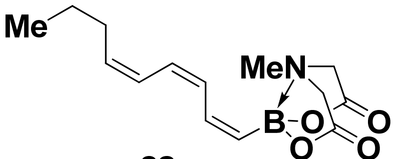

22

TLC (EtOAc)

R_f = 0.56, visualized by UV lamp (λ = 254 nm) *or* with $KMnO_4$

^{1}H NMR (500 MHz, CD_3CN)

δ 7.22 (app t, *J* = 12.75 Hz, 1H), 6.56 (app tt, *J* = 11.5, 1.5 Hz, 1H), 6.49-6.38 (m, 2H), 5.62-5.56 (m, 1H), 5.42 (app dt, *J* = 14, 1.5 Hz, 1H), 3.96 (d, 2H, *J* = 17 Hz, 2H), 3.79 (d, *J* = 16.5 Hz, 2H),

2.79 (s, 3H), 2.18 (app qd, *J* = 7.5, 1.5 Hz, 2H), 1.40 (app sext, *J* = 7.25 Hz, 2H), 0.90 (t, *J* = 7.25 Hz, 3H)

^{13}C NMR (125 MHz, CD_3CN)

δ 169.1, 139.1, 135.2, 127.8, 127.2, 123.9, 62.4, 47.5, 30.0, 23.3, 13.9

HRMS (ESI)

Calculated for $C_{14}H_{21}BNO_4$ $(M+H)^+$:	278.1564
Found:	278.1572

IV. Synthesis of the Heptaene Core of Vacidin A

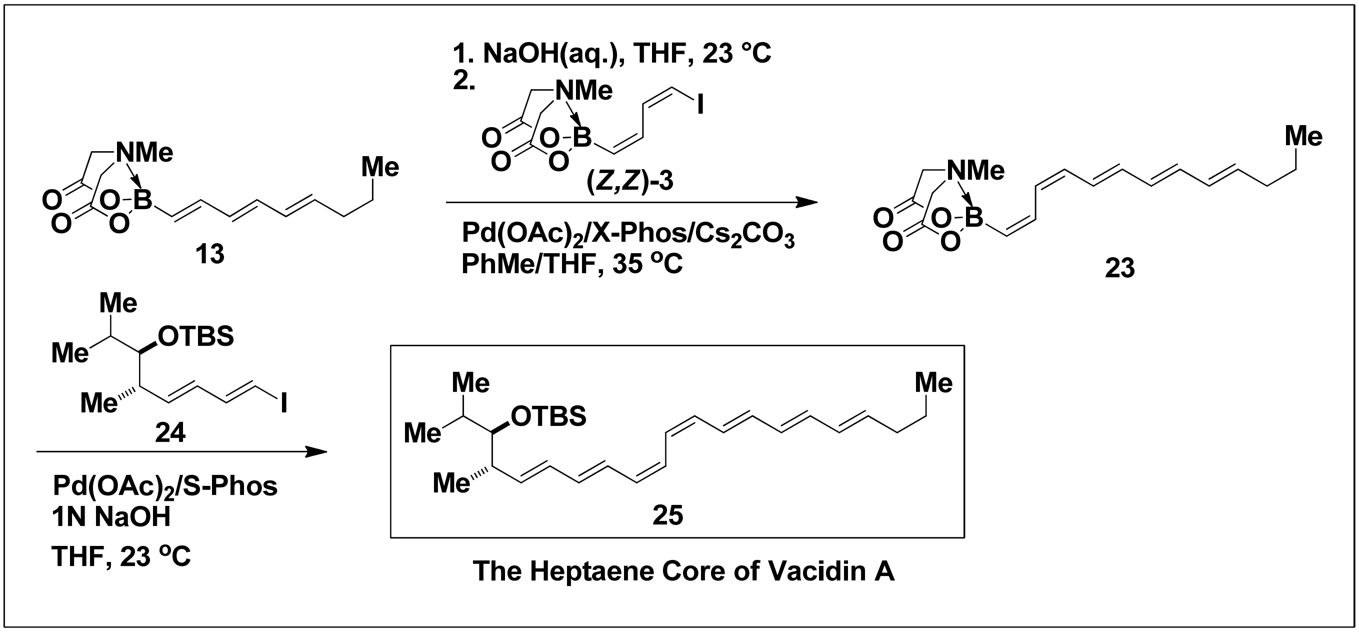

Synthesis of 24

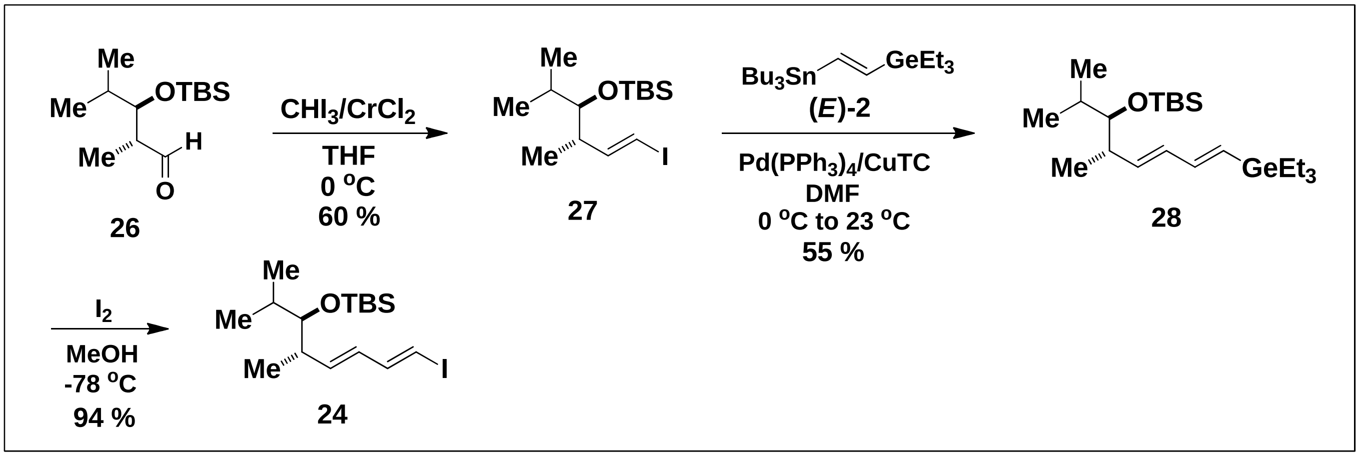

(*E*)-27

An oven-dried 100-ML one-neck round bottom flask equipped with a magnetic stir bar was charged with anhydrous $CrCl_2$ (2.212 g, 18.00 mmol, 6.0 eq.) and capped with rubber septum in an argon-filled glove box. The reaction flask was cooled to 0 °C and THF (30 mL) was added via syringe. A solution of aldehyde **26** (0.733 g, 3.00 mmol, 1.0 eq.) and CHI_3 (2.362 g, 6.00 mmol, 2.0 eq.) dissolved in THF (15 mL) was dropwise added to the suspension over the course of 30 min at 0 °C and the resulting reddish mixture was stirred for 6 h at 0 °C. The reaction mixture was poured into (sat.) aqueous NaCl (125 mL) and extracted with Et_2O (3 × 150 mL). The combined organic extracts were dried over anhydrous magnesium sulfate, and concentrated *in vacuo* to provide the crude product as a reddish oil. The crude product was purified by flash chromatography on silica gel (petroleum ether) to give the title compound **(*E*)-27** as very slightly pink oil (0.668 g, 1.813 mmol, 60 %).

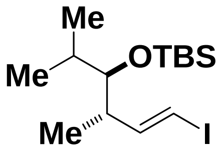

27

TLC (Pet. ether)

R_f = 0.76, visualized by UV lamp (λ = 254 nm).

^{1}H NMR (400 MHz, $CDCl_3$)

δ 6.51 (dd, *J* = 14.4, 8.8 Hz, 1H), 5.93 (dd, *J* = 14.8, 0.8 Hz, 1H), 3.24 (app t, *J* = 4.8 Hz, 1H), 2.39-2.33 (m, 1H), 1.75-1.67 (m, 1H), 0.97 (d, *J* = 7.2 Hz, 3H), 0.89 (s, 9H), 0.85 (d, *J* = 8.5 Hz, 3H), 0.84 (d, *J* = 8.5 Hz, 3H), 0.03 (s, 3H), 0.02 (s, 3H)

^{13}C NMR (100 MHz, $CDCl_3$)

δ 149.7, 80.1, 74.4, 44.6, 32.1, 26.1, 19.8, 18.4, 17.9, 17.8, -3.6, -3.7

***(E,E)*-28**

A 25-ML Wheaton vial equipped with a magnetic stir bar was charged with $Pd(PPh_3)_4$ (89.0 mg, 0.077 mmol, 5 mol% Pd) and copper(I) thiophene-2-carboxylate (Cu(TC)) (439.0 mg, 2.301 mmol, 1.50 eq.) and the vial was then sealed with a PTFE-lined plastic cap in an argon-filled glove box. The reaction mixture was cooled to 0 °C and DMF (4.0 mL) was added via syringe under nitrogen. **27** (565 mg, 1.534 mmol, 1.0 eq.) dissolved in DMF (2.0 mL) and ***(E)*-2** (840 mg, 1.764 mmol, 1.15 eq.) dissolved in DMF (1.70 mL) were then added via syringe. The resulting reddish mixture was stirred for 3h 30 min. at 0 °C. Additional ***(E)*-2** (840 mg, 1.764 mmol, 1.15 eq.) was added and the reaction mixture was warmed to 23 °C and allowed to stir for 2 h. The reaction mixture was treated with (sat.) aqueous NaCl (100 mL) and extracted with ethyl acetate (3 × 125 mL). The combined organic phases were dried over anhydrous magnesium sulfate, and concentrated *in vacuo* to provide light yellow oil. The residue of DMF was removed via azeotrope with toluene at 30 °C *in vacuo*. The crude product was purified by flash chromatography on silica gel (petroleum ether) to give the title compound ***(E,E)*-28** as a colorless oil (361 mg, 0.845 mmol, 55 %).

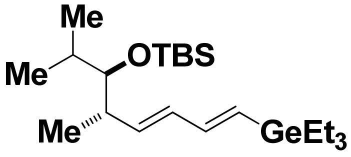

28

TLC (Pet. ether)

R_f = 0.81, visualized by UV lamp (λ = 254 nm).

^{1}H NMR (400 MHz, $CDCl_3$)

δ 6.41 (dd, *J* = 18.4, 10 Hz, 1H), 5.98 (dd, *J* = 15.2, 10 Hz, 1H), 5.82 (d, *J* = 18 Hz, 1H), 5.67 (dd, *J* = 15.6, 8.4 Hz, 1H), 3.26 (app t, *J* = 4.8 Hz, 1H), 2.39-2.34 (m, 1H), 1.76-1.68 (m, 1H), 1.01 (app t, *J* = 7.8 Hz, 9H), 0.99 (d, *J* = 7.2 Hz, 3H), 0.89 (s, 9H), 0.85 (d, *J* = 8.5 Hz, 3H), 0.85 (d, *J* = 8.5 Hz, 3H), 0.77 (app q, *J* = 7.8 Hz, 6H), 0.02 (s, 3H), 0.01 (s, 3H)

^{13}C NMR (100 MHz, $CDCl_3$)

δ 144.6, 137.6, 132.4, 128.9, 81.0, 41.2, 31.9, 26.2, 20.2, 18.4, 18.2, 18.1, 8.9, 4.4, -3.7, -3.7.

HRMS (EI)

Calculated for $C_{22}H_{45}OSiGe$ $(M\text{-}H)^+$:	427.2452
Found:	427.2463

(*E,E*)-24

A 50-ML one-neck round bottom flask with a magnetic stir bar was charged with **(*E,E*)-28** (125 mg, 0.293 mmol, 1.0 eq.) and capped with rubber septum. The reaction flask was evacuated and refilled with nitrogen three-times. CH_2Cl_2 (2.0 mL) and MeOH (5.0 mL) were added into the flask via syringe and the reaction mixture was cooled to –78 °C. I_2 (372 mg, 1.465 mmol, 5.0 eq.) dissolved in MeOH (5.0 mL) was added dropwise into the reaction mixture over the course of 15 min. The reddish reaction mixture was stirred for 3 h at –78 °C under nitrogen. (sat.) Aqueous $Na_2S_2O_3$ (10 mL) was added to the reaction mixture at –78 °C and the resulting yellow solution was warmed to 23 °C. The mixture was treated with additional (sat.) aqueous $Na_2S_2O_3$ (100 mL) and immediately extracted with EtOAc (3 × 125 mL). The combined organic extracts were washed with (sat.) aqueous $Na_2S_2O_3$ (100 mL) again. The organic layer was dried over anhydrous magnesium sulfate, and concentrated *in vacuo* to provide colorless oil as crude product. The crude product was purified by flash chromatography on Florisil® (petroleum ether) to give the title compound **(*E,E*)-24** as a colorless oil (109 mg, 0.276 mmol, 94 %).

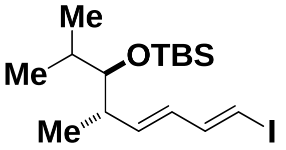

24

TLC (Pet. ether)

R_f = 0.73, visualized by UV lamp (λ = 254 nm).

^1H NMR (400 MHz, $CDCl_3$)

δ 6.98 (dd, *J* = 14.4, 10 Hz, 1H), 6.13 (d, *J* = 14.4, 1H), 5.91 (dd, *J* = 15.4, 10.2 Hz, 1H), 5.76 (dd, *J* = 15.4, 8.2 Hz, 1H), 3.25 (dd, *J* = 5.4, 3.8 Hz, 1H), 2.37-2.31 (m, 1H), 1.72-1.64 (m, 1H), 0.98 (d, *J* = 7.2 Hz, 3H), 0.89 (s, 9H), 0.83 (app t, *J* = 7.2 Hz, 6H), 0.02 (s, 3H), 0.01 (s, 3H)

^{13}C NMR (100 MHz, $CDCl_3$)

δ 145.8, 139.4, 129.4, 80.7, 76.1, 40.8, 32.3, 26.1, 19.8, 18.4, 18.4, 18.4 -3.7, -3.7.

HRMS (EI)

Calculated for $C_{16}H_{32}OISi$ $(M+H)^+$:	395.1268
Found:	395.1254

(*Z,Z,E,E,E*)-1,3,5,7,9-decapentenyl-(10-propyl) boronate ester (23)

*MIDA boronate **13** was converted to (E,E,E)-1,3,5-nonatrienyl boronic acid via the following procedure:* To a stirred mixture of **13** (74.0 mg, 0.267 mmol, 1.0 eq.) in THF (1.0 mL) at 23 °C was added 1N NaOH (aq.) (0.801 mL, 0.801 mmol, 3.0 eq.) *via* syringe. The reaction mixture was stirred at 23 °C for 15 min. The resulting mixture was treated with sat. NH_4Cl (aq.) (1.50 mL) and diluted with Et_2O (1.0 mL). The

organic layer was separated and aqueous layer was extracted with THF:Et_2O 1:1 (2 × 2.5 mL). The combined organic layers were dried over anhydrous magnesium sulfate. After filtration, the resulting colorless solution was concentrated to ~ 0.50 mL volume of THF *in vacuo*. THF (5.0 mL) was added and concentrated again to ~ 0.25 mL volume of THF *in vacuo*. The isolated yield of boronic acid was assumed to be quantitative, and accordingly a 0.267 N solution of boronic acid in THF (0.267 mmol/1.0 mL of THF) was prepared using a 3.0-ML volumetric vial. This solution was saturated with nitrogen for 30 min. and immediately used in the next reaction without further purification.
TLC (EtOAc) R_f = 0.83, visualized by UV lamp (λ = 254 nm) *or* with $KMnO_4$.

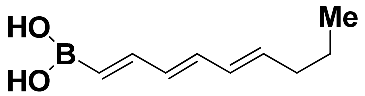

A solution of the catalyst was prepared as follows: A 20-ML Wheaton vial equipped with a magnetic stir bar was charged with $Pd(OAc)_2$ (5.60 mg, 0.025 mmol, 1.0 eq.) and 2-dicyclohexylphosphino-2'.4',6'-tri-*iso*-propyl-1,1'-biphenyl (X-Phos) (24.5 mg, 0.050 mmol, 2.0 eq.). Toluene (2.0 mL) was added and the vial was sealed with a PTFE-lined plastic cap. The resulting mixture was stirred for 1 h at 23 °C to yield a reddish Pd/X-Phos catalyst solution (0.0125 N Pd in toluene).
This catalyst solution was then utilized in the following procedure: A 7.0-ML Wheaton vial equipped with a magnetic stir bar was charged with **(Z,Z)-4** (40.0 mg, 0.120 mmol, 1.0 eq.) and Cs_2CO_3 (117.0 mg, 0.360 mmol, 3.0 eq.). The 0.267 N boronic acid in THF solution (0.267 mmol, 1.0 mL, 2.25 eq.), the catalyst solution (0.480 mL, 0.006 mmol 5.0 mol% Pd) and additional THF (0.20 mL) were then added in order and the vial was sealed with a PTFE-lined plastic cap. The resulting reddish mixture was allowed to stir for 26 h at 35 °C. After being stirred for 26 h, the resulting orange mixture was diluted with EtOAc (1.0 mL) and was immediately purified by flash chromatography on Florisil® (petroleum ether:EtOAc 1:1 → EtOAc → EtOAc:MeCN 9:1, 30 minutes elution time) to give the title compound **23** as a light yellow solid containing a small amount of an unidentified impurity (18.00 mg, 0.0547 mmol, 46 %).

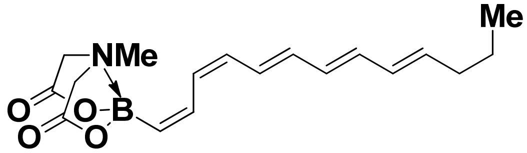

23

TLC (EtOAc)
R_f = 0.54, visualized by UV lamp (λ = 365 nm) *or* with $KMnO_4$

^{1}H NMR (500 MHz, CD_3CN)
δ 7.24 (app t, *J* = 13 Hz, 1H), 6.79 (app t, *J* = 13 Hz, 1H), 6.44 (app t, *J* = 11.75 Hz, 1H), 6.36-6.22 (m, 3H), 6.17-6.06 (m, 2H), 5.81-5.75 (m, 1H), 5.42 (dt, *J* = 14.25, 1.25, 1H), 3.95 (d, *J* = 17 Hz, 2H), 3.79 (d, *J* = 17 Hz, 2H), 2.79 (s, 3H), 2.08 (app qd, *J* = 7.25, 1.25 Hz, 2H), 1.43-1.38 (m, 2H), 0.89 (t, *J* = 7.5 Hz, 3H)

^{13}C NMR (125 MHz, CD_3CN)
δ 169.1, 144.7, 139.2, 137.2, 135.9, 135.4, 132.6, 131.5, 128.1, 127.7, 62.4, 47.5, 35.5, 23.0, 13.9

HRMS (ESI)
Calculated for $C_{18}H_{25}BNO_4$ $(M+H)^+$: 330.1877
Found: 330.1876

The Heptaene Core of Vacidin A (25)

A solution of the Pd/S-Phos catalyst was prepared as follows: An oven-dried 20-ML Wheaton vial equipped with a magnetic stir bar was charged with $Pd(OAc)_2$ (5.60 mg, 0.025 mmol) and 2-dicyclohexylphosphino-2',6'-dimethoxy-1,1'-biphenyl (S-Phos) (20.5 mg, 0.050 mmol). Toluene (3.00 mL) was added and the vial was sealed with a PTFE-lined plastic cap. The resulting mixture was stirred at 23 °C for 45 min resulting in a yellow Pd/S-Phos catalyst solution (0.00833 N Pd in toluene).

This catalyst solution was then utilized in the following procedure: An oven-dried 7.0-ML Wheaton vial equipped with a magnetic stir bar was charged with **24** (11.00 mg, 0.0278 mmol, 1.0 eq.), **23** (17.50 mg, 0.0531 mmol, 1.91 eq.) as a solution in THF (1.70 mL), and the Pd/S-Phos catalyst solution (0.144 mL, 0.0012 mmol, 5.0 mol% Pd). Degassed 1N NaOH (aq.) (0.266 mL, 0.266 mmol, 5.00 eq. based on **23**) was then added into the vial. The vial was sealed with a PTFE-lined plastic cap. The reddish reaction mixture was stirred for 16 h at 23 °C. The resulting heterogeneous deep orange mixture was diluted with ethyl acetate (3.0 mL) and dried over anhydrous magnesium sulfate. The orange solution was filtered through short pad Celite® and the filtrate was concentrated to ~0.5 mL of reddish solution *in vacuo*. The crude product was immediately purified by flash chromatography on Florisil® (petroleum ether:EtOAc 50:1, elution time 5 minutes) to give the title compound **25** as a yellow solid containing a small amount of an unidentified impurity (10.8 mg, 0.0245 mmol, 88 %).

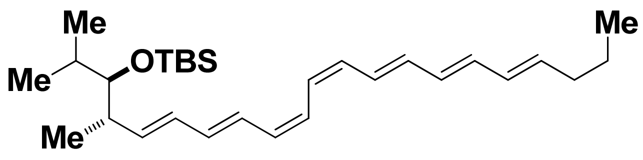

25

TLC (EtOAc: Petroleum ether 1:50)

R_f = 0.39, visualized by UV lamp (λ = 365 nm) *or* with CAM

^{1}H NMR (600 MHz, Pyridine-*d*5:CD_3OD, 9:1) (see below for coupling constants derived from PSCOSY NMR experiments)

δ 7.01-6.88 (m, 2H), 6.72-6.62 (m, 1H), 6.50-6.38 (m, 4H), 6.37-6.35 (m, 1H), 6.32-6.25 (m, 3H), 6.22-6.17 (m, 1H), 5.98-5.91 (m, 1H), 5.79-5.74 (m, 1H), 3.37 (dd, *J* = 4.8, 4.2 Hz, 1H), 2.52-2.48 (m, 1H), 2.04 (app q, *J* = 7.2 Hz, 2H), 1.80-1.75 (m, 1H), 1.35 (app sext, *J* = 7.2 Hz, 2H), 1.07 (d, *J* = 7.2 Hz, 3H), 0.97 (s, 9H), 0.93 (d, *J* = 6.6 Hz, 3H), 0.91 (d, *J* = 7.2 Hz, 3H), 0.85 (app t, *J* = 7.5 Hz, 3H), 0.11 (s, 3H), 0.10 (s, 3H).

^{13}C NMR (150 MHz, Pyridine-*d*5:CD_3OD, 9:1)

δ 140.2, 135.5, 135.3, 135.0, 131.6, 130.9, 130.7, 129.9, 129.0, 128.1, 127.2, 126.8, 124.9, 124.6, 81.4, 42.0, 35.3, 32.6, 30.2, 26.4, 22.8, 20.3, 18.6, 18.5, 13.9, -3.41, -3.54

HRMS (EI)

Calculated for $C_{29}H_{48}OSi$:	440.3475
Found:	440.3476

Stereochemical characterization of 25

The ^1H NMR spectrum of **25** was fully assigned (vacidin A numbering, Figure S1) from gradient COSY spectra (Figure S2, Table S1, see SI.B for full spectra of **25**).

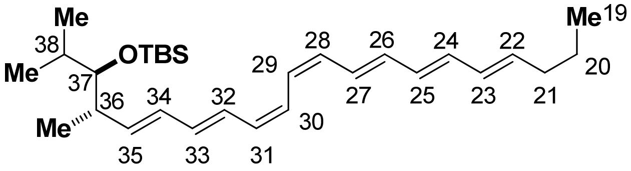

Figure S1. Vacidin A numbering for **25**

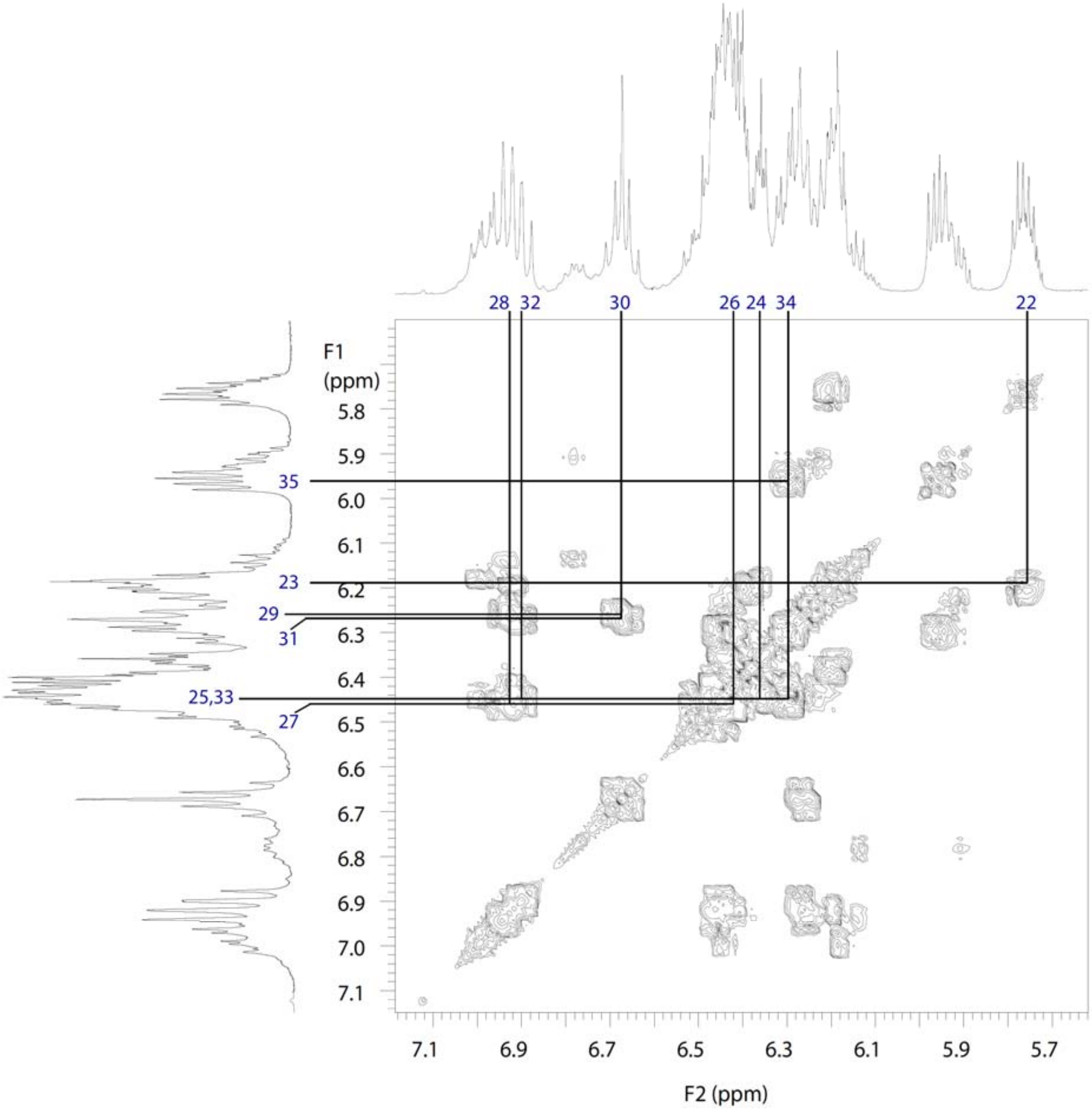

Figure S2. Polyene region of the gradient COSY spectrum of **25** with vinyl proton assignments labeled (vacidin A numbering). See SI.B for the full gradient COSY spectrum.

Table S1. ^{1}H NMR assignments for **25** (Vacidin numbering)

Proton	δ (ppm)
19	0.85
20	1.35
21	2.04
22	5.76
23	6.20
24	6.38
25	6.45
26	6.42
27	6.46
28	6.94
29	6.26
30	6.67
31	6.27
32	6.90
33	6.45
34	6.29
35	5.96
36	2.50
37	3.37
38	1.78
39	0.92
40	1.07

Phase-sensitive (PS) COSY NMR spectroscopy[11] was employed to determine the coupling constants for the majority of the olefinic protons of **25**. The PSCOSY spectrum of **25** was processed using NMRPipe,[12] with diagonal suppression[13] to minimize interference from the diagonal. The Sparky program,[14] version 3.113, was used for peak-picking and measurement of *J* values. The ^{1}H assignments of **25** were verified in the PSCOSY spectrum (Figure S2), and the vinyl H-H J^3 values were measured directly from the PSCOSY spectrum as described by Claridge[11] (Table S2). The 31-30 and 29-28 coupling constants (9.5 and 10.6 Hz, respectively) are consistent with *Z*-olefins. In contrast, the remaining olefins have *J* values consistent with the *E*-geometry (the 27-26 and 25-24 coupling constants were not determined due to interference from the diagonal).

[11] Claridge, T.D.W. High Resolution NMR Techniques in Organic Chemistry. Pergamon: Amsterdam, 1999.
[12] Delaglio, F.; Grzesiek, S.; Vuister, G. W.; Zhu, G.; Pfeifer, J.; Bax, A. *J. Biomol. NMR*, **1995**, *6*, 277-293.
[13] Delaglio, F.; Wu, Z.; Bax, A. *J. Magn. Reson.* **2001**, *149*, 276-281.
[14] Goddard, T. D.; Kneller, D. G. SPARKY 3, University of California, San Francisco, http://www.cgl.ucsf.edu/home/sparky/

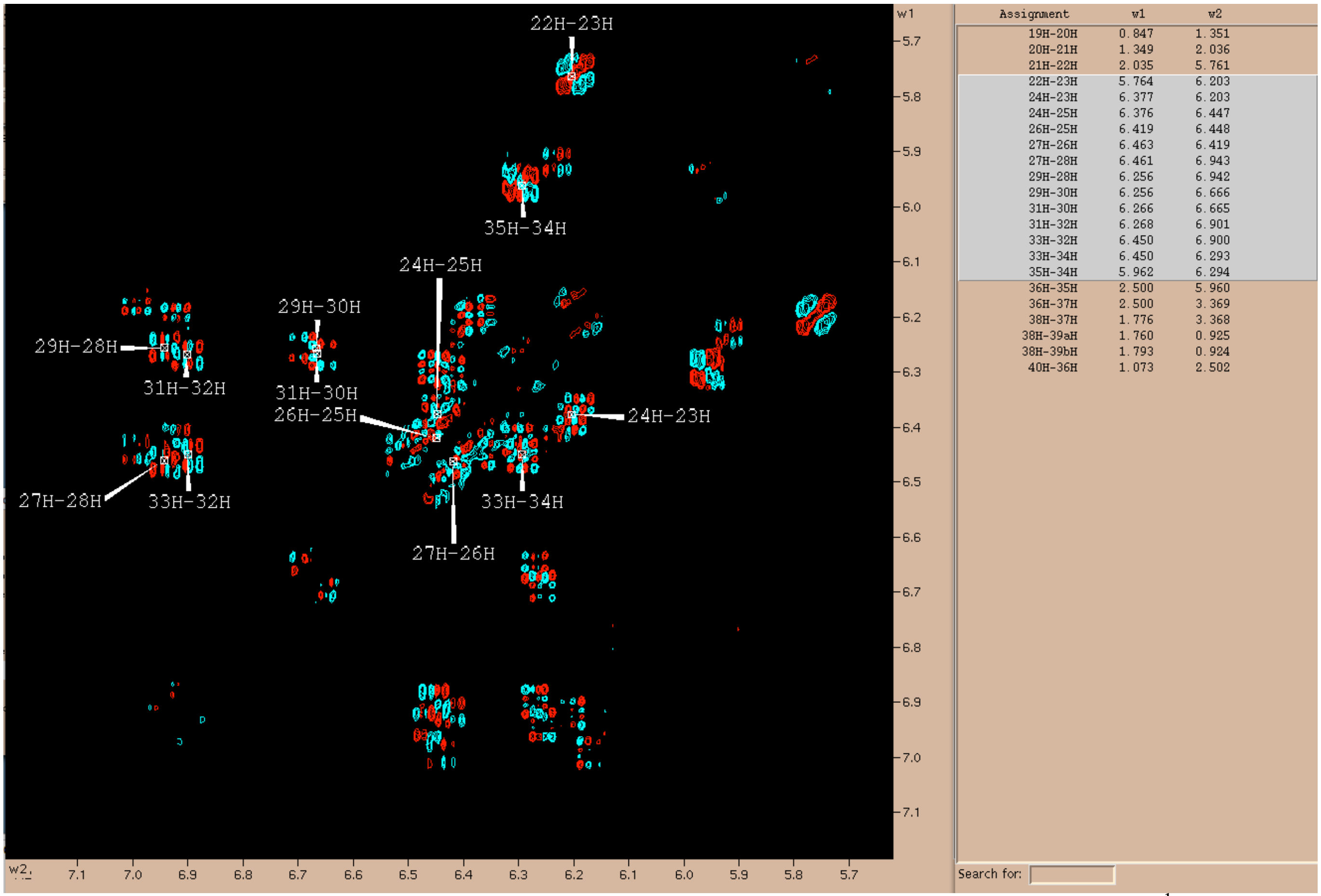

Figure S3. PSCOSY spectrum of the polyene region of **25** with the diagonal suppressed. The ^{1}H assignments were verified and the labeled crosspeaks were used for calculating coupling constants.

Table S2. H-H *J* values for the **25** heptaene measured directly from PSCOSY crosspeaks. Bolded entries correspond to protons about a π-bond.

Crosspeak	^1H-^1H J^3 (Hz)
**35-34**	**16.2**
34-33	10.2
**33-32**	**15.6**
32-31	12.2
**31-30**	**9.5**
30-29	8.2
**29-28**	**10.6**
28-27	14.7
**27-26**	Not determined[a]
26-25	Not determined[a]
**25-24**	Not determined[a]
24-23	8.4
**23-22**	**15.3**

[a]These crosspeaks were too close to the diagonal to accurately measure the coupling constants.

LSJII-63

exp1 std1h

SAMPLE		DEC. & VT	
date	Jun 13 2010	dn	H1
solvent	CD3CN	dof	0
file	/export/home/~	dm	nnn
data/u400/Burke/le~		dmm	c
esj/LSJII-63.fid		dmf	200
ACQUISITION		dpwr	20
sfrq	399.949	PROCESSING	
tn	H1	lb	0.30
at	4.096	wtfile	
np	65536	proc	ft
sw	8000.0	fn	not used
fb	4000	math	f
bs	16		
tpwr	58	werr	
pw	5.8	wexp	svf(n1)
d1	0	wbs	
tof	-425.7	wnt	wft('acq')
nt	32	DISPLAY	
ct	32	sp	-200.1
alock	n	wp	3999.3
gain	not used	vs	162
FLAGS		sc	0
il	n	wc	250
in	n	hzmm	16.00
dp	y	is	500.00
hs	nn	rfl	2425.7
		rfp	0
		th	20
		ins	100.000
		nm	ph

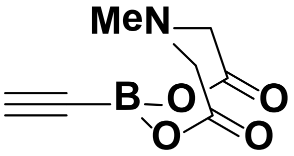

6

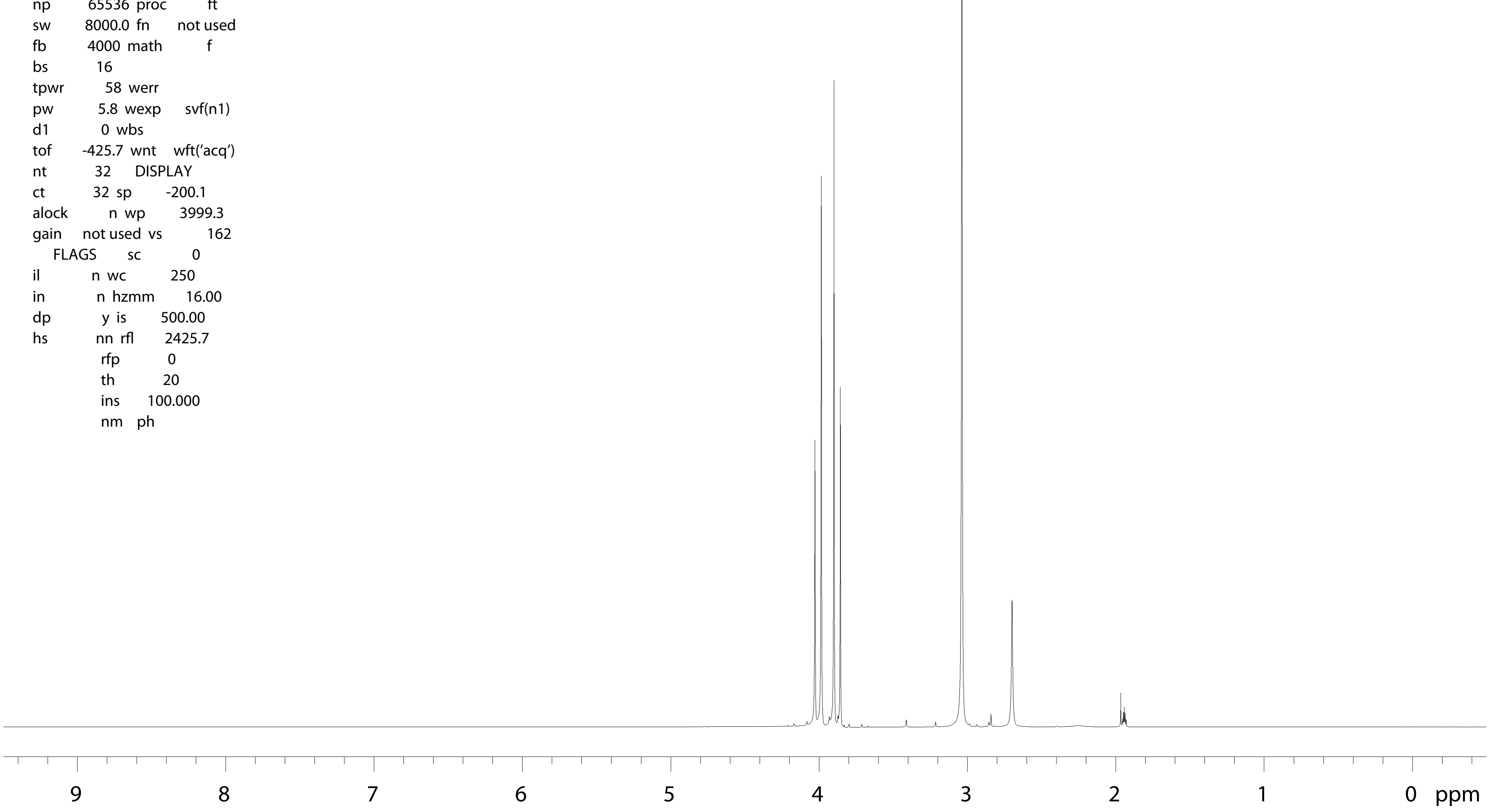

alkynyl_BMIDA_062210

Pulse Sequence: s2pul

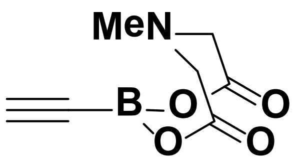

6

(after 6 months storage on the bench in a vial under air in a subdued light environment)

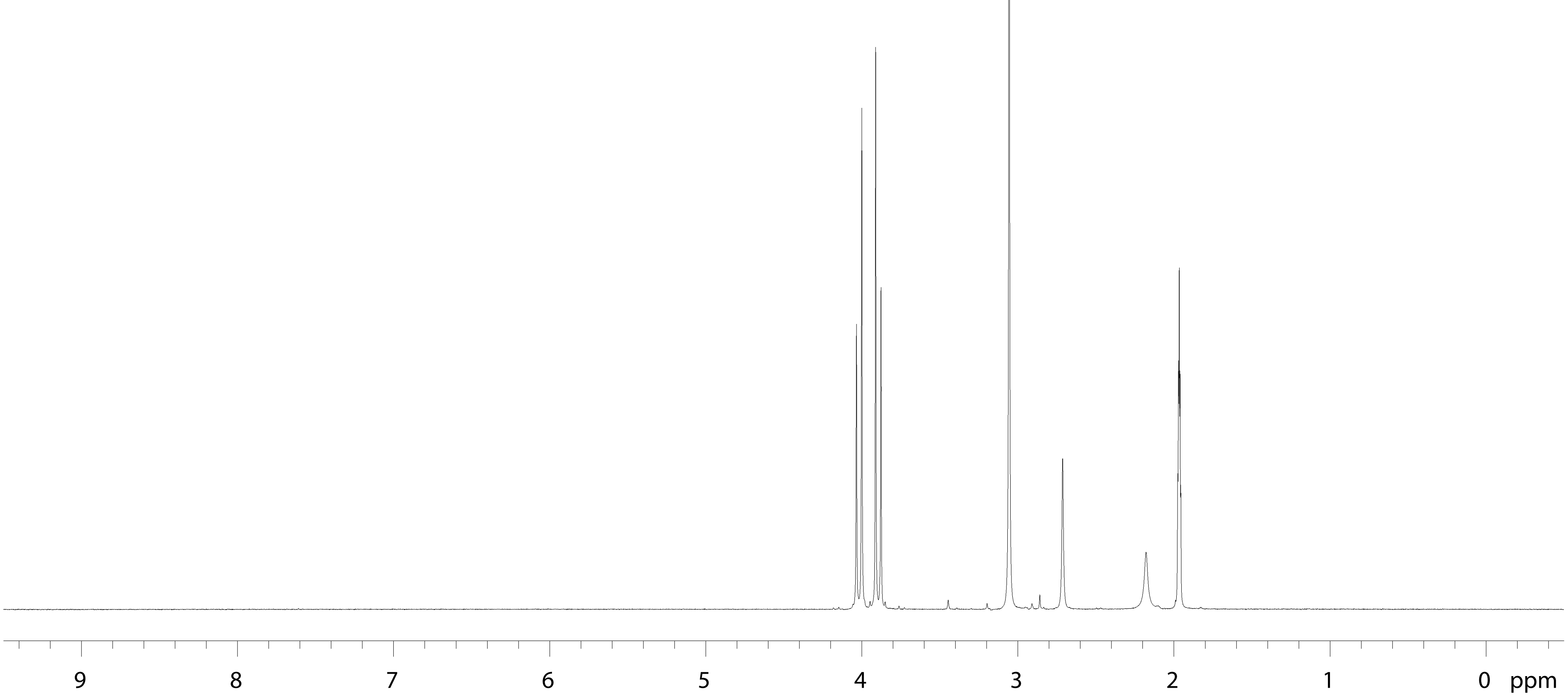

LSJII-63-13C

exp1 std13c

SAMPLE		DEC. & VT	
date	Jun 13 2010	dn	H1
solvent	CD3CN	dof	-1092.3
file	exp	dm	yyy
ACQUISITION		dmm	w
sfrq	100.578	dmf	13889
tn	C13	dpwr	43
at	1.311	PROCESSING	
np	65536	lb	1.00
sw	25000.0	wtfile	
fb	14000	proc	ft
bs	16	fn	not used
tpwr	54	math	f
pw	5.1		
d1	1.000	werr	
tof	1966.4	wexp	
nt	11111	wbs	
ct	2894	wnt	
alock	n	DISPLAY	
gain	not used	sp	-928.1
FLAGS		wp	22995.8
il	n	vs	82
in	n	sc	0
dp	y	wc	250
hs	nn	hzmm	91.98
		is	500.00
		rfl	12852.5
		rfp	11887.0
		th	20
		ins	100.000
		nm	ph

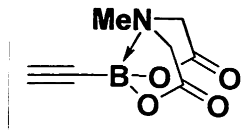

6

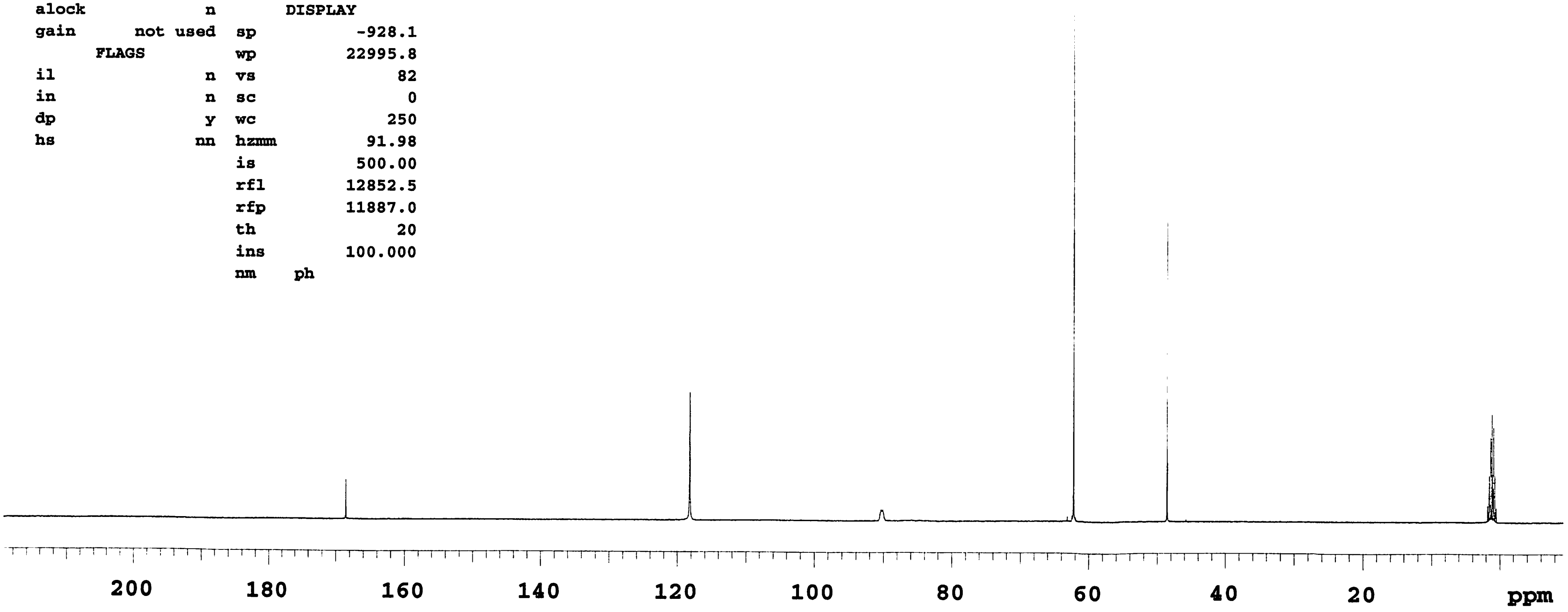

LSJV-33

exp1 s2pul

SAMPLE		DEC. & VT	
date	Jun 14 2010	dfrq	499.696
solvent	CD3CN	dn	H1
file	exp	dpwr	20
ACQUISITION		dof	0
sfrq	499.696	dm	nnn
tn	H1	dmm	c
at	4.665	dmf	200
np	65536	dseq	
sw	7024.9	dres	1.0
fb	4000	homo	n
bs	4	DEC2	
tpwr	63	dfrq2	0
pw	6.5	dn2	
d1	0	dpwr2	1
tof	2.0	dof2	0
nt	32	dm2	n
ct	32	dmm2	c
alock	n	dmf2	200
gain	not used	dseq2	
FLAGS		dres2	1.0
il	n	homo2	n
in	n	PROCESSING	
dp	y	lb	not used
hs	nn	wtfile	
DISPLAY		proc	ft
sp	21.3	fn	not used
wp	5459.1	math	f
vs	15		
sc	0	werr	
wc	250	wexp	svf(n1)
hzmm	21.84	wbs	
is	858.69	wnt	wft('acq')
rfl	1993.8		
rfp	964.4		
th	7		
ins	3.000		
ai	ph		

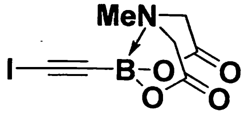

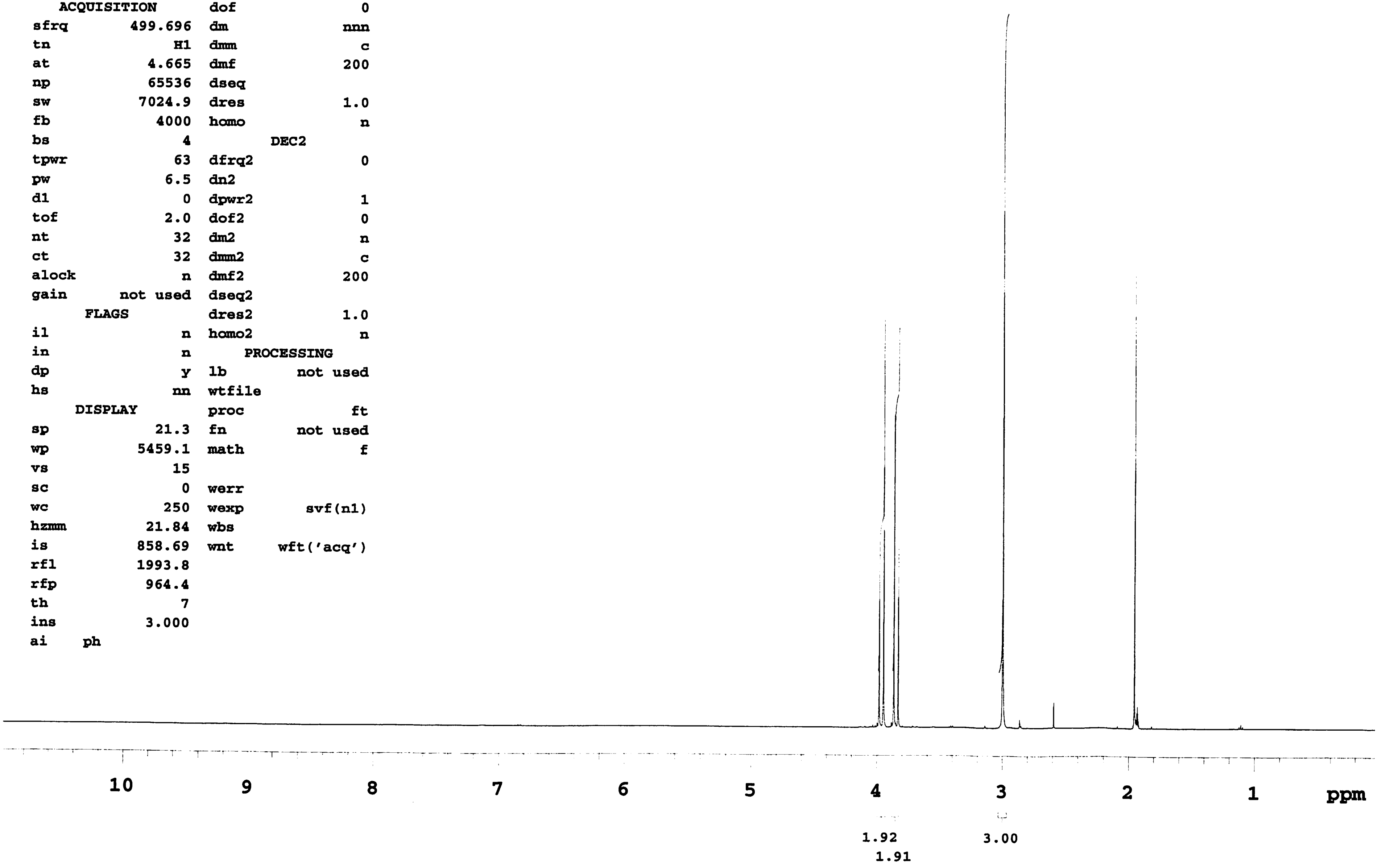

LSJV-33-13C

exp1 s2pul

SAMPLE		DEC. & VT	
date	Jun 14 2010	dfrq	499.695
solvent	CD3CN	dn	H1
file	exp	dpwr	44
ACQUISITION		dof	-827.6
sfrq	125.662	dm	yyy
tn	C13	dmm	w
at	1.086	dmf	19608
np	65536	dseq	
sw	30165.9	dres	90.0
fb	17000	homo	n
bs	16	DEC2	
ss	1	dfrq2	0
tpwr	54	dn2	
pw	6.0	dpwr2	1
d1	1.000	dof2	0
tof	1884.7	dm2	n
nt	11111	dmm2	c
ct	1002	dmf2	10000
alock	n	dseq2	
gain	not used	dres2	1.0
FLAGS		homo2	n
il	n	PROCESSING	
in	n	lb	1.00
dp	y	wtfile	
hs	nn	proc	ft
DISPLAY		fn	not used
sp	-1144.6	math	f
wp	28660.7		
vs	83	werr	
sc	0	wexp	
wc	250	wbs	
hzmm	114.64	wnt	
is	500.00		
rfl	16041.3		
rfp	14851.6		
th	68		
ins	100.000		
nm	ph		

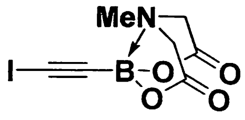

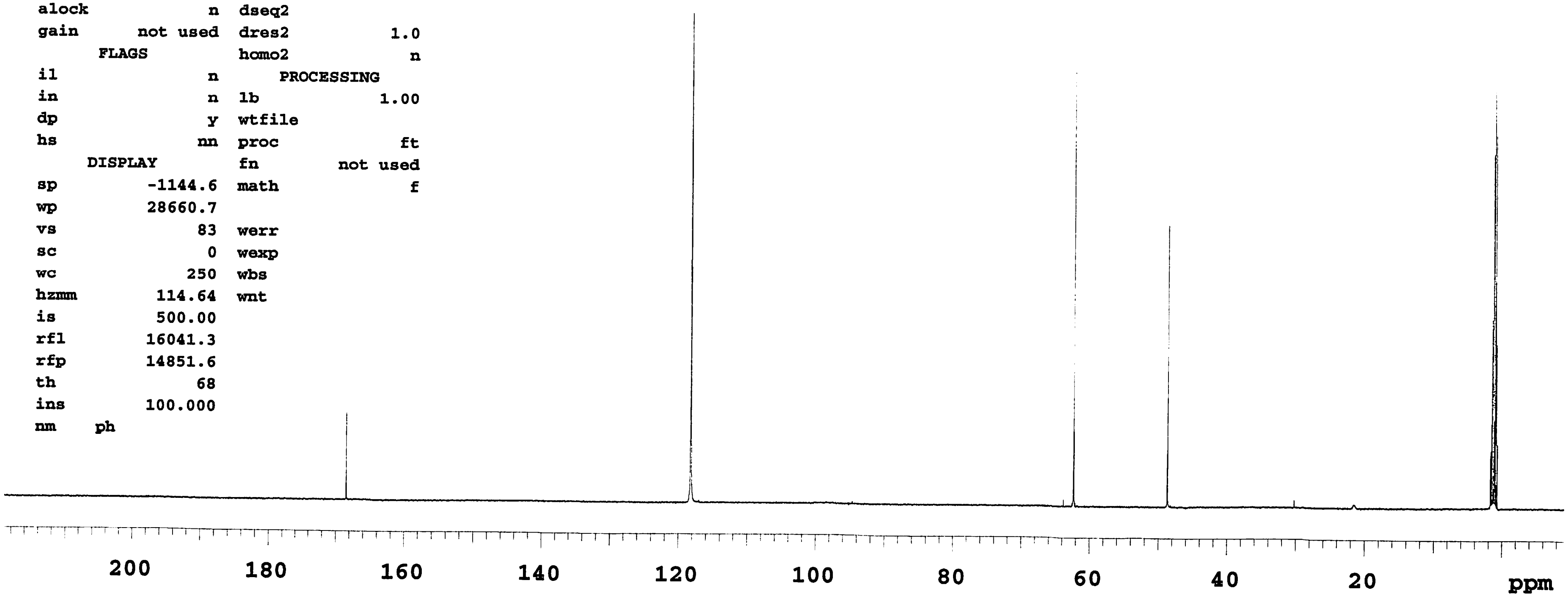

LSJIV-5

exp1 std1h

SAMPLE		DEC. & VT	
date	Jun 13 2010	dn	H1
solvent	CD3CN	dof	0
file	exp	dm	nnn
ACQUISITION		dmm	c
sfrq	399.949	dmf	200
tn	H1	dpwr	20
at	4.096	PROCESSING	
np	65536	lb	not used
sw	8000.0	wtfile	
fb	4000	proc	ft
bs	16	fn	not used
tpwr	58	math	f
pw	5.8		
d1	0	werr	
tof	-425.7	wexp	
nt	32	wbs	
ct	32	wnt	
alock	n	DISPLAY	
gain	not used	sp	45.3
FLAGS		wp	4313.7
il	n	vs	89
in	n	sc	0
dp	y	wc	250
hs	nn	hzmm	17.25
		is	1006.55
		rfl	3201.9
		rfp	771.9
		th	13
		ins	3.000
		nm	ph

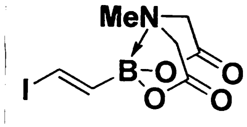

(E)-1

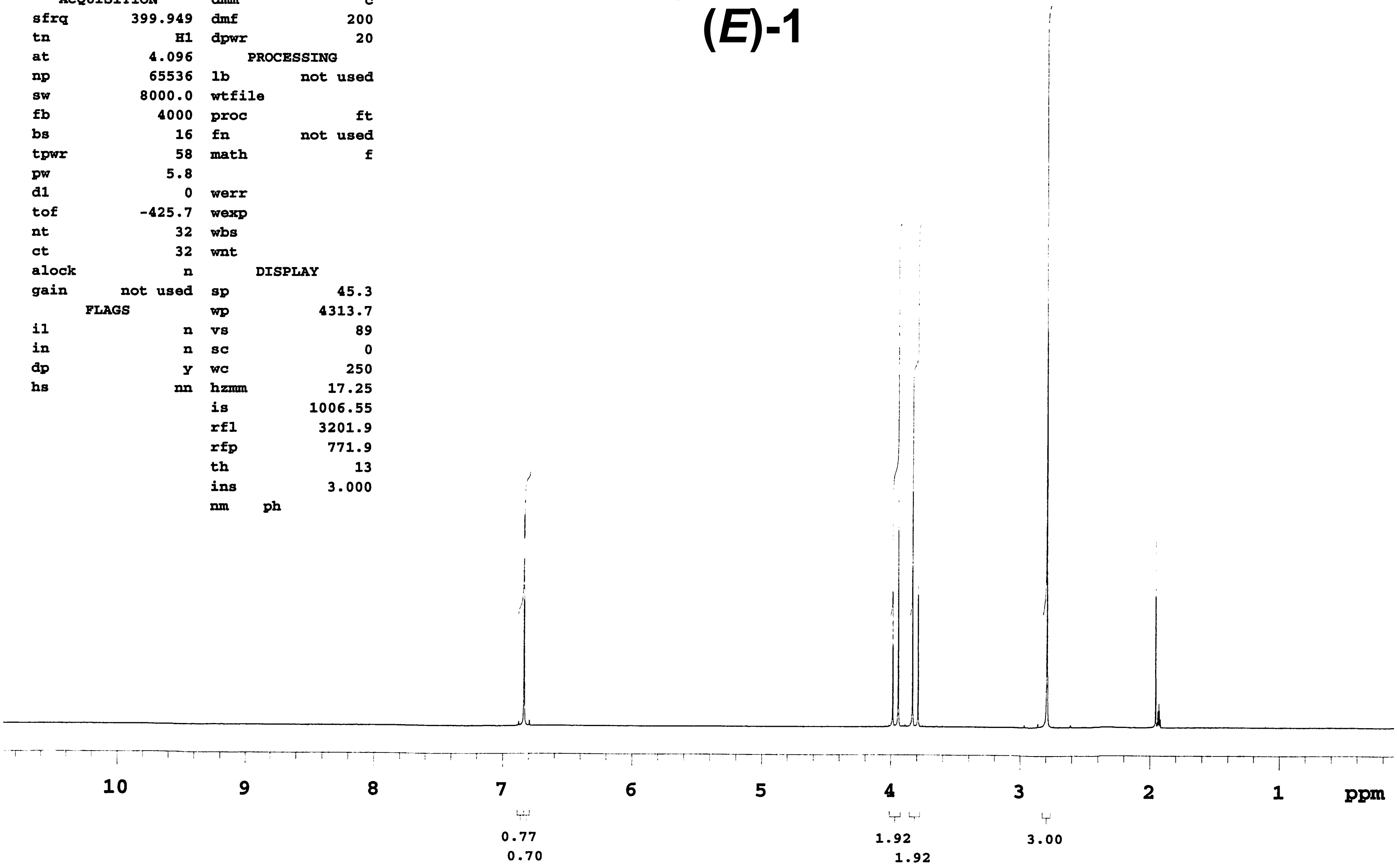

trans_IBB1_062210

Pulse Sequence: s2pul

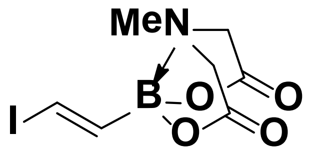

(*E*)-1

(after 6 months storage on the bench in a vial under air in a subdued light environment)

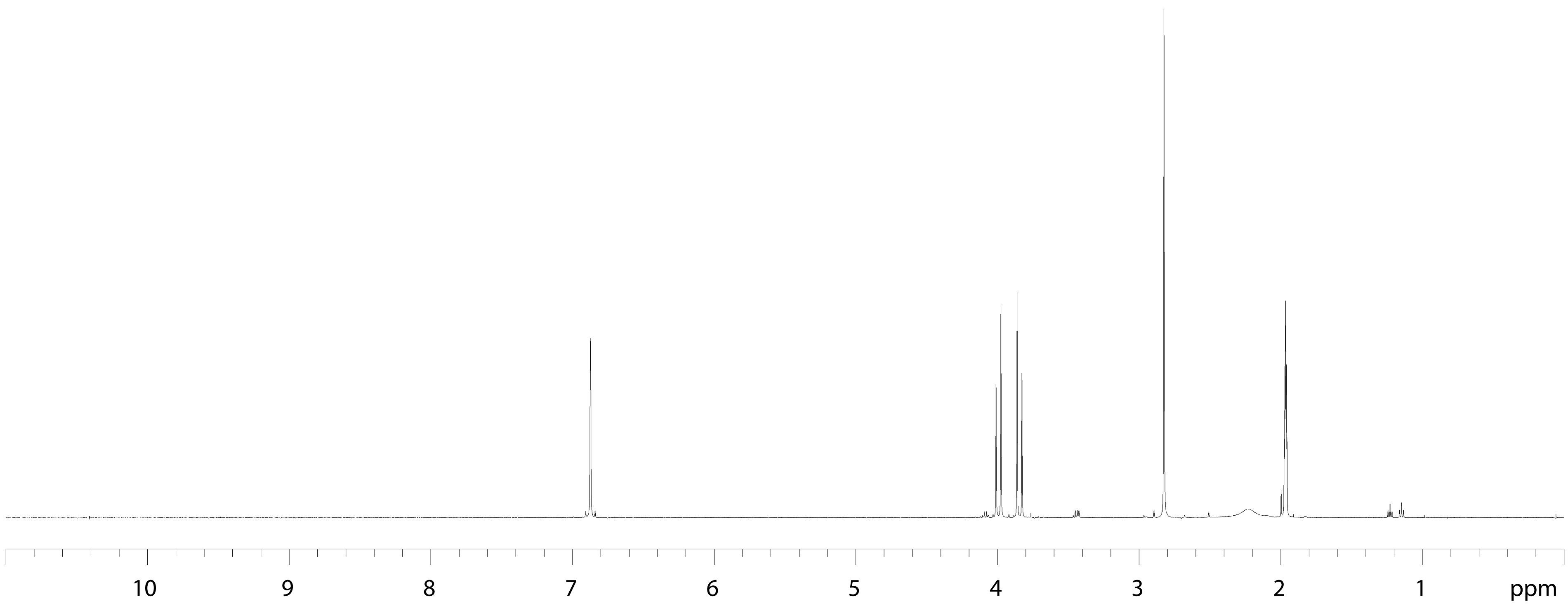

LSJIV-5-13C

exp1 std13c

SAMPLE		DEC. & VT	
date	Jun 13 2010	dn	H1
solvent	CD3CN	dof	-1092.3
file	exp	dm	yyy
ACQUISITION		dmm	w
sfrq	100.578	dmf	13889
tn	C13	dpwr	43
at	1.311	PROCESSING	
np	65536	lb	1.00
sw	25000.0	wtfile	
fb	14000	proc	ft
bs	16	fn	not used
tpwr	54	math	f
pw	5.1		
d1	1.000	werr	
tof	1966.4	wexp	
nt	11111	wbs	
ct	4720	wnt	
alock	n	DISPLAY	
gain	not used	sp	-918.2
FLAGS		wp	22959.1
il	n	vs	82
in	n	sc	0
dp	y	wc	250
hs	nn	hzmm	91.84
		is	500.00
		rfl	12842.6
		rfp	11887.0
		th	20
		ins	100.000
		nm	ph

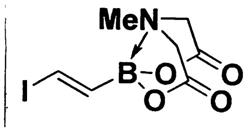

(*E*)-1

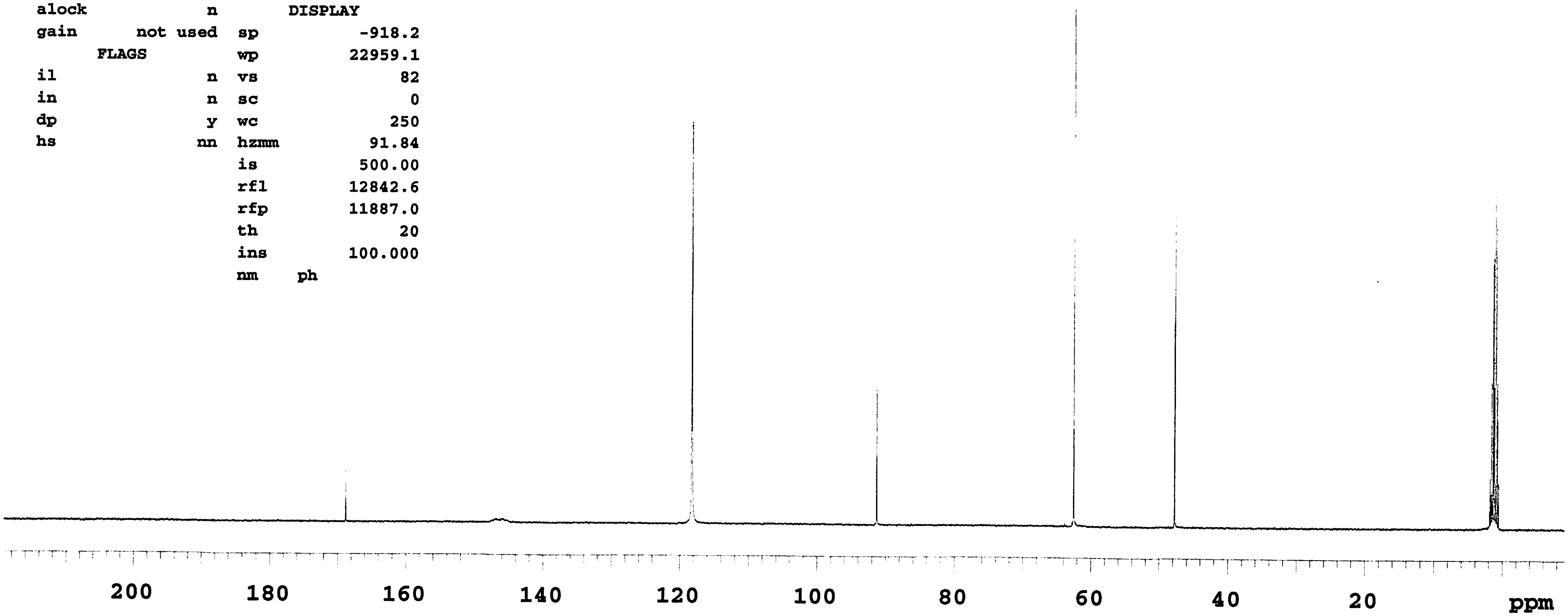

LSJV-34

exp2 std1h

SAMPLE		DEC. & VT	
date	Nov 8 2009	dn	H1
solvent	CD3CN	dof	0
file	/export/home/~	dm	nnn
data/u400/Burke/le~		dmm	c
esj/LSJV-34.fid		dmf	200
ACQUISITION		dpwr	20
sfrq	399.949	PROCESSING	
tn	H1	lb	0.30
at	4.096	wtfile	
np	65536	proc	ft
sw	8000.0	fn	not used
fb	4000	math	f
bs	16		
tpwr	58	werr	
pw	5.8	wexp	svf(n1)
d1	0	wbs	
tof	-425.7	wnt	wft('acq')
nt	64	DISPLAY	
ct	64	sp	-200.1
alock	n	wp	3999.3
gain	not used	vs	162
FLAGS		sc	0
il	n	wc	250
in	n	hzmm	16.00
dp	y	is	500.00
hs	nn	rfl	2425.7
		rfp	0
		th	20
		ins	100.000
		nm	ph

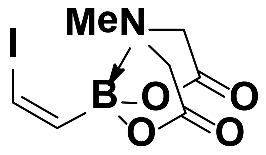

(*Z*)-1

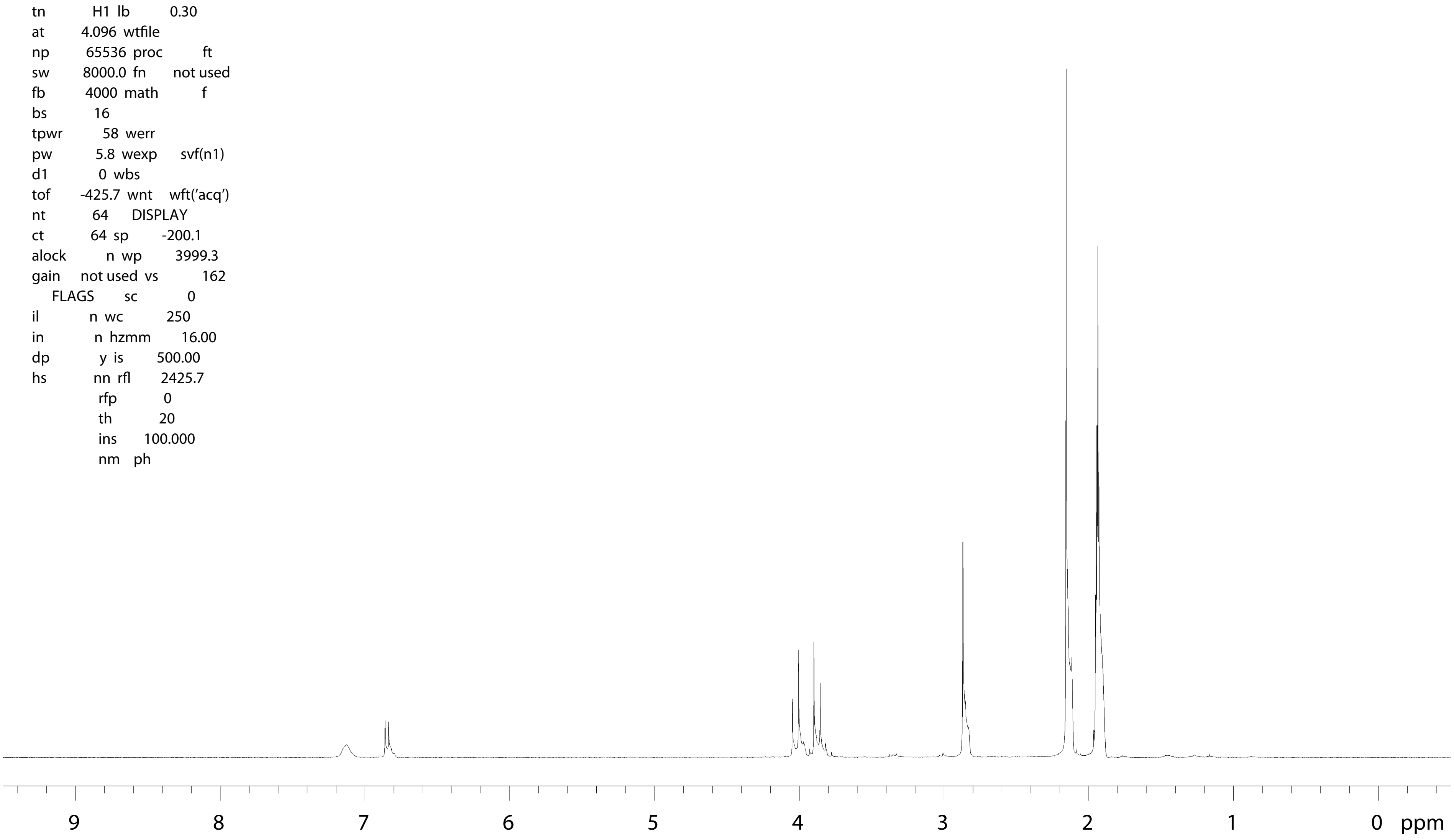

cis_IBB1

Pulse Sequence: s2pul

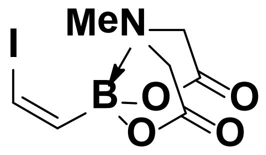

(*Z*)-1

(after 6 months storage on the bench in a vial under air in a subdued light environment)

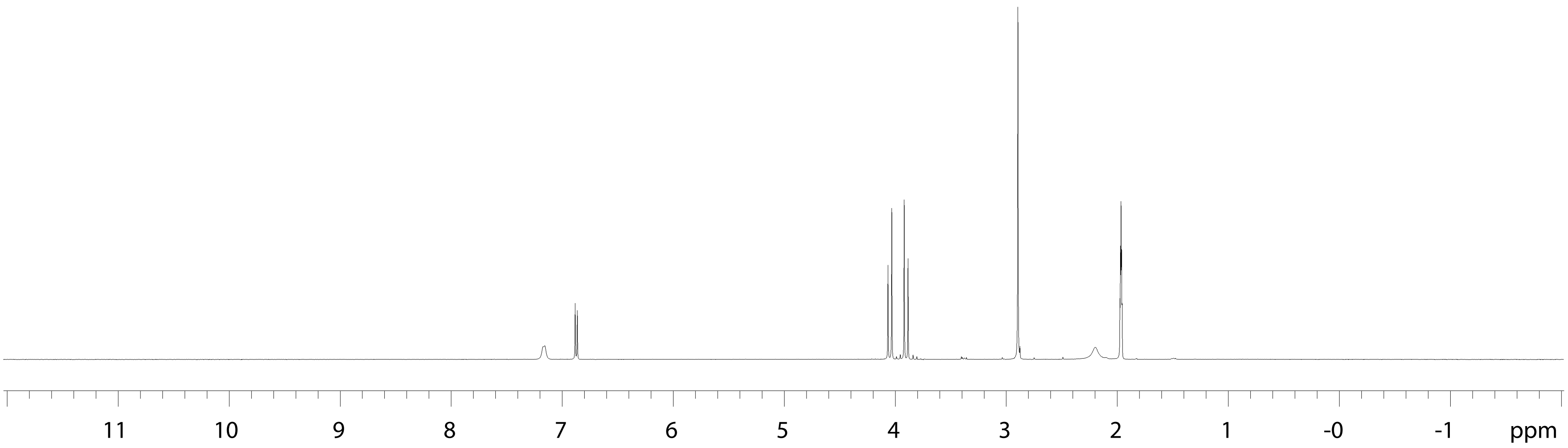

LSJV-34-13C

exp1 s2pul

SAMPLE		DEC. & VT	
date	Jun 14 2010	dfrq	499.695
solvent	CD3CN	dn	H1
file	exp	dpwr	44
ACQUISITION		dof	-827.6
sfrq	125.662	dm	yyy
tn	C13	dmm	w
at	1.086	dmf	19608
np	65536	dseq	
sw	30165.9	dres	90.0
fb	17000	homo	n
bs	16	DEC2	
ss	1	dfrq2	0
tpwr	54	dn2	
pw	6.0	dpwr2	1
d1	1.000	dof2	0
tof	1884.7	dm2	n
nt	11111	dmm2	c
ct	1054	dmf2	10000
alock	n	dseq2	
gain	not used	dres2	1.0
FLAGS		homo2	n
il	n	PROCESSING	
in	n	lb	1.00
dp	y	wtfile	
hs	nn	proc	ft
DISPLAY		fn	not used
sp	-1152.9	math	f
wp	28660.7		
vs	95	werr	
sc	0	wexp	
wc	250	wbs	
hzmm	114.64	wnt	
is	500.00		
rfl	16049.6		
rfp	14851.6		
th	5		
ins	100.000		
nm	ph		

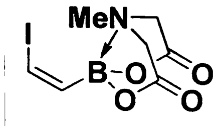

(*Z*)-1

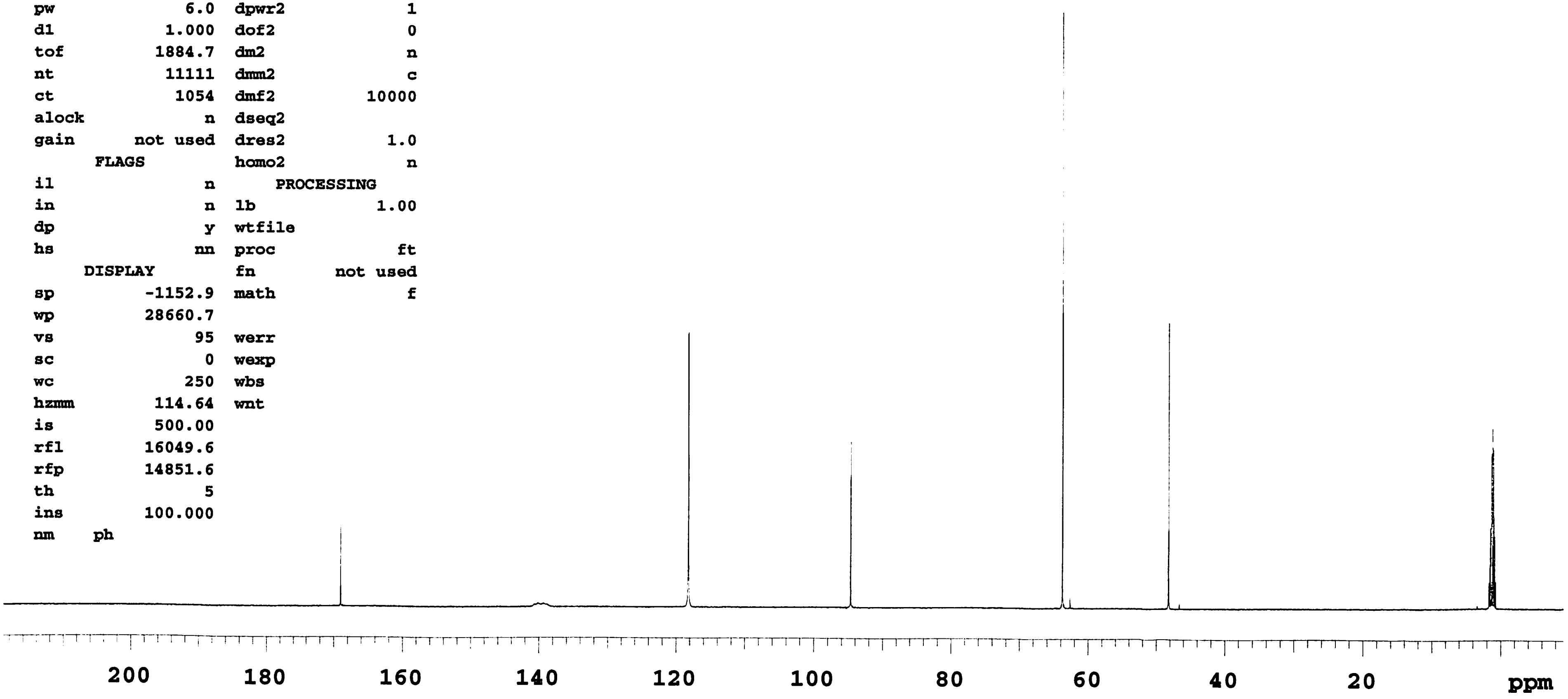

cis-Bu3SnCH=CHGeEt3

exp1 s2pul

SAMPLE		DEC. & VT	
date	Feb 28 2010	dfrq	499.693
solvent	CDCl3	dn	H1
file	exp	dpwr	20
ACQUISITION		dof	0
sfrq	499.693	dm	nnn
tn	H1	dmm	c
at	4.665	dmf	200
np	65536	dseq	
sw	7024.9	dres	1.0
fb	4000	homo	n
bs	4	DEC2	
tpwr	63	dfrq2	0
pw	6.5	dn2	
d1	0	dpwr2	1
tof	2.0	dof2	0
nt	32	dm2	n
ct	32	dmm2	c
alock	n	dmf2	200
gain	not used	dseq2	
FLAGS		dres2	1.0
il	n	homo2	n
in	n	PROCESSING	
dp	y	lb	not used
hs	nn	wtfile	
DISPLAY		proc	ft
sp	28.1	fn	not used
wp	5438.3	math	f
vs	55		
sc	0	werr	
wc	250	wexp	svf(n1)
hzmm	21.75	wbs	
is	1318.27	wnt	wft('acq')
rfl	4639.5		
rfp	3617.8		
th	3		
ins	1.000		
ai	ph		

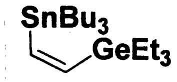

(*Z*)-2

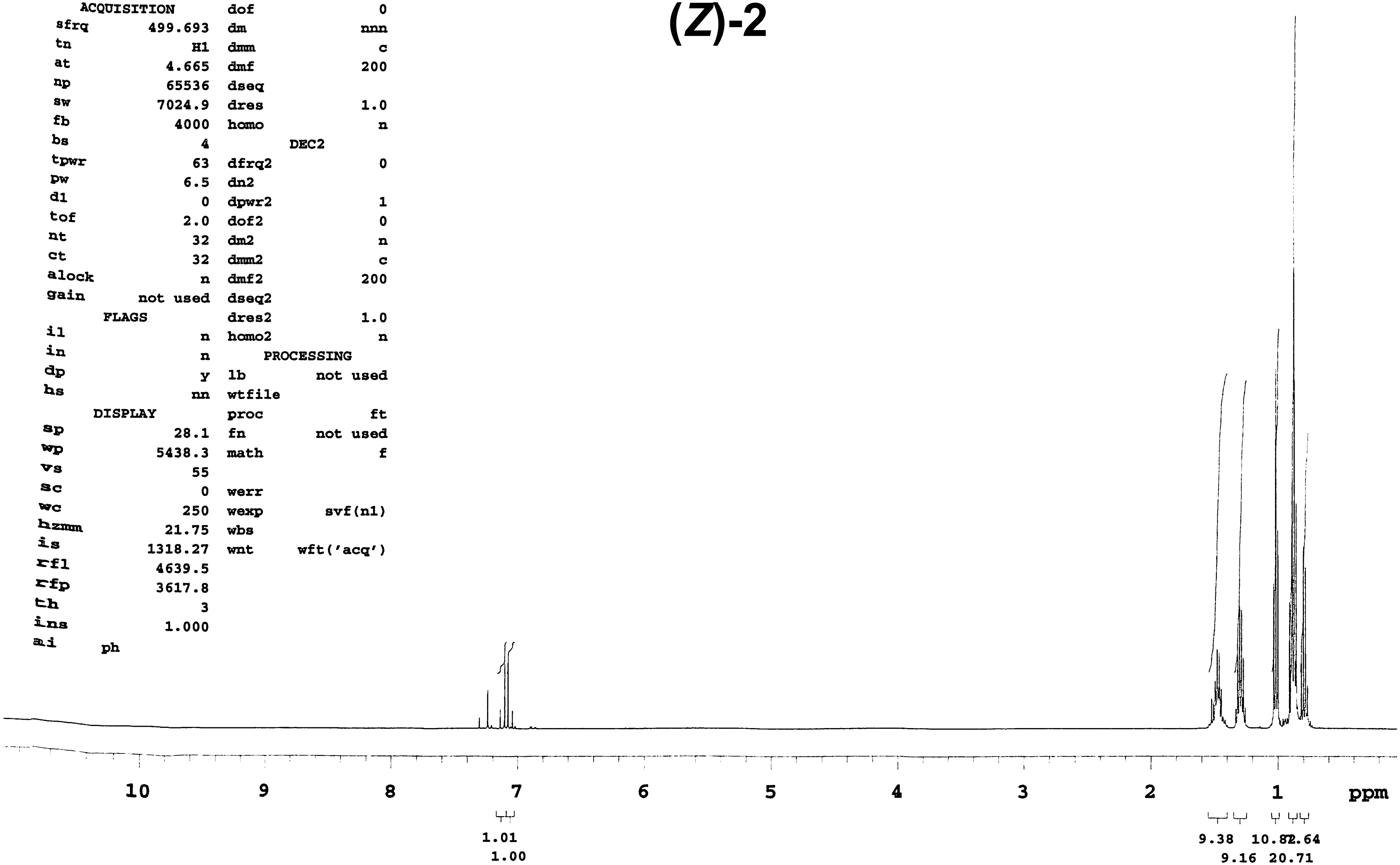

cis-Bu3SnCH=CHGeEt3

exp1 s2pul

SAMPLE		DEC. & VT	
date	Feb 28 2010	dfrq	499.692
solvent	CDCl3	dn	H1
file	exp	dpwr	44
ACQUISITION		dof	-827.6
sfrq	125.661	dm	yyy
tn	C13	dmm	w
at	1.086	dmf	19608
np	65536	dseq	
sw	30165.9	dres	90.0
fb	17000	homo	n
bs	16	DEC2	
ss	1	dfrq2	0
tpwr	54	dn2	
pw	6.0	dpwr2	1
d1	1.000	dof2	0
tof	1884.7	dm2	n
nt	5000	dmm2	c
ct	3707	dmf2	10000
alock	n	dseq2	
gain	not used	dres2	1.0
FLAGS		homo2	n
il	n	PROCESSING	
in	n	lb	1.00
dp	y	wtfile	
hs	nn	proc	ft
DISPLAY		fn	not used
sp	-1156.8	math	f
wp	28658.9		
vs	54	werr	
sc	0	wexp	
wc	250	wbs	
hzmm	114.64	wnt	
is	500.00		
rfl	10965.2		
rfp	9674.8		
th	21		
ins	100.000		
nm	ph		

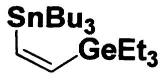

(*Z*)-2

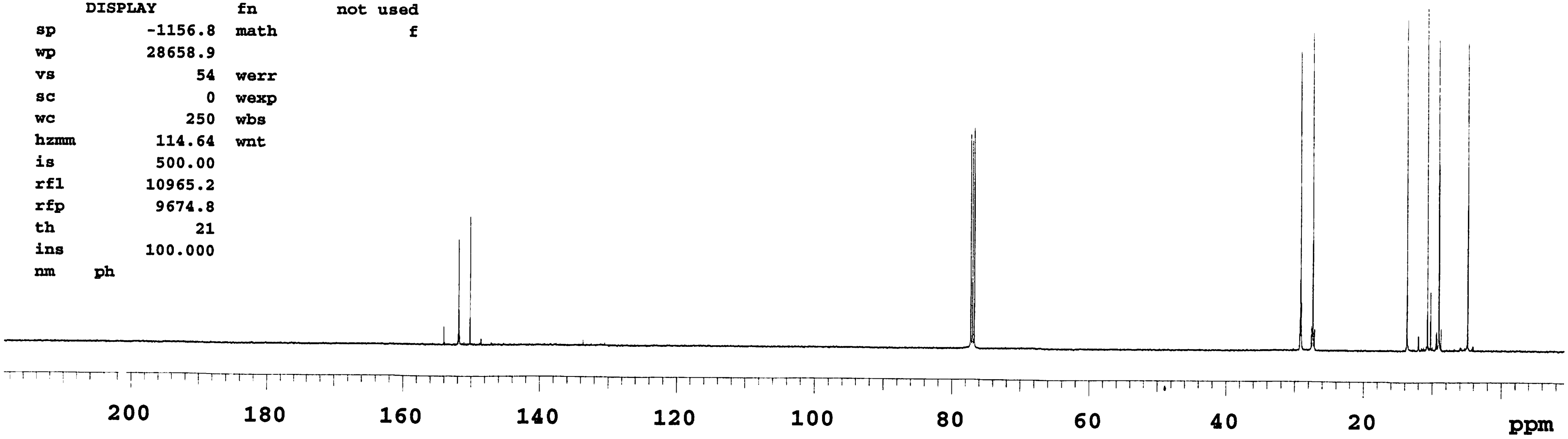

LSJV-61

exp1 s2pul

SAMPLE		DEC. & VT	
date	Jun 11 2010	dfrq	499.696
solvent	CD3CN	dn	H1
file	exp	dpwr	20
ACQUISITION		dof	0
sfrq	499.696	dm	nnn
tn	H1	dmm	c
at	4.665	dmf	200
np	65536	dseq	
sw	7024.9	dres	1.0
fb	4000	homo	n
bs	4	DEC2	
tpwr	63	dfrq2	0
pw	6.5	dn2	
d1	0	dpwr2	1
tof	2.0	dof2	0
nt	32	dm2	n
ct	32	dmm2	c
alock	n	dmf2	200
gain	not used	dseq2	
FLAGS		dres2	1.0
il	n	homo2	n
in	n	PROCESSING	
dp	y	lb	not used
hs	nn	wtfile	
DISPLAY		proc	ft
sp	16.4	fn	not used
wp	5459.9	math	f
vs	15		
sc	0	werr	
wc	250	wexp	svf(n1)
hzmm	21.84	wbs	
is	1049.33	wnt	wft('acq')
rfl	1993.8		
rfp	964.4		
th	7		
ins	1.000		
ai	ph		

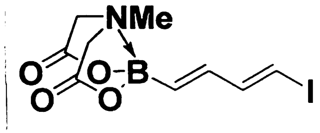

(*E*,*E*)-3

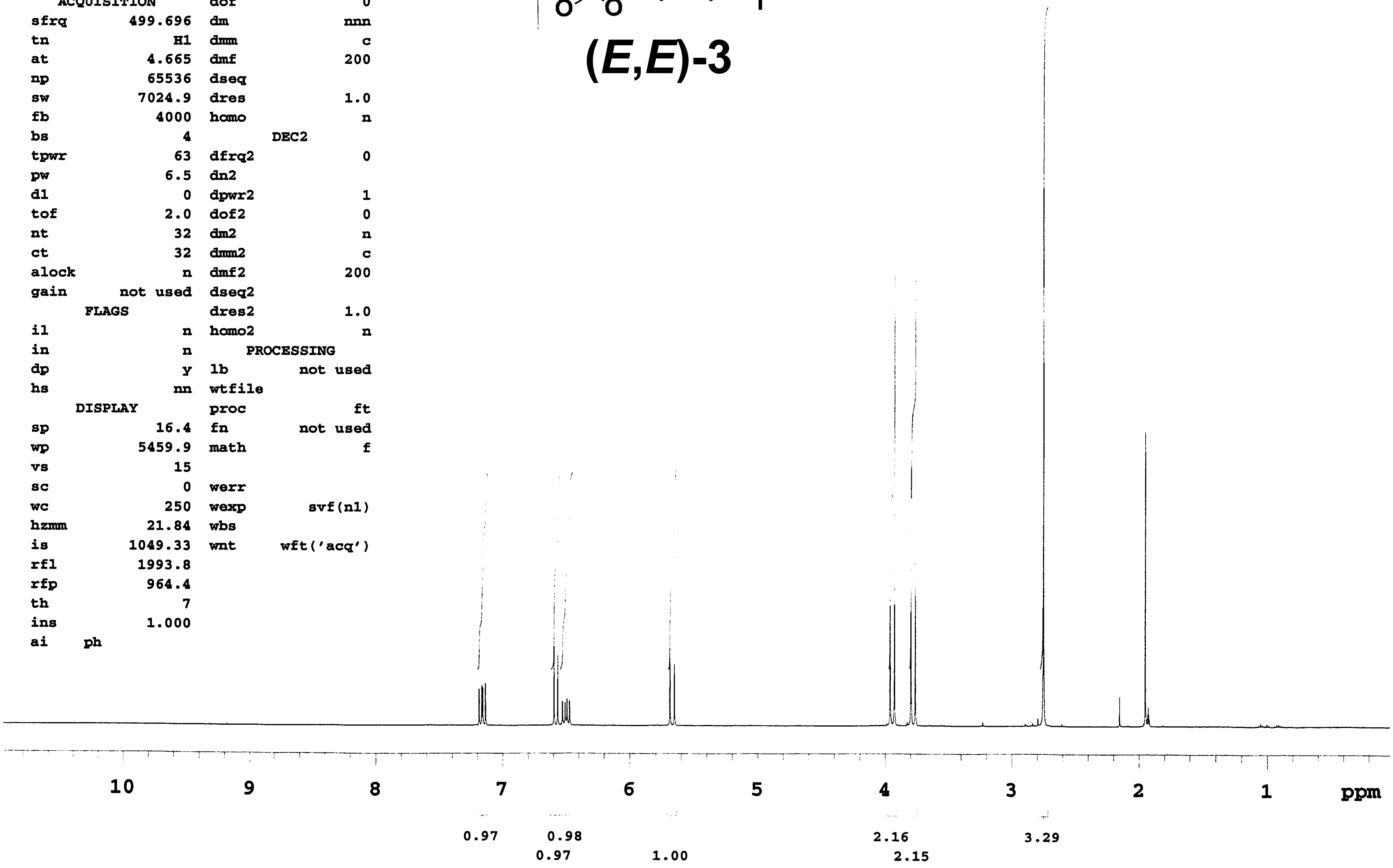

LSJV-61-13C

exp1 s2pul

SAMPLE		DEC. & VT	
date	Jun 11 2010	dfrq	499.695
solvent	CD3CN	dn	H1
file	exp	dpwr	44
ACQUISITION		dof	-827.6
sfrq	125.662	dm	yyy
tn	C13	dmm	w
at	1.086	dmf	19608
np	65536	dseq	
sw	30165.9	dres	90.0
fb	17000	homo	n
bs	16	DEC2	
ss	1	dfrq2	0
tpwr	54	dn2	
pw	6.0	dpwr2	1
d1	1.000	dof2	0
tof	1884.7	dm2	n
nt	11111	dmm2	c
ct	1355	dmf2	10000
alock	n	dseq2	
gain	not used	dres2	1.0
FLAGS		homo2	n
il	n	PROCESSING	
in	n	lb	1.00
dp	y	wtfile	
hs	nn	proc	ft
DISPLAY		fn	not used
sp	-1144.6	math	f
wp	28739.0		
vs	90	werr	
sc	0	wexp	
wc	250	wbs	
hzmm	114.96	wnt	
is	500.00		
rfl	16041.3		
rfp	14851.6		
th	68		
ins	100.000		
nm	ph		

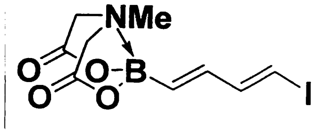

(*E*,*E*)-3

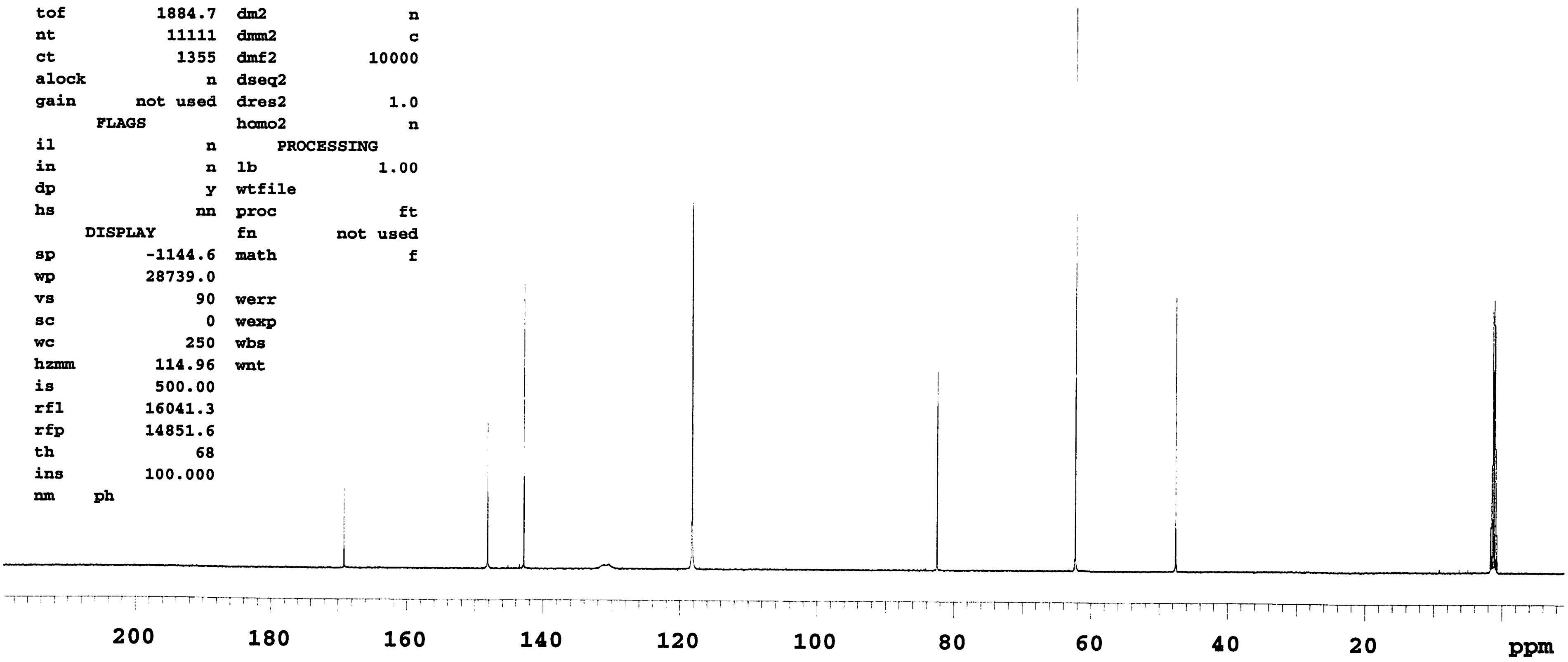

LSJV-65

exp1 s2pul

SAMPLE		DEC. & VT	
date	Jun 11 2010	dfrq	499.696
solvent	CD3CN	dn	H1
file	exp	dpwr	20
ACQUISITION		dof	0
sfrq	499.696	dm	nnn
tn	H1	dmm	c
at	4.665	dmf	200
np	65536	dseq	
sw	7024.9	dres	1.0
fb	4000	homo	n
bs	4	DEC2	
tpwr	63	dfrq2	0
pw	6.5	dn2	
d1	0	dpwr2	1
tof	2.0	dof2	0
nt	32	dm2	n
ct	32	dmm2	c
alock	n	dmf2	200
gain	not used	dseq2	
FLAGS		dres2	1.0
il	n	homo2	n
in	n	PROCESSING	
dp	y	lb	not used
hs	nn	wtfile	
DISPLAY		proc	ft
sp	37.0	fn	not used
wp	5418.6	math	f
vs	22		
sc	0	werr	
wc	250	wexp	svf(n1)
hzmm	21.67	wbs	
is	1474.97	wnt	wft('acq')
rfl	1993.8		
rfp	964.4		
th	7		
ins	1.000		
ai	ph		

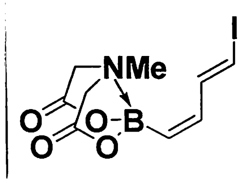

(*E*,*Z*)-3

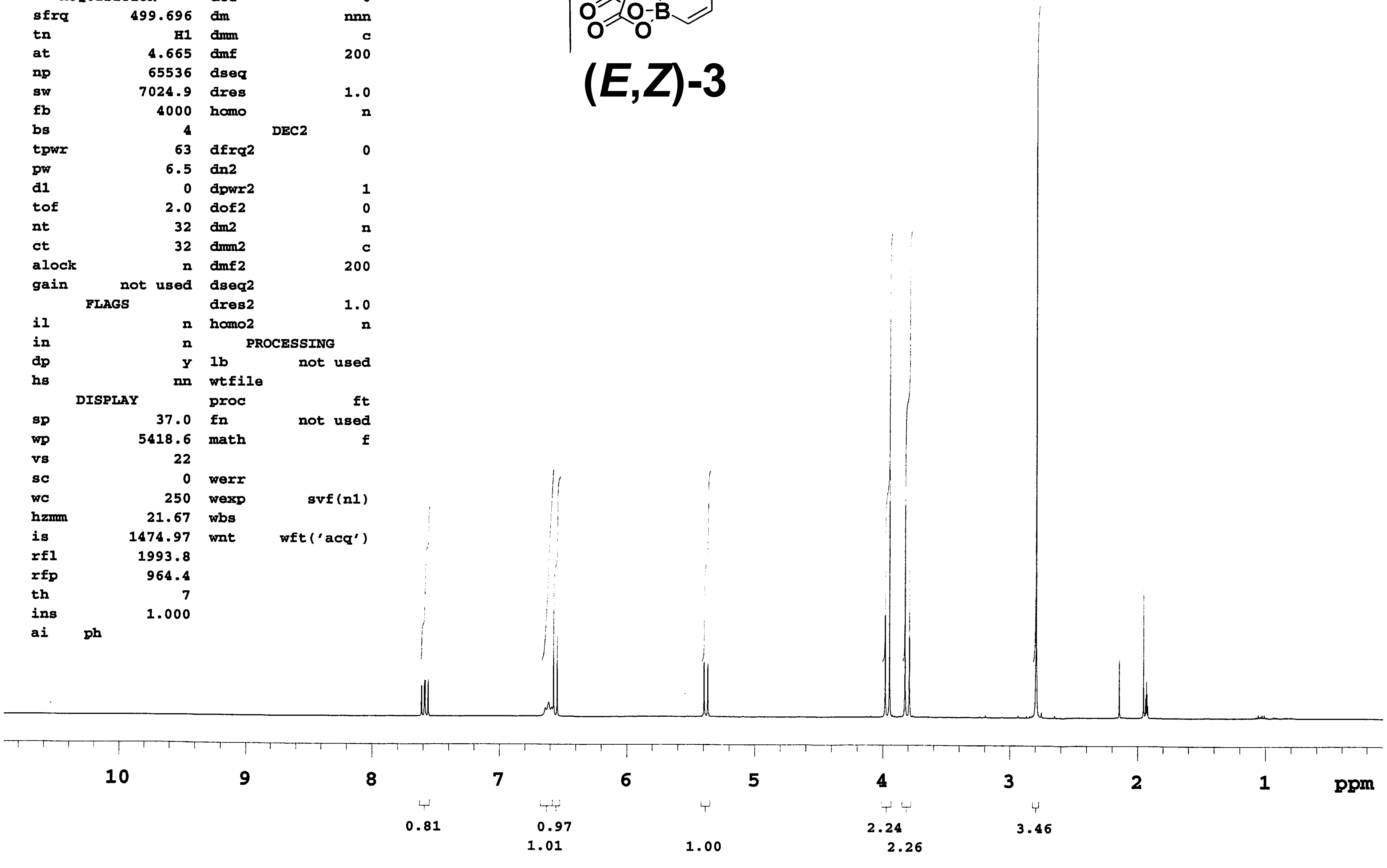

LSJV-65-13C

exp1 s2pul

SAMPLE		DEC. & VT	
date	Jun 11 2010	dfrq	499.695
solvent	CD3CN	dn	H1
file	exp	dpwr	44
ACQUISITION		dof	-827.6
sfrq	125.662	dm	yyy
tn	C13	dmm	w
at	1.086	dmf	19608
np	65536	dseq	
sw	30165.9	dres	90.0
fb	17000	homo	n
bs	16	DEC2	
ss	1	dfrq2	0
tpwr	54	dn2	
pw	6.0	dpwr2	1
d1	1.000	dof2	0
tof	1884.7	dm2	n
nt	11111	dmm2	c
ct	1581	dmf2	10000
alock	n	dseq2	
gain	not used	dres2	1.0
FLAGS		homo2	n
il	n	PROCESSING	
in	n	lb	1.00
dp	y	wtfile	
hs	nn	proc	ft
DISPLAY		fn	not used
sp	-1140.9	math	f
wp	28649.7		
vs	84	werr	
sc	0	wexp	
wc	250	wbs	
hzmm	114.60	wnt	
is	500.00		
rfl	16037.6		
rfp	14851.6		
th	68		
ins	100.000		
nm	ph		

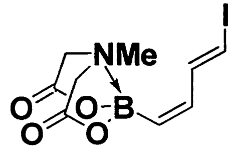

(*E*,*Z*)-3

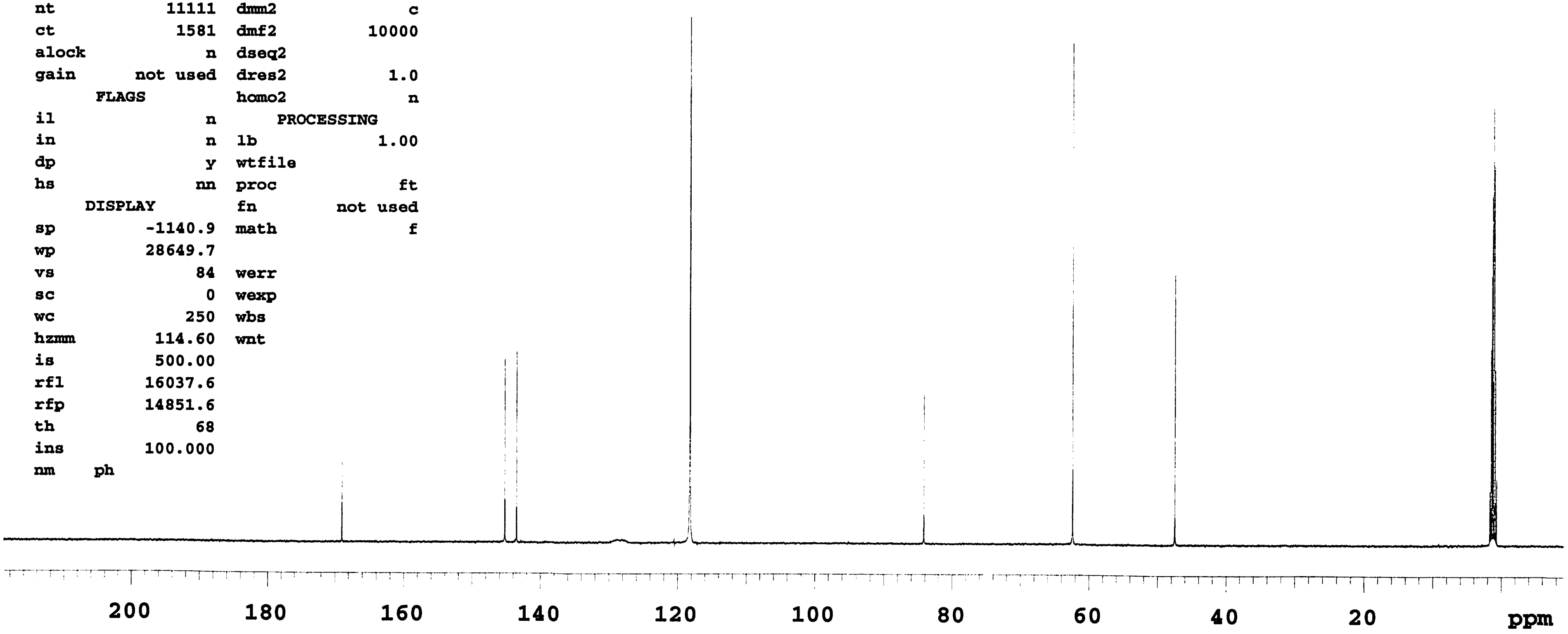

LSJV-69

exp1 s2pul

SAMPLE		DEC. & VT	
date	Jun 12 2010	dfrq	499.696
solvent	CD3CN	dn	H1
file	exp	dpwr	20
ACQUISITION		dof	0
sfrq	499.696	dm	nnn
tn	H1	dmm	c
at	4.665	dmf	200
np	65536	dseq	
sw	7024.9	dres	1.0
fb	4000	homo	n
bs	4	DEC2	
tpwr	63	dfrq2	0
pw	6.5	dn2	
d1	0	dpwr2	1
tof	2.0	dof2	0
nt	32	dm2	n
ct	32	dmm2	c
alock	n	dmf2	200
gain	not used	dseq2	
FLAGS		dres2	1.0
il	n	homo2	n
in	n	PROCESSING	
dp	y	lb	not used
hs	nn	wtfile	
DISPLAY		proc	ft
sp	47.5	fn	not used
wp	5418.6	math	f
vs	19		
sc	0	werr	
wc	250	wexp	svf(n1)
hzmm	21.67	wbs	
is	1109.34	wnt	wft('acq')
rfl	1993.8		
rfp	964.4		
th	2		
ins	1.000		
ai	ph		

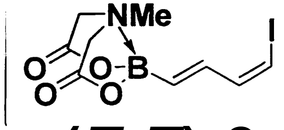

(*Z*,*E*)-3

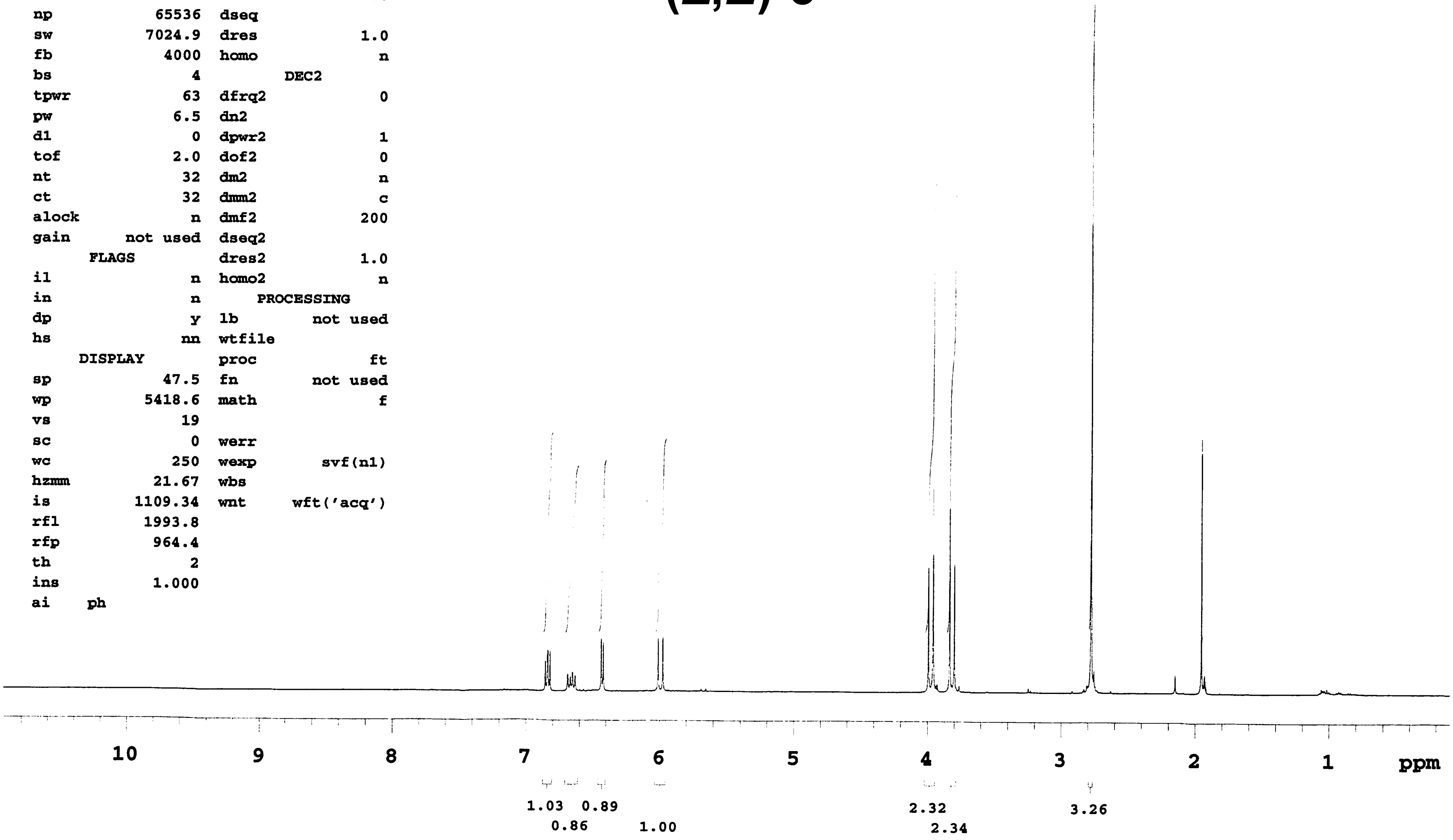

LSJV-69-13C

exp1 s2pul

SAMPLE		DEC. & VT	
date	Jun 12 2010	dfrq	499.695
solvent	CD3CN	dn	H1
file	exp	dpwr	44
ACQUISITION		dof	-827.6
sfrq	125.662	dm	yyy
tn	C13	dmm	w
at	1.086	dmf	19608
np	65536	dseq	
sw	30165.9	dres	90.0
fb	17000	homo	n
bs	16	DEC2	
ss	1	dfrq2	0
tpwr	54	dn2	
pw	6.0	dpwr2	1
d1	1.000	dof2	0
tof	1884.7	dm2	n
nt	11111	dmm2	c
ct	1554	dmf2	10000
alock	n	dseq2	
gain	not used	dres2	1.0
FLAGS		homo2	n
il	n	PROCESSING	
in	n	lb	1.00
dp	y	wtfile	
hs	nn	proc	ft
DISPLAY		fn	not used
sp	-1098.6	math	f
wp	28649.7		
vs	85	werr	
sc	0	wexp	
wc	250	wbs	
hzmm	114.60	wnt	
is	500.00		
rfl	16039.4		
rfp	14851.6		
th	68		
ins	100.000		
nm	ph		

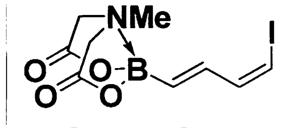

(*Z*,*E*)-3

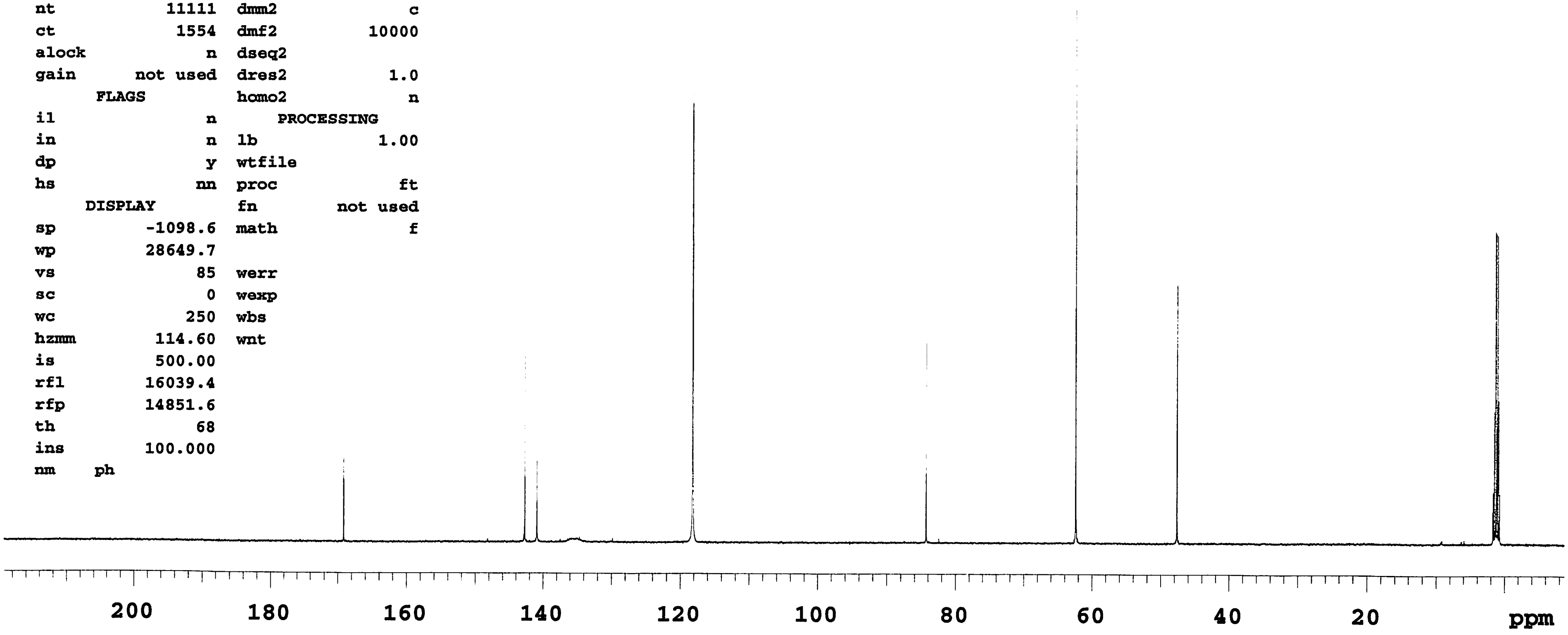

LSJV-75

exp1 std1h

SAMPLE		DEC. & VT	
date	Apr 4 2010	dn	H1
solvent	CD3CN	dof	0
file	exp	dm	nnn
ACQUISITION		dmm	c
sfrq	399.949	dmf	200
tn	H1	dpwr	20
at	4.096	PROCESSING	
np	65536	lb	not used
sw	8000.0	wtfile	
fb	4000	proc	ft
bs	16	fn	not used
tpwr	58	math	f
pw	5.8		
d1	0	werr	
tof	-425.7	wexp	svf(n1)
nt	32	wbs	
ct	32	wnt	wft('acq')
alock	n	DISPLAY	
gain	not used	sp	19.0
FLAGS		wp	4358.9
il	n	vs	71
in	n	sc	0
dp	y	wc	250
hs	nn	hzmm	17.44
		is	1183.89
		rfl	3201.9
		rfp	771.9
		th	1
		ins	1.000
		nm	ph

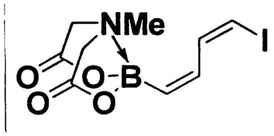

(*Z*,*Z*)-3

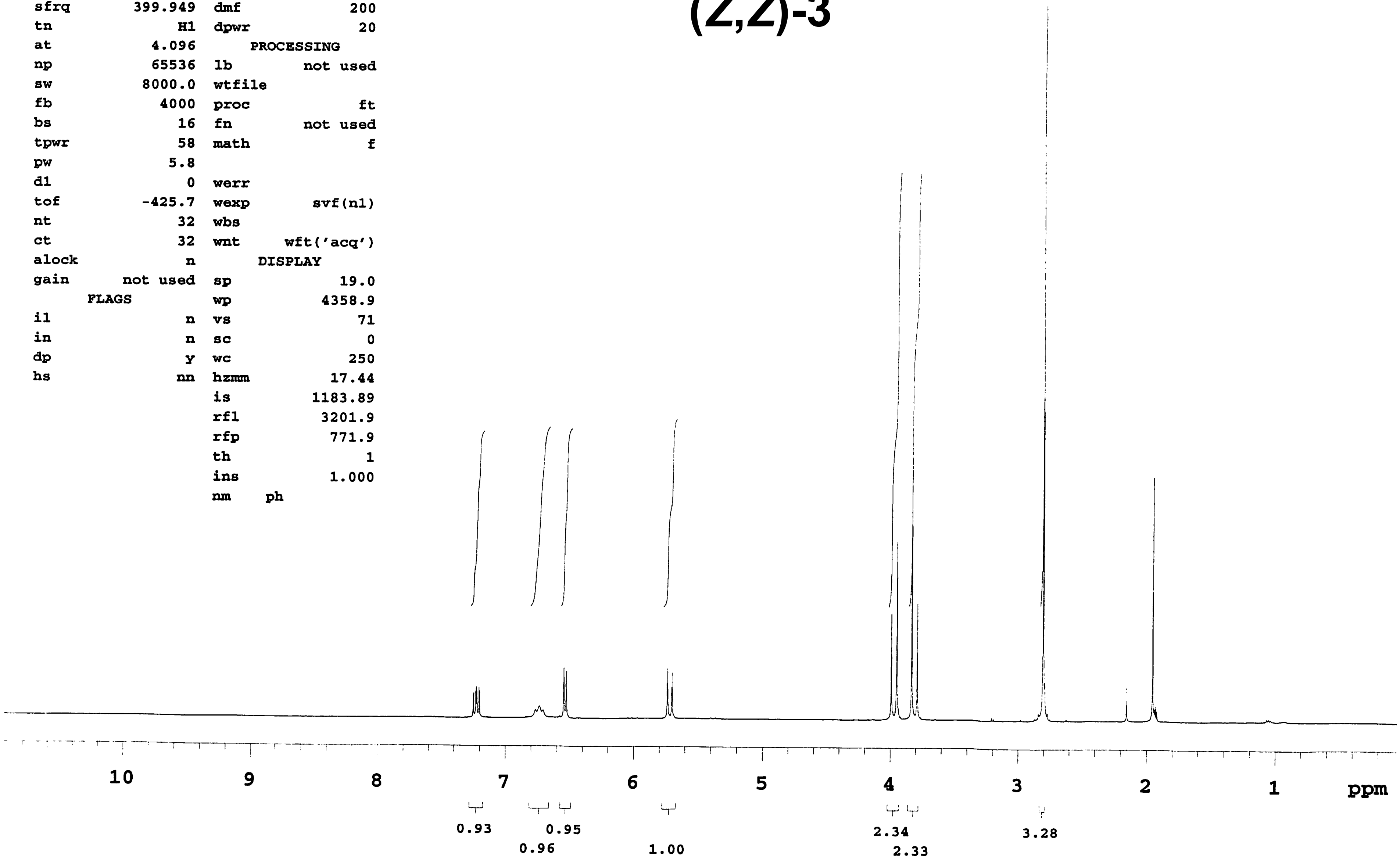

LSJV-75-13C

exp1 std13c

SAMPLE		DEC. & VT	
date	Apr 4 2010	dn	H1
solvent	CD3CN	dof	-1092.3
file	exp	dm	yyy
ACQUISITION		dmm	w
sfrq	100.578	dmf	13889
tn	C13	dpwr	43
at	1.311	PROCESSING	
np	65536	lb	1.00
sw	25000.0	wtfile	
fb	14000	proc	ft
bs	16	fn	not used
tpwr	54	math	f
pw	5.1		
d1	1.000	werr	
tof	1966.4	wexp	
nt	11111	wbs	
ct	2022	wnt	
alock	n	DISPLAY	
gain	not used	sp	-851.8
FLAGS		wp	22800.4
il	n	vs	76
in	n	sc	0
dp	y	wc	250
hs	nn	hzmm	91.20
		is	500.00
		rfl	12843.3
		rfp	11887.0
		th	20
		ins	100.000
		nm	ph

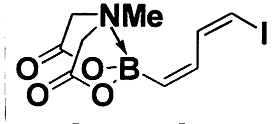

(*Z*,*Z*)-3

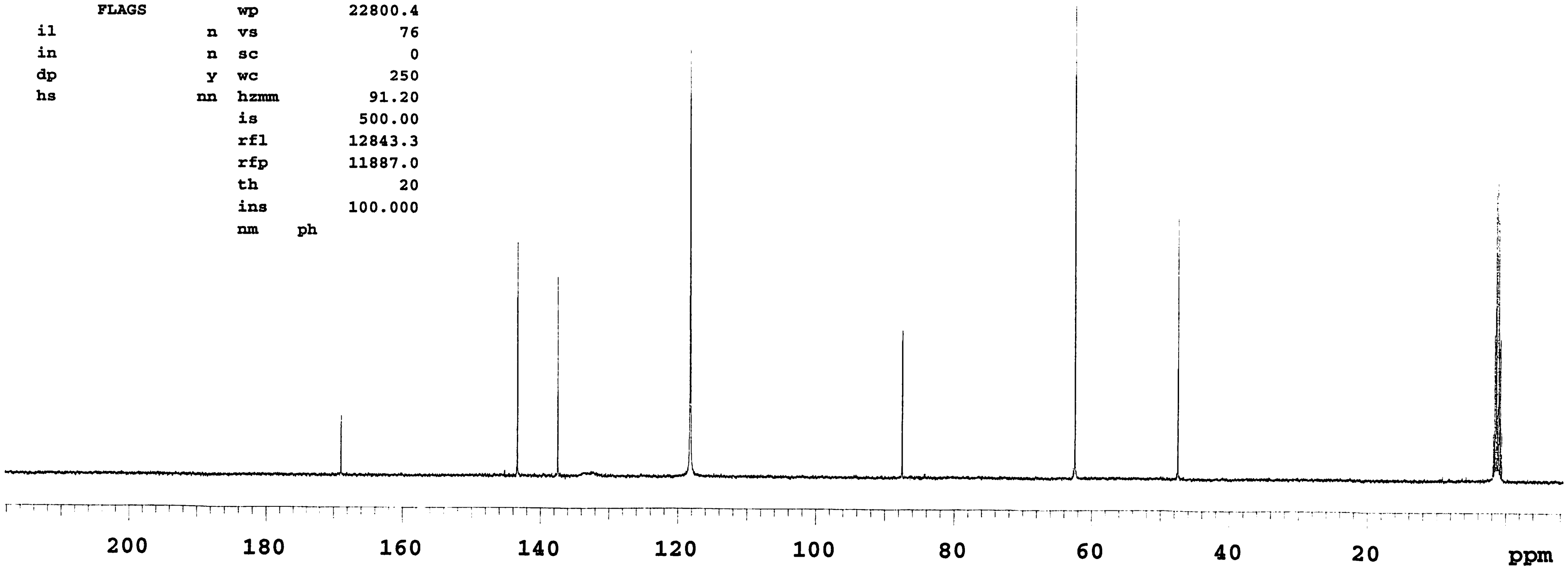

LSJIV-18

exp1 std1h

SAMPLE		DEC. & VT	
date	Jan 12 2009	dn	H1
solvent	CD3CN	dof	0
file	exp	dm	nnn
ACQUISITION		dmm	c
sfrq	399.949	dmf	200
tn	H1	dpwr	20
at	4.096	PROCESSING	
np	65536	lb	not used
sw	8000.0	wtfile	
fb	4000	proc	ft
bs	16	fn	not used
tpwr	58	math	f
pw	5.8		
d1	0	werr	
tof	-425.7	wexp	svf(n1)
nt	16	wbs	
ct	16	wnt	wft('acq')
alock	n	DISPLAY	
gain	not used	sp	12.8
FLAGS		wp	4377.2
il	n	vs	70
in	n	sc	0
dp	y	wc	250
hs	nn	hzmm	17.51
		is	1569.59
		rfl	2425.7
		rfp	0
		th	20
		ins	1.000
		nm	ph

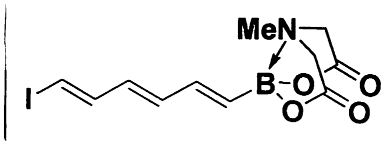

(*E*,*E*,*E*)-4

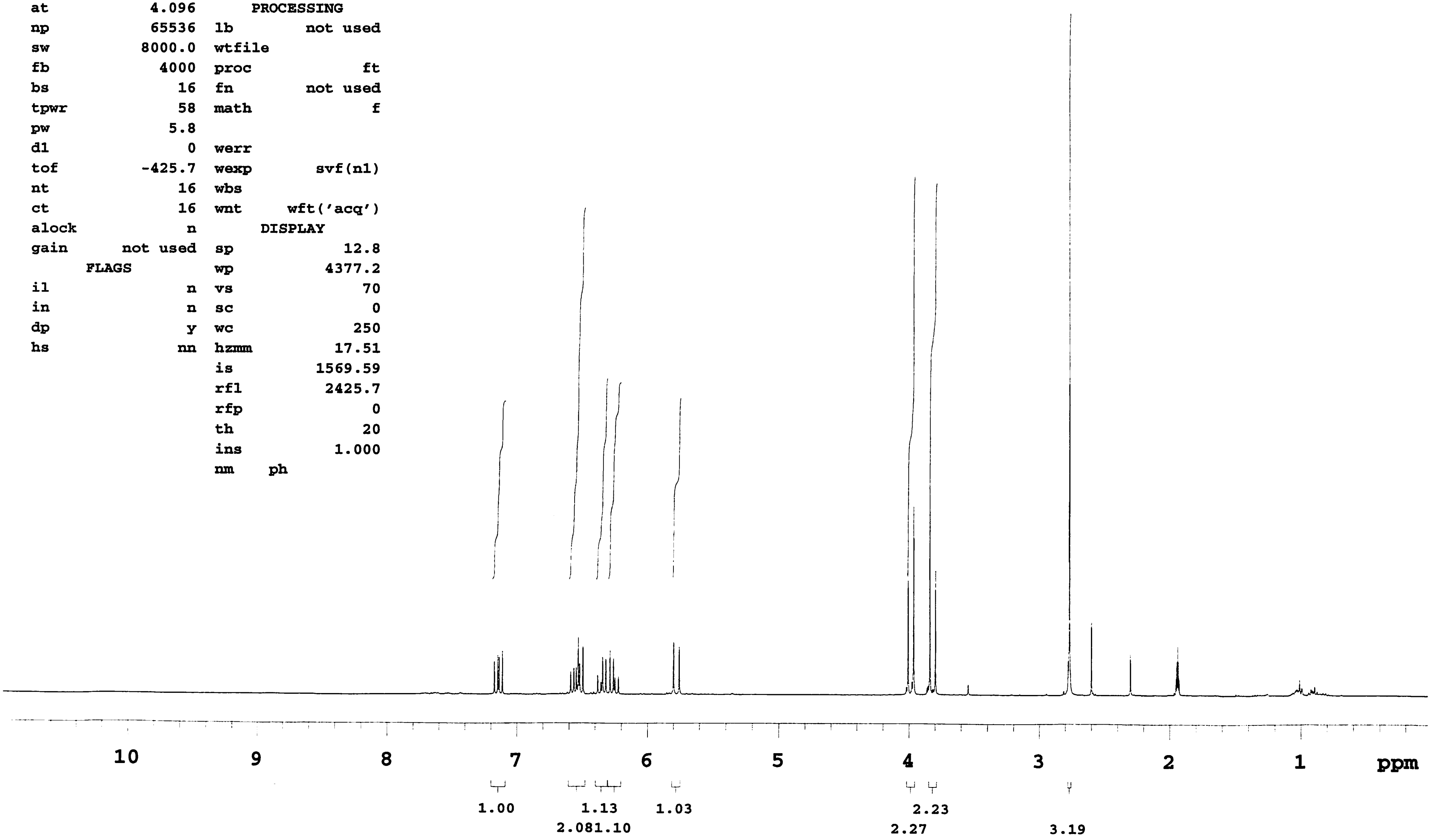

LSJIV-36-13C

exp1 s2pul

SAMPLE		DEC. & VT	
date	Jun 14 2010	dfrq	499.695
solvent	CD3CN	dn	H1
file	exp	dpwr	44
ACQUISITION		dof	-827.6
sfrq	125.662	dm	yyy
tn	C13	dmm	w
at	1.086	dmf	19608
np	65536	dseq	
sw	30165.9	dres	90.0
fb	17000	homo	n
bs	16	DEC2	
ss	1	dfrq2	0
tpwr	54	dn2	
pw	6.0	dpwr2	1
d1	1.000	dof2	0
tof	1884.7	dm2	n
nt	11111	dmm2	c
ct	441	dmf2	10000
alock	n	dseq2	
gain	not used	dres2	1.0
FLAGS		homo2	n
il	n	PROCESSING	
in	n	lb	1.00
dp	y	wtfile	
hs	nn	proc	ft
DISPLAY		fn	not used
sp	-1096.7	math	f
wp	28660.7		
vs	74	werr	
sc	0	wexp	
wc	250	wbs	
hzmm	114.64	wnt	
is	500.00		
rfl	16037.6		
rfp	14851.6		
th	68		
ins	100.000		
nm	ph		

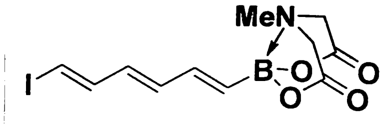

(*E*,*E*,*E*)-4

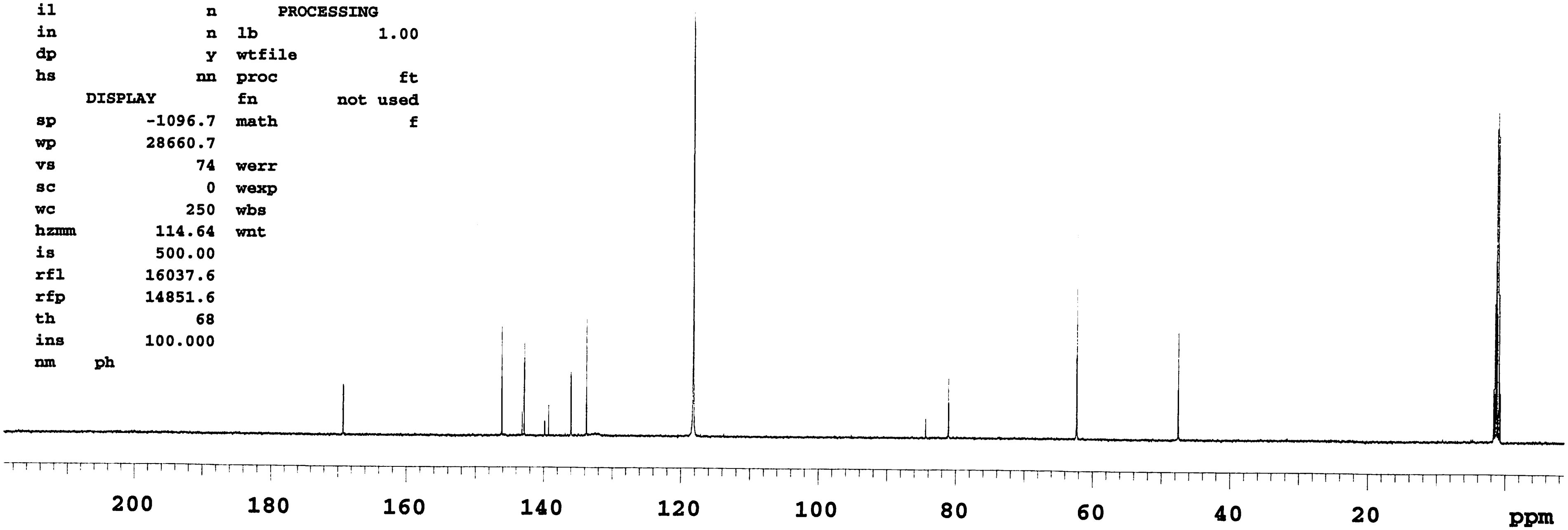

LSJV-60

exp1 s2pul

SAMPLE		DEC. & VT	
date	Jun 13 2010	dfrq	499.696
solvent	CD3CN	dn	H1
file	exp	dpwr	20
ACQUISITION		dof	0
sfrq	499.696	dm	nnn
tn	H1	dmm	c
at	4.665	dmf	200
np	65536	dseq	
sw	7024.9	dres	1.0
fb	4000	homo	n
bs	4	DEC2	
tpwr	63	dfrq2	0
pw	6.5	dn2	
d1	0	dpwr2	1
tof	2.0	dof2	0
nt	32	dm2	n
ct	32	dmm2	c
alock	n	dmf2	200
gain	not used	dseq2	
FLAGS		dres2	1.0
il	n	homo2	n
in	n	PROCESSING	
dp	y	lb	not used
hs	nn	wtfile	
DISPLAY		proc	ft
sp	8.7	fn	not used
wp	5450.9	math	f
vs	35		
sc	0	werr	
wc	250	wexp	svf(n1)
hzmm	21.80	wbs	
is	1280.96	wnt	wft('acq')
rfl	1993.8		
rfp	964.4		
th	7		
ins	3.000		
ai	ph		

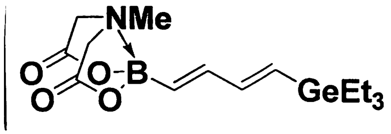

(*E*,*E*)-8

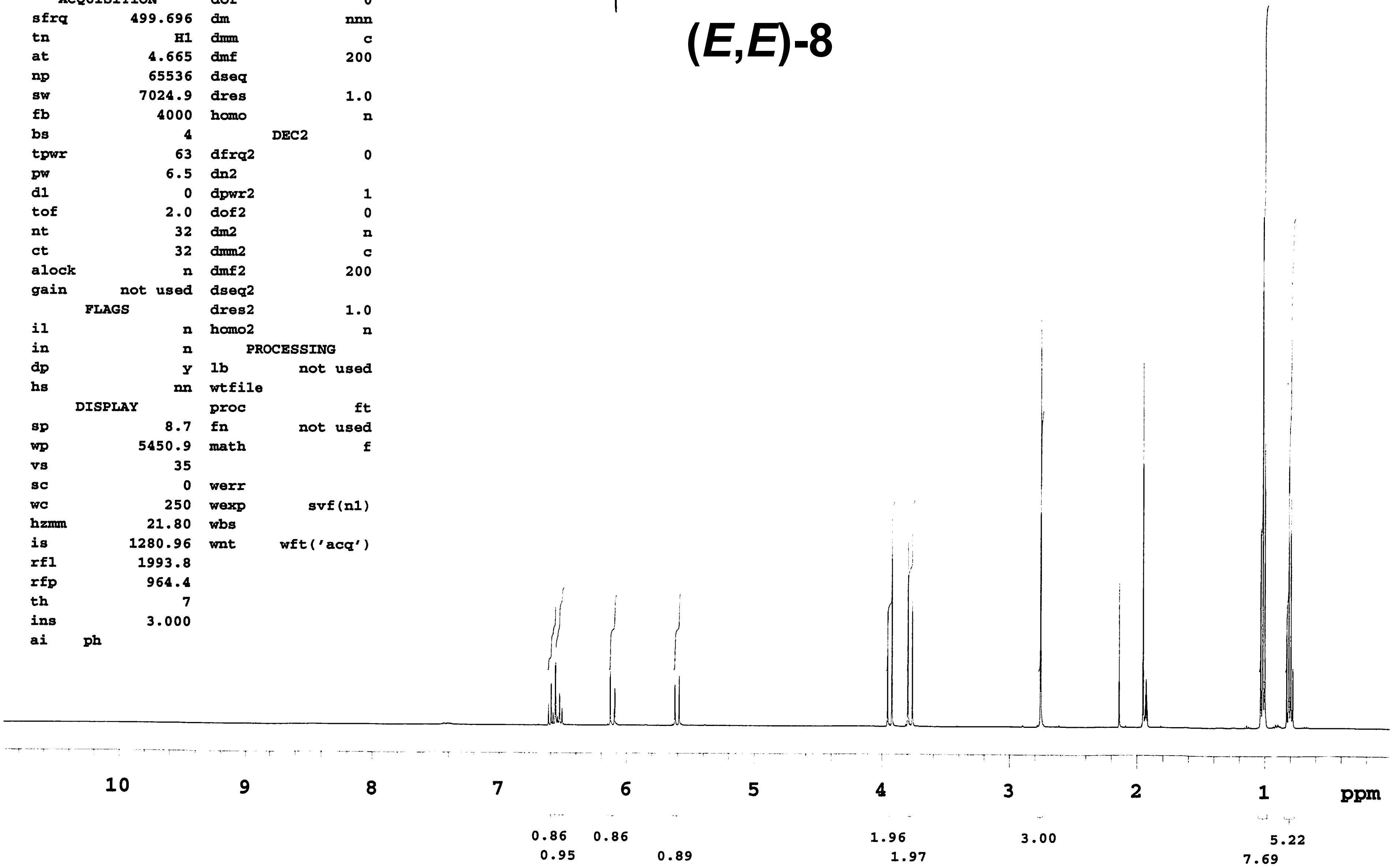

LSJV-60-13C

exp1 s2pul

SAMPLE		DEC. & VT	
date	Jun 13 2010	dfrq	499.695
solvent	CD3CN	dn	H1
file	exp	dpwr	44
ACQUISITION		dof	-827.6
sfrq	125.662	dm	yyy
tn	C13	dmm	w
at	1.086	dmf	19608
np	65536	dseq	
sw	30165.9	dres	90.0
fb	17000	homo	n
bs	16	DEC2	
ss	1	dfrq2	0
tpwr	54	dn2	
pw	6.0	dpwr2	1
d1	1.000	dof2	0
tof	1884.7	dm2	n
nt	11111	dmm2	c
ct	1955	dmf2	10000
alock	n	dseq2	
gain	not used	dres2	1.0
FLAGS		homo2	n
il	n	PROCESSING	
in	n	lb	1.00
dp	y	wtfile	
hs	nn	proc	ft
DISPLAY		fn	not used
sp	-1094.9	math	f
wp	28574.2		
vs	98	werr	
sc	0	wexp	
wc	250	wbs	
hzmm	114.30	wnt	
is	500.00		
rfl	16033.0		
rfp	14851.6		
th	68		
ins	100.000		
nm	ph		

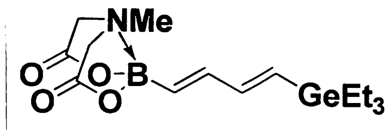

(*E*,*E*)-8

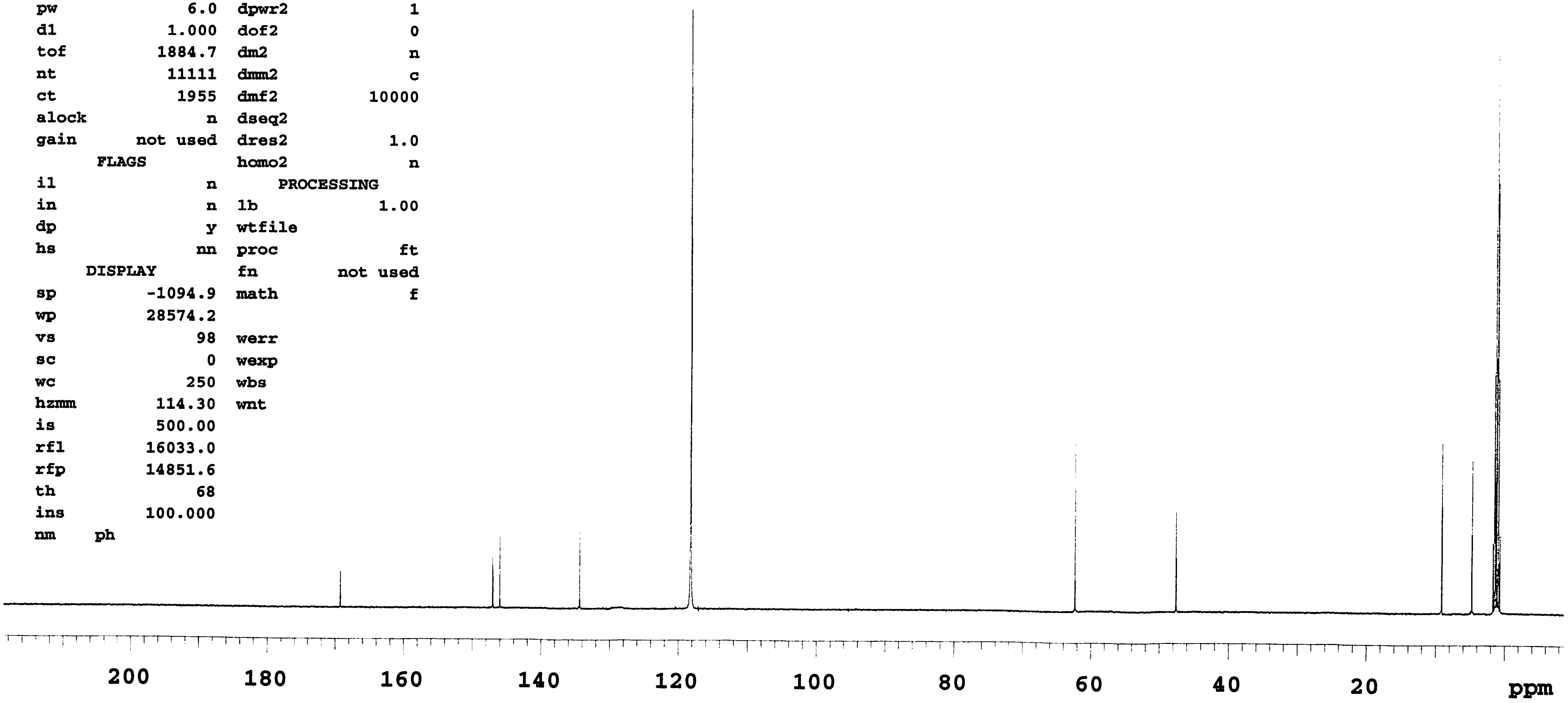

LSJV-64

exp1 s2pul

SAMPLE		DEC. & VT	
date	Jun 13 2010	dfrq	499.696
solvent	CD3CN	dn	H1
file	exp	dpwr	20
ACQUISITION		dof	0
sfrq	499.696	dm	nnn
tn	H1	dmm	c
at	4.665	dmf	200
np	65536	dseq	
sw	7024.9	dres	1.0
fb	4000	homo	n
bs	4	DEC2	
tpwr	63	dfrq2	0
pw	6.5	dn2	
d1	0	dpwr2	1
tof	2.0	dof2	0
nt	32	dm2	n
ct	32	dmm2	c
alock	n	dmf2	200
gain	not used	dseq2	
FLAGS		dres2	1.0
il	n	homo2	n
in	n	PROCESSING	
dp	y	lb	not used
hs	nn	wtfile	
DISPLAY		proc	ft
sp	8.7	fn	not used
wp	5471.5	math	f
vs	17		
sc	0	werr	
wc	250	wexp	svf(n1)
hzmm	21.89	wbs	
is	1216.02	wnt	wft('acq')
rfl	1993.8		
rfp	964.4		
th	7		
ins	3.000		
ai	ph		

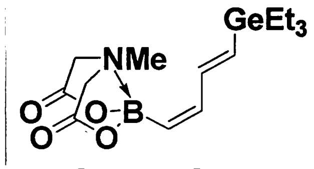

(E,Z)-8

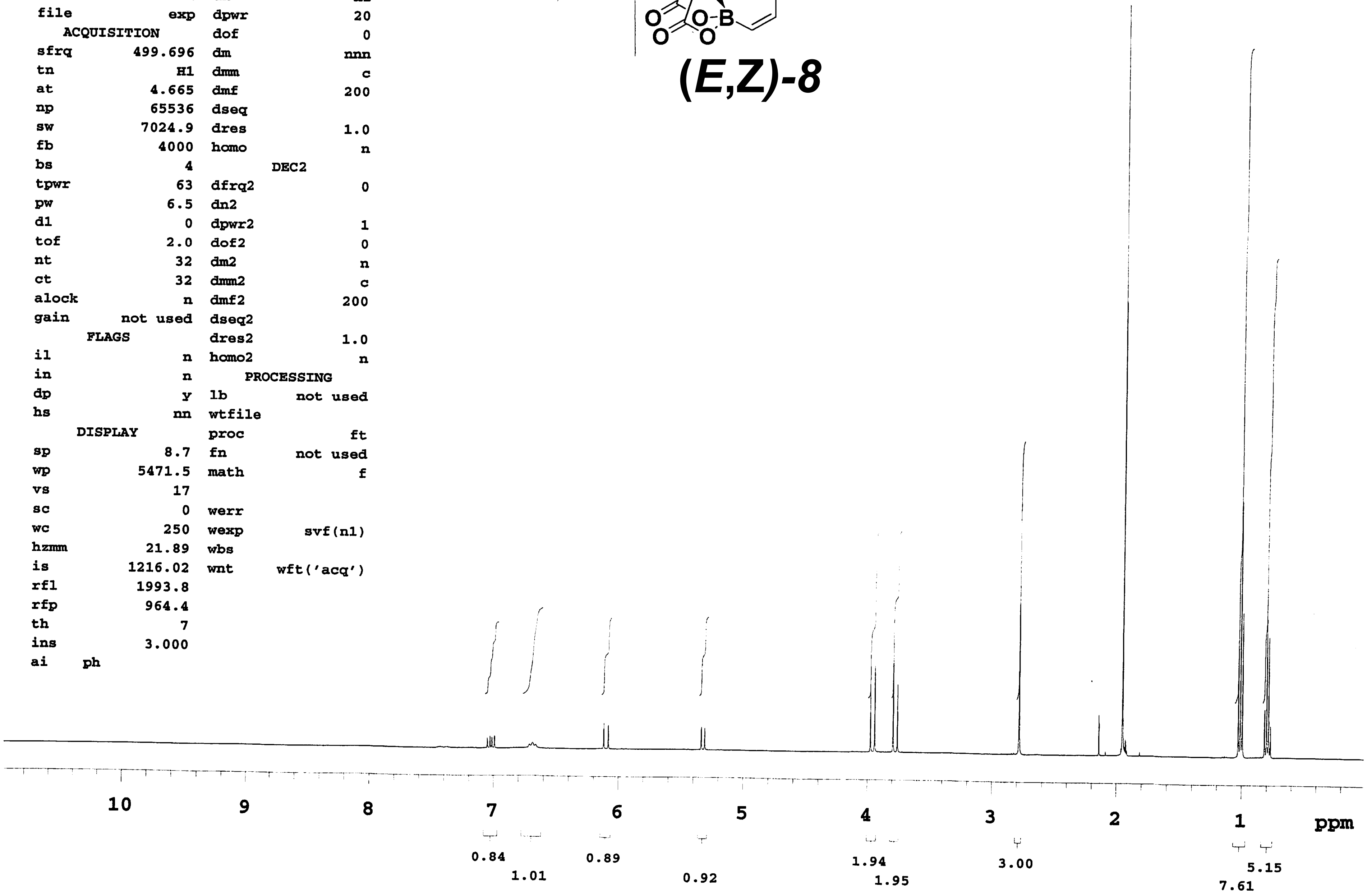

LSJV-64-13C

exp1 s2pul

```
      SAMPLE                 DEC. & VT
date   Jun 13 2010    dfrq        499.695
solvent      CD3CN    dn               H1
file           exp    dpwr             44
   ACQUISITION        dof          -827.6
sfrq       125.662    dm              yyy
tn             C13    dmm               w
at           1.086    dmf           19608
np           65536    dseq
sw         30165.9    dres           90.0
fb           17000    homo              n
bs              16          DEC2
ss               1    dfrq2             0
tpwr            54    dn2
pw             6.0    dpwr2             1
d1           1.000    dof2              0
tof         1884.7    dm2               n
nt           11111    dmm2              c
ct            1957    dmf2          10000
alock            n    dseq2
gain      not used    dres2           1.0
      FLAGS           homo2             n
il               n       PROCESSING
in               n    lb             1.00
dp               y    wtfile
hs              nn    proc             ft
     DISPLAY          fn         not used
sp         -1094.9    math              f
wp         28662.6
vs              81    werr
sc               0    wexp
wc             250    wbs
hzmm        114.65    wnt
is          500.00
rfl        16033.0
rfp        14851.6
th              68
ins        100.000
nm    ph
```

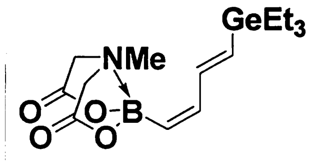

(*E*,*Z*)-8

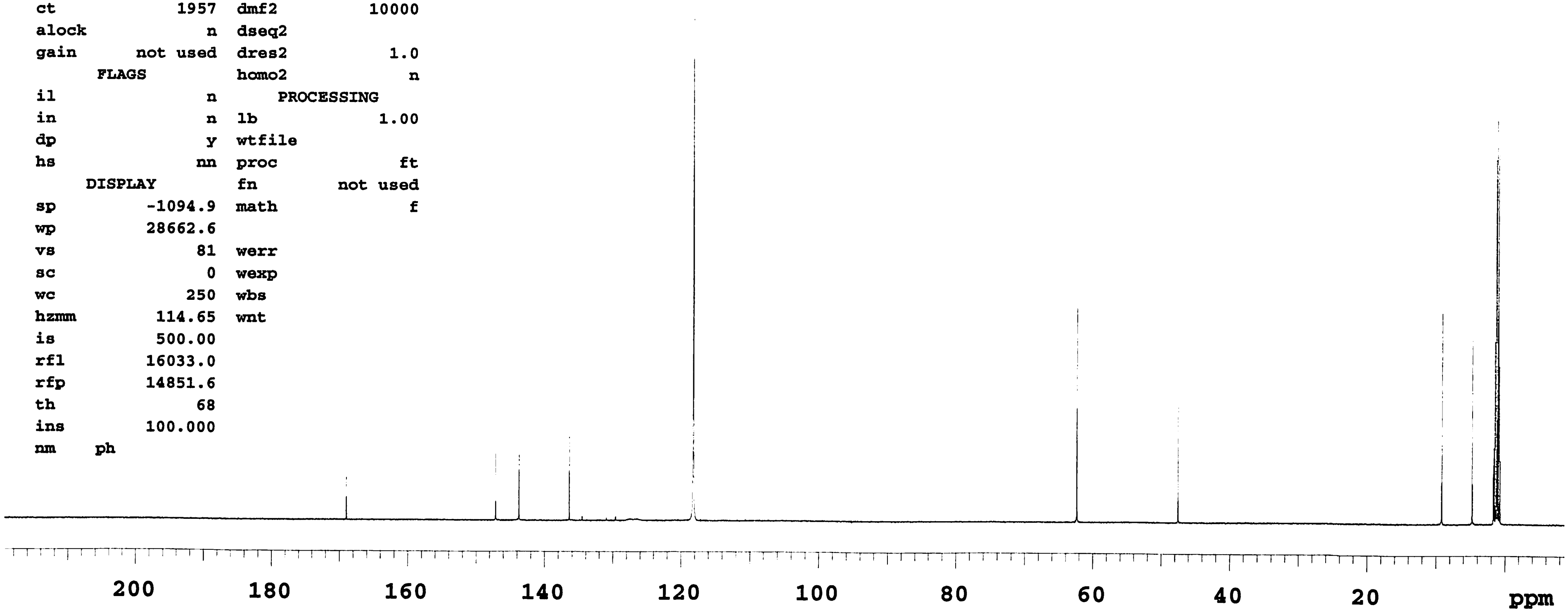

LSJV-68

exp1 s2pul

SAMPLE		DEC. & VT	
date	Jun 14 2010	dfrq	499.696
solvent	CD3CN	dn	H1
file	exp	dpwr	20
ACQUISITION		dof	0
sfrq	499.696	dm	nnn
tn	H1	dmm	c
at	4.665	dmf	200
np	65536	dseq	
sw	7024.9	dres	1.0
fb	4000	homo	n
bs	4	DEC2	
tpwr	63	dfrq2	0
pw	6.5	dn2	
d1	0	dpwr2	1
tof	2.0	dof2	0
nt	32	dm2	n
ct	32	dmm2	c
alock	n	dmf2	200
gain	not used	dseq2	
FLAGS		dres2	1.0
il	n	homo2	n
in	n	PROCESSING	
dp	y	lb	not used
hs	nn	wtfile	
DISPLAY		proc	ft
sp	47.5	fn	not used
wp	5422.2	math	f
vs	50		
sc	0	werr	
wc	250	wexp	svf(nl)
hzmm	21.69	wbs	
is	2600.34	wnt	wft('acq')
rfl	1993.8		
rfp	964.4		
th	5		
ins	3.000		
ai	ph		

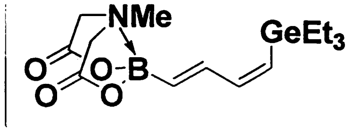

(*Z*,*E*)-8

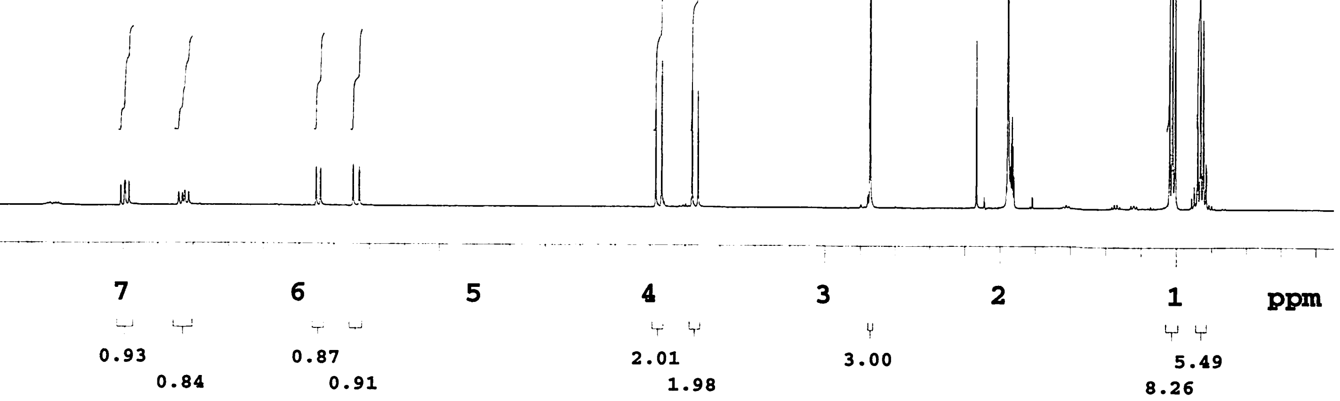

LSJV-68-13C

exp1 s2pul

SAMPLE		DEC. & VT	
date	Jun 14 2010	dfrq	499.695
solvent	CD3CN	dn	H1
file	exp	dpwr	44
ACQUISITION		dof	-827.6
sfrq	125.662	dm	yyy
tn	C13	dmm	w
at	1.086	dmf	19608
np	65536	dseq	
sw	30165.9	dres	90.0
fb	17000	homo	n
bs	16	DEC2	
ss	1	dfrq2	0
tpwr	54	dn2	
pw	6.0	dpwr2	1
d1	1.000	dof2	0
tof	1884.7	dm2	n
nt	11111	dmm2	c
ct	1813	dmf2	10000
alock	n	dseq2	
gain	not used	dres2	1.0
FLAGS		homo2	n
il	n	PROCESSING	
in	n	lb	1.00
dp	y	wtfile	
hs	nn	proc	ft
DISPLAY		fn	not used
sp	-1135.4	math	f
wp	28616.6		
vs	143	werr	
sc	0	wexp	
wc	250	wbs	
hzmm	114.47	wnt	
is	500.00		
rfl	16032.1		
rfp	14851.6		
th	68		
ins	100.000		
nm ph			

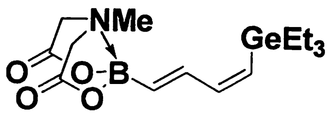

(*Z*,*E*)-8

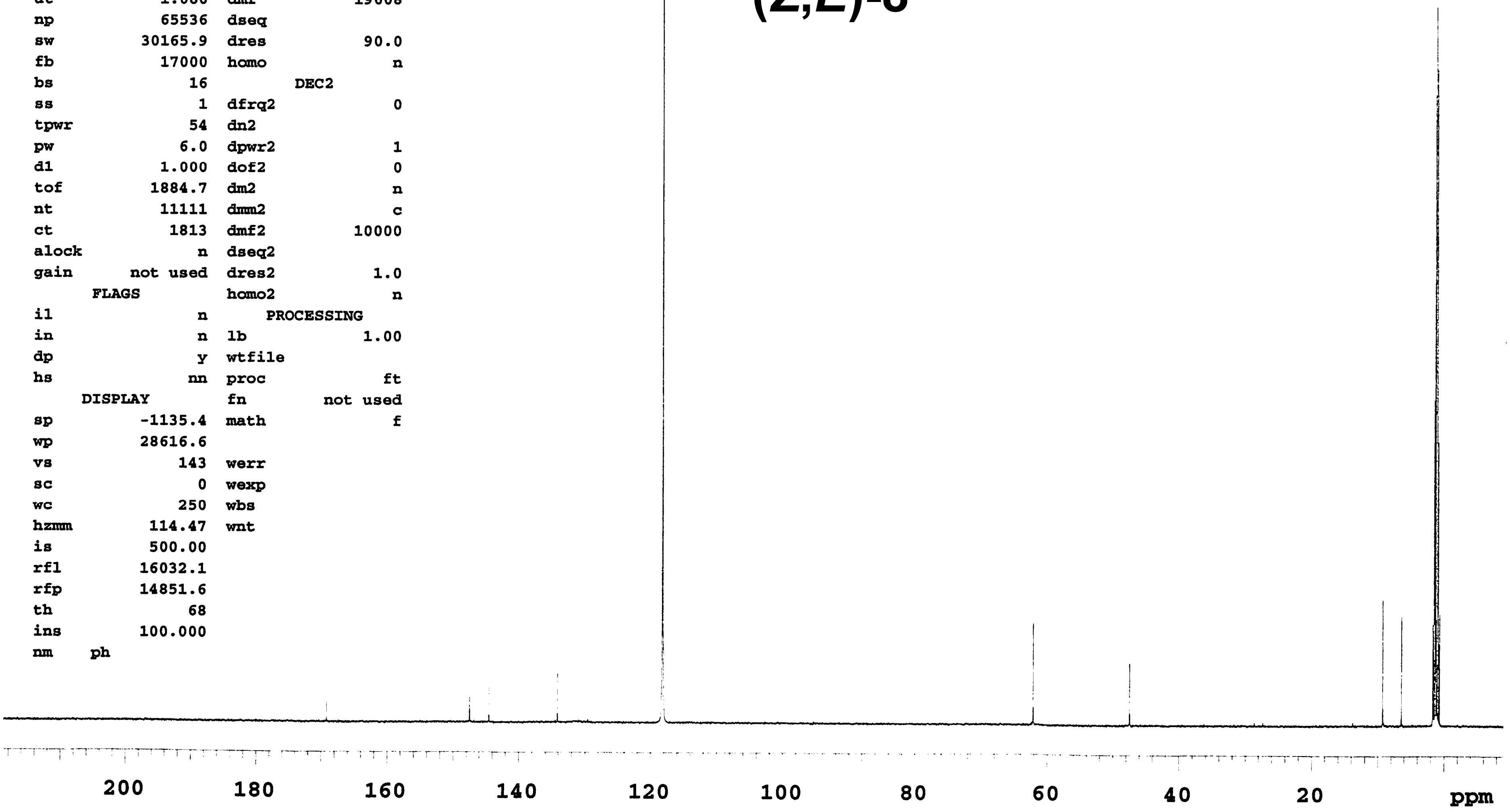

LSJV-74

exp1 s2pul

SAMPLE		DEC. & VT	
date	Jun 14 2010	dfrq	499.696
solvent	CD3CN	dn	H1
file	exp	dpwr	20
ACQUISITION		dof	0
sfrq	499.696	dm	nnn
tn	H1	dmm	c
at	4.665	dmf	200
np	65536	dseq	
sw	7024.9	dres	1.0
fb	4000	homo	n
bs	4	DEC2	
tpwr	63	dfrq2	0
pw	6.5	dn2	
d1	0	dpwr2	1
tof	2.0	dof2	0
nt	32	dm2	n
ct	32	dmm2	c
alock	n	dmf2	200
gain	not used	dseq2	
FLAGS		dres2	1.0
il	n	homo2	n
in	n	PROCESSING	
dp	y	lb	not used
hs	nn	wtfile	
DISPLAY		proc	ft
sp	19.0	fn	not used
wp	5461.2	math	f
vs	45		
sc	0	werr	
wc	250	wexp	svf(n1)
hzmm	21.84	wbs	
is	1174.19	wnt	wft('acq')
rfl	1993.8		
rfp	964.4		
th	7		
ins	3.000		
ai	ph		

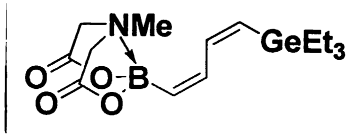

(Z,Z)-8

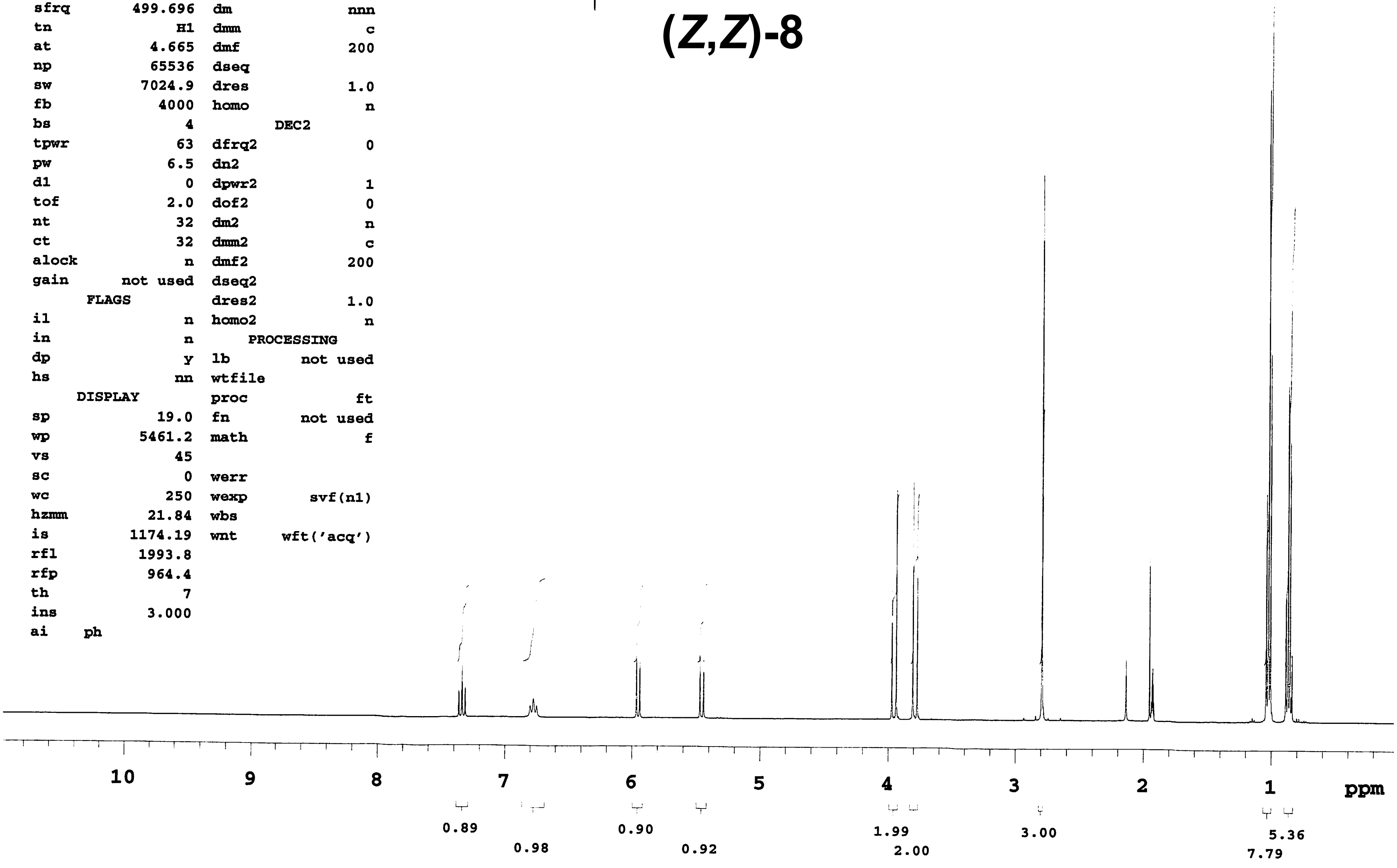

LSJV-74-13C

exp1 s2pul

SAMPLE		DEC. & VT	
date	Jun 14 2010	dfrq	499.695
solvent	CD3CN	dn	H1
file	exp	dpwr	44
ACQUISITION		dof	-827.6
sfrq	125.662	dm	yyy
tn	C13	dmm	w
at	1.086	dmf	19608
np	65536	dseq	
sw	30165.9	dres	90.0
fb	17000	homo	n
bs	16	DEC2	
ss	1	dfrq2	0
tpwr	54	dn2	
pw	6.0	dpwr2	1
d1	1.000	dof2	0
tof	1884.7	dm2	n
nt	11111	dmm2	c
ct	1757	dmf2	10000
alock	n	dseq2	
gain	not used	dres2	1.0
FLAGS		homo2	n
il	n	PROCESSING	
in	n	lb	1.00
dp	y	wtfile	
hs	nn	proc	ft
DISPLAY		fn	not used
sp	-1136.3	math	f
wp	28749.1		
vs	118	werr	
sc	0	wexp	
wc	250	wbs	
hzmm	115.00	wnt	
is	500.00		
rfl	16033.0		
rfp	14851.6		
th	68		
ins	100.000		
nm ph			

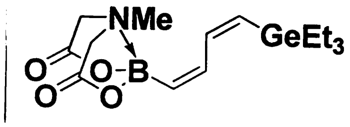

(Z,Z)-8

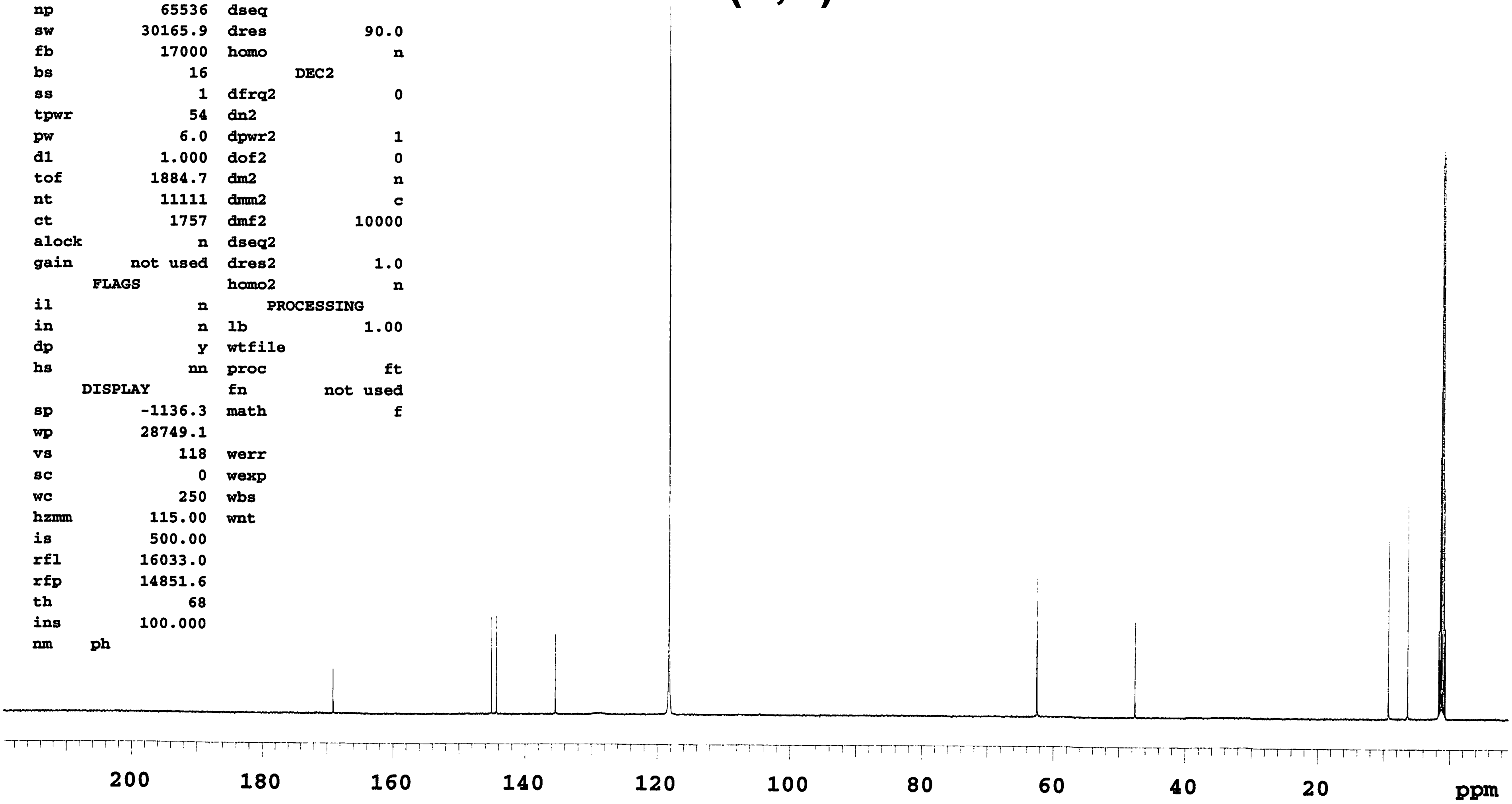

LSJIV-10-2(Purification)

exp2 s2pul

```
      SAMPLE               DEC. & VT
date   Dec 12 2008   dfrq       499.696
solvent      CD3CN   dn              H1
file           exp   dpwr            20
   ACQUISITION       dof              0
sfrq       499.696   dm             nnn
tn              H1   dmm              c
at           4.665   dmf            200
np           65536   dseq
sw          7024.9   dres           1.0
fb            4000   homo             n
bs               4          DEC2
tpwr            63   dfrq2            0
pw             6.5   dn2
d1               0   dpwr2            1
tof            2.0   dof2             0
nt              16   dm2              n
ct              16   dmm2             c
alock            n   dmf2           200
gain      not used   dseq2
      FLAGS          dres2          1.0
il               n   homo2            n
in               n      PROCESSING
dp               y   lb        not used
hs              nn   wtfile
     DISPLAY         proc            ft
sp             6.3   fn        not used
wp          5489.1   math             f
vs              34
sc               0   werr
wc             250   wexp      svf(n1)
hzmm         21.96   wbs
is         1068.02   wnt    wft('acq')
rfl         1993.6
rfp          964.4
th               7
ins          1.000
ai    ph
```

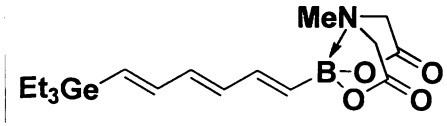

(*E*,*E*,*E*)-9

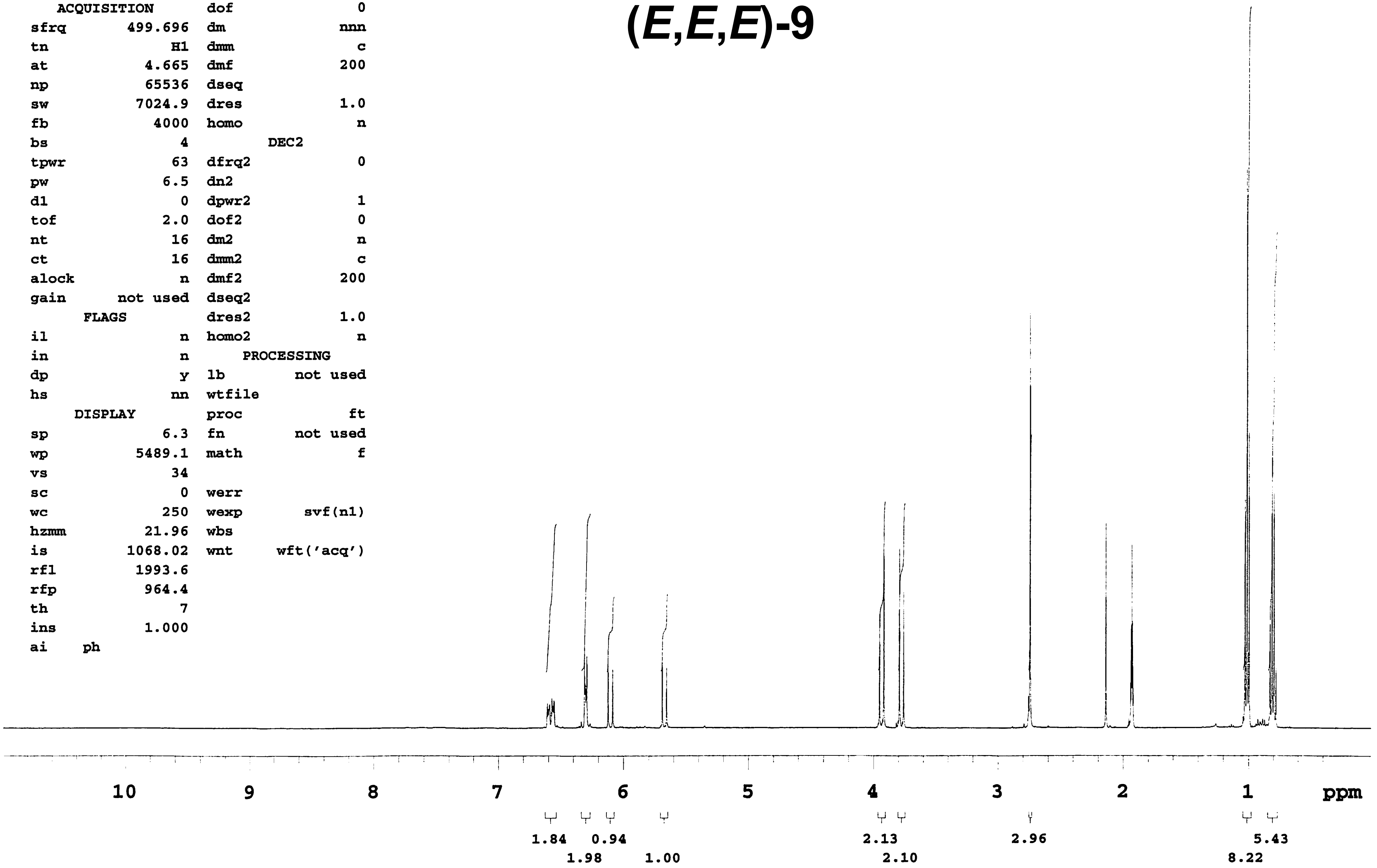

LSJIV-45-13C

exp1 s2pul

SAMPLE		DEC. & VT	
date	Jun 14 2010	dfrq	499.695
solvent	CD3CN	dn	H1
file	exp	dpwr	44
ACQUISITION		dof	-827.6
sfrq	125.662	dm	yyy
tn	C13	dmm	w
at	1.086	dmf	19608
np	65536	dseq	
sw	30165.9	dres	90.0
fb	17000	homo	n
bs	16	DEC2	
ss	1	dfrq2	0
tpwr	54	dn2	
pw	6.0	dpwr2	1
d1	1.000	dof2	0
tof	1884.7	dm2	n
nt	11111	dmm2	c
ct	703	dmf2	10000
alock	n	dseq2	
gain	not used	dres2	1.0
FLAGS		homo2	n
il	n	PROCESSING	
in	n	lb	1.00
dp	y	wtfile	
hs	nn	proc	ft
DISPLAY		fn	not used
sp	-1093.1	math	f
wp	28660.7		
vs	71	werr	
sc	0	wexp	
wc	250	wbs	
hzmm	114.64	wnt	
is	500.00		
rfl	16033.9		
rfp	14851.6		
th	68		
ins	100.000		
nm	ph		

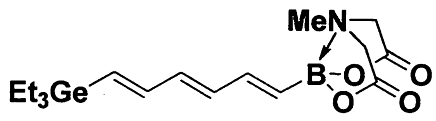

(*E*,*E*,*E*)-9

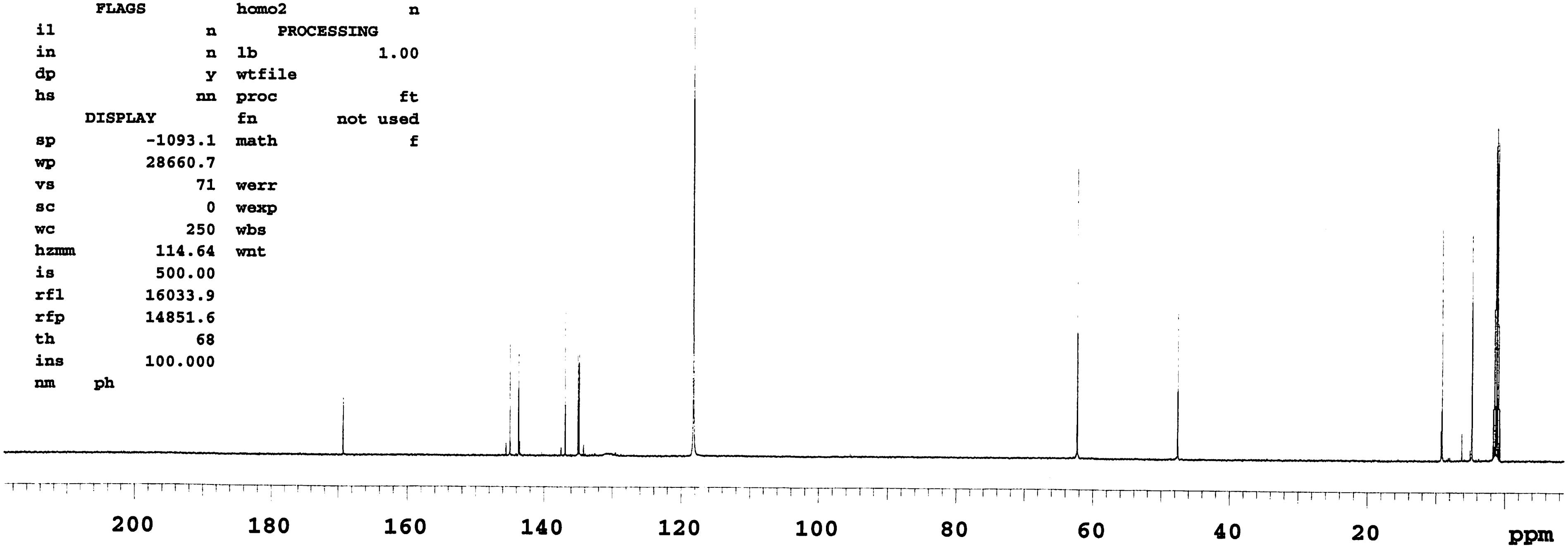

Z_pentenyl_boronic_acid_071010

exp1 std1h

SAMPLE		DEC. & VT	
date	Jul 10 2010	dn	H1
solvent	CDCl3	dof	0
file	exp	dm	nnn
ACQUISITION		dmm	c
sfrq	399.947	dmf	200
tn	H1	dpwr	20
at	4.096	PROCESSING	
np	65536	lb	0.30
sw	8000.0	wtfile	
fb	4000	proc	ft
bs	16	fn	131072
tpwr	58	math	f
pw	5.8		
d1	25.000	werr	
tof	-425.7	wexp	
nt	64	wbs	
ct	64	wnt	
alock	n	DISPLAY	
gain	not used	sp	-2416.8
FLAGS		wp	7999.9
il	n	vs	162
in	n	sc	0
dp	y	wc	250
hs	nn	hzmm	16.00
		is	0.01
		rfl	5320.6
		rfp	2903.6
		th	12
		ins	3.000
		nm ph	

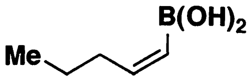

(*Z*)-10

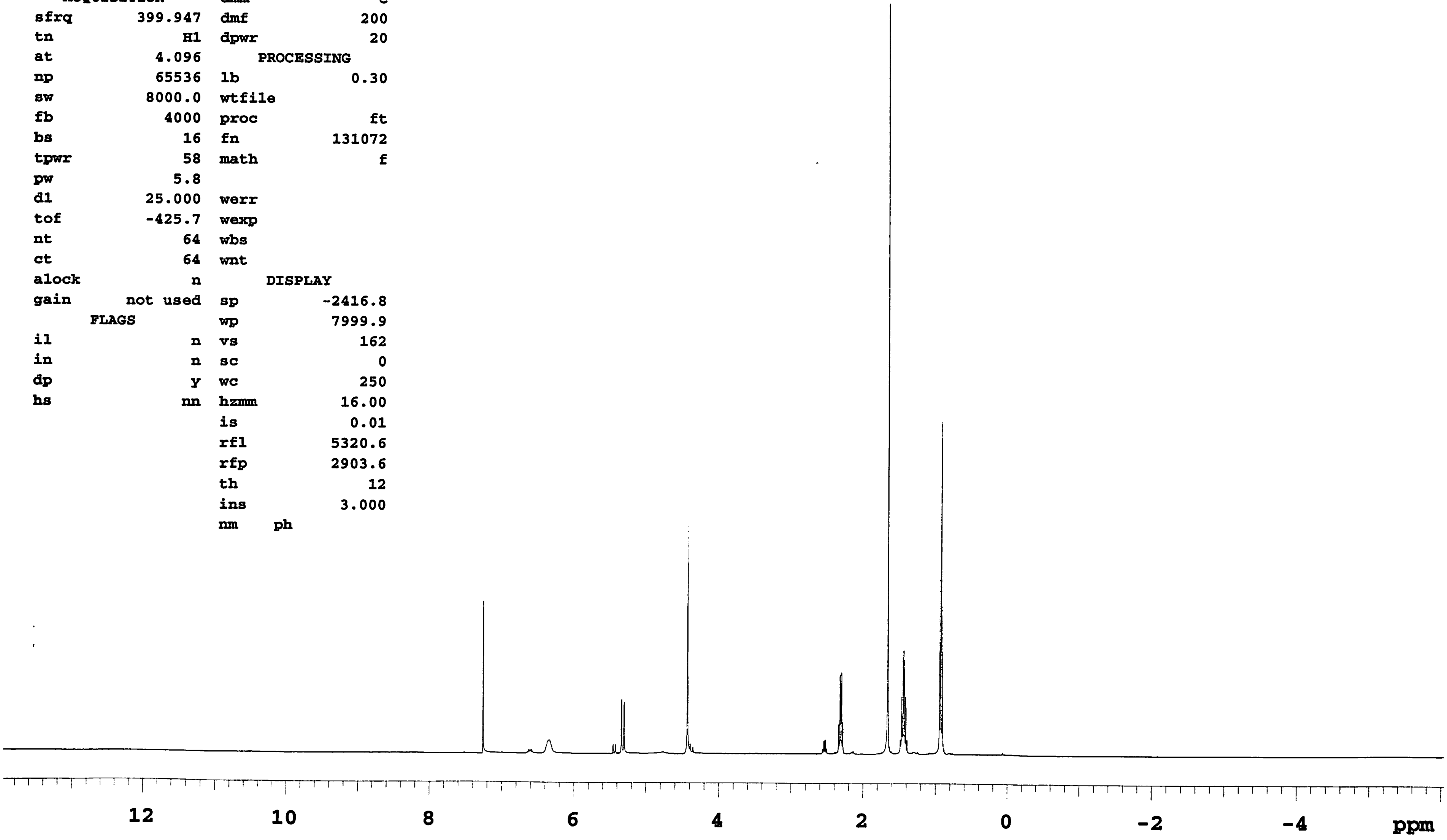

LSJV-45(Purification)

expl stdlh

SAMPLE		DEC. & VT	
date	Dec 6 2009	dn	H1
solvent	CD3CN	dof	0
file	exp	dm	nnn
ACQUISITION		dmm	c
sfrq	399.949	dmf	200
tn	H1	dpwr	20
at	4.096	PROCESSING	
np	65536	lb	not used
sw	8000.0	wtfile	
fb	4000	proc	ft
bs	16	fn	not used
tpwr	58	math	f
pw	5.8		
d1	0	werr	
tof	-425.7	wexp	svf(n1)
nt	32	wbs	
ct	32	wnt	wft('acq')
alock	n	DISPLAY	
gain	not used	sp	8.2
FLAGS		wp	4354.0
il	n	vs	102
in	n	sc	0
dp	y	wc	250
hs	nn	hzmm	17.42
		is	1791.68
		rfl	3202.1
		rfp	771.9
		th	12
		ins	3.000
		nm	ph

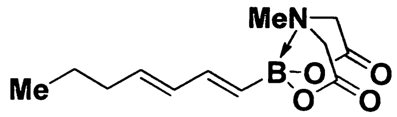

11

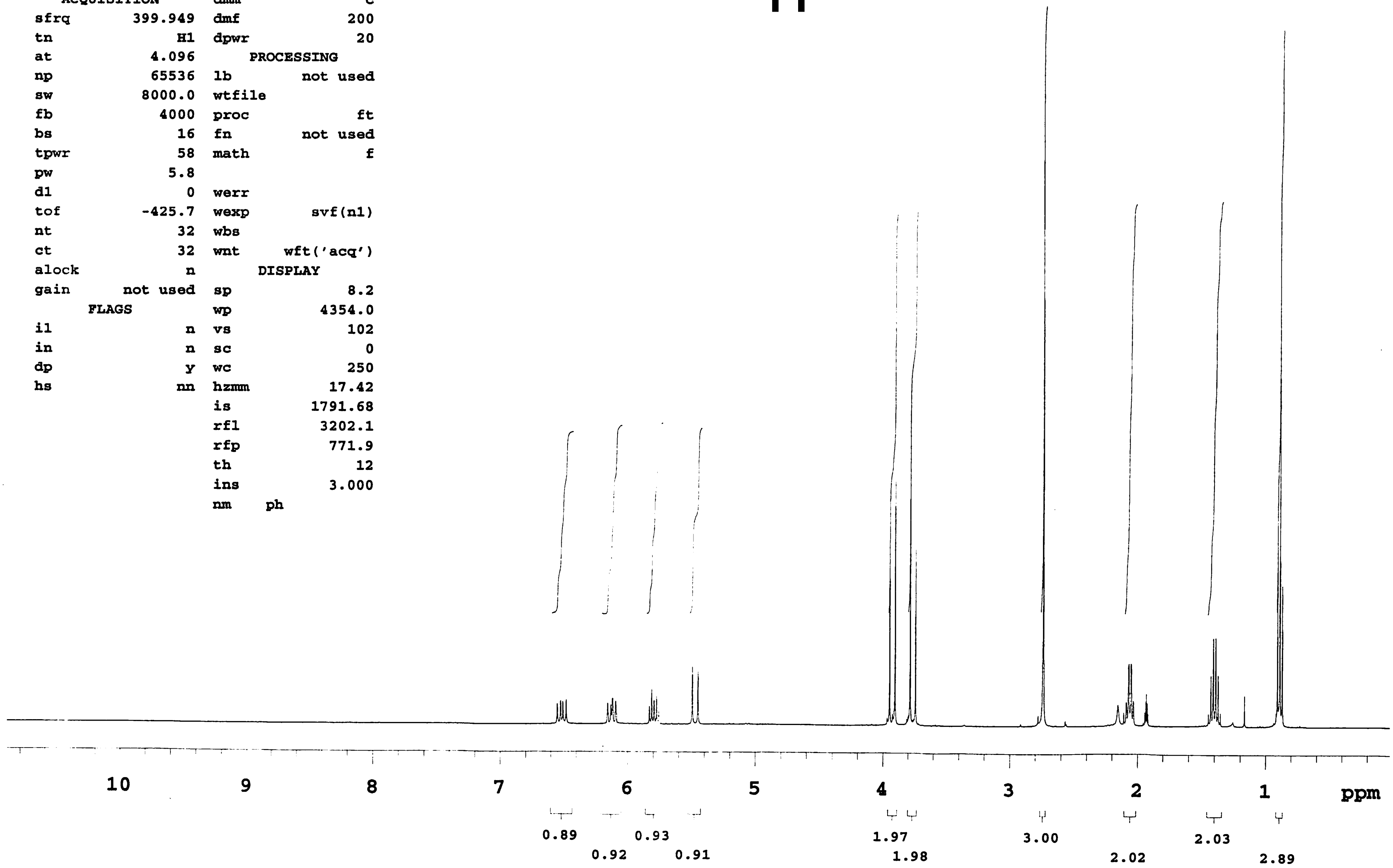

LSJV-57

exp1 s2pul

SAMPLE		DEC. & VT	
date	Jan 23 2010	dfrq	499.696
solvent	CD3CN	dn	H1
file	exp	dpwr	20
ACQUISITION		dof	0
sfrq	499.696	dm	nnn
tn	H1	dmm	c
at	4.665	dmf	200
np	65536	dseq	
sw	7024.9	dres	1.0
fb	4000	homo	n
bs	4	DEC2	
tpwr	63	dfrq2	0
pw	6.5	dn2	
d1	0	dpwr2	1
tof	2.0	dof2	0
nt	32	dm2	n
ct	32	dmm2	c
alock	n	dmf2	200
gain	not used	dseq2	
FLAGS		dres2	1.0
il	n	homo2	n
in	n	PROCESSING	
dp	y	lb	not used
hs	nn	wtfile	
DISPLAY		proc	ft
sp	11.5	fn	not used
wp	5330.7	math	f
vs	29		
sc	0	werr	
wc	250	wexp	svf(n1)
hzmm	21.32	wbs	
is	2096.53	wnt	wft('acq')
rfl	1993.6		
rfp	964.4		
th	19		
ins	1.000		
ai	ph		

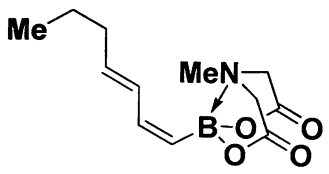

12

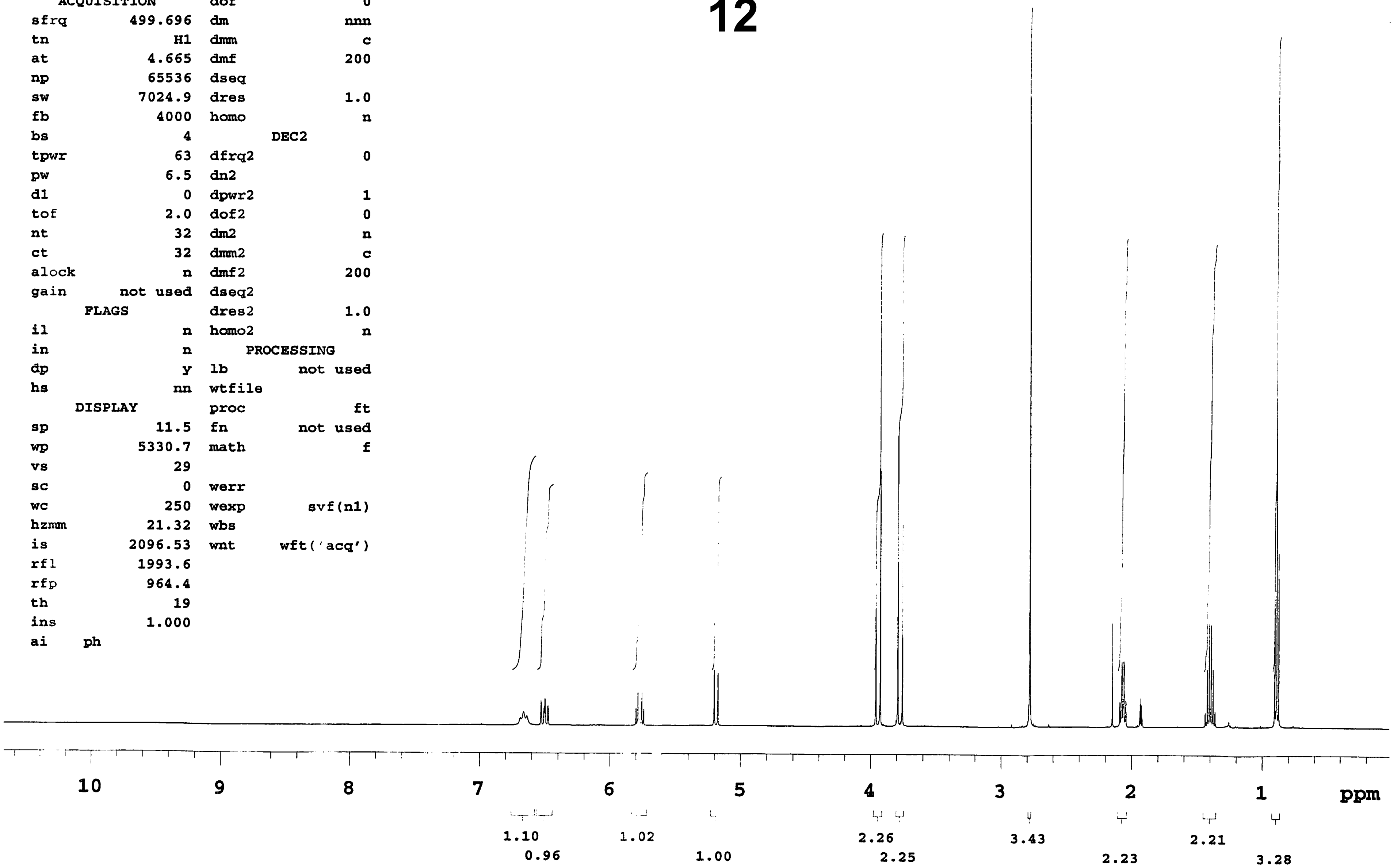

LSJV-57-13C

exp1 s2pul

**SAMPLE**		**DEC. & VT**	
date	Jan 23 2010	dfrq	499.695
solvent	CD3CN	dn	H1
file	exp	dpwr	44
**ACQUISITION**		dof	-827.6
sfrq	125.662	dm	yyy
tn	C13	dmm	w
at	1.086	dmf	19608
np	65536	dseq	
sw	30165.9	dres	90.0
fb	17000	homo	n
bs	16	**DEC2**	
ss	1	dfrq2	0
tpwr	54	dn2	
pw	6.0	dpwr2	1
d1	1.000	dof2	0
tof	1884.7	dm2	n
nt	3000	dmm2	c
ct	2017	dmf2	10000
alock	n	dseq2	
gain	not used	dres2	1.0
**FLAGS**		homo2	n
il	n	**PROCESSING**	
in	n	lb	1.00
dp	y	wtfile	
hs	nn	proc	ft
**DISPLAY**		fn	not used
sp	-1139.1	math	f
wp	28110.2		
vs	80	werr	
sc	0	wexp	
wc	250	wbs	
hzmm	112.44	wnt	
is	500.00		
rfl	16034.8		
rfp	14851.6		
th	6		
ins	100.000		
nm	ph		

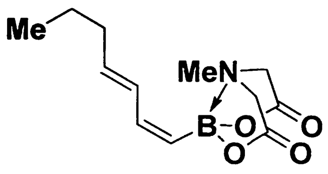

12

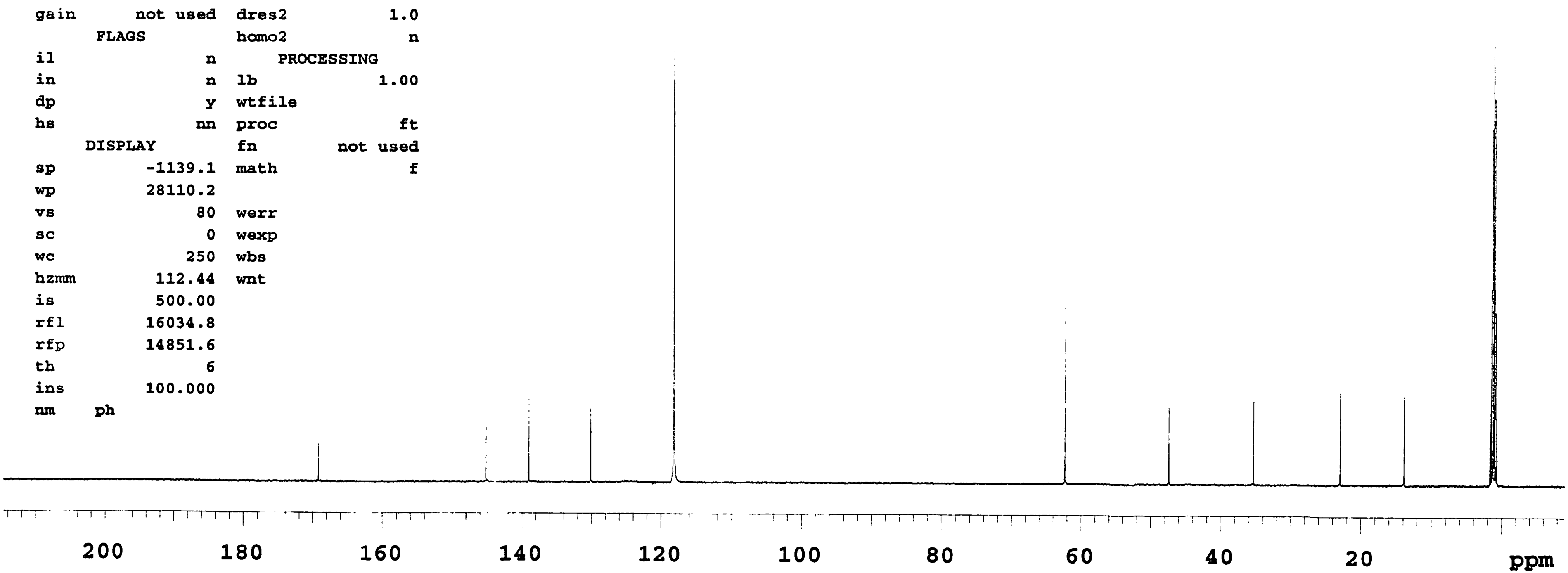

LSJV-62

exp1 s2pul

SAMPLE		DEC. & VT	
date	Jan 31 2010	dfrq	499.696
solvent	CD3CN	dn	H1
file	exp	dpwr	20
ACQUISITION		dof	0
sfrq	499.696	dm	nnn
tn	H1	dmm	c
at	4.665	dmf	200
np	65536	dseq	
sw	7024.9	dres	1.0
fb	4000	homo	n
bs	4	DEC2	
tpwr	63	dfrq2	0
pw	6.5	dn2	
d1	0	dpwr2	1
tof	2.0	dof2	0
nt	32	dm2	n
ct	32	dmm2	c
alock	n	dmf2	200
gain	not used	dseq2	
FLAGS		dres2	1.0
il	n	homo2	n
in	n	PROCESSING	
dp	y	lb	not used
hs	nn	wtfile	
DISPLAY		proc	ft
sp	36.6	fn	not used
wp	5422.8	math	f
vs	43		
sc	0	werr	
wc	250	wexp	svf(n1)
hzmm	21.69	wbs	
is	2368.57	wnt	wft('acq')
rfl	1993.6		
rfp	964.4		
th	7		
ins	1.000		
ai	ph		

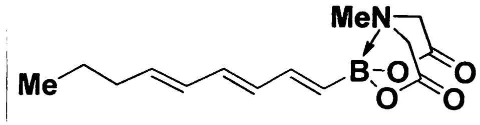

13

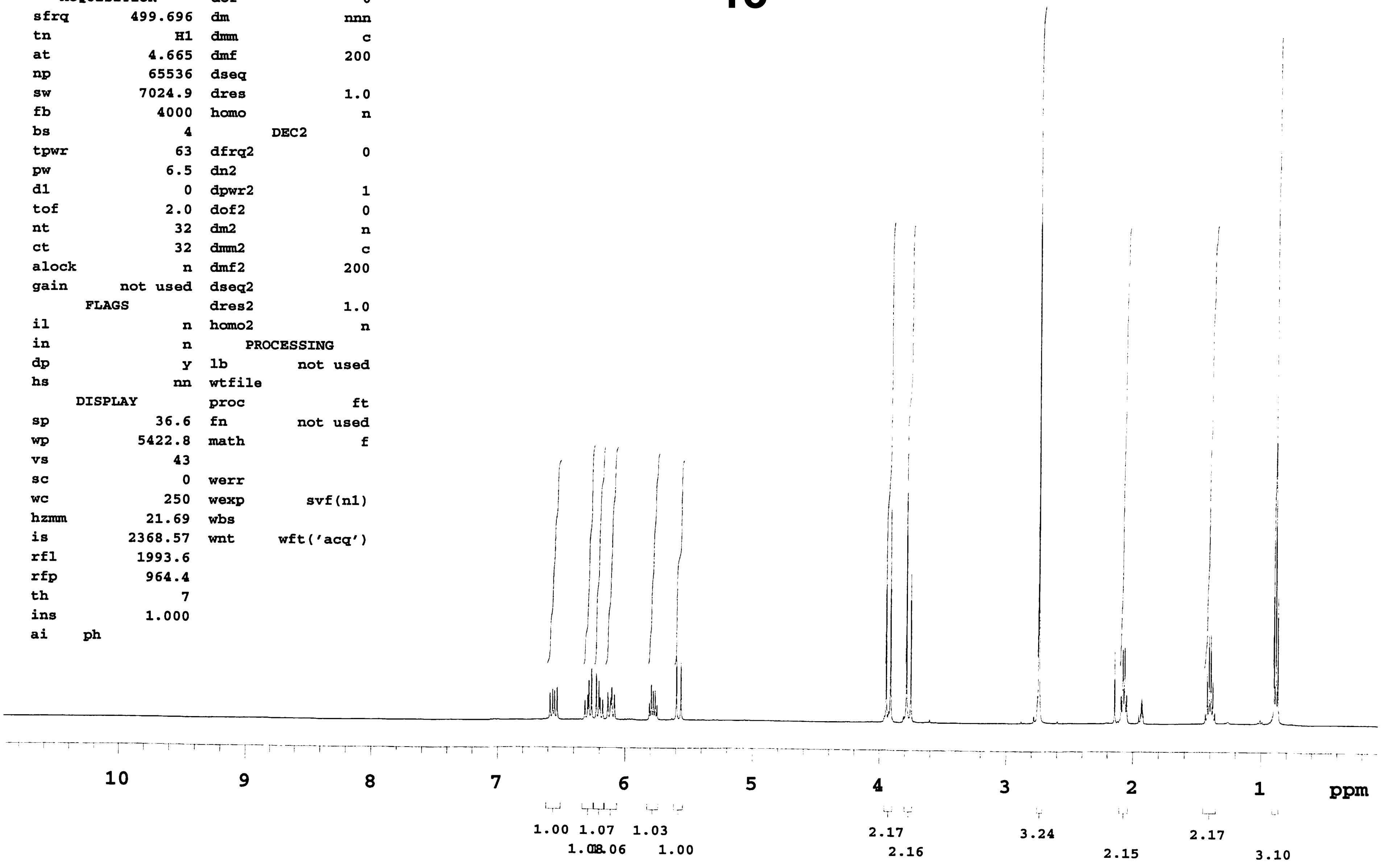

LSJV-62-13C

exp1 s2pul

SAMPLE		DEC. & VT	
date	Jan 31 2010	dfrq	499.695
solvent	CD3CN	dn	H1
file	exp	dpwr	44
ACQUISITION		dof	-827.6
sfrq	125.662	dm	yyy
tn	C13	dmm	w
at	1.086	dmf	19608
np	65536	dseq	
sw	30165.9	dres	90.0
fb	17000	homo	n
bs	16	DEC2	
ss	1	dfrq2	0
tpwr	54	dn2	
pw	6.0	dpwr2	1
d1	1.000	dof2	0
tof	1884.7	dm2	n
nt	5000	dmm2	c
ct	1861	dmf2	10000
alock	n	dseq2	
gain	not used	dres2	1.0
FLAGS		homo2	n
il	n	PROCESSING	
in	n	lb	1.00
dp	y	wtfile	
hs	nn	proc	ft
DISPLAY		fn	not used
sp	-1099.5	math	f
wp	28680.1		
vs	97	werr	
sc	0	wexp	
wc	250	wbs	
hzmm	114.72	wnt	
is	500.00		
rfl	16034.8		
rfp	14851.6		
th	68		
ins	100.000		
nm	ph		

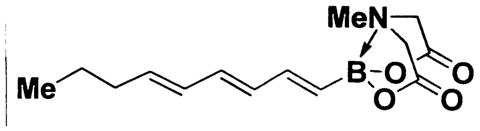

13

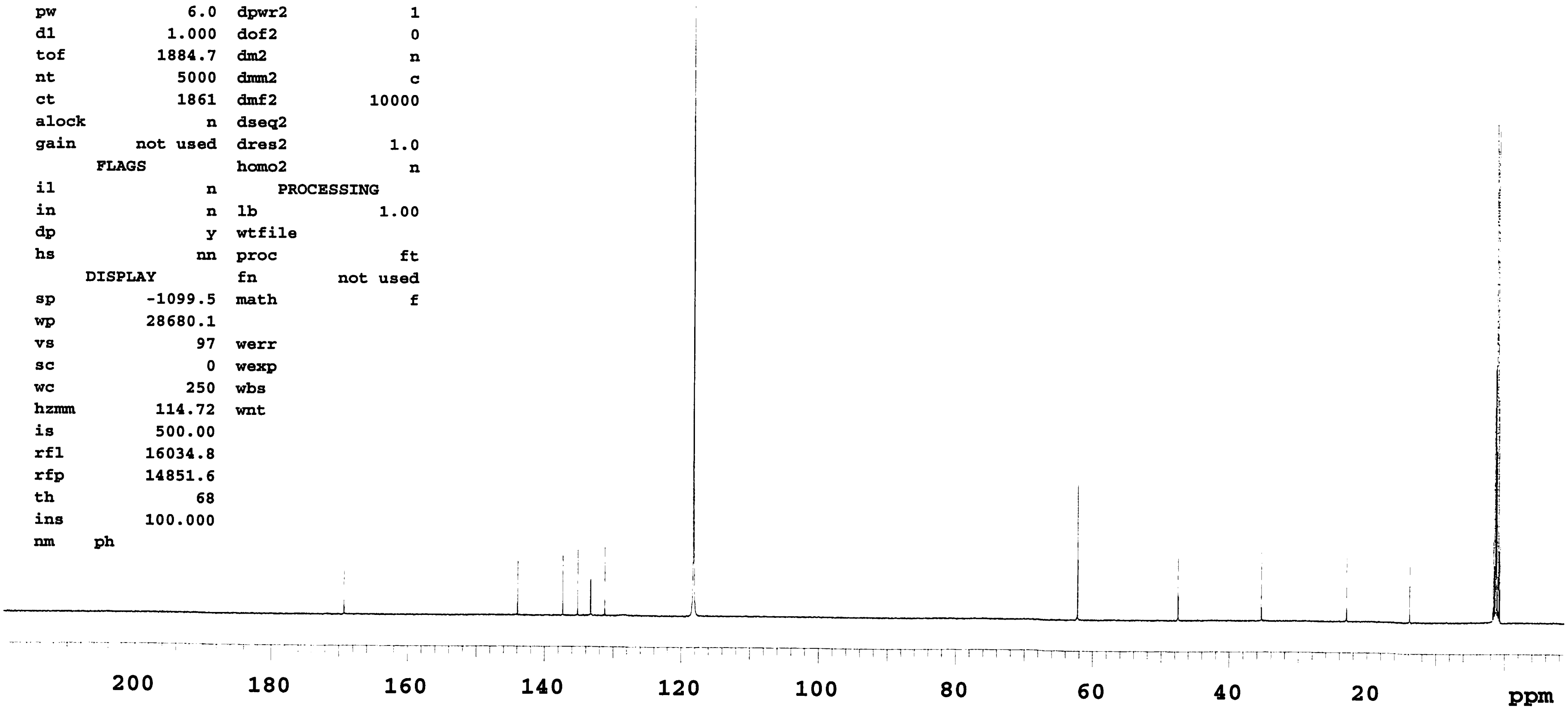

LSJV-66

exp1 s2pul

SAMPLE		DEC. & VT	
date	Feb 14 2010	dfrq	499.696
solvent	CD3CN	dn	H1
file	exp	dpwr	20
ACQUISITION		dof	0
sfrq	499.696	dm	nnn
tn	H1	dmm	c
at	4.665	dmf	200
np	65536	dseq	
sw	7024.9	dres	1.0
fb	4000	homo	n
bs	4	DEC2	
tpwr	63	dfrq2	0
pw	6.5	dn2	
d1	0	dpwr2	1
tof	2.0	dof2	0
nt	32	dm2	n
ct	32	dmm2	c
alock	n	dmf2	200
gain	not used	dseq2	
FLAGS		dres2	1.0
il	n	homo2	n
in	n	PROCESSING	
dp	y	lb	not used
hs	nn	wtfile	
DISPLAY		proc	ft
sp	26.3	fn	not used
wp	5438.7	math	f
vs	24		
sc	0	werr	
wc	250	wexp	svf(n1)
hzmm	21.75	wbs	
is	1979.16	wnt	wft('acq')
rfl	1993.6		
rfp	964.4		
th	7		
ins	1.000		
ai	ph		

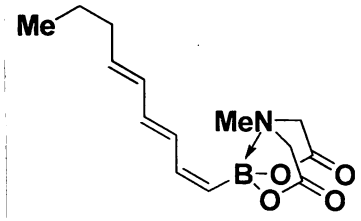

14

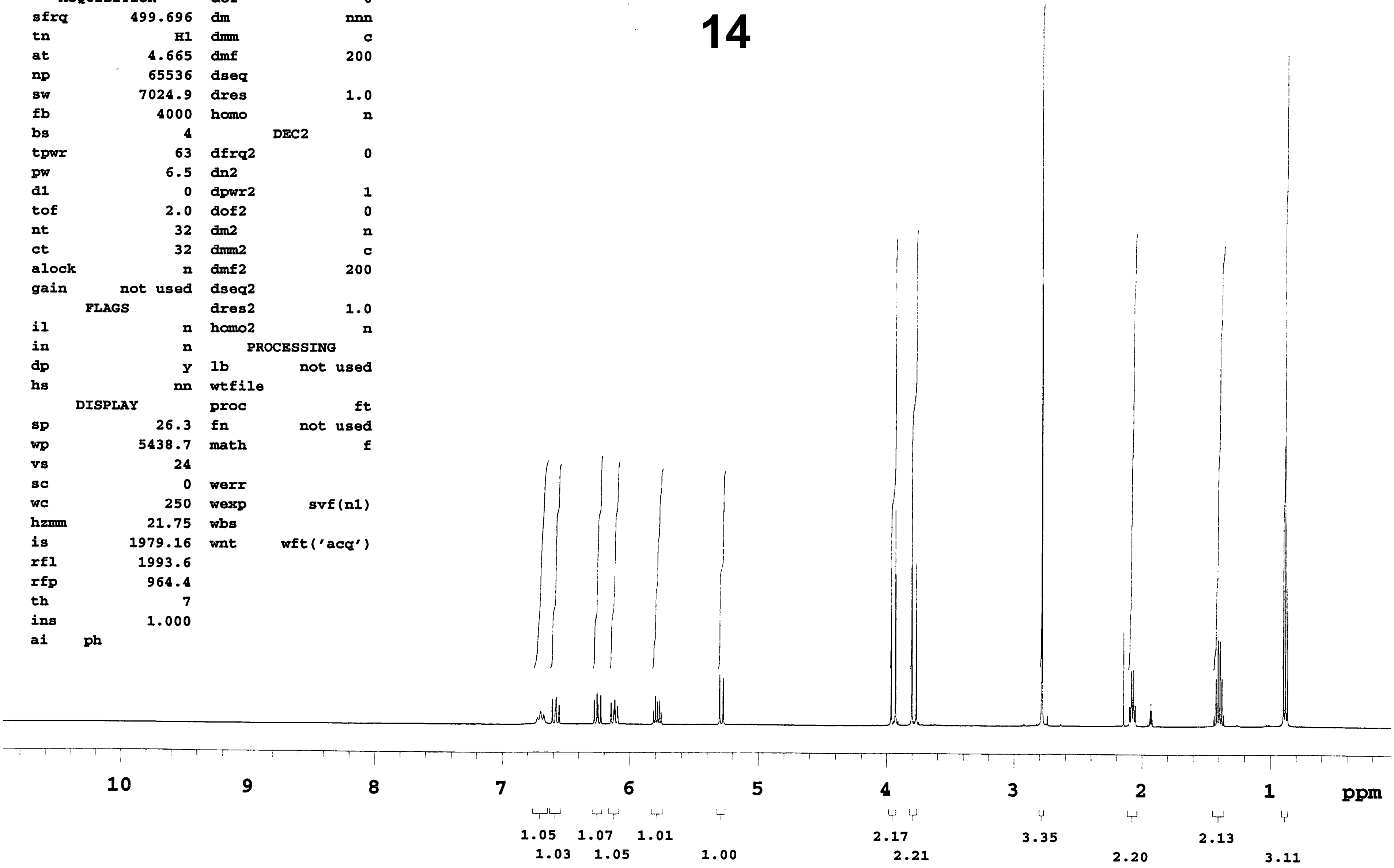

LSJV-66-13C

exp1 s2pul

SAMPLE		DEC. & VT	
date	Feb 14 2010	dfrq	499.695
solvent	CD3CN	dn	H1
file	exp	dpwr	44
ACQUISITION		dof	-827.6
sfrq	125.662	dm	yyy
tn	C13	dmm	w
at	1.086	dmf	19608
np	65536	dseq	
sw	30165.9	dres	90.0
fb	17000	homo	n
bs	16	DEC2	
ss	1	dfrq2	0
tpwr	54	dn2	
pw	6.0	dpwr2	1
d1	1.000	dof2	0
tof	1884.7	dm2	n
nt	5000	dmm2	c
ct	2509	dmf2	10000
alock	n	dseq2	
gain	not used	dres2	1.0
FLAGS		homo2	n
il	n	PROCESSING	
in	n	lb	1.00
dp	y	wtfile	
hs	nn	proc	ft
DISPLAY		fn	not used
sp	-1057.1	math	f
wp	28635.0		
vs	83	werr	
sc	0	wexp	
wc	250	wbs	
hzmm	114.54	wnt	
is	500.00		
rfl	16035.8		
rfp	14851.6		
th	68		
ins	100.000		
nm ph			

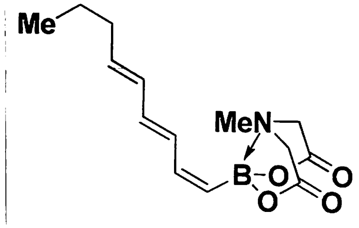

14

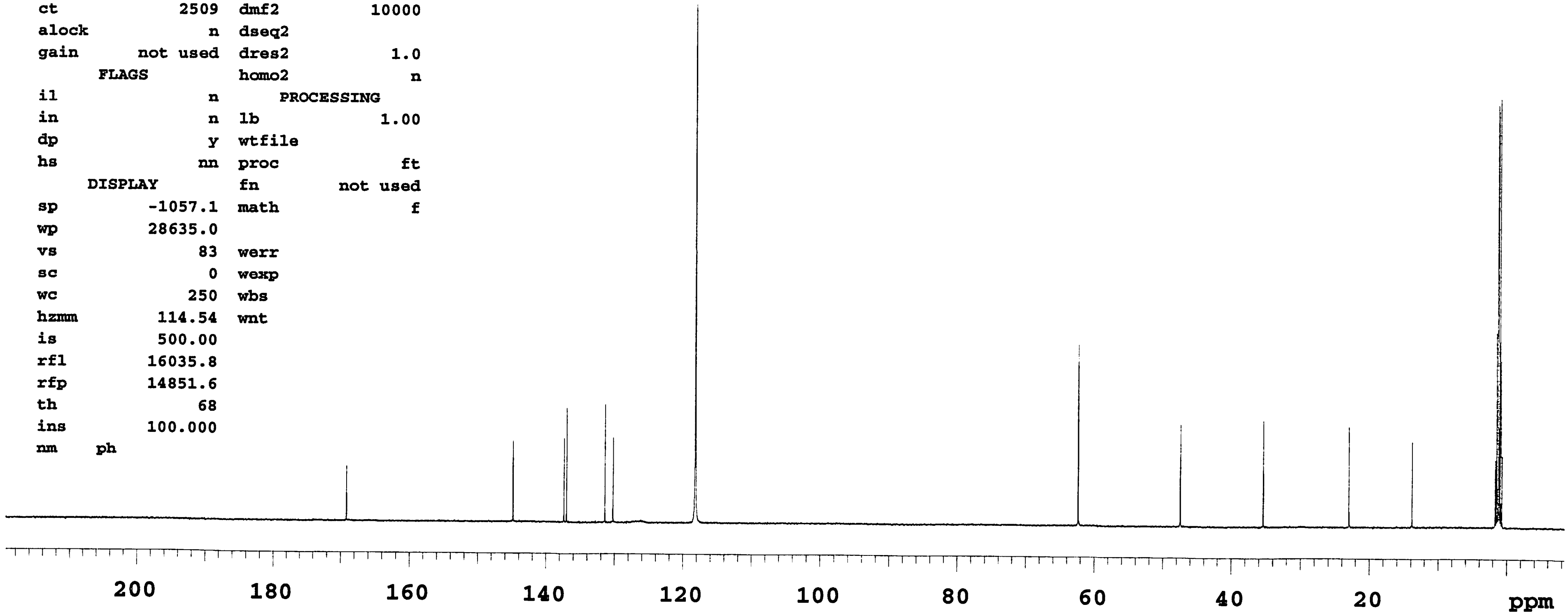

LSJV-70

exp1 s2pul

SAMPLE		DEC. & VT	
date	Feb 20 2010	dfrq	499.696
solvent	CD3CN	dn	H1
file	exp	dpwr	20
ACQUISITION		dof	0
sfrq	499.696	dm	nnn
tn	H1	dmm	c
at	4.665	dmf	200
np	65536	dseq	
sw	7024.9	dres	1.0
fb	4000	homo	n
bs	4	DEC2	
tpwr	63	dfrq2	0
pw	6.5	dn2	
d1	0	dpwr2	1
tof	2.0	dof2	0
nt	32	dm2	n
ct	32	dmm2	c
alock	n	dmf2	200
gain	not used	dseq2	
FLAGS		dres2	1.0
il	n	homo2	n
in	n	PROCESSING	
dp	y	lb	not used
hs	nn	wtfile	
DISPLAY		proc	ft
sp	25.6	fn	not used
wp	5444.9	math	f
vs	25		
sc	0	werr	
wc	250	wexp	svf(n1)
hzmm	21.78	wbs	
is	1950.77	wnt	wft('acq')
rfl	1993.6		
rfp	964.4		
th	7		
ins	1.000		
ai	ph		

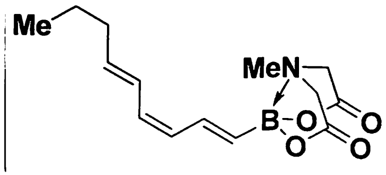

15

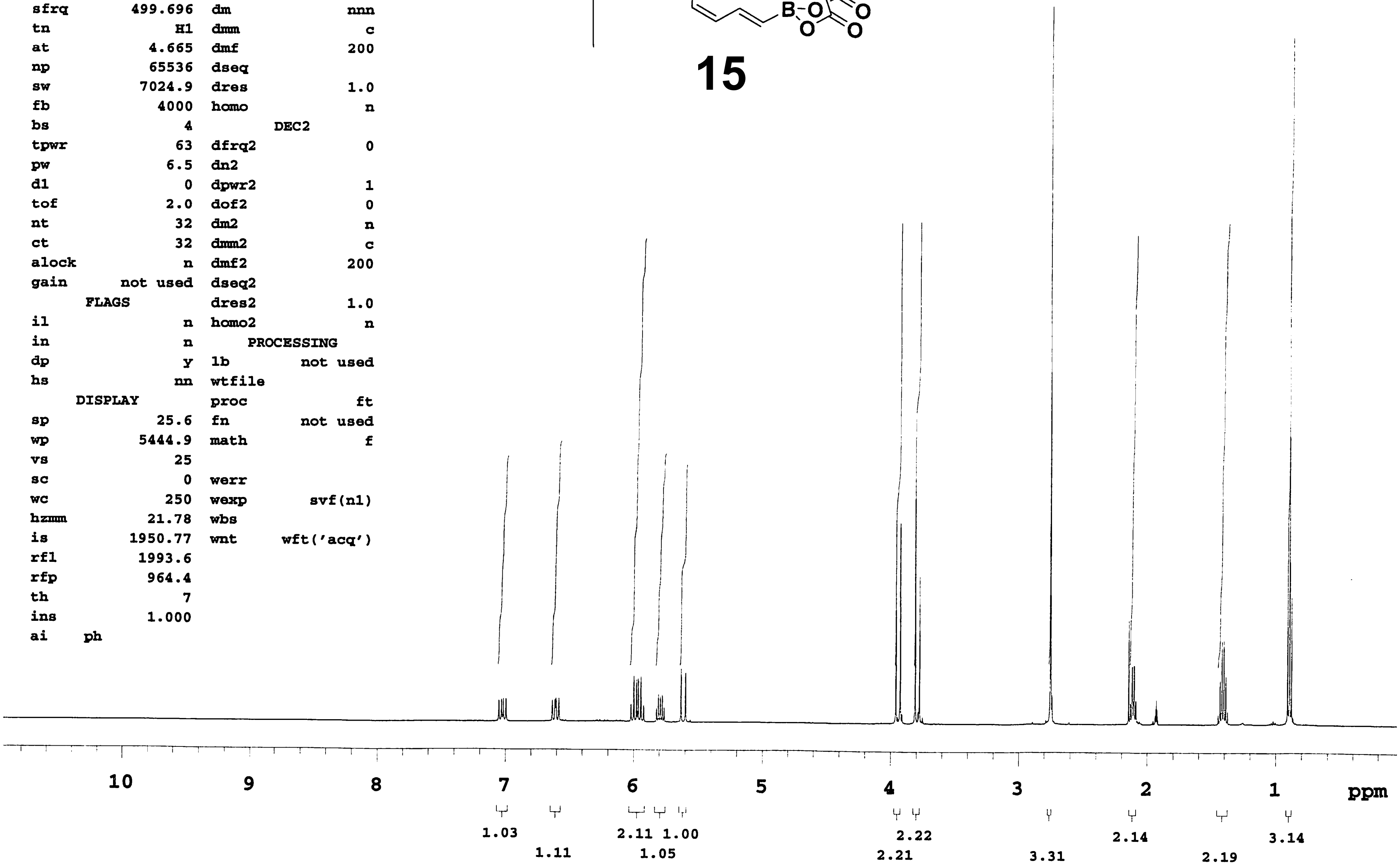

LSJV-70-13C

exp1 s2pul

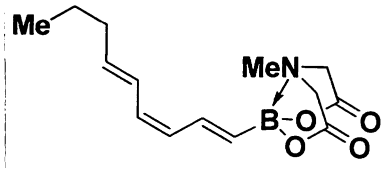

15

SAMPLE		DEC. & VT	
date	Feb 20 2010	dfrq	499.695
solvent	CD3CN	dn	H1
file	exp	dpwr	44
ACQUISITION		dof	-827.6
sfrq	125.662	dm	yyy
tn	C13	dmm	w
at	1.086	dmf	19608
np	65536	dseq	
sw	30165.9	dres	90.0
fb	17000	homo	n
bs	16	DEC2	
ss	1	dfrq2	0
tpwr	54	dn2	
pw	6.0	dpwr2	1
d1	1.000	dof2	0
tof	1884.7	dm2	n
nt	5000	dmm2	c
ct	1859	dmf2	10000
alock	n	dseq2	
gain	not used	dres2	1.0
FLAGS		homo2	n
il	n	PROCESSING	
in	n	lb	1.00
dp	y	wtfile	
hs	nn	proc	ft
DISPLAY		fn	not used
sp	-1013.9	math	f
wp	28493.2		
vs	88	werr	
sc	0	wexp	
wc	250	wbs	
hzmm	113.97	wnt	
is	500.00		
rfl	16033.9		
rfp	14851.6		
th	68		
ins	100.000		
nm	ph		

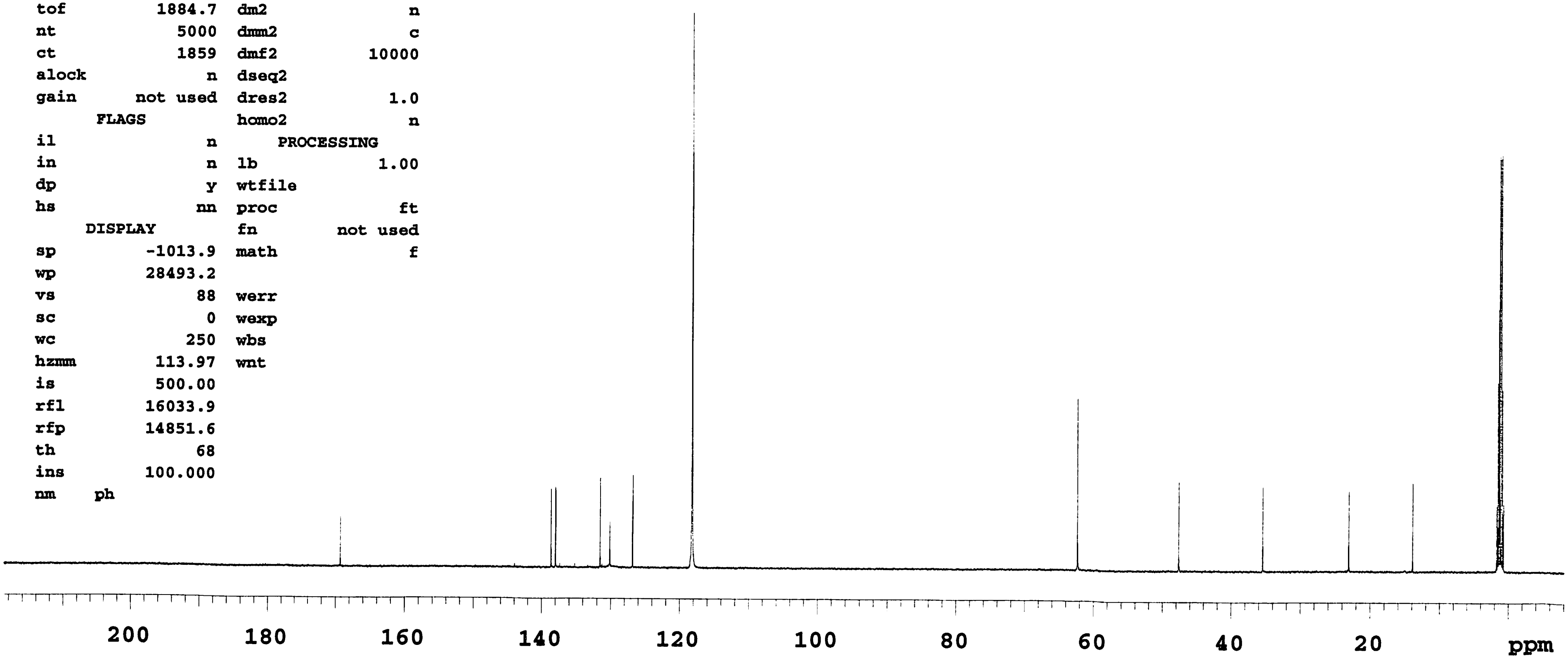

LSJV-76

exp1 s2pul

SAMPLE		DEC. & VT	
date	Mar 8 2010	dfrq	499.696
solvent	CD3CN	dn	H1
file	exp	dpwr	20
ACQUISITION		dof	0
sfrq	499.696	dm	nnn
tn	H1	dmm	c
at	4.665	dmf	200
np	65536	dseq	
sw	7024.9	dres	1.0
fb	4000	homo	n
bs	4	DEC2	
tpwr	63	dfrq2	0
pw	6.5	dn2	
d1	0	dpwr2	1
tof	2.0	dof2	0
nt	32	dm2	n
ct	32	dmm2	c
alock	n	dmf2	200
gain	not used	dseq2	
FLAGS		dres2	1.0
il	n	homo2	n
in	n	PROCESSING	
dp	y	lb	not used
hs	nn	wtfile	
DISPLAY		proc	ft
sp	38.9	fn	not used
wp	5426.3	math	f
vs	38		
sc	0	werr	
wc	250	wexp	svf(n1)
hzmm	21.71	wbs	
is	2975.85	wnt	wft('acq')
rfl	1993.6		
rfp	964.4		
th	7		
ins	1.000		
ai	ph		

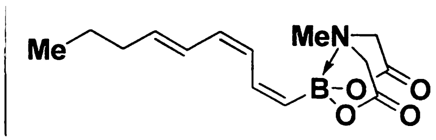

16

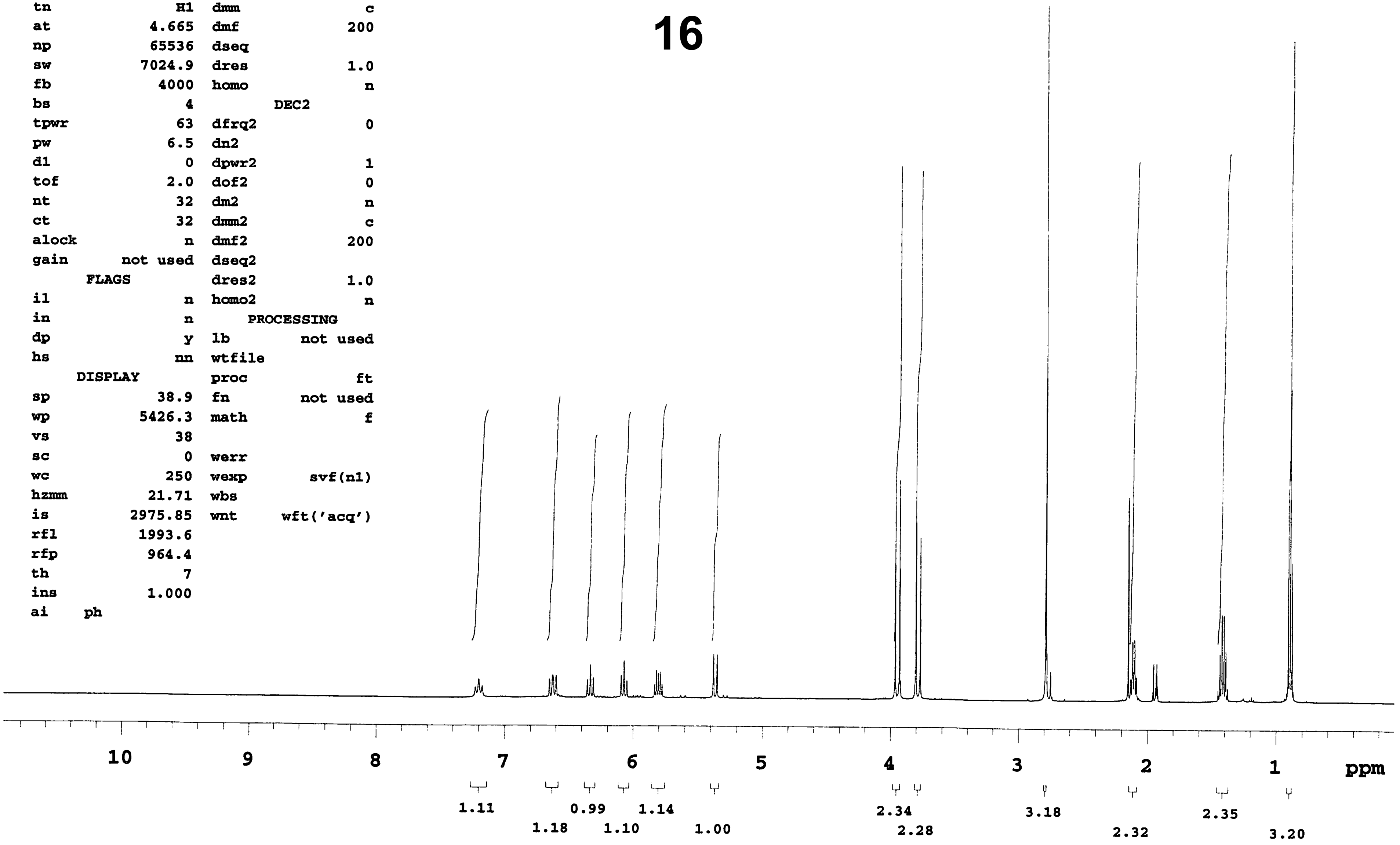

LSJV-76-13C

exp1 s2pul

**SAMPLE**		**DEC. & VT**	
date	Mar 8 2010	dfrq	499.695
solvent	CD3CN	dn	H1
file	exp	dpwr	44
**ACQUISITION**		dof	-827.6
sfrq	125.662	dm	yyy
tn	C13	dmm	w
at	1.086	dmf	19608
np	65536	dseq	
sw	30165.9	dres	90.0
fb	17000	homo	n
bs	16	**DEC2**	
ss	1	dfrq2	0
tpwr	54	dn2	
pw	6.0	dpwr2	1
d1	1.000	dof2	0
tof	1884.7	dm2	n
nt	5000	dmm2	c
ct	2699	dmf2	10000
alock	n	dseq2	
gain	not used	dres2	1.0
**FLAGS**		homo2	n
il	n	**PROCESSING**	
in	n	lb	1.00
dp	y	wtfile	
hs	nn	proc	ft
**DISPLAY**		fn	not used
sp	-1058.1	math	f
wp	28635.0		
vs	83	werr	
sc	0	wexp	
wc	250	wbs	
hzmm	114.54	wnt	
is	500.00		
rfl	16036.7		
rfp	14851.6		
th	68		
ins	100.000		
nm ph			

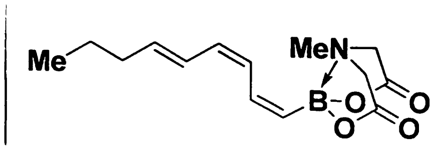

16

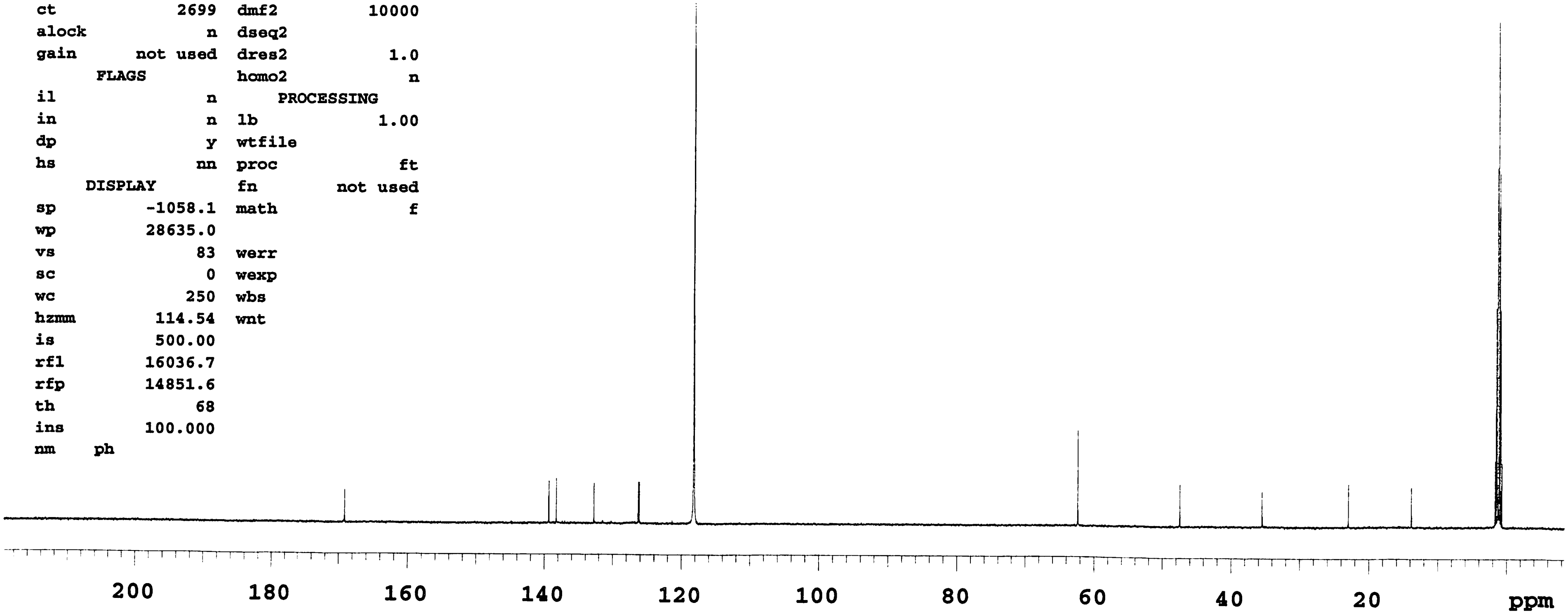

LSJV-56

exp1 s2pul

SAMPLE		DEC. & VT	
date	Jan 21 2010	dfrq	499.696
solvent	CD3CN	dn	H1
file	exp	dpwr	20
ACQUISITION		dof	0
sfrq	499.696	dm	nnn
tn	H1	dmm	c
at	4.665	dmf	200
np	65536	dseq	
sw	7024.9	dres	1.0
fb	4000	homo	n
bs	4	DEC2	
tpwr	63	dfrq2	0
pw	6.5	dn2	
d1	0	dpwr2	1
tof	2.0	dof2	0
nt	32	dm2	n
ct	32	dmm2	c
alock	n	dmf2	200
gain	not used	dseq2	
FLAGS		dres2	1.0
il	n	homo2	n
in	n	PROCESSING	
dp	y	lb	not used
hs	nn	wtfile	
DISPLAY		proc	ft
sp	9.3	fn	not used
wp	5456.7	math	f
vs	25		
sc	0	werr	
wc	250	wexp	svf(n1)
hzmm	21.83	wbs	
is	1645.03	wnt	wft('acq')
rfl	1993.6		
rfp	964.4		
th	15		
ins	1.000		
ai	ph		

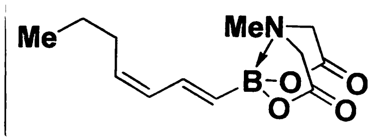

17

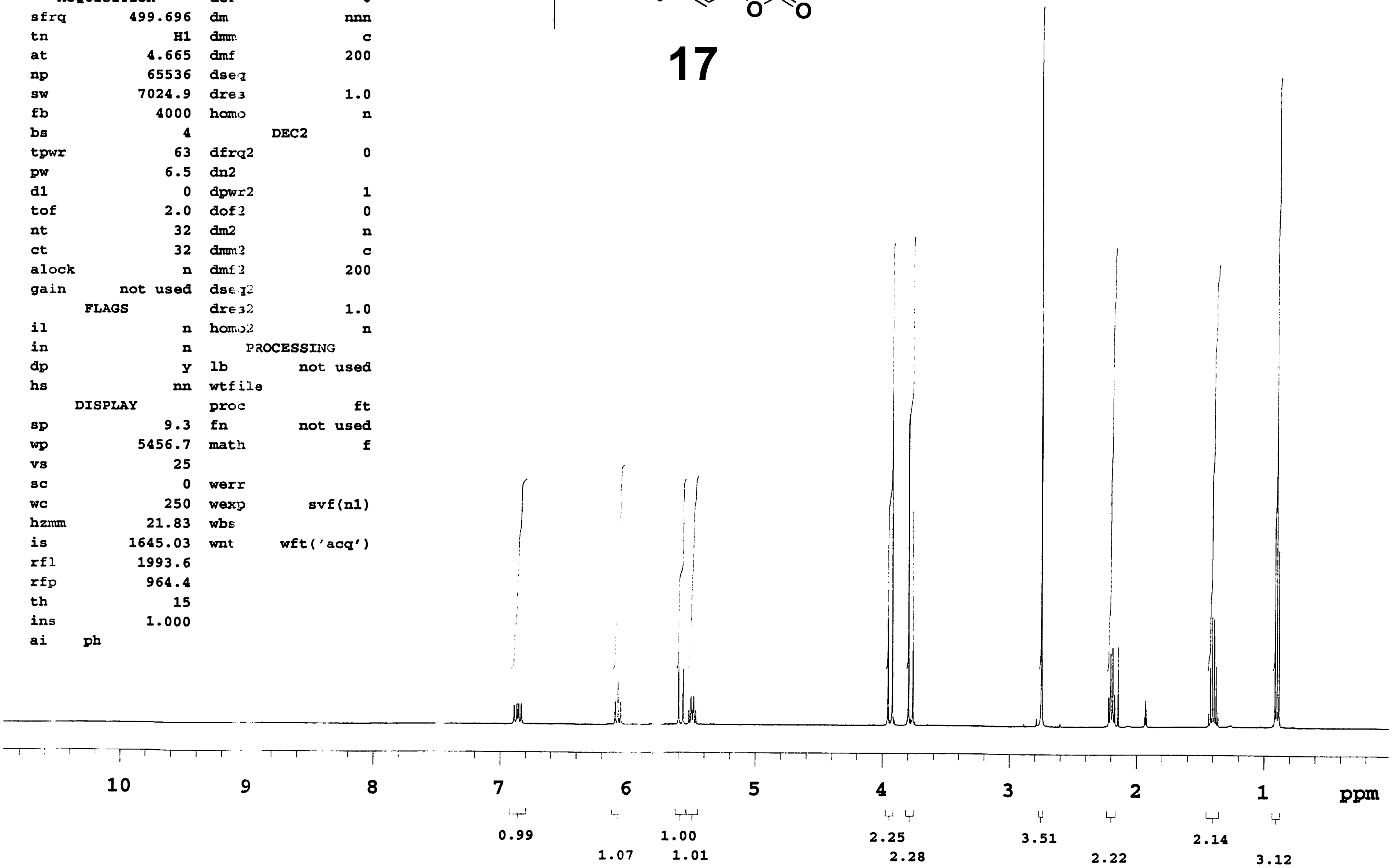

LSJV-56-13C

exp1 s2pul

SAMPLE		DEC. & VT	
date	Jan 21 2010	dfrq	499.695
solvent	CD3CN	dn	H1
file	exp	dpwr	44
ACQUISITION		dof	-827.6
sfrq	125.662	dm	yyy
tn	C13	dmm	w
at	1.086	dmf	19608
np	65536	dseq	
sw	30165.9	dres	90.0
fb	17000	homo	n
bs	16	DEC2	
ss	1	dfrq2	0
tpwr	54	dn2	
pw	6.0	dpwr2	1
d1	1.000	dof2	0
tof	1884.7	dm2	n
nt	1500	dmm2	c
ct	1500	dmf2	10000
alock	n	dseq2	
gain	not used	dres2	1.0
FLAGS		homo2	n
il	n	PROCESSING	
in	n	lb	1.00
dp	y	wtfile	
hs	nn	proc	ft
DISPLAY		fn	not used
sp	-1098.6	math	f
wp	28635.9		
vs	108	werr	
sc	0	wexp	
wc	250	wbs	
hzmm	114.54	wnt	
is	500.00		
rfl	16035.8		
rfp	14851.6		
th	68		
ins	100.000		
nm	ph		

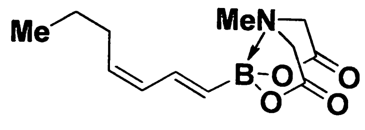

17

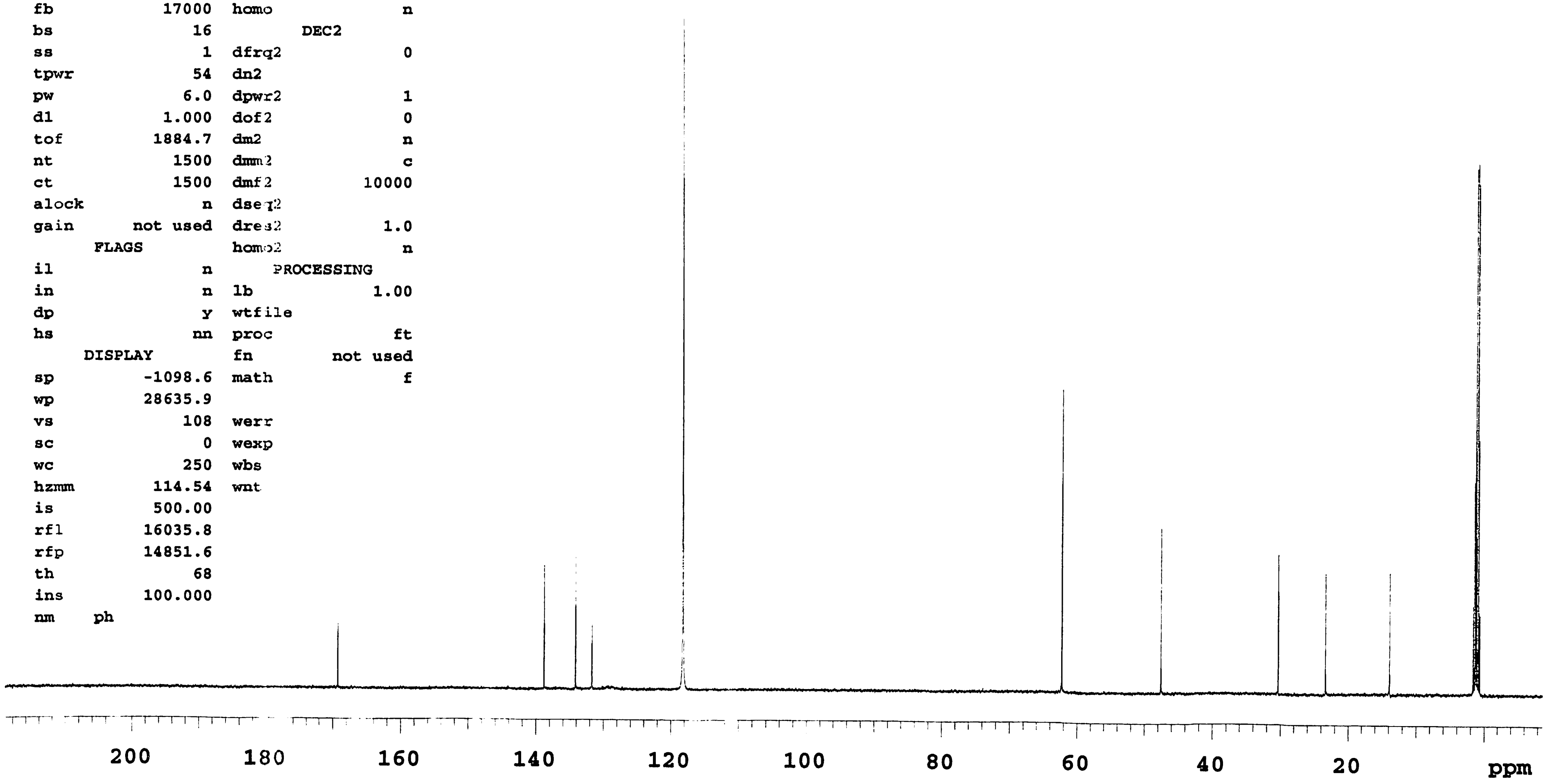

LSJV-58

exp1 s2pul

**SAMPLE**		**DEC. & VT**	
date	Jan 23 2010	dfrq	499.696
solvent	CD3CN	dn	H1
file	exp	dpwr	20
**ACQUISITION**		dof	0
sfrq	499.696	dm	nnn
tn	H1	dmm	c
at	4.665	dmf	200
np	65536	dseq	
sw	7024.9	dres	1.0
fb	4000	homo	n
bs	4	**DEC2**	
tpwr	63	dfrq2	0
pw	6.5	dn2	
d1	0	dpwr2	1
tof	2.0	dof2	0
nt	32	dm2	n
ct	32	dmm2	c
alock	n	dmf2	200
gain	not used	dseq2	
**FLAGS**		dres2	1.0
il	n	homo2	n
in	n	**PROCESSING**	
dp	y	lb	not used
hs	nn	wtfile	
**DISPLAY**		proc	ft
sp	9.3	fn	not used
wp	5446.6	math	f
vs	45		
sc	0	werr	
wc	250	wexp	svf(n1)
hzmm	21.79	wbs	
is	2659.85	wnt	wft('acq')
rfl	1993.6		
rfp	964.4		
th	7		
ins	1.000		
ai	ph		

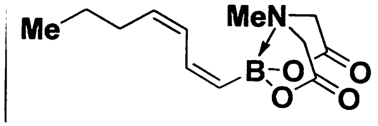

18

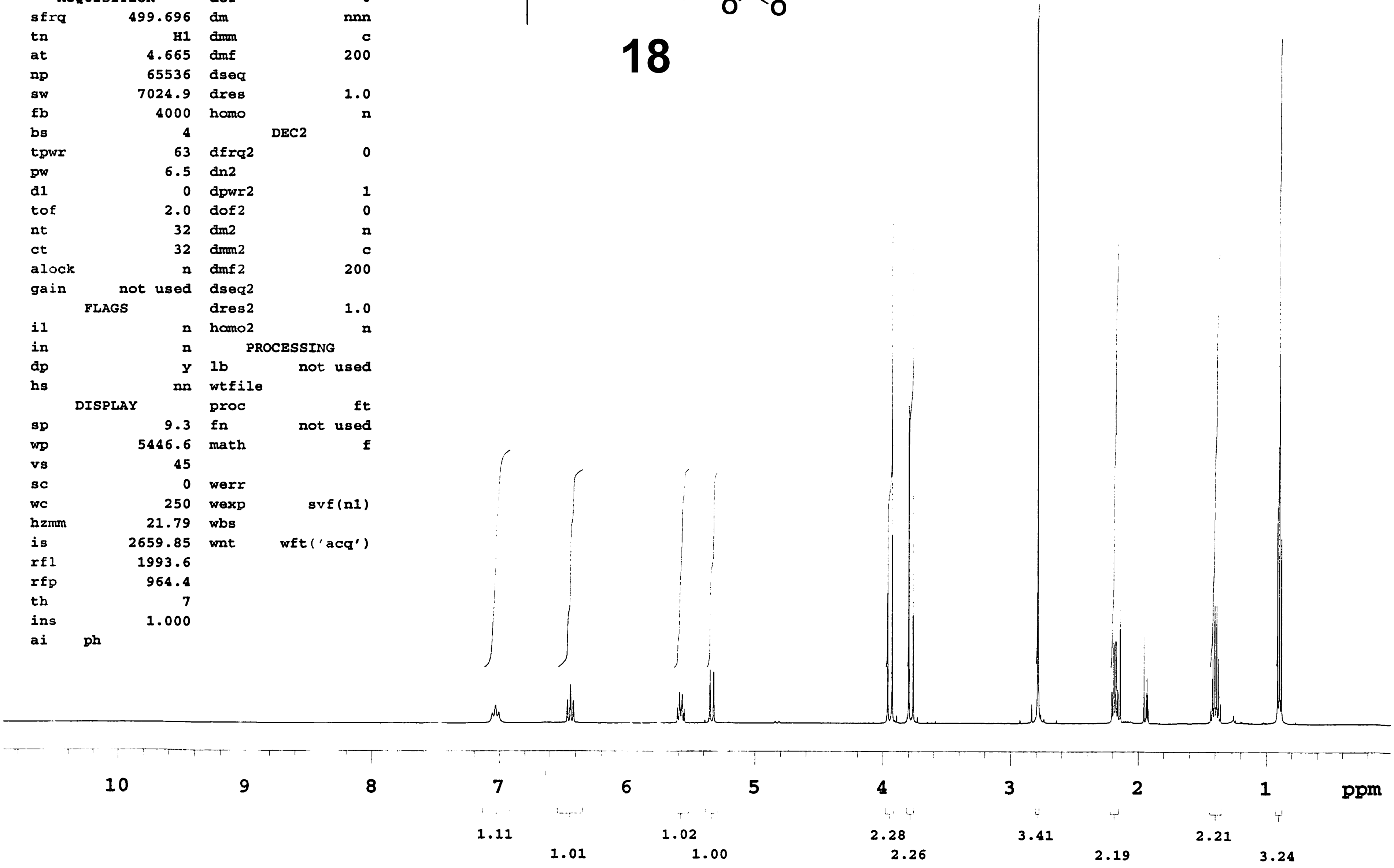

LSJV-58-13C

exp1 s2pul

SAMPLE		DEC. & VT	
date	Jan 23 2010	dfrq	499.695
solvent	CD3CN	dn	H1
file	exp	dpwr	44
ACQUISITION		dof	-827.6
sfrq	125.662	dm	yyy
tn	C13	dmm	w
at	1.086	dmf	19608
np	65536	dseq	
sw	30165.9	dres	90.0
fb	17000	homo	n
bs	16	DEC2	
ss	1	dfrq2	0
tpwr	54	dn2	
pw	6.0	dpwr2	1
d1	1.000	dof2	0
tof	1884.7	dm2	n
nt	3000	dmm2	c
ct	2010	dmf2	10000
alock	n	dseq2	
gain	not used	dres2	1.0
FLAGS		homo2	n
il	n	PROCESSING	
in	n	lb	1.00
dp	y	wtfile	
hs	nn	proc	ft
DISPLAY		fn	not used
sp	-1140.9	math	f
wp	28635.9		
vs	79	werr	
sc	0	wexp	
wc	250	wbs	
hzmm	114.54	wnt	
is	500.00		
rfl	16034.8		
rfp	14851.6		
th	5		
ins	100.000		
nm	ph		

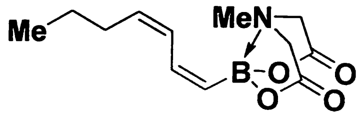

18

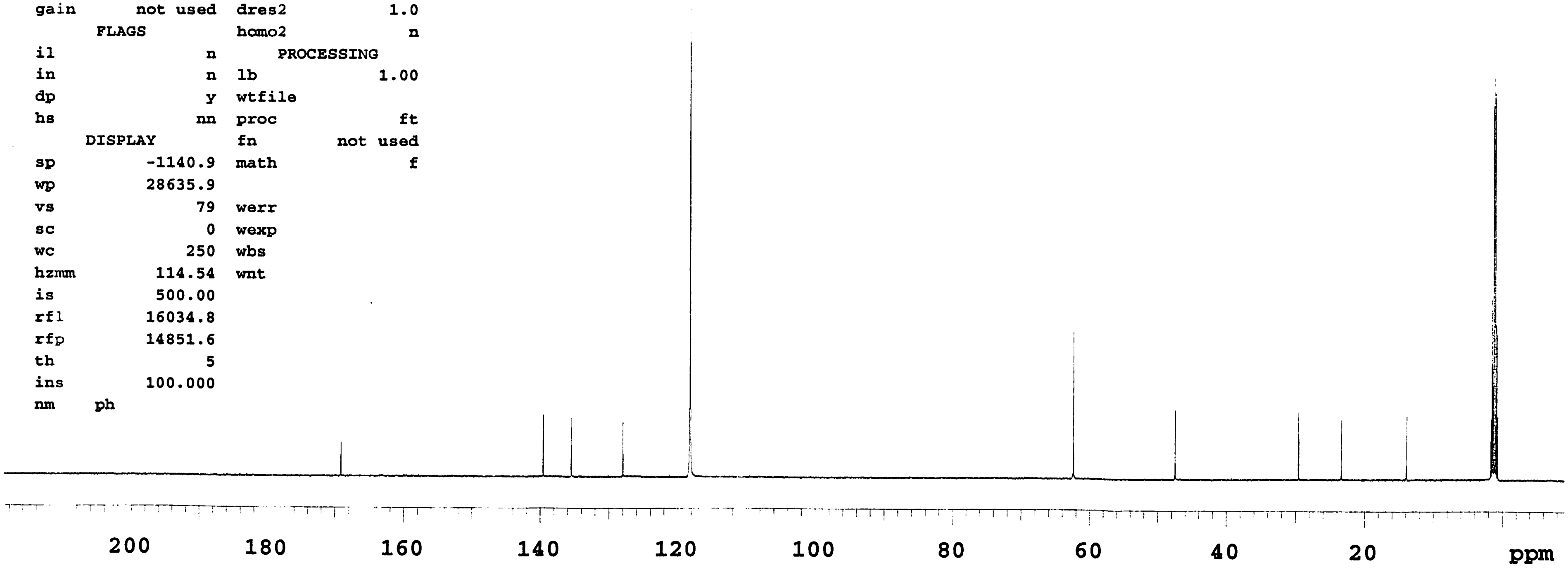

LSJV-63

exp1 s2pul

**SAMPLE**		**DEC. & VT**	
date	Jan 30 2010	dfrq	499.696
solvent	CD3CN	dn	H1
file	exp	dpwr	20
**ACQUISITION**		dof	0
sfrq	499.696	dm	nnn
tn	H1	dmm	c
at	4.665	dmf	200
np	65536	dseq	
sw	7024.9	dres	1.0
fb	4000	homo	n
bs	4	**DEC2**	
tpwr	63	dfrq2	0
pw	6.5	dn2	
d1	0	dpwr2	1
tof	2.0	dof2	0
nt	32	dm2	n
ct	32	dmm2	c
alock	n	dmf2	200
gain	not used	dseq2	
**FLAGS**		dres2	1.0
il	n	homo2	n
in	n	**PROCESSING**	
dp	y	lb	not used
hs	nn	wtfile	
**DISPLAY**		proc	ft
sp	18.8	fn	not used
wp	5456.7	math	f
vs	38		
sc	0	werr	
wc	250	wexp	svf(nl)
hzmm	21.83	wbs	
is	2178.03	wnt	wft('acq')
rfl	1993.6		
rfp	964.4		
th	15		
ins	1.000		
ai	ph		

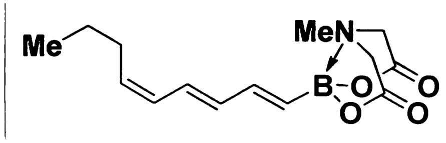

19

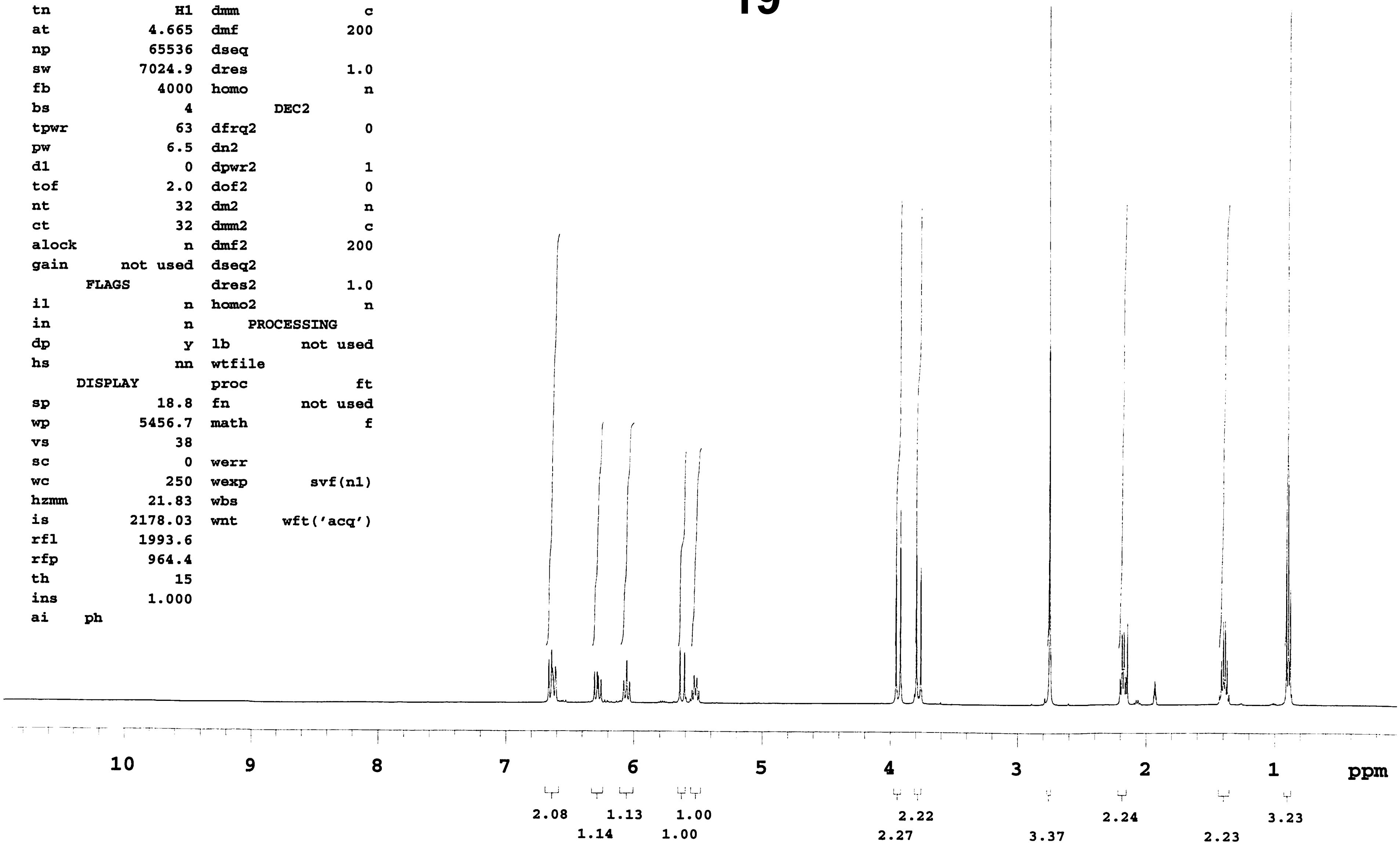

LSJV-63-13C

exp1 s2pul

SAMPLE		DEC. & VT	
date	Jan 30 2010	dfrq	499.695
solvent	CD3CN	dn	H1
file	exp	dpwr	44
ACQUISITION		dof	-827.6
sfrq	125.662	dm	yyy
tn	C13	dmm	w
at	1.086	dmf	19608
np	65536	dseq	
sw	30165.9	dres	90.0
fb	17000	homo	n
bs	16	DEC2	
ss	1	dfrq2	0
tpwr	54	dn2	
pw	6.0	dpwr2	1
d1	1.000	dof2	0
tof	1884.7	dm2	n
nt	3000	dmm2	c
ct	1867	dmf2	10000
alock	n	dseq2	
gain	not used	dres2	1.0
FLAGS		homo2	n
il	n	PROCESSING	
in	n	lb	1.00
dp	y	wtfile	
hs	nn	proc	ft
DISPLAY		fn	not used
sp	-1058.1	math	f
wp	28468.3		
vs	87	werr	
sc	0	wexp	
wc	250	wbs	
hzmm	113.87	wnt	
is	500.00		
rfl	16035.8		
rfp	14851.6		
th	68		
ins	100.000		
nm ph			

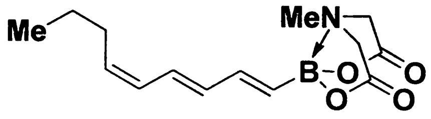

19

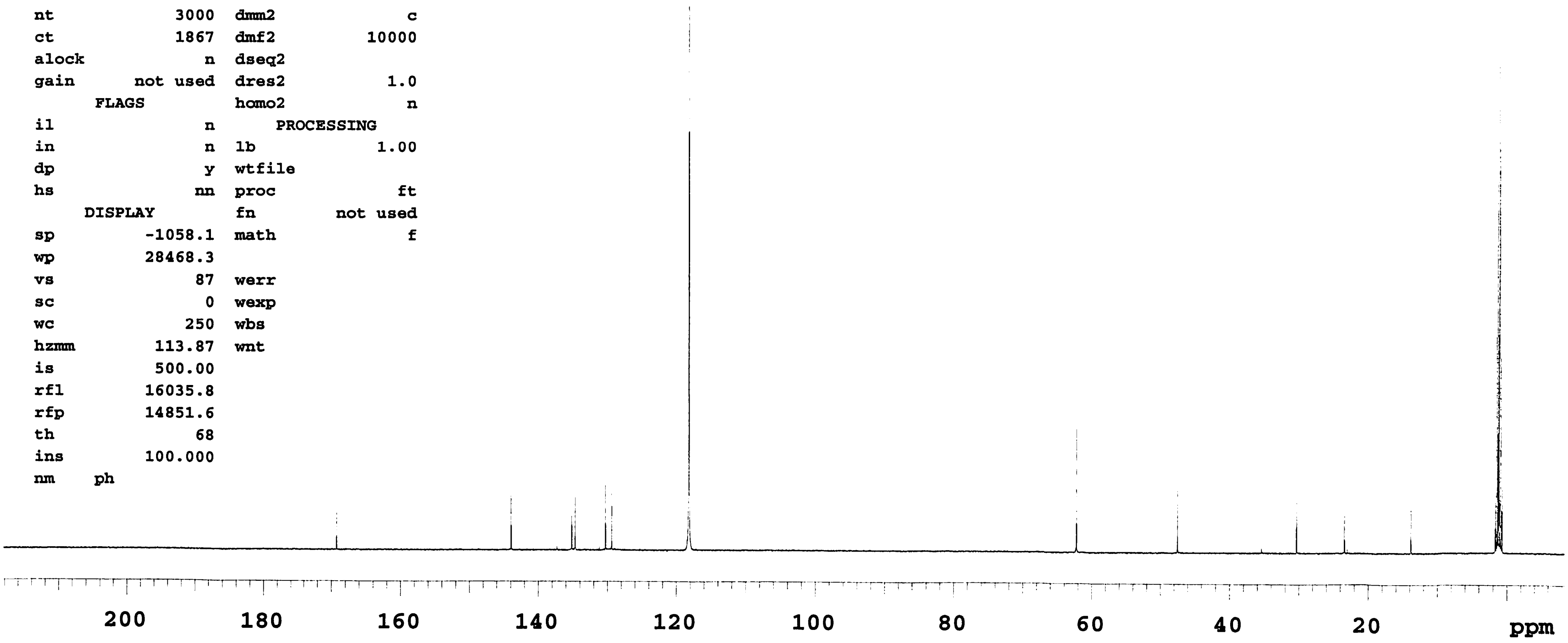

LSJV-67

exp1 s2pul

SAMPLE		DEC. & VT	
date	Feb 15 2010	dfrq	499.696
solvent	CD3CN	dn	H1
file	exp	dpwr	20
ACQUISITION		dof	0
sfrq	499.696	dm	nnn
tn	H1	dmm	c
at	4.665	dmf	200
np	65536	dseq	
sw	7024.9	dres	1.0
fb	4000	homo	n
bs	4	DEC2	
tpwr	63	dfrq2	0
pw	6.5	dn2	
d1	0	dpwr2	1
tof	2.0	dof2	0
nt	32	dm2	n
ct	32	dmm2	c
alock	n	dmf2	200
gain	not used	dseq2	
FLAGS		dres2	1.0
il	n	homo2	n
in	n	PROCESSING	
dp	y	lb	not used
hs	nn	wtfile	
DISPLAY		proc	ft
sp	30.3	fn	not used
wp	5440.6	math	f
vs	48		
sc	0	werr	
wc	250	wexp	svf(n1)
hzmm	21.76	wbs	
is	2243.74	wnt	wft('acq')
rfl	1993.6		
rfp	964.4		
th	7		
ins	1.000		
ai	ph		

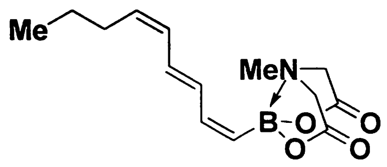

20

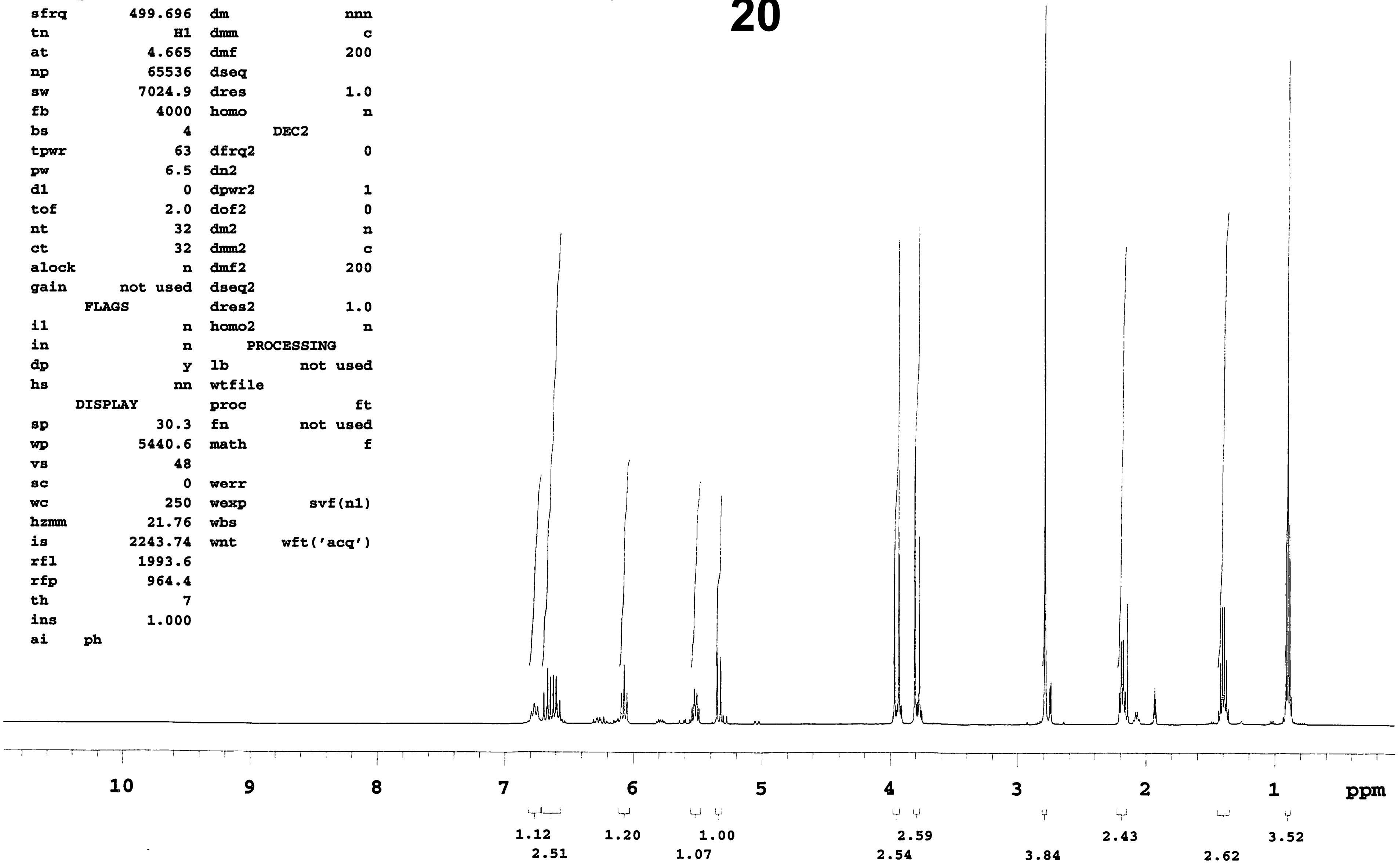

LSJV-67-13C

exp1 s2pul

SAMPLE		DEC. & VT	
date	Feb 15 2010	dfrq	499.695
solvent	CD3CN	dn	H1
file	exp	dpwr	44
ACQUISITION		dof	-827.6
sfrq	125.662	dm	yyy
tn	C13	dmm	w
at	1.086	dmf	19608
np	65536	dseq	
sw	30165.9	dres	90.0
fb	17000	homo	n
bs	16	DEC2	
ss	1	dfrq2	0
tpwr	54	dn2	
pw	6.0	dpwr2	1
d1	1.000	dof2	0
tof	1884.7	dm2	n
nt	5000	dmm2	c
ct	1679	dmf2	10000
alock	n	dseq2	
gain	not used	dres2	1.0
FLAGS		homo2	n
il	n	PROCESSING	
in	n	lb	1.00
dp	y	wtfile	
hs	nn	proc	ft
DISPLAY		fn	not used
sp	-1097.7	math	f
wp	28664.4		
vs	83	werr	
sc	0	wexp	
wc	250	wbs	
hzmm	114.66	wnt	
is	500.00		
rfl	16034.8		
rfp	14851.6		
th	68		
ins	100.000		
nm	ph		

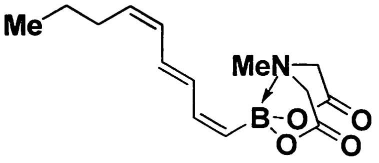

20

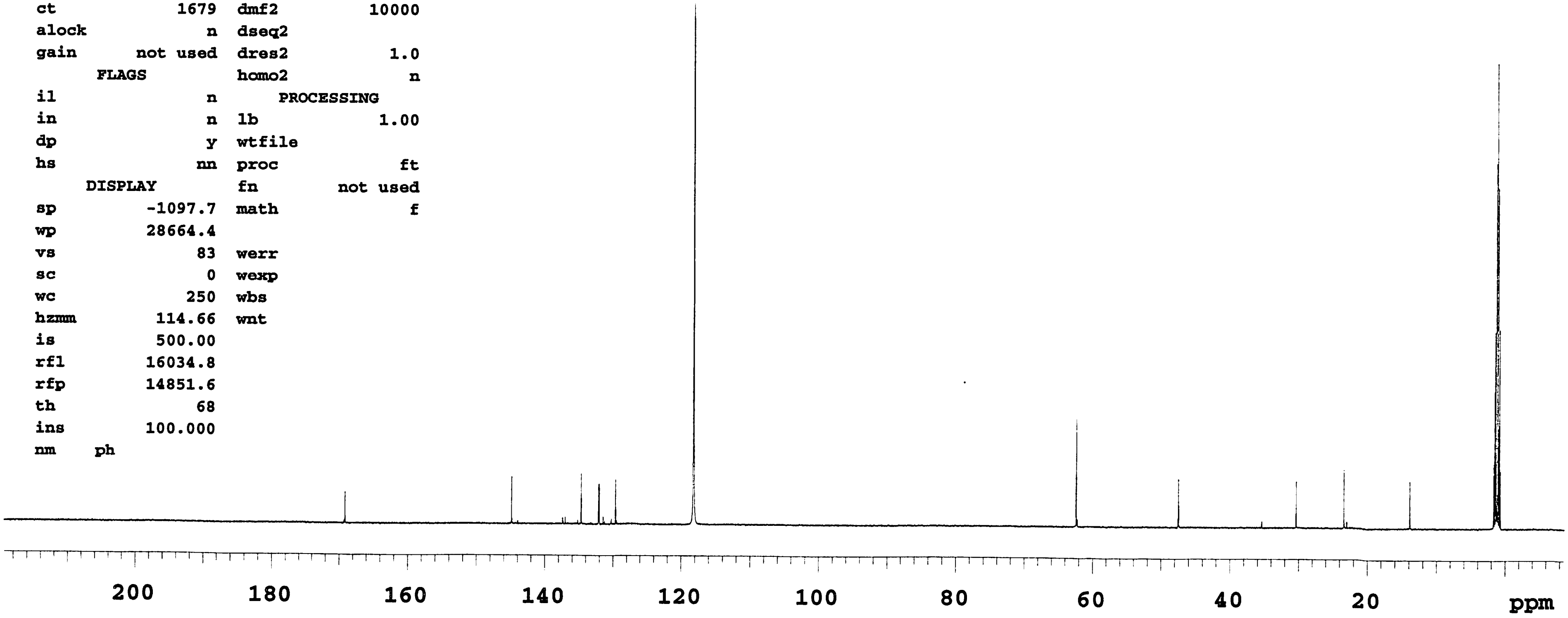

LSJV-71

exp1 s2pul

SAMPLE		DEC. & VT	
date	Feb 21 2010	dfrq	499.696
solvent	CD3CN	dn	H1
file	exp	dpwr	20
ACQUISITION		dof	0
sfrq	499.696	dm	nnn
tn	H1	dmm	c
at	4.665	dmf	200
np	65536	dseq	
sw	7024.9	dres	1.0
fb	4000	homo	n
bs	4	DEC2	
tpwr	63	dfrq2	0
pw	6.5	dn2	
d1	0	dpwr2	1
tof	2.0	dof2	0
nt	32	dm2	n
ct	32	dmm2	c
alock	n	dmf2	200
gain	not used	dseq2	
FLAGS		dres2	1.0
il	n	homo2	n
in	n	PROCESSING	
dp	y	lb	not used
hs	nn	wtfile	
DISPLAY		proc	ft
sp	10.0	fn	not used
wp	5461.2	math	f
vs	26		
sc	0	werr	
wc	250	wexp	svf(n1)
hzmm	21.84	wbs	
is	1878.50	wnt	wft('acq')
rfl	1993.3		
rfp	964.4		
th	12		
ins	1.000		
ai	ph		

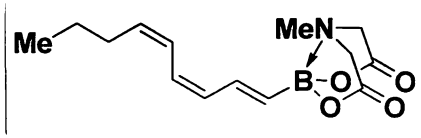

21

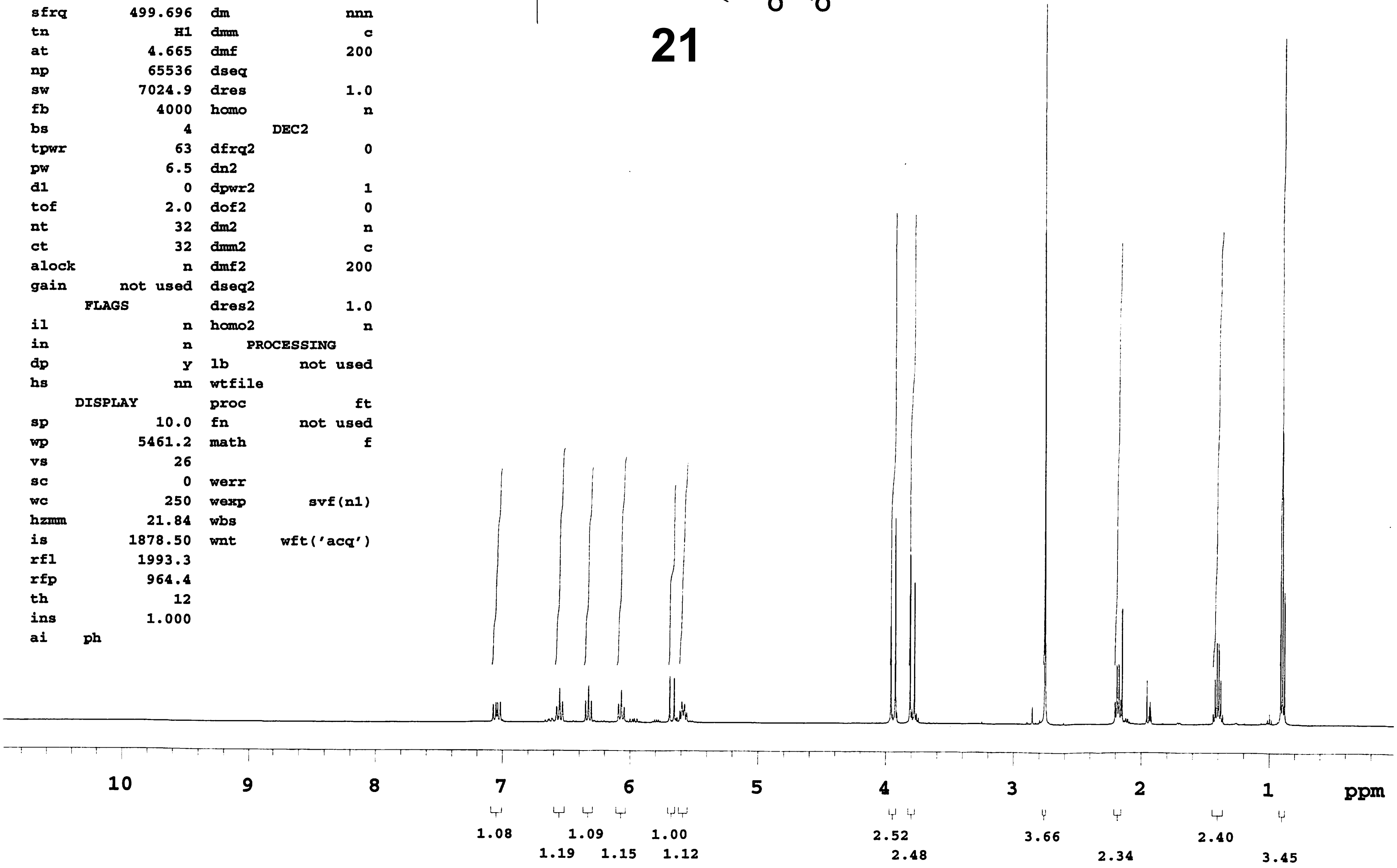

LSJV-71-13C

exp1 s2pul

SAMPLE		DEC. & VT	
date	Feb 21 2010	dfrq	499.695
solvent	CD3CN	dn	H1
file	exp	dpwr	44
ACQUISITION		dof	-827.6
sfrq	125.662	dm	yyy
tn	C13	dmm	w
at	1.086	dmf	19608
np	65536	dseq	
sw	30165.9	dres	90.0
fb	17000	homo	n
bs	16	DEC2	
ss	1	dfrq2	0
tpwr	54	dn2	
pw	6.0	dpwr2	1
d1	1.000	dof2	0
tof	1884.7	dm2	n
nt	2000	dmm2	c
ct	1854	dmf2	10000
alock	n	dseq2	
gain	not used	dres2	1.0
FLAGS		homo2	n
il	n	PROCESSING	
in	n	lb	1.00
dp	y	wtfile	
hs	nn	proc	ft
DISPLAY		fn	not used
sp	-1097.7	math	f
wp	28708.6		
vs	77	werr	
sc	0	wexp	
wc	250	wbs	
hzmm	114.83	wnt	
is	500.00		
rfl	16034.8		
rfp	14851.6		
th	5		
ins	100.000		
nm	ph		

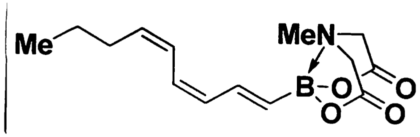

21

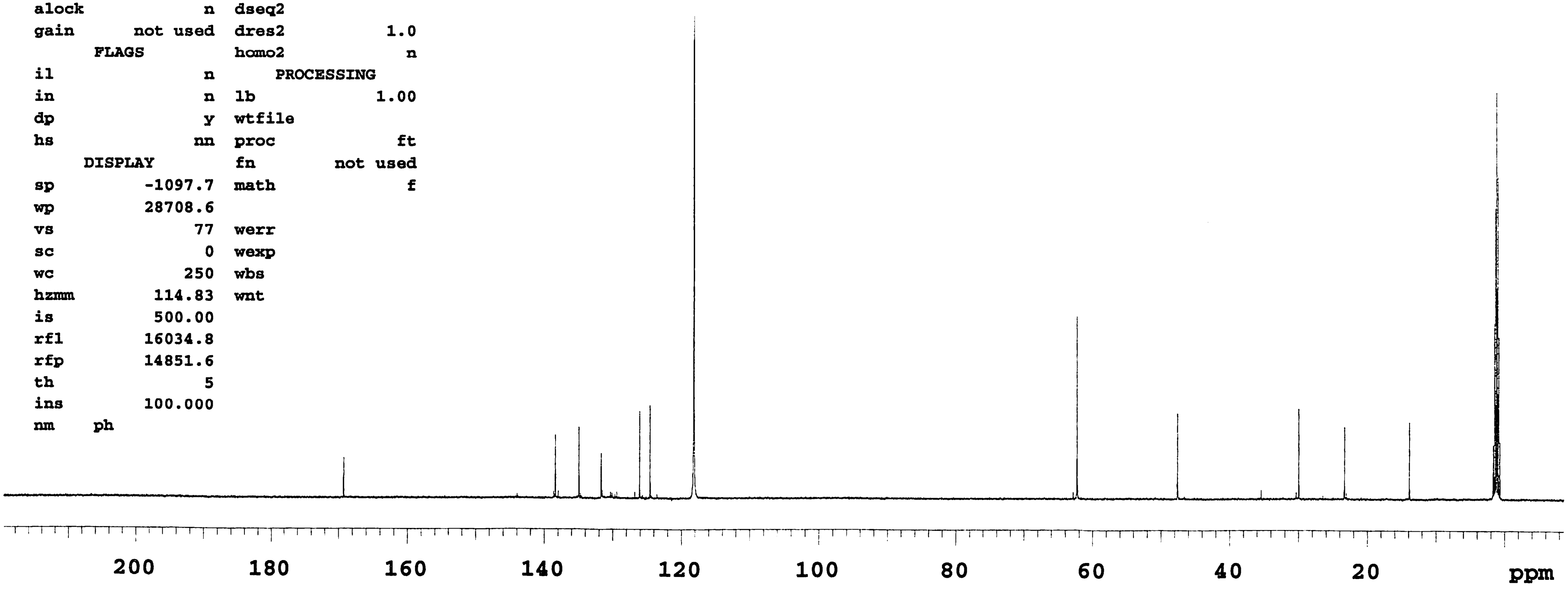

LSJV-77

exp1 s2pul

SAMPLE		DEC. & VT	
date	Mar 8 2010	dfrq	499.696
solvent	CD3CN	dn	H1
file	exp	dpwr	20
ACQUISITION		dof	0
sfrq	499.696	dm	nnn
tn	H1	dmm	c
at	4.665	dmf	200
np	65536	dseq	
sw	7024.9	dres	1.0
fb	4000	homo	n
bs	4	DEC2	
tpwr	63	dfrq2	0
pw	6.5	dn2	
d1	0	dpwr2	1
tof	2.0	dof2	0
nt	32	dm2	n
ct	32	dmm2	c
alock	n	dmf2	200
gain	not used	dseq2	
FLAGS		dres2	1.0
il	n	homo2	n
in	n	PROCESSING	
dp	y	lb	not used
hs	nn	wtfile	
DISPLAY		proc	ft
sp	46.6	fn	not used
wp	5428.4	math	f
vs	36		
sc	0	werr	
wc	250	wexp	svf(n1)
hzmm	21.71	wbs	
is	2957.42	wnt	wft('acq')
rfl	1993.6		
rfp	964.4		
th	7		
ins	1.000		
ai	ph		

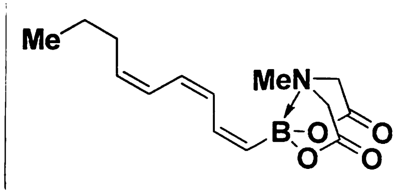

22

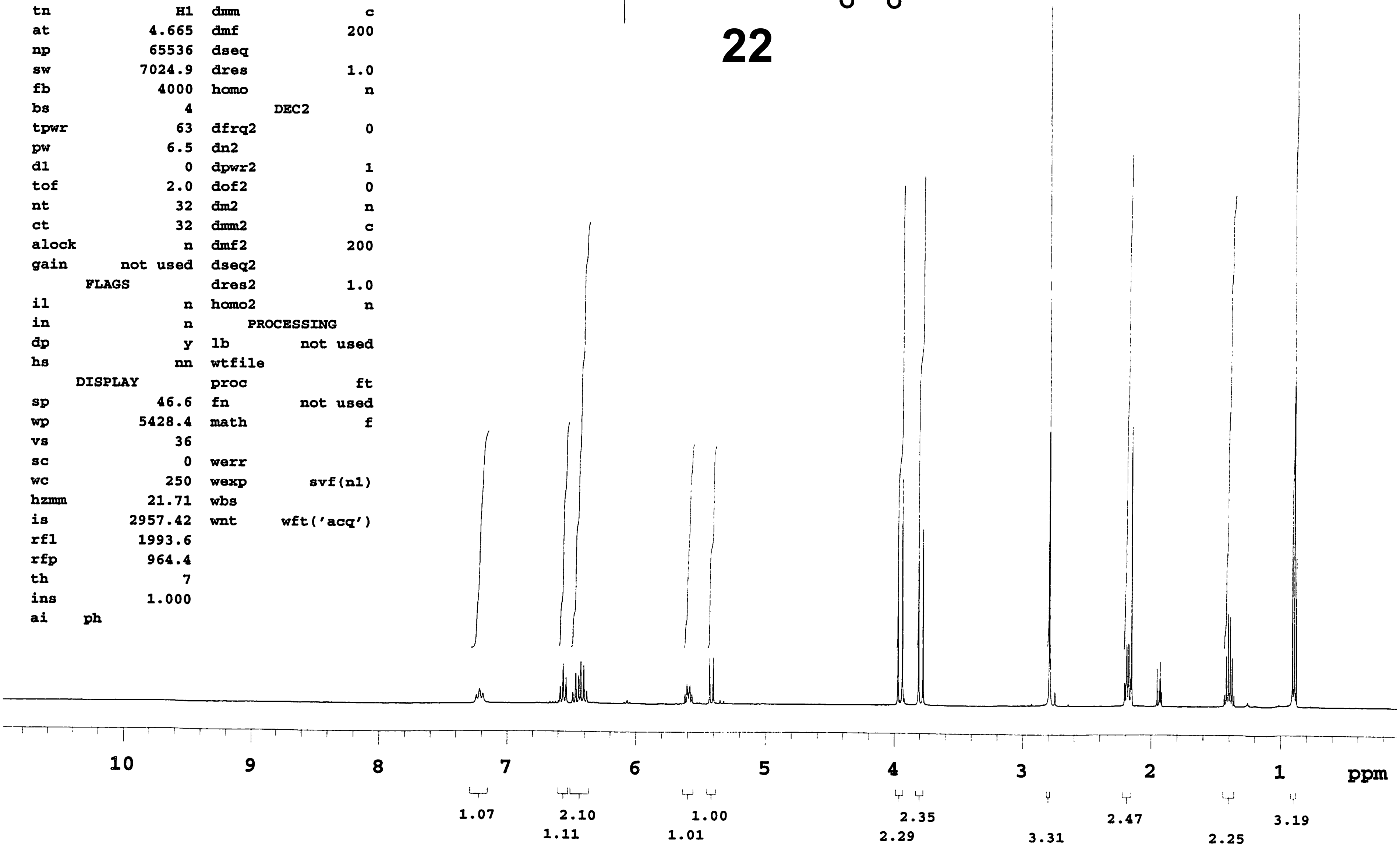

```
LSJV-77-13C

exp1  s2pul

      SAMPLE              DEC. & VT
date    Mar  8 2010  dfrq      499.695
solvent       CD3CN  dn             H1
file            exp  dpwr           44
   ACQUISITION       dof        -827.6
sfrq        125.662  dm            yyy
tn              C13  dmm             w
at            1.086  dmf         19608
np            65536  dseq
sw          30165.9  dres         90.0
fb            17000  homo            n
bs               16          DEC2
ss                1  dfrq2           0
tpwr             54  dn2
pw              6.0  dpwr2           1
d1            1.000  dof2            0
tof          1884.7  dm2             n
nt             5000  dmm2            c
ct             2509  dmf2        10000
alock             n  dseq2
gain       not used  dres2         1.0
      FLAGS          homo2           n
il                n      PROCESSING
in                n  lb           1.00
dp                y  wtfile
hs               nn  proc           ft
     DISPLAY         fn       not used
sp          -1099.5  math            f
wp          28634.0
vs               80  werr
sc                0  wexp
wc              250  wbs
hzmm         114.54  wnt
is           500.00
rfl         16036.7
rfp         14851.6
th               68
ins         100.000
nm    ph
```

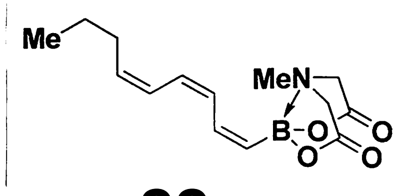

22

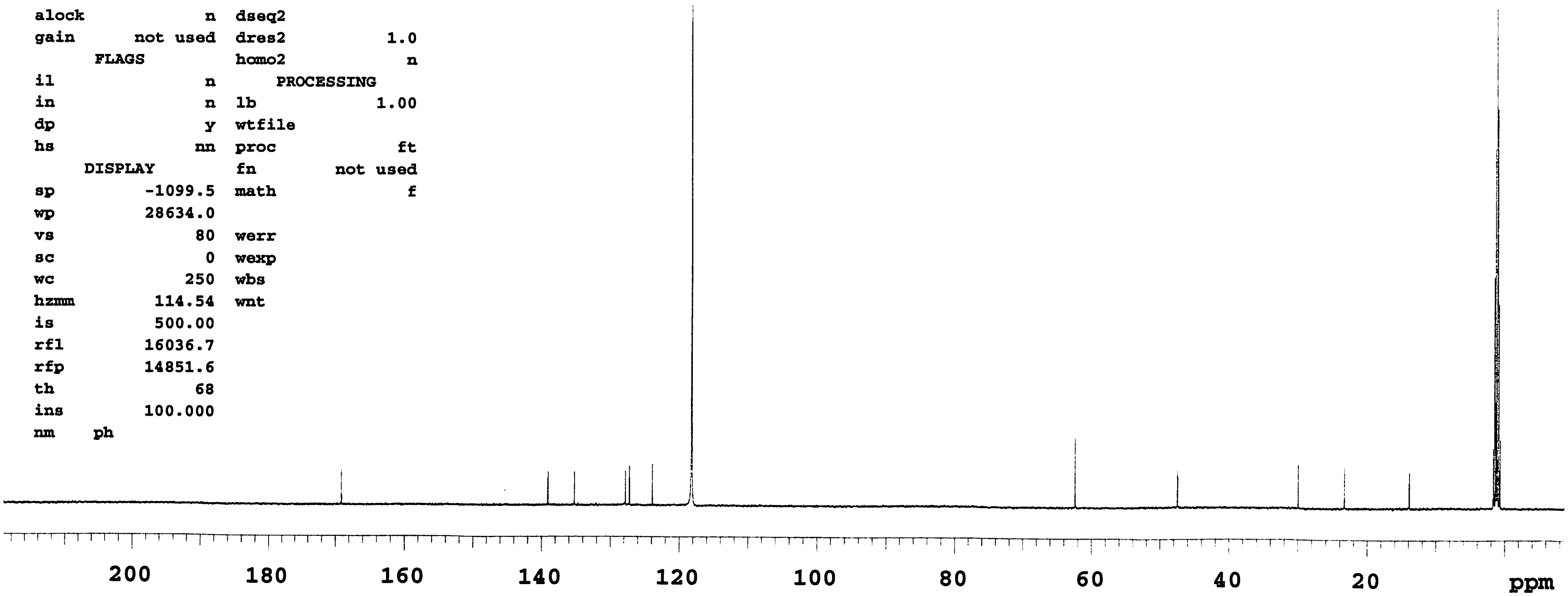

LSJV-91

exp1 s2pul

SAMPLE		DEC. & VT	
date	May 28 2010	dfrq	499.696
solvent	CD3CN	dn	H1
file	exp	dpwr	20
ACQUISITION		dof	0
sfrq	499.696	dm	nnn
tn	H1	dmm	c
at	4.665	dmf	200
np	65536	dseq	
sw	7024.9	dres	1.0
fb	4000	homo	n
bs	4	DEC2	
tpwr	63	dfrq2	0
pw	6.5	dn2	
d1	0	dpwr2	1
tof	2.0	dof2	0
nt	128	dm2	n
ct	128	dmm2	c
alock	n	dmf2	200
gain	not used	dseq2	
FLAGS		dres2	1.0
il	n	homo2	n
in	n	PROCESSING	
dp	y	lb	not used
hs	nn	wtfile	
DISPLAY		proc	ft
sp	-0.7	fn	not used
wp	5461.2	math	f
vs	84		
sc	0	werr	
wc	250	wexp	svf(n1)
hzmm	21.84	wbs	
is	3541.69	wnt	wft('acq')
rfl	1993.8		
rfp	964.4		
th	2		
ins	3.000		
ai	ph		

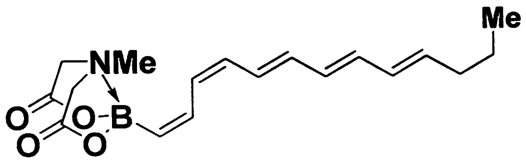

23

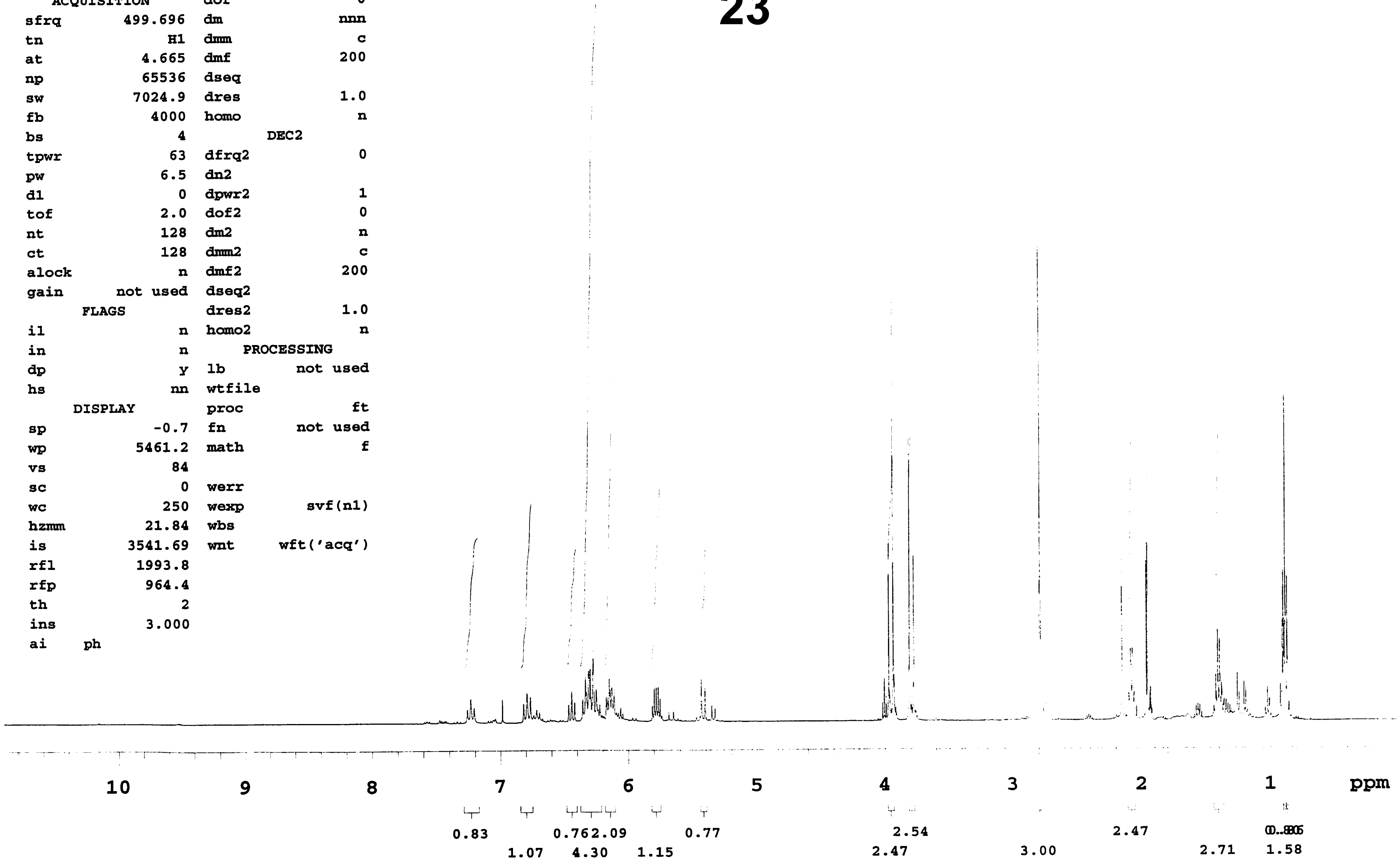

LSJV-91-13C

exp1 s2pul

SAMPLE		DEC. & VT	
date	May 28 2010	dfrq	499.695
solvent	CD3CN	dn	H1
file	exp	dpwr	44
ACQUISITION		dof	-827.6
sfrq	125.662	dm	yyy
tn	C13	dmm	w
at	1.086	dmf	19608
np	65536	dseq	
sw	30165.9	dres	90.0
fb	17000	homo	n
bs	16	DEC2	
ss	1	dfrq2	0
tpwr	54	dn2	
pw	6.0	dpwr2	1
d1	1.000	dof2	0
tof	1884.7	dm2	n
nt	11111	dmm2	c
ct	2556	dmf2	10000
alock	n	dseq2	
gain	not used	dres2	1.0
FLAGS		homo2	n
il	n	PROCESSING	
in	n	lb	1.00
dp	y	wtfile	
hs	nn	proc	ft
DISPLAY		fn	not used
sp	-1138.2	math	f
wp	28706.8		
vs	111	werr	
sc	0	wexp	
wc	250	wbs	
hzmm	114.83	wnt	
is	5359.58		
rfl	16033.9		
rfp	14851.6		
th	1		
ins	100.000		
nm	ph		

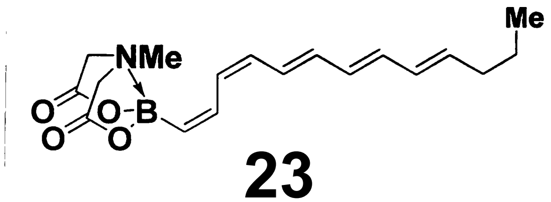

23

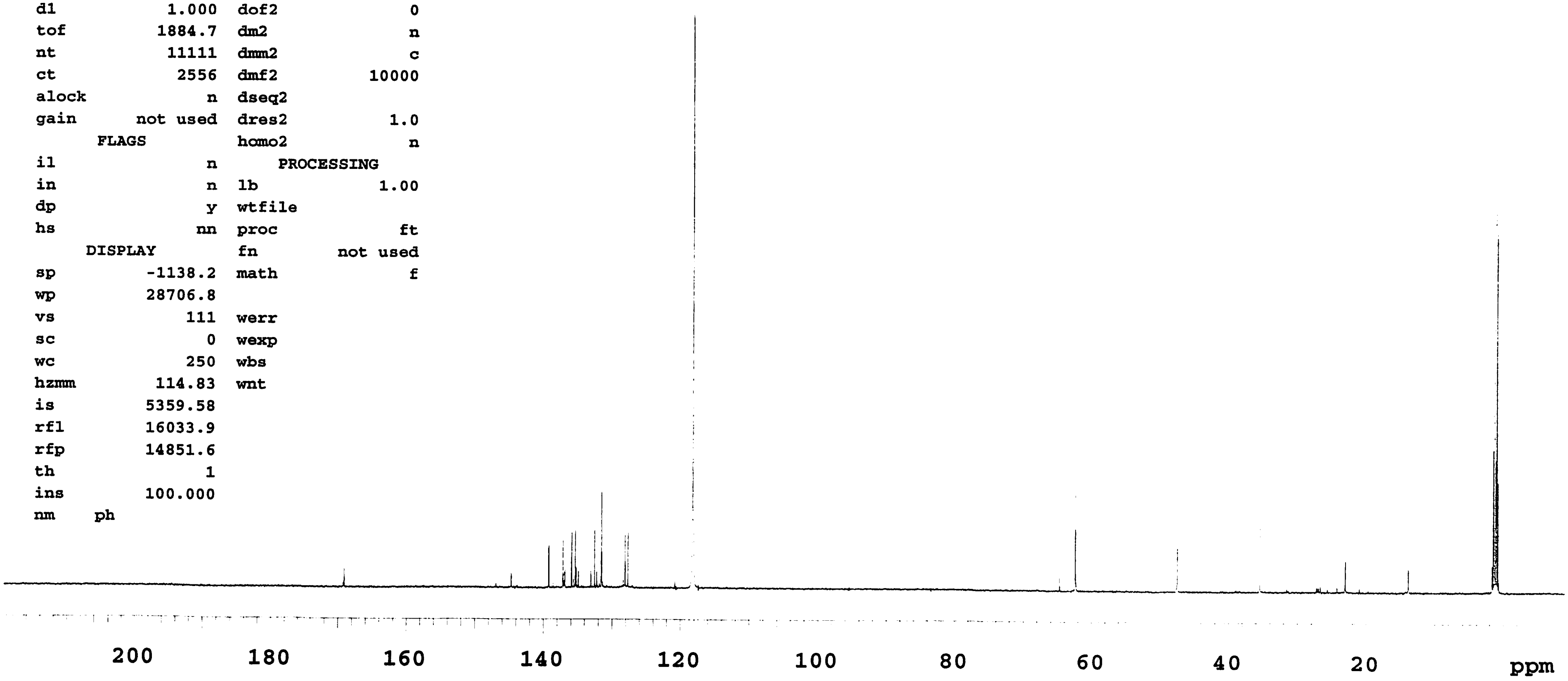

LSJV-80

exp1 std1h

SAMPLE		DEC. & VT	
date	Apr 1 2010	dn	H1
solvent	CDCl3	dof	0
file	exp	dm	nnn
ACQUISITION		dmm	c
sfrq	399.947	dmf	200
tn	H1	dpwr	20
at	4.096	PROCESSING	
np	65536	lb	not used
sw	8000.0	wtfile	
fb	4000	proc	ft
bs	16	fn	not used
tpwr	58	math	f
pw	5.8		
d1	0	werr	
tof	-425.7	wexp	svf(n1)
nt	32	wbs	
ct	32	wnt	wft('acq')
alock	n	DISPLAY	
gain	not used	sp	-371.7
FLAGS		wp	4743.2
il	n	vs	151
in	n	sc	0
dp	y	wc	250
hs	nn	hzmm	18.97
		is	1053.33
		rfl	5320.6
		rfp	2895.6
		th	20
		ins	1.000
		nm	ph

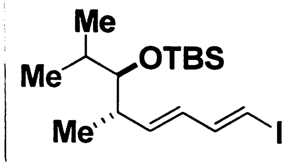

24

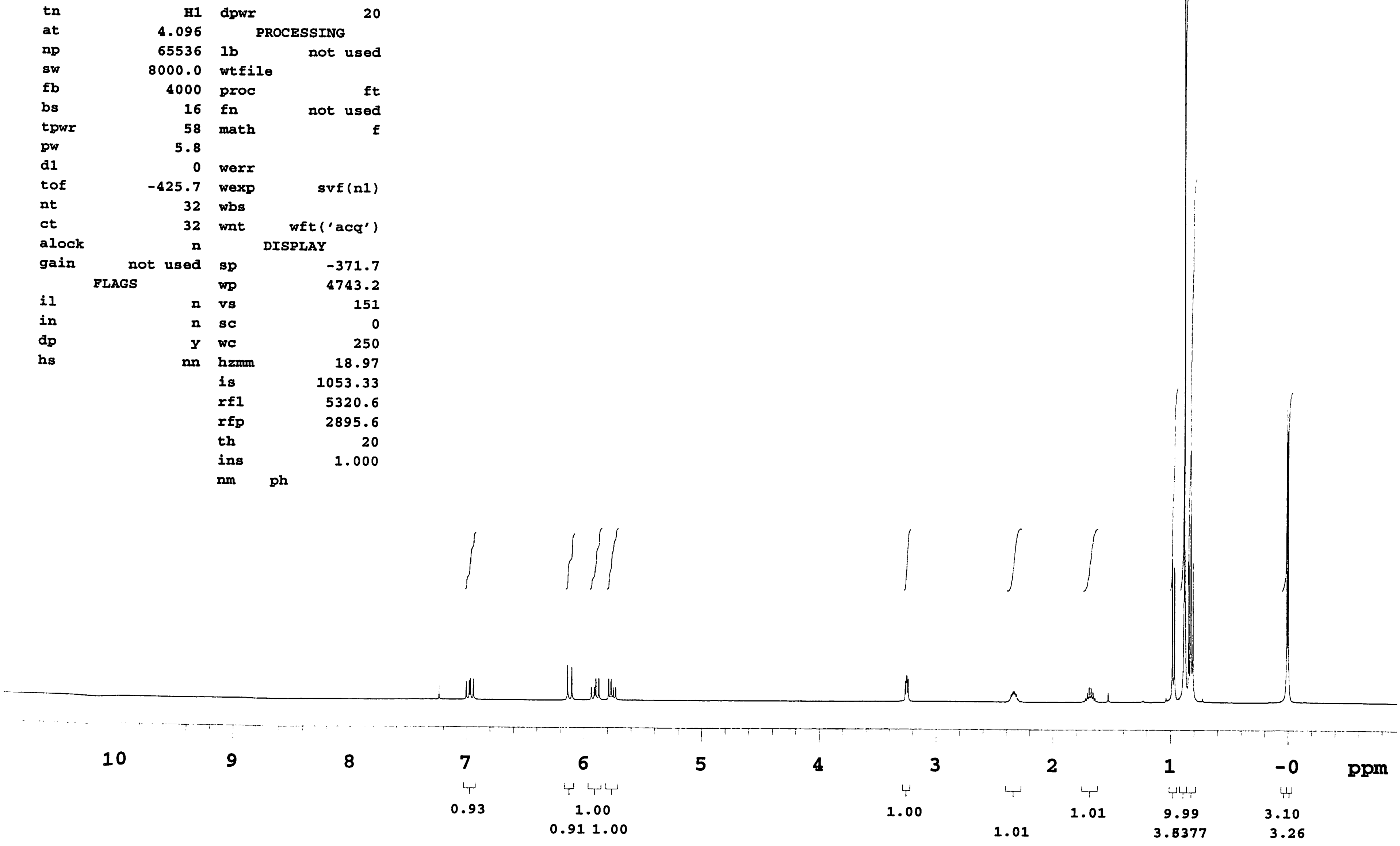

LSJV-80-13C

exp1 std13c

SAMPLE		DEC. & VT	
date	Apr 1 2010	dn	H1
solvent	CDCl3	dof	-1092.3
file	exp	dm	yyy
ACQUISITION		dmm	w
sfrq	100.578	dmf	13889
tn	C13	dpwr	43
at	1.311	PROCESSING	
np	65536	lb	1.00
sw	25000.0	wtfile	
fb	14000	proc	ft
bs	16	fn	not used
tpwr	54	math	f
pw	5.1		
d1	1.000	werr	
tof	1966.4	wexp	
nt	11111	wbs	
ct	2002	wnt	
alock	n	DISPLAY	
gain	not used	sp	-1002.7
FLAGS		wp	23008.0
il	n	vs	100
in	n	sc	0
dp	y	wc	250
hs	nn	hzmm	92.03
		is	500.00
		rfl	8783.7
		rfp	7743.6
		th	20
		ins	100.000
		nm ph	

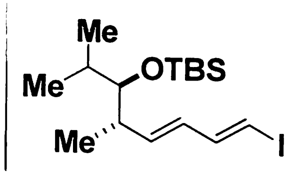

24

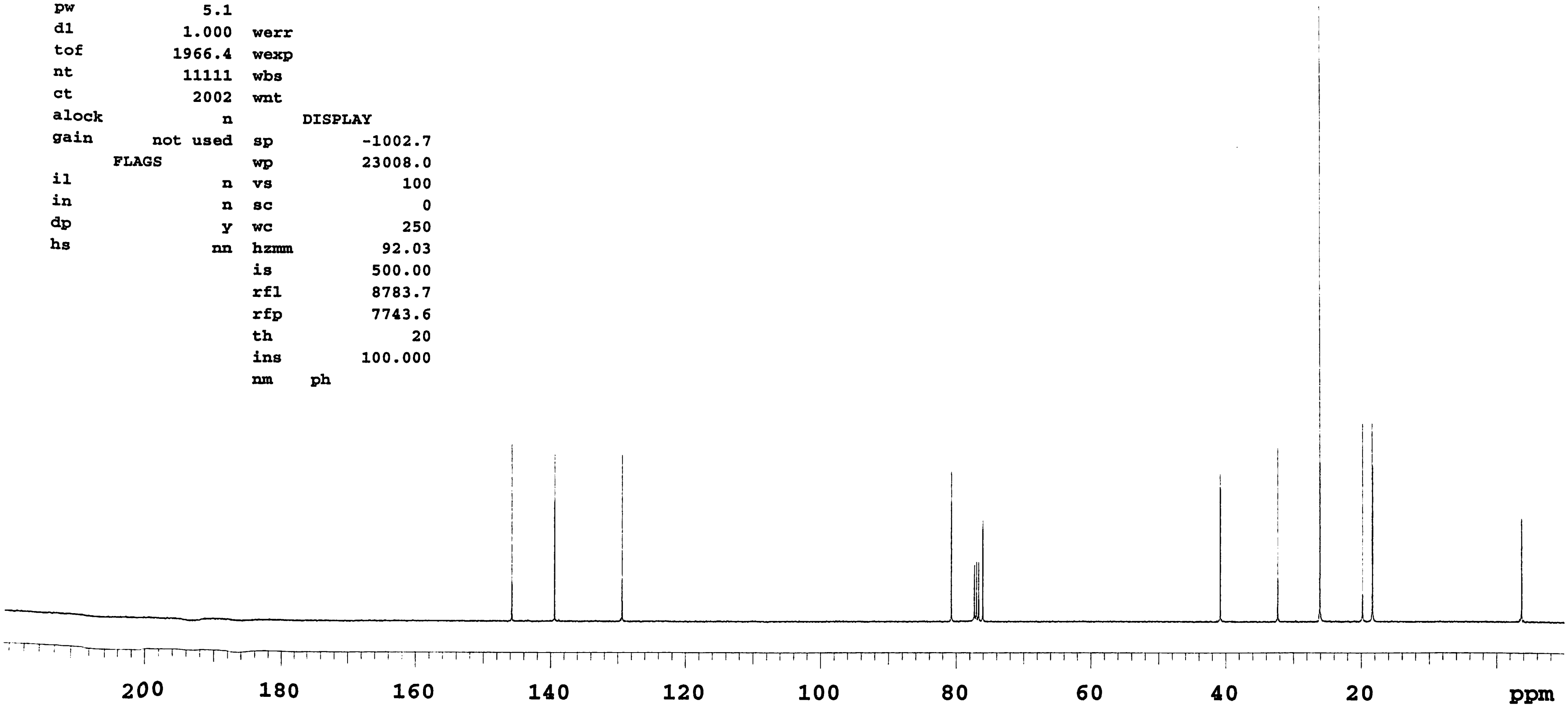

LSJV-94-1H

exp1 s2pul

SAMPLE		DEC. & VT	
date	Jun 6 2010	dfrq	599.765
solvent	Benzene	dn	H1
file	exp	dpwr	20
ACQUISITION		dof	0
sfrq	599.765	dm	nnn
tn	H1	dmm	c
at	4.096	dmf	200
np	71242	dseq	
sw	8696.6	dres	1.0
fb	5000	homo	n
bs	4	temp	25.0
tpwr	63	DEC2	
pw	12.2	dfrq2	0
d1	0	dn2	
tof	103.8	dpwr2	1
nt	16	dof2	0
ct	16	dm2	n
alock	n	dmm2	c
gain	50	dmf2	200
FLAGS		dseq2	
il	n	dres2	1.0
in	n	homo2	n
dp	y	PROCESSING	
hs	nn	wtfile	
DISPLAY		proc	ft
sp	-287.0	fn	131072
wp	7461.3	math	f
vs	32		
sc	0	werr	
wc	250	wexp	svf(n1)
hzmm	29.85	wbs	
is	1192.55	wnt	
rfl	299.8		
rfp	0		
th	115		
ins	1.000		
ai	ph		

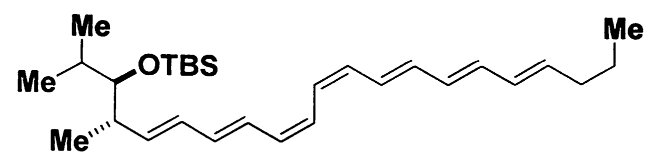

25

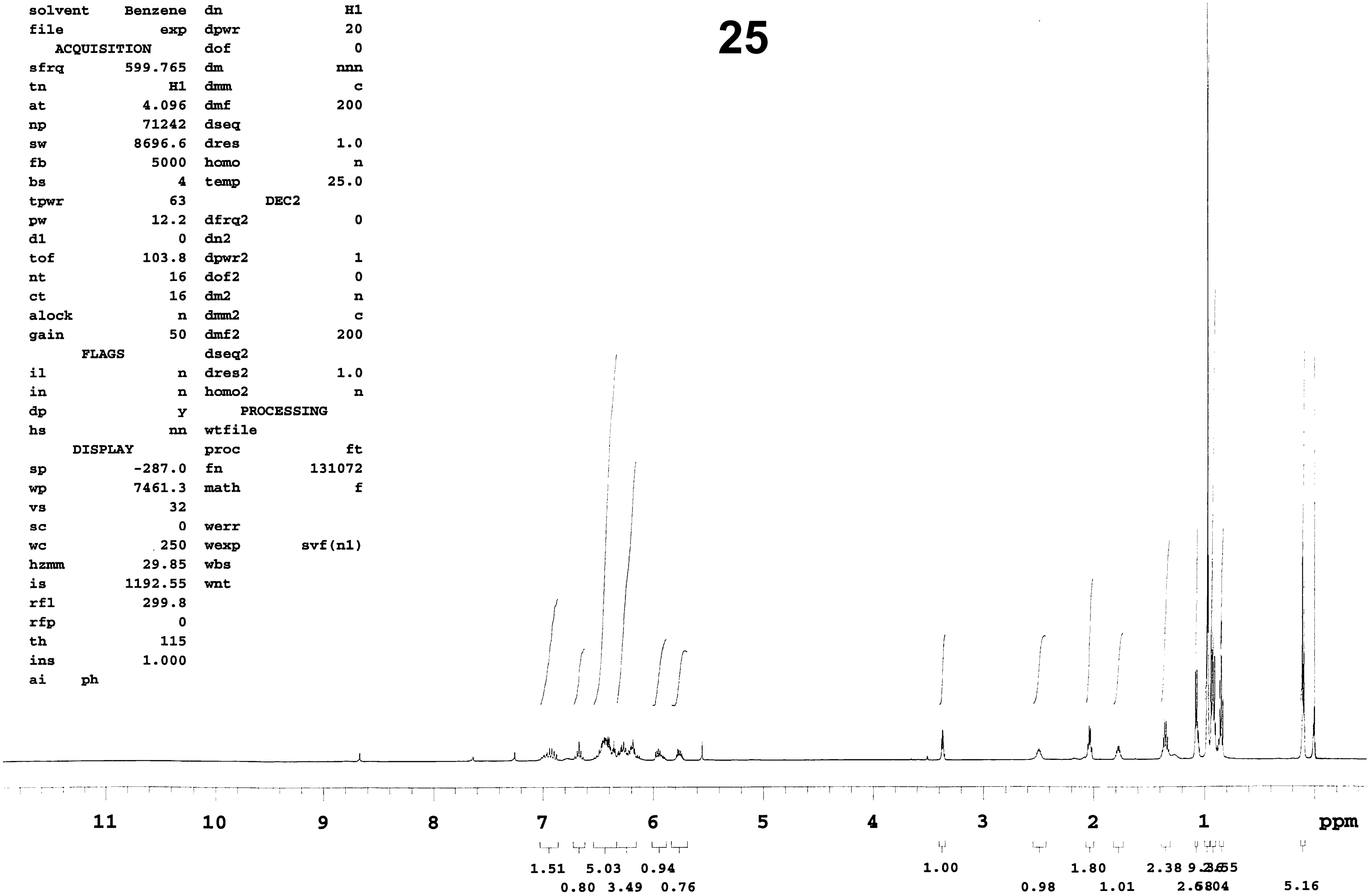

LSJV-94-13C

exp1 s2pul

SAMPLE		DEC. & VT	
date	Jun 6 2010	dfrq	599.764
solvent	Benzene	dn	H1
file	/export/home/~	dpwr	46
data/ui600/Burke/a~		dof	-1005.0
nderson/LSJV-94-13~		dm	nny
	C.fid	dmm	w
ACQUISITION		dmf	17100
sfrq	150.826	dseq	
tn	C13	dres	1.0
at	1.769	homo	n
np	122614	temp	25.0
sw	34647.0	PROCESSING	
fb	19000	lb	1.50
bs	16	wtfile	
tpwr	58	proc	ft
pw	7.0	fn	not used
d1	1.000	math	f
tof	1260.9		
nt	30000	werr	
ct	20607	wexp	
alock	n	wbs	
gain	60	wnt	
FLAGS			
il	n		
in	n		
dp	y		
hs	nn		
DISPLAY			
sp	-1466.0		
wp	34494.2		
vs	641		
sc	0		
wc	250		
hzmm	137.98		
is	500.00		
rfl	1466.5		
rfp	0		
th	68		
ins	100.000		
ai	ph		

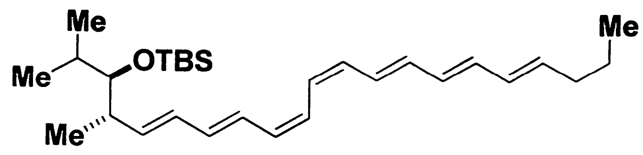

25

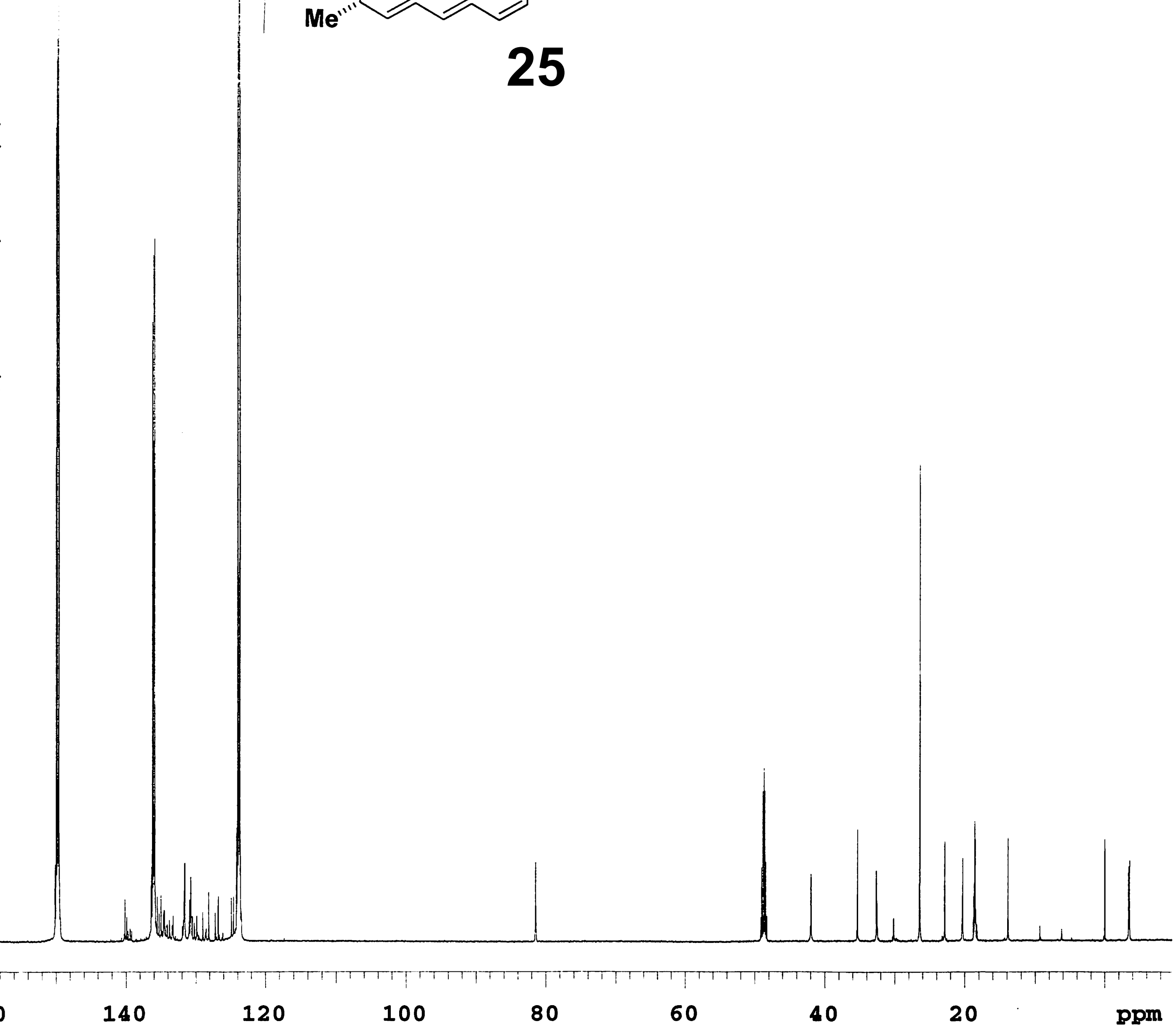

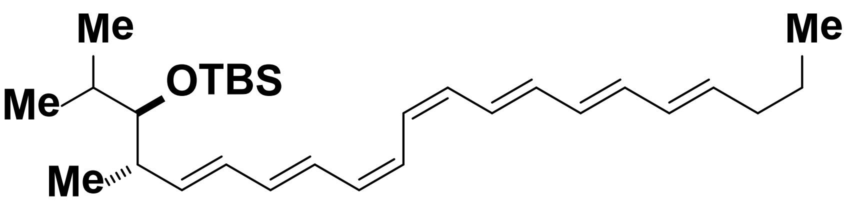

25

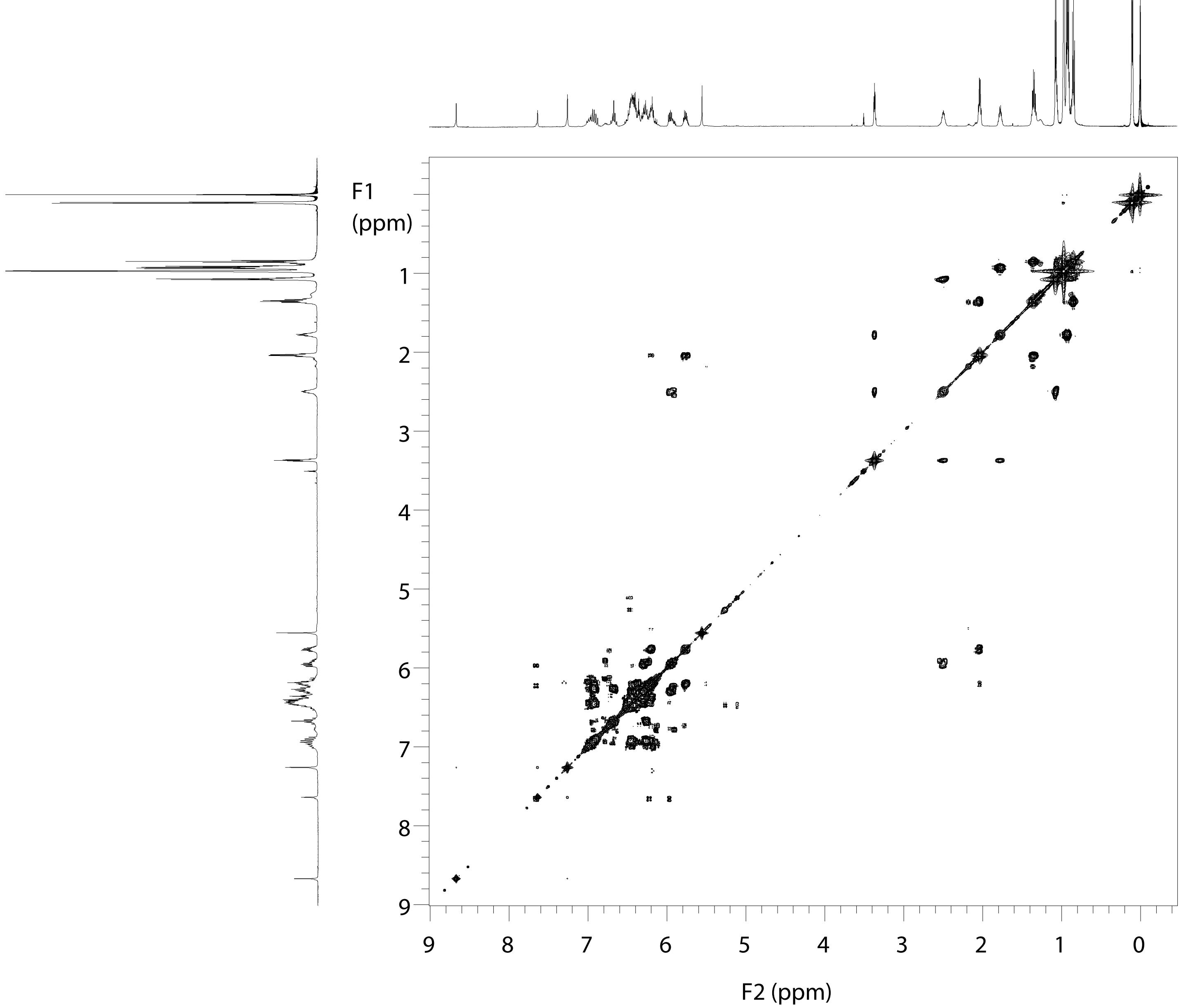

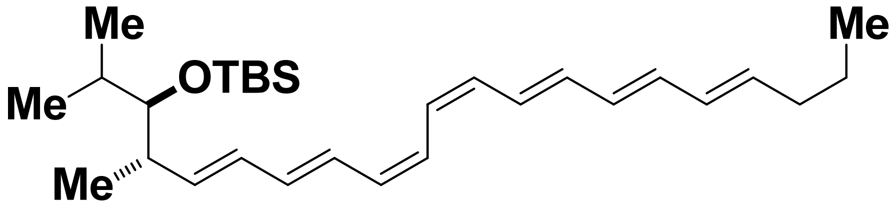

25

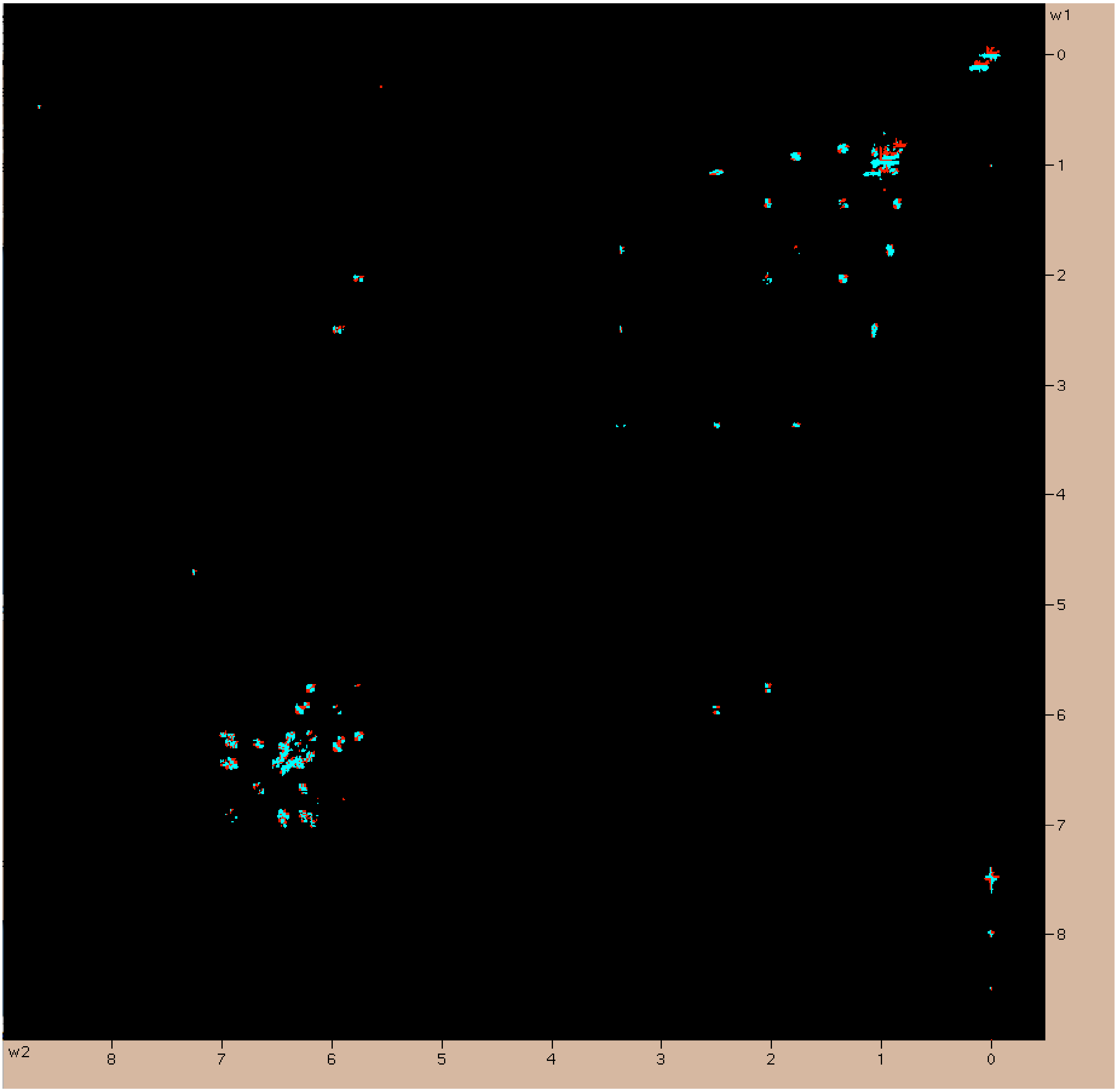

Diagonal-suppressed phase sensitive COSY spectrum of **25**

LSJV-78

exp1 std1h

SAMPLE		DEC. & VT	
date	Mar 27 2010	dn	H1
solvent	CDCl3	dof	0
file	exp	dm	nnn
ACQUISITION		dmm	c
sfrq	399.947	dmf	200
tn	H1	dpwr	20
at	4.096	PROCESSING	
np	65536	lb	not used
sw	8000.0	wtfile	
fb	4000	proc	ft
bs	16	fn	not used
tpwr	58	math	f
pw	5.8		
d1	0	werr	
tof	-425.7	wexp	svf(n1)
nt	32	wbs	
ct	32	wnt	wft('acq')
alock	n	DISPLAY	
gain	not used	sp	-356.6
FLAGS		wp	4741.0
il	n	vs	136
in	n	sc	0
dp	y	wc	250
hs	nn	hzmm	18.96
		is	1099.30
		rfl	5320.6
		rfp	2895.6
		th	2
		ins	1.000
		nm	ph

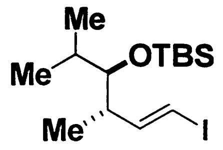

27

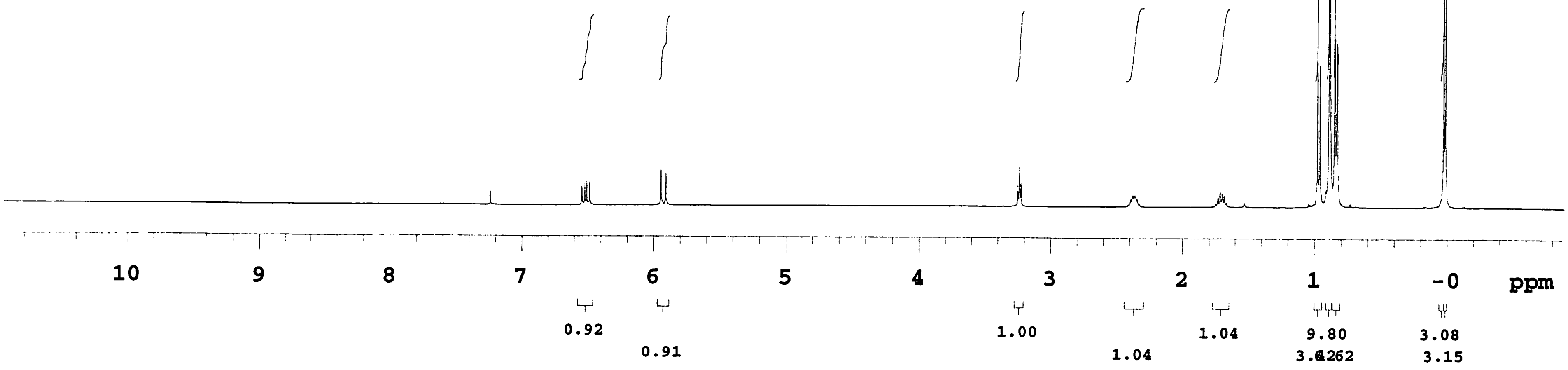

LSJV-78-13C

exp1 std13C

SAMPLE		DEC. & VT	
date	Mar 27 2010	dn	H1
solvent	CDCl3	dof	-1092.3
file	exp	dm	yyy
ACQUISITION		dmm	w
sfrq	100.578	dmf	13889
tn	C13	dpwr	43
at	1.311	PROCESSING	
np	65536	lb	1.00
sw	25000.0	wtfile	
fb	14000	proc	ft
bs	16	fn	not used
tpwr	54	math	f
pw	5.1		
d1	1.000	werr	
tof	1966.4	wexp	
nt	11111	wbs	
ct	2647	wnt	
alock	n	DISPLAY	
gain	not used	sp	-969.9
FLAGS		wp	23024.7
il	n	vs	84
in	n	sc	0
dp	y	wc	250
hs	nn	hzmm	92.10
		is	500.00
		rfl	8783.7
		rfp	7743.6
		th	6
		ins	100.000
		nm	ph

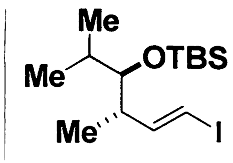

27

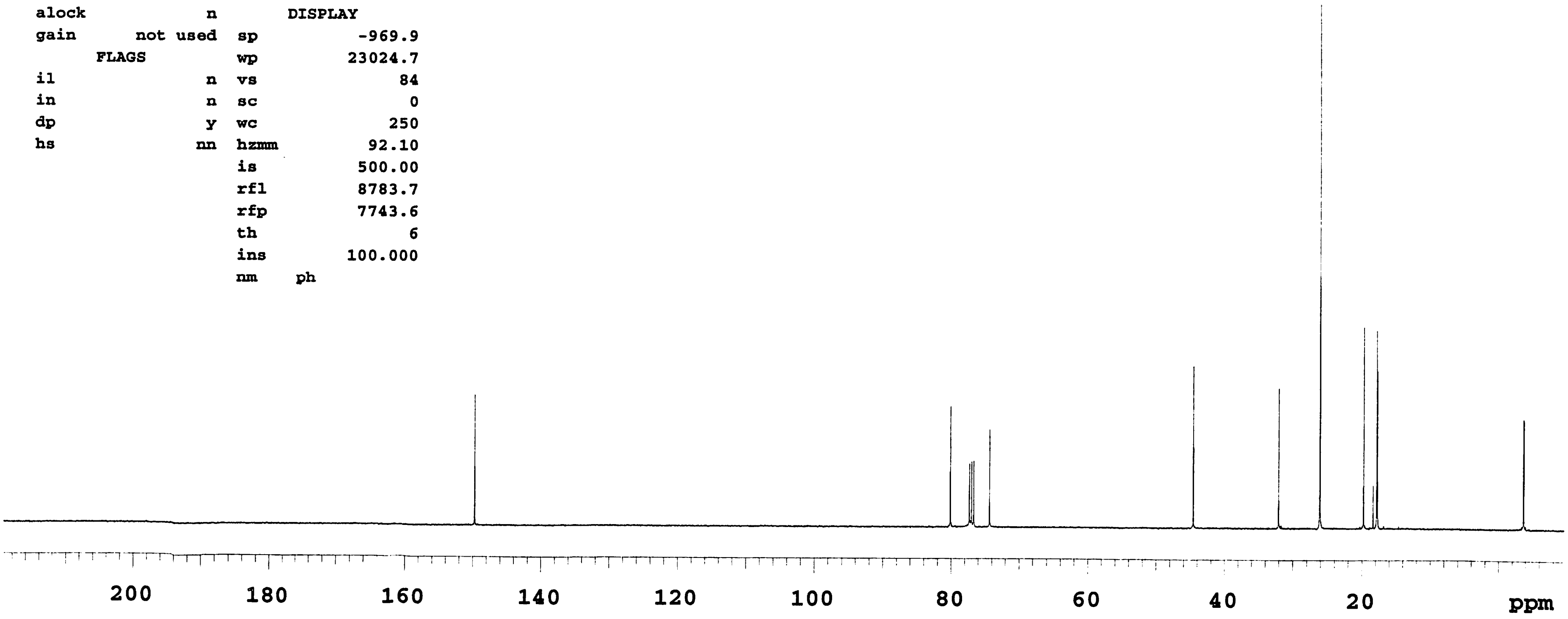

LSJV-79

exp1 std1h

SAMPLE		DEC. & VT	
date	Mar 30 2010	dn	H1
solvent	CDCl3	dof	0
file	exp	dm	nnn
ACQUISITION		dmm	c
sfrq	399.947	dmf	200
tn	H1	dpwr	20
at	4.096	PROCESSING	
np	65536	lb	not used
sw	8000.0	wtfile	
fb	4000	proc	ft
bs	16	fn	not used
tpwr	58	math	f
pw	5.8		
d1	0	werr	
tof	-425.7	wexp	svf(nl)
nt	32	wbs	
ct	32	wnt	wft('acq')
alock	n	DISPLAY	
gain	not used	sp	-372.2
FLAGS		wp	4762.2
il	n	vs	151
in	n	sc	0
dp	y	wc	250
hs	nn	hzmm	19.05
		is	2297.74
		rfl	5320.6
		rfp	2895.6
		th	20
		ins	1.000
		nm	ph

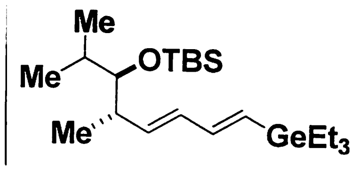

28

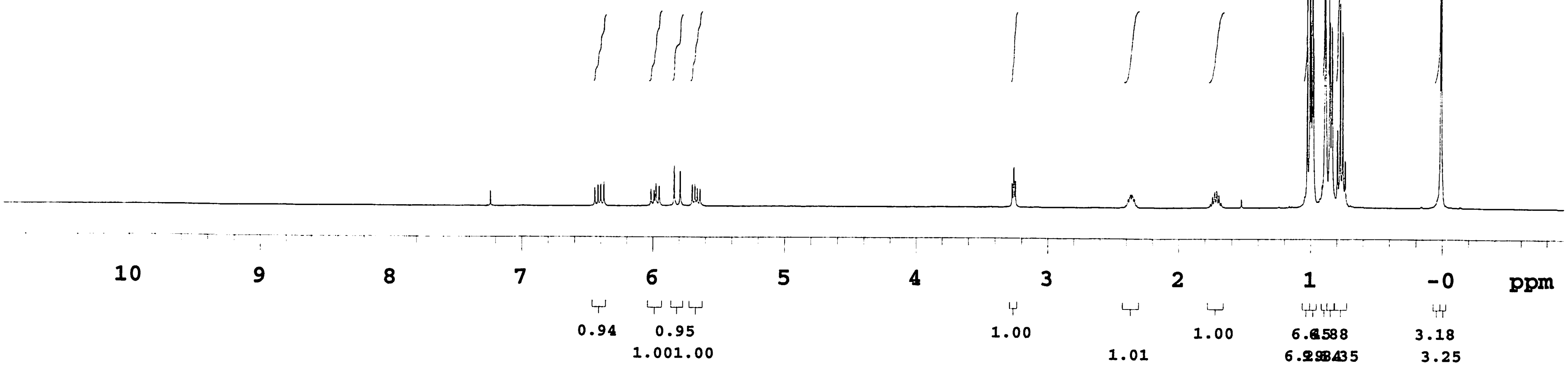

LSJV-79-13C

exp1 std13c

SAMPLE		DEC. & VT	
date	Mar 30 2010	dn	H1
solvent	CDCl3	dof	-1092.3
file	exp	dm	yyy
ACQUISITION		dmm	w
sfrq	100.578	dmf	13889
tn	C13	dpwr	43
at	1.311	PROCESSING	
np	65536	lb	1.00
sw	25000.0	wtfile	
fb	14000	proc	ft
bs	16	fn	not used
tpwr	54	math	f
pw	5.1		
d1	1.000	werr	
tof	1966.4	wexp	
nt	11111	wbs	
ct	2206	wnt	
alock	n	DISPLAY	
gain	not used	sp	-1003.5
FLAGS		wp	22985.1
il	n	vs	100
in	n	sc	0
dp	y	wc	250
hs	nn	hzmm	91.94
		is	500.00
		rfl	8781.4
		rfp	7743.6
		th	20
		ins	100.000
		nm	ph

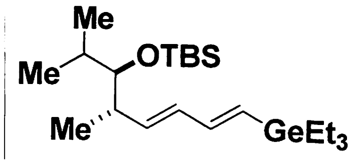

28

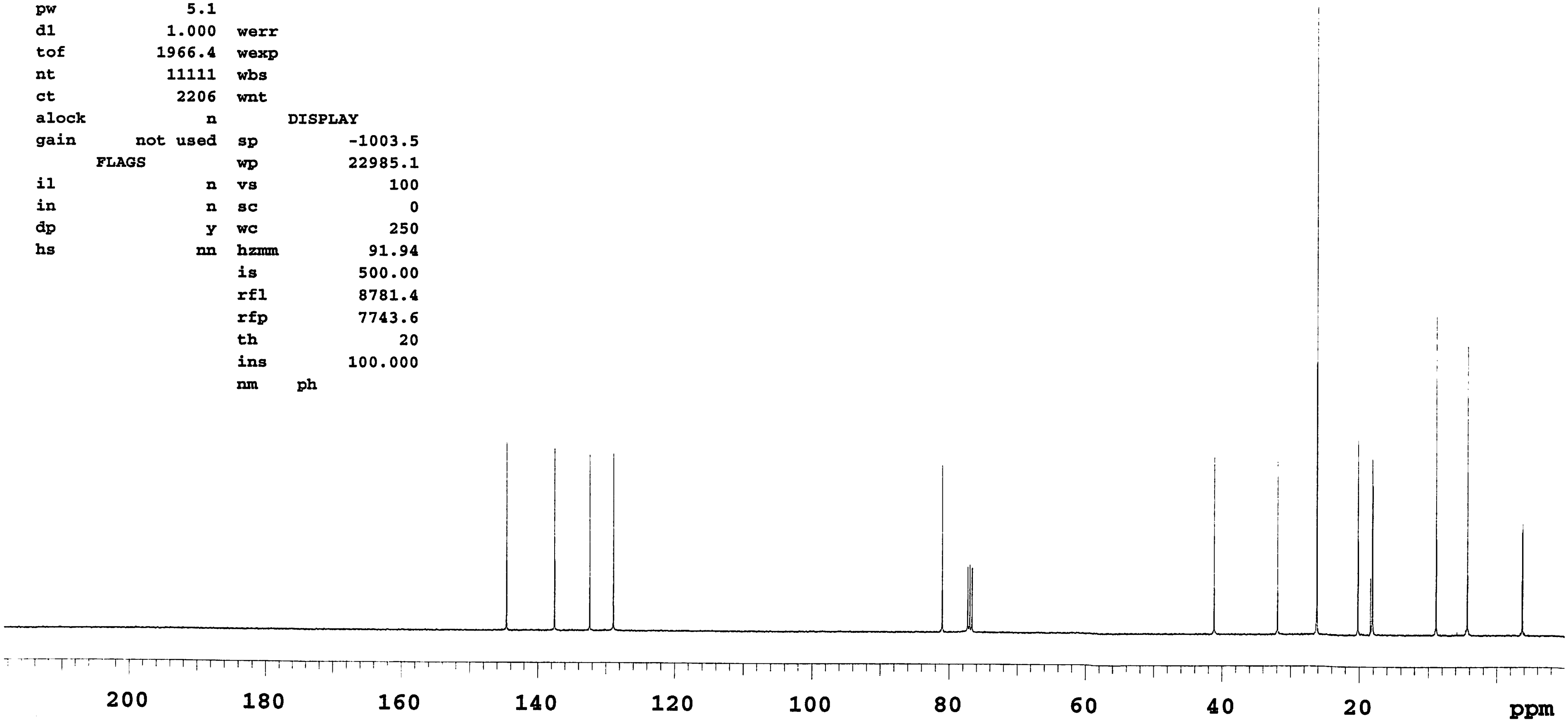

Supplement: Supplementary file 1 [file anie0049-8860-SD1.pdf]
